# Salivary proteomic profile of young healthy subjects

**DOI:** 10.3389/fmolb.2023.1327233

**Published:** 2023-11-30

**Authors:** P. Dongiovanni, M. Meroni, Gilda Aiello, A. D’Amato, N. Cenzato, S. Casati, G. Damiani, C. Fenoglio, D. Galimberti, E. Grossi, D. Prati, G. Lamorte, C. Bianco, L. Valenti, A. Soggiu, S. Zapperi, C. A. M. La Porta, M. Del Fabbro, G. M. Tartaglia

**Affiliations:** ^1^ Medicine and Metabolic Diseases, Fondazione IRCCS Cà Granda Ospedale Maggiore Policlinico, Milan, Italy; ^2^ Department of Human Science and Quality of Life Promotion, Telematic University San Raffaele, Rome, Italy; ^3^ Department of Pharmaceutical Sciences, University of Milan, Milan, Italy; ^4^ Department of Biomedical, Surgical and Dental Sciences, University of Milan, Milan, Italy; ^5^ Neurodegenerative Diseases Unit, Fondazione IRCCS Cà Granda Ospedale Maggiore Policlinico, Milan, Italy; ^6^ Villa Santa Maria Foundation, Tavernerio, Italy; ^7^ Biological Resource Center, Department of Transfusion Medicine, Fondazione IRCCS Cà Granda Ospedale Maggiore Policlinico, Milan, Italy; ^8^ Department of Physics, Center for Complexity and Biosystems, University of Milan, Milan, Italy; ^9^ Department of Environmental Science and Policy, Center for Complexity and Biosystems, University of Milan, Milan, Italy; ^10^ SC Maxillo-Facial Surgery and Dentistry, Fondazione IRCCS Cà Granda Ospedale Maggiore Policlinico, Milan, Italy

**Keywords:** saliva, proteomic analysis, biomarker, healthy subjects, liquid biopsy

## Abstract

**Background:** The incidence of noncommunicable diseases (NCDs) has been rapidly ramped up worldwide. Hence, there is an urgent need to non-invasively detect NCDs possibly by exploiting saliva as a ‘liquid biopsy’ to identify biomarkers of the health status. Since, the absence of standardized procedures of collection/analysis and the lack of normal ranges makes the use of saliva still tricky, our purpose was to outline a salivary proteomic profile which features healthy individuals.

**Methods:** We collected saliva samples from 19 young blood donors as reference population and the proteomic profile was investigated through mass-spectrometry.

**Results:** We identified 1,004 proteins of whose 243 proteins were shared by all subjects. By applying a data clustering approach, we found a set of six most representative proteins across all subjects including Coronin-1A, F-actin-capping protein subunit alpha, Immunoglobulin J chain, Prosaposin, 78 kDa glucose-regulated protein and Heat shock 70 kDa protein 1A and 1B.

**Conclusion:** All of these proteins are involved in immune system activation, cellular stress responses, proliferation, and invasion thus suggesting their use as biomarkers in patients with NCDs.

## Introduction

Communicable and noncommunicable diseases (NCDs) and mental health conditions represent the main medical and socio-economic burden in the 21st century. The prevalence of NCDs, including cancers, type 2 diabetes (T2D), cardiovascular, metabolic, and chronic respiratory diseases, has rapidly increased worldwide (Meroni et al., 2022; Mahaman et al., 2022). Scientists and funding agencies are trying their best to reduce the spread of communicable and NCDs by further advances in risk assessments, investigations, diagnosis, and treatment. However, the real challenge is the earliest detection of these diseases before the appearance of symptoms and the progression to advanced stages. In this context, it is becoming attractive to identify biomarkers in easily achievable fluids as blood, urine, sweat and saliva (Basilicata et al., 2023).

Specifically, saliva is gaining attention as liquid biopsy for the detection of several diseases, including cancer, inflammatory and metabolic disorders. Its collection is stress-free, easy, and non-invasive; therefore, it is advisable to use it as an alternative diagnostic tool to detect the systemic health status (Dongiovanni et al., 2023).

Saliva is a clear fluid directly secreted from salivary glands, mainly composed of water, but it is also extremely enriched in proteins, that may enter saliva through the blood flow by passive diffusion or active transport and in some case, there is a close relation between the salivary and serum protein concentrations (Abdul et al., 2022; Huang et al., 2023).

The proteins in saliva are important to mediate biological processes as they may participate in the defense against pathogens or contribute to the salivary buffering properties of inorganic molecules, to digestion and lubrification (Miranda et al., 2023). Traditional approaches based on biochemical and molecular procedures have highlighted the structure and function of several proteins. However, most proteins which populate saliva are still unknown thus requiring a deeper investigation of oral fluid (Hu et al., 2007). A previous salivary proteome has revealed around 3,000 proteins and peptides mainly secreted by three couples of “major” glands, namely, parotid, sub-mandibular and sub-lingual, whereas other components are derived from minor glands, gingival crevicular fluid, mucosal exudates and oral microflora (Castagnola et al., 2017). Specifically, the latter has emerged to provide novel oral biomarkers of health status since its composition may be altered by pathological conditions (Grassl et al., 2016).

The identification of the protein composition in human saliva will allow to define the presence of oral health or of a pathological condition, and to discover disease biomarkers at a very early stage using large screening populations. Indeed, salivary protein composition has been investigated in patients with different diseases, including those with oral squamous cell carcinoma (Sivadasan et al., 2020), Sjögren’s syndrome (Sembler-Møller et al., 2020), mental disorders (Iavarone et al., 2014), hepatic autoimmune (Olianas et al., 2023) and Wilson’s diseases (Cabras et al., 2015) suggesting a dysregulation of immune and stress pathways in all these disorders.

Although several studies have been focused on the salivary components among which proteins, the lack of standardized procedures of collection/analysis and the absence of reference ranges of normality make the use of saliva still tricky (Carpenter, 2013; Manjushree et al., 2022).

One of the first steps in salivary biomarker discovery for clinical implementation is the assessment of their source of variability. Thus, the evaluation of the latter from a technical and biological point of view represents a prerequisite to exploit saliva as a diagnostic and prognostic tool for biomarker identification (Hu et al., 2007; Khurshid et al., 2016). There are limited studies on intra-individual and inter-individual variability in saliva protein composition at different time-points (Ventura et al., 2018).

Here, we exploited a comprehensive proteomic approach to outline the common salivary profile which characterizes young healthy subjects on a series of saliva samples with the purpose to identify a protein pattern which features the health status by reducing as much as possible the interindividual variability using all age-matched subjects, in 3-h fasting morning conditions, with a rigorous evaluation of their clinical records.

## Materials and methods

### Volunteers’ information

A total of 19 blood donors, including 6 males and 13 females, with a median age of 28 years (range, 25–38 years) were enrolled at the Transfusion Medicine department of the IRCCS Ca’ Granda Ospedale Maggiore Policlinico, Milan, according to current national and European guidelines. All donors underwent a clinical evaluation, which included a review of their medical history, use of drugs and medications, risk factors for transmissible diseases, and a physical examination.

Data were collected and stored using a dedicated software (EMONET, GPI Italy). Donors who agreed to participate in the present study provided additional blood samples as well as a saliva sample for biobanking. Individuals with poor oral hygiene, respiratory disease, obstructive Sleep Apnea Syndrome or night snoring, congenital syndromes, pregnancy, immunocompromised patients, xerostomia patients, salivary gland diseases, blood disorders, kidney diseases, any other systemic disease, tobacco chewers and smokers were not included. Informed written consent was obtained from each patient and the study protocol was approved by the Ethical Committee of Fondazione IRCCS Cà Granda, Milan and it is conformed to the 1975 Declaration of Helsinki.

### Saliva collection

Salivary samples collection was performed by passive drooling technique by a standardized protocol (Costa et al., 2021), from one operator and samples were assessed in the same run and batch of the laboratory and by the same laboratory technician. Non-randomization was applied. Data analysis was blinded by anonymizing each patient. Saliva was collected during the morning after a quick mouthwash to remove drink and food residues. Saliva was self-collected in a sterile 50 mL plastic tube by spitting the saliva in order to obtain about 2 mL of sample. Immediately after saliva collection, samples were vortexed and centrifuged (10 min, 2,000 rpm) to remove cell debris. Samples were processed by adding protease and phosphatase inhibitors to block proteins degradation and then were frozen and stored at −80°C in the Biobank of Policlinico, Milan and then analyzed at the Unitech Omics Institute of University of Milan.

### Proteomic analysis

The protein content of each saliva sample was quantified by using the BCA assay. Then, 20 µg of proteins was diluted in 50 mM NH₄HCO₃ and then were reduced with 5 mM DL-dithiothreitol (DTT, Sigma-Aldrich) for 30 min at 52°C, then centrifuged at 500 rpm and alkylated with 15 mM iodoacetamide (Sigma-Aldrich) for 20 min in the dark at room temperature. The trypsin digestion was performed in 1:20 enzyme/protein ratio (w/w) (Trypsin Sequencing Grade; Roche, Monza, Italy) overnight at 37°C. The obtained peptides were desalted using zip-tip C18, then dry and storage at −20°C before the analysis. Tryptic peptides were analysed using a Dionex Ultimate 3,000 nano-LC system (Sunnyvale CA, United States) connected to an Orbitrap Fusion Tribrid Mass Spectrometer (ThermoFisher Scientific, Bremen, Germany) equipped with a nano-electrospray ion source (Aiello et al., 2023). Peptide mixtures were pre-concentrated onto an Acclaim PepMap 100 – 100 μm × 2 cm C18 and separated on EASY-Spray column, 15 cm × 75 µm ID packed with ThermoFisher Scientific Acclaim PepMap RSLC C18, 3 μm, 100 Å. The temperature was setting to 35 °C and the flow rate was 300 nL/min. Mobile phases were the following: 0.1% Formic acid (FA) in water (solvent A); 0.1% FA in water/acetonitrile (solvent B) with 2/8 ratio. Peptides were eluted from the column with the following gradient: 4%–28% of B for 90 min and then 28%–40% of B in 10 min, and to 95% within the following 6 min to rinse the column. Column was re-equilibrated for 20 min. Total run time was 130 min. One blank was run between triplicates to prevent sample carryover. MS spectra were collected over an m/z range of 375–1,500 at 120,000 resolutions, operating in the data dependent mode, cycle time of 3 s. Higher-energy collisional dissociation (HCD) was performed with collision energy set at 35%. Each sample was analysed in three technical triplicates (McAlister et al., 2011). Resulting MS raw data from all the technical and biological replicates were analysed by using MaxQuant software (version 1.6.2.3). Andromeda search engine was used to identify MS/MS based peptide and proteins in MaxQuant comprises a target-decoy approach with less than 1% of False Discovery Rate (FDR) setting Uniprot-*Homo Sapiens* as protein database (Ciulla et al., 2021).

Trypsin was selected as cutting enzyme, two missed cleavages and maximum five number of modifications per peptide was allowed. Methionine oxidation and acetylation (N terminus) was used as a variable modification. Carbamidomethylation was used as a fixed modification. The proteins were selected with a minimum of two peptides.

### Bioinformatics and statistical analyses

For the label-free quantification of proteins, we applied MaxLFQ algorithm.

Hierarchical clustering of the cross-correlation matrix is performed using the unweighted pair group method with arithmetic mean as implemented in python within the scipy package and the seaborn package. Clustering with the k-Medoid is implemented in python using the scikit. learn package. Principal component analysis (PCA) is implemented in python using the scikit.learn package.

## Results

**FIGURE 1:**
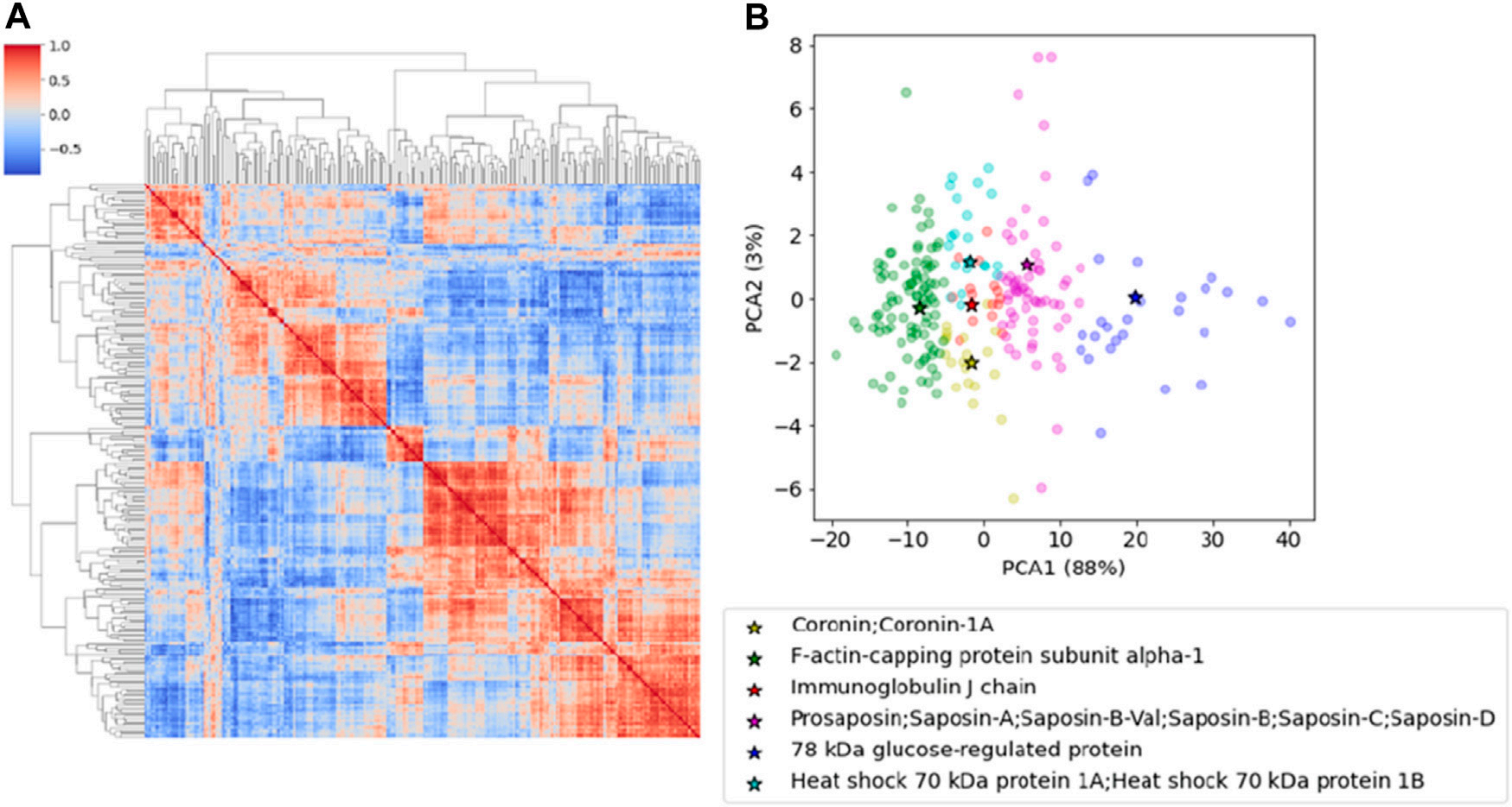
**(A)** The cross-correlation matrix of the proteins after hierarchical clustering. The red-color represents the Pearson correlation coefficient. **(B)** PCA representation of the proteins clustered according to the k-Medoid algorithm. Representative elements for each cluster are reported in the table and indicated in the plot.

We identified 1,004 proteins with a minimum of two peptides (Supplementary Table S1). We first selected proteins that had been found in at least one of the replicates of all samples and then we averaged the LFQ intensity when two replicates were present thus obtaining a set of 243 proteins shared by all subjects (Supplementary Table S2). This first screening is crucial to reduce individual fluctuations and focus on those that are commonly expressed. We then performed a cross-correlation analysis by computing pairwise Pearson coefficients among proteins which were then clustered hierarchically. This analysis applies an algorithm which permits to include in the same cluster co-regulated proteins which might be functionally related or involved in the same biological pathways. Hierarchical clustering pools together proteins with similar characteristics, creating a tree-like structure called a dendrogram, and reorder lines and columns of the covariance matrix so that co-expressed proteins are showed in close proximity (Figure 1A).

Proteins were clustered using the k-Medoid algorithm with k = 6, as suggested by Figure 1A that shows a set of six blocks along the diagonal. The most representative protein for each cluster was then associated with the corresponding medoid. The list includes Coronin-1A (Coro1A), F-actin-capping protein subunit alpha (Capza1), Immunoglobulin J chain (Jchain), Prosaposin (Psap), 78 kDa glucose-regulated protein (Grp78) and Heat shock 70 kDa protein 1A and 1B (Hspa1a/1b) (Table 1). The results can be represented graphically using the first two components of PCA, as shown in Figure 1B. A two-dimensional representation is appropriate since the first two components of the PCA explain 91% of the variance in the data.

**TABLE 1:** List of the six most representative proteins identified in all samples.

Accession number	Protein names	Gene name	N peptides	Mol. weight (KDa)
A0A024R611	Coronin; Coronin-1A	CORO1A	19	51.03
P52907	F-actin-capping protein subunit alpha-1	CAPZA1	13	32.92
P01591	Immunoglobulin J chain	IGJ	16	18.1
A0A024QZQ2	Prosaposin	PSAP	21	58.11
V9HWB4	78 kDa glucose-regulated protein	HEL-S-89n	30	72.33
P0DMV8	Heat shock 70 kDa protein 1A; Heat shock 70 kDa protein 1B	HSPA1A	33	70.05

## Discussion

The possibility to exploit saliva as biological fluid to detect systemic disorders is becoming increasingly attractive. Indeed, salivary collection is non-intrusive, applicable for mass screening and in all-age subjects. Saliva carries a burgeoning number of proteins, which may be representative of global health condition or pathological status, thus constituting appealing biomarkers.

However, the small sample size, the differences in experimental plan of sample collection/preparation and the lack of references in healthy subjects still make the salivary-derived biomarkers not easily exploitable. In addition, different approaches may introduce a further technical variability in the proteomic analysis. Castagnola and others widely addressed the salivary proteome in the last years, critically comparing two methods which reveal qualitative/quantitative differences across samples occurring in a selected pool of salivary proteins or analyze fragmented proteins by enzymatic digestion and giving an overview of the major families of salivary proteins (Castagnola et al., 2012; Messana et al., 2015; Castagnola et al., 2017; Di Pietro et al., 2023).

In details, these authors extensively faced this topic reporting the saliva composition across the lifetime, both in health and disease (Scarano et al., 2010). Even more, other authors further investigated the human salivary proteome in relation to that of the diverse oral microbial communities, also referred to as meta-proteomics (Grassl et al., 2016).

We selected 243 proteins that were detected in all subjects including amylases (Alpha-amylase-1), mucins (Mucin-5B, Mucin-7) and cystatins (Cystatin-B, Cystatin-C, Cystatin-D, Cystatin-S, Cystatin-SA, Cystatin-SN) that are essential for the maintenance of the oral health as previously reported (Scarano et al., 2010). We found additional amylases, mucins and cystatins although they were not shared by all the subjects thus confirming the reliability of our technical approach.

Next, by applying a clustering method we identified 6 clusters which include amylases, mucins and cystatins. However the most representative proteins in the six clusters were Coro1A, Capza1, Jchain, Psap, Grp78 and Hspa1a/1b.

Coro1A which belongs to the Coronins family is an actin-binding protein that plays important roles in cell signaling, migration, phagocytosis, and vesicle trafficking (Uetrecht and Bear, 2006). Plasma membrane of leucocytes links to the actin cytoskeleton through Coro1a and its downregulation alters innate immunity. It has been shown to be specifically involved in T-lymphocyte migration and survival, to the point that *Coro1a* ^*−/−*^ mice suppress T cell responses and reduce allograft rejection (Jayachandran et al., 2019). Accordingly, it has been found to be suppressed in salivary samples of patients with different tumors, among which breast and renal cancers (Zhang et al., 2020; Sinha et al., 2023). Coro1A is a regulator of β2-integrin that interacts with CD18 for the induction of polymorphonuclear neutrophils (PMNs) rolling, adhesion and invasion (Pick et al., 2017).

Capping protein, a protein complex referred to CapZ is as actin-binding complex which regulates the cytoskeleton remodeling and cell motility (Zhang et al., 2021). CAPZA1 is the α1 subunit of this complex and has been reported to regulate the autolysosome formation and its deregulation leads to higher risk of gastric cancer and metastasis (Tsugawa et al., 2019). Moreover, Huang and colleagues demonstrated that CAPZA1 inhibits epithelial mesenchymal transition (EMT) in hepatocellular carcinoma (HCC) cells by regulating actin filament assembly, thereby reducing the invasion and migration abilities of HCC cells. Therefore, it has been suggested that CAPZA1 could be a biomarker to determine the prognosis of HCC patients (Huang et al., 2017).

The J-chain is mainly expressed in both T and B cells during their early development and after the differentiation its expression is maintained in B cells producing IgA and IgM. J-chain is essential for the polymerization of IgA and IgM antibodies and for their secretion. J-chain is localized in minor salivary glands of the lip and palate where it is involved in the transport of immunoglobulin A (sIgA) into the saliva (Sumi et al., 1988). During malignant transformation J-chain expression is dramatically changed in the context of lung cancer (Slizhikova et al., 2007).

Psap is a conserved precursor of Saponins A, B, C and D and participates in the lysosomal degradation of glycosphingolipids. Genetic mutations in any of the saponins lead to lysosomal storage diseases. Although Psap is mainly localized in lysosomes, it can also be secreted, and it has been found in several fluids including serum (Hineno et al., 1991). Psap is expressed in salivary glands and its levels differ based on the type of gland, acinar cells, age, and sex (Islam et al., 2018). It has been demonstrated that *Psap* ^*−/−*^ mice develop inflammation, plaque formation and show a reduction of oxidative phosphorylation thus pointing out Psap as a potential therapeutic target for atherosclerosis (van Leent et al., 2021). Finally, it has been found reduced in salivary samples obtained from patients with T2D (Jia et al., 2021).

GRP78 is a member of the Hsp70 family, it is constitutively expressed in the endoplasmic reticulum (ER) where acts as chaperone involved in the correct folding of nascent proteins (Haas, 1994). GRP78 has also been implicated in non-classic antigen presentation and regulation of cytotoxic T cell responses. Moreover, under stress conditions, it migrates out of the ER to the cell surface thus representing a marker of cell stress. GRP78 can also be secreted, and it has been described its upregulation by anoxia, hypoglycemia and ROS (Giusti et al., 2010). It is significantly increased in several tumors where favors cell proliferation and angiogenesis, and it has been found to be increased in calcified arteries (Kaira et al., 2016; Furmanik et al., 2021).

Hsp70 is a ubiquitous heat shock protein well known for its chaperone activity in folding and remodeling processes. It localizes both in membrane and extracellularly and, depending on its subcellular location, it may stimulate the immune response. During stress conditions such as inflammation, infections, and oncological diseases, Hsp70 has been found in serum from which it may diffuse passively in saliva (Fábián et al., 2003). In the latter Hsp70 has been linked to a host defense mechanism by activating TLR4 and TNFα (Rinecker et al., 2022).

Overall, proteomic analysis of salivary samples of young healthy subjects allowed us to identify a set of six proteins that are most representative of all the proteins detectable in saliva. Since all these proteins are involved in pivotal physiological pathways regulating the immune system and inflammation, we could speculate that aberrancies in their expressions may represent a hallmark of pathological states, thus opening the possibility to be pointed out as novel non-invasive biomarkers useful for precision and personalized medicine.

## Data Availability

The original contributions presented in the study are included in the article/Supplementary Materials, further inquiries can be directed to the corresponding author.

## References

[B1] Abdul N. S. AlGhannam S. M. Almughaiseeb A. A. Bindawoad F. A. Alduraibi S. M. Shenoy M. (2022). A review on salivary constituents and their role in diagnostics. Bioinformation 18 (10), 1021–1028. 10.6026/973206300181021 37693919 PMC10492514

[B2] Aiello G. Rescigno F. Meloni M. Zoanni B. Aldini G. Carini M. (2023). The effect of carnosine on UVA-induced changes in intracellular signaling of human skin fibroblast spheroids. Antioxidants (Basel). 12 (2), 300. 10.3390/antiox12020300 36829859 PMC9951876

[B3] Basilicata M. Pieri M. Marrone G. Nicolai E. Di Lauro M. Paolino V. (2023). Saliva as biomarker for oral and chronic degenerative non-communicable diseases. Metabolites 13 (8), 889. 10.3390/metabo13080889 37623833 PMC10456419

[B4] Cabras T. Sanna M. Manconi B. Fanni D. Demelia L. Sorbello O. (2015). Proteomic investigation of whole saliva in Wilson's disease. J. Proteomics 128, 154–163. 10.1016/j.jprot.2015.07.033 26254010

[B5] Carpenter G. H. (2013). The secretion, components, and properties of saliva. Annu. Rev. Food Sci. Technol. 4, 267–276. 10.1146/annurev-food-030212-182700 23464573

[B6] Castagnola M. Cabras T. Iavarone F. Vincenzoni F. Vitali A. Pisano E. (2012). Top-down platform for deciphering the human salivary proteome. J. Matern. Fetal Neonatal Med. 25 (Suppl. 5), 27–43. 10.3109/14767058.2012.714647 23025766

[B7] Castagnola M. Scarano E. Passali G. C. Messana I. Cabras T. Iavarone F. (2017). Salivary biomarkers and proteomics: future diagnostic and clinical utilities. Acta Otorhinolaryngol. Ital. 37 (2), 94–101. 10.14639/0392-100X-1598 28516971 PMC5463528

[B8] Ciulla M. M. Re D. Gilardoni E. D'Amato A. Altomare A. Baron G. (2021). PHoral: effects of carnosine supplementation on quantity/quality of oral salivae in healthy volunteer and in subjects affected by common oral pathologies. Med. Baltim. 100 (25), e26369. 10.1097/MD.0000000000026369 PMC8238340 34160409

[B9] Costa M. M. Benoit N. Saby F. Pradines B. Granjeaud S. Almeras L. (2021). Optimization and standardization of human saliva collection for MALDI-TOF MS. Diagn. (Basel) 11 (8), 1304. 10.3390/diagnostics11081304 PMC8392517 34441239

[B10] Di Pietro L. Boroumand M. Lattanzi W. Manconi B. Salvati M. Cabras T. (2023). A catalog of coding sequence variations in salivary proteins' genes occurring during recent human evolution. Int. J. Mol. Sci. 24 (19), 15010. 10.3390/ijms241915010 37834461 PMC10573131

[B11] Dongiovanni P. Meroni M. Casati S. Goldoni R. Thomaz D. V. Kehr N. S. (2023). Salivary biomarkers: novel noninvasive tools to diagnose chronic inflammation. Int. J. Oral Sci. 15 (1), 27. 10.1038/s41368-023-00231-6 37386003 PMC10310701

[B12] Fábián T. K. Gáspár J. Fejérdy L. Kaán B. Bálint M. Csermely P. (2003). Hsp70 is present in human saliva. Med. Sci. Monit. 9 (1), Br62–5. 12552239

[B13] Furmanik M. van Gorp R. Whitehead M. Ahmad S. Bordoloi J. Kapustin A. (2021). Endoplasmic reticulum stress mediates vascular smooth muscle cell calcification via increased release of Grp78 (Glucose-Regulated protein, 78 kDa)-Loaded extracellular vesicles. Arterioscler. Thromb. Vasc. Biol. 41 (2), 898–914. 10.1161/ATVBAHA.120.315506 33297752 PMC7837691

[B14] Giusti L. Baldini C. Ciregia F. Giannaccini G. Giacomelli C. De Feo F. (2010). Is GRP78/BiP a potential salivary biomarker in patients with rheumatoid arthritis? Proteomics Clin. Appl. 4 (3), 315–324. 10.1002/prca.200900082 21137052

[B15] Grassl N. Kulak N. A. Pichler G. Geyer P. E. Jung J. Schubert S. (2016). Ultra-deep and quantitative saliva proteome reveals dynamics of the oral microbiome. Genome Med. 8 (1), 44. 10.1186/s13073-016-0293-0 27102203 PMC4841045

[B17] Haas I. G. (1994). BiP (GRP78), an essential hsp70 resident protein in the endoplasmic reticulum. Experientia 50 (11-12), 1012–1020. 10.1007/BF01923455 7988659

[B18] Hineno T. Sano A. Kondoh K. Ueno S. Kakimoto Y. Yoshida K. (1991). Secretion of sphingolipid hydrolase activator precursor, prosaposin. Biochem. Biophys. Res. Commun. 176 (2), 668–674. 10.1016/s0006-291x(05)80236-0 2025281

[B19] Hu S. Loo J. A. Wong D. T. (2007). Human saliva proteome analysis. Ann. N. Y. Acad. Sci. 1098, 323–329. 10.1196/annals.1384.015 17435138

[B20] Huang D. Cao L. Zheng S. (2017). CAPZA1 modulates EMT by regulating actin cytoskeleton remodelling in hepatocellular carcinoma. J. Exp. Clin. Cancer Res. 36 (1), 13. 10.1186/s13046-016-0474-0 28093067 PMC5240199

[B21] Huang Z. Yang X. Huang Y. Tang Z. Chen Y. Liu H. (2023). Saliva - a new opportunity for fluid biopsy. Clin. Chem. Lab. Med. 61 (1), 4–32. 10.1515/cclm-2022-0793 36285724

[B22] Iavarone F. Melis M. Platania G. Cabras T. Manconi B. Petruzzelli R. (2014). Characterization of salivary proteins of schizophrenic and bipolar disorder patients by top-down proteomics. J. Proteomics 103, 15–22. 10.1016/j.jprot.2014.03.020 24690516

[B23] Islam F. Khan M. S. I. Nabeka H. Saito S. Li X. Shimokawa T. (2018). Prosaposin and its receptors are differentially expressed in the salivary glands of male and female rats. Cell Tissue Res. 373 (2), 439–457. 10.1007/s00441-018-2835-9 29656342

[B24] Jayachandran R. Gumienny A. Bolinger B. Ruehl S. Lang M. J. Fucile G. (2019). Disruption of Coronin 1 signaling in T cells promotes allograft tolerance while maintaining anti-pathogen immunity. Immunity 50 (1), 152–165. 10.1016/j.immuni.2018.12.011 30611611

[B25] Jia S. Y. Zhang Y. L. Sun X. Y. Yuan C. Zheng S. G. (2021). Impact of the glycemic level on the salivary proteome of middle-aged and elderly people with type 2 diabetes mellitus: an observational study. Front. Mol. Biosci. 8, 790091. 10.3389/fmolb.2021.790091 34957219 PMC8703016

[B26] Kaira K. Toyoda M. Shimizu A. Shino M. Sakakura K. Takayasu Y. (2016). Expression of ER stress markers (GRP78/BiP and PERK) in adenoid cystic carcinoma. Acta Otolaryngol. 136 (1), 1–7. 10.3109/00016489.2015.1083120 26366837

[B27] Khurshid Z. Zohaib S. Najeeb S. Zafar M. S. Slowey P. D. Almas K. (2016). Human saliva collection devices for proteomics: an update. Int. J. Mol. Sci. 17 (6), 846. 10.3390/ijms17060846 27275816 PMC4926380

[B28] Mahaman Y. A. R. Embaye K. S. Huang F. Li L. Zhu F. Wang J. Z. (2022). Biomarkers used in Alzheimer's disease diagnosis, treatment, and prevention. Ageing Res. Rev. 74, 101544. 10.1016/j.arr.2021.101544 34933129

[B29] Manjushree R. Anandakrishna L. Prasad Ks K. Shetty A. K. (2022). Evaluation of salivary components and dental plaque in relation to dental caries status in type 1 diabetes mellitus. Int. J. Clin. Pediatr. Dent. 15 (Suppl. 2), S121–s125. 10.5005/jp-journals-10005-2325 35645528 PMC9108845

[B30] McAlister G. C. Phanstiel D. H. Brumbaugh J. Westphall M. S. Coon J. J. (2011). Higher-energy collision-activated dissociation without a dedicated collision cell. Mol. Cell Proteomics 10 (5), O111.009456. 10.1074/mcp.O111.009456 PMC3098599 21393638

[B16] Meroni M. Longo M. Lombardi R. Paolini E. Macchi C. Corsini A. (2022). Low lipoprotein(a) levels predict hepatic fibrosis in patients with nonalcoholic fatty liver disease. Hepatol. Commun. 6 (3), 535–549. 10.1002/hep4.1830 34677008 PMC8870034

[B31] Messana I. Cabras T. Iavarone F. Manconi B. Huang L. Martelli C. (2015). Chrono-proteomics of human saliva: variations of the salivary proteome during human development. J. Proteome Res. 14 (4), 1666–1677. 10.1021/pr501270x 25761918

[B32] Miranda L. F. B. Lima C. V. Pagin R. Costa R. C. Pereira M. M. A. de Avila E. D. (2023). Effect of processing methods of human saliva on the proteomic profile and protein-mediated biological processes. J. Proteome Res. 22 (3), 857–870. 10.1021/acs.jproteome.2c00652 36779809

[B33] Olianas A. Guadalupi G. Cabras T. Contini C. Serrao S. Iavarone F. (2023). Top-Down proteomics detection of potential salivary biomarkers for autoimmune liver diseases classification. Int. J. Mol. Sci. 24 (2), 959. 10.3390/ijms24020959 36674470 PMC9866740

[B34] Pick R. Begandt D. Stocker T. J. Salvermoser M. Thome S. Böttcher R. T. (2017). Coronin 1A, a novel player in integrin biology, controls neutrophil trafficking in innate immunity. Blood 130 (7), 847–858. 10.1182/blood-2016-11-749622 28615221

[B35] Rinecker J. Roesch R. Krippgans S. Nieberler M. Stark L. Stangl S. (2022). Comparing TIMP-1 and Hsp70 in blood and saliva as potential prognostic markers in HNSCC. Biomedicines 10 (12), 3225. 10.3390/biomedicines10123225 36551979 PMC9775946

[B36] Scarano E. Fiorita A. Picciotti P. M. Passali G. C. Calò L. Cabras T. (2010). Proteomics of saliva: personal experience. Acta Otorhinolaryngol. Ital. 30 (3), 125–130. 20948587 PMC2914523

[B37] Sembler-Møller M. L. Belstrøm D. Locht H. Pedersen A. M. L. (2020). Proteomics of saliva, plasma, and salivary gland tissue in Sjögren's syndrome and non-Sjögren patients identify novel biomarker candidates. J. Proteomics 225, 103877. 10.1016/j.jprot.2020.103877 32540407

[B38] Sinha I. Fogle R. L. Gulfidan G. Stanley A. E. Walter V. Hollenbeak C. S. (2023). Potential early markers for breast cancer: a proteomic approach comparing saliva and serum samples in a pilot study. Int. J. Mol. Sci. 24 (4), 4164. 10.3390/ijms24044164 36835577 PMC9966955

[B39] Sivadasan P. Gupta M. K. Sathe G. Sudheendra H. V. Sunny S. P. Renu D. (2020). Salivary proteins from dysplastic leukoplakia and oral squamous cell carcinoma and their potential for early detection. J. Proteomics 212, 103574. 10.1016/j.jprot.2019.103574 31706945

[B40] Slizhikova D. K. Zinov'eva M. V. Kuz'min D. V. Snezhkov E. B. Shakhparonov M. I. Dmitriev R. I. (2007). Decrease in expression of human J-chain in lung squamous cell cancer and adenocarcinoma. Mol. Biol. Mosk. 41 (4), 659–665. 17936986

[B41] Sumi Y. Nagura H. Kaneda T. Oka T. (1988). Immunoelectron microscopical localization of immunoglobulins, secretory component and J chain in the human minor salivary glands. J. Oral Pathol. 17 (8), 390–395. 10.1111/j.1600-0714.1988.tb01303.x 3146624

[B42] Tsugawa H. Mori H. Matsuzaki J. Sato A. Saito Y. Imoto M. (2019). CAPZA1 determines the risk of gastric carcinogenesis by inhibiting *Helicobacter pylori* CagA-degraded autophagy. Autophagy 15 (2), 242–258. 10.1080/15548627.2018.1515530 30176157 PMC6333452

[B43] Uetrecht A. C. Bear J. E. (2006). Coronins: the return of the crown. Trends Cell Biol. 16 (8), 421–426. 10.1016/j.tcb.2006.06.002 16806932

[B44] van Leent M. M. T. Beldman T. J. Toner Y. C. Lameijer M. A. Rother N. Bekkering S. (2021). Prosaposin mediates inflammation in atherosclerosis. Sci. Transl. Med. 13 (584), eabe1433. 10.1126/scitranslmed.abe1433 33692130 PMC8209679

[B45] Ventura T. Ribeiro N. R. Dionizio A. S. Sabino I. T. Buzalaf M. A. R. (2018). Standardization of a protocol for shotgun proteomic analysis of saliva. J. Appl. Oral Sci. 26, e20170561. 10.1590/1678-7757-2017-0561 29898185 PMC6007968

[B46] Zhang X. L. Wu Z. Z. Xu Y. Wang J. G. Wang Y. Q. Cao M. Q. (2020). Saliva proteomic analysis reveals possible biomarkers of renal cell carcinoma. Open Chem. 18 (1), 918–926. 10.1515/chem-2020-0048

[B47] Zhang Y. G. Niu J. T. Wu H. W. Si X. L. Zhang S. J. Li D. H. (2021). Actin-binding proteins as potential biomarkers for chronic inflammation-induced cancer diagnosis and therapy. Anal. Cell Pathol. (Amst) 2021, 6692811. 10.1155/2021/6692811 34194957 PMC8203385

