# Supplementary material for: Salivary proteomic profile of young healthy subjects
Source: Front Mol Biosci. 2023 Nov 30;10:1327233. doi: 10.3389/fmolb.2023.1327233 (PMC10720708; doi:10.3389/fmolb.2023.1327233)
Supplement: Supplementary file 1 [file DataSheet2.pdf]

N: Mol. weight [kDa]	T: Protein IDs	T: Majority protein IDs	T: Protein names	T: Gene names
116,72	A0A024QZN4;V9HWK2;P18206;B4E3(	A0A024QZN4;V9HWK2;P18206;B4E3	Vinculin	VCL;HEL114
58,112	A0A024QZQ2;P07602;Q53FJ5;C9JIZ6;	A0A024QZQ2;P07602;Q53FJ5;C9JIZ6	Prosaposin;Saposin-A;Sapos	PSAP
13,989	Q99879;U3KQK0;A0A024QZZ7;B4DR5	Q99879;U3KQK0;A0A024QZZ7;B4DR	Histone H2B type 1-M;Histo	HIST1H2BM;HIST1H2BN;HI
32,922	P52907;A0A024R0E5;A8K0T9	P52907;A0A024R0E5;A8K0T9	F-actin-capping protein sub	CAPZA1
117,85	A0A024R1A3;P22314;B4DDE4;B4DL6	A0A024R1A3;P22314;B4DDE4;B4DL(	Ubiquitin-like modifier-acti	UBE1;UBA1
226,53	P35579;A0A024R1N1;A0A0U4BW16;(	P35579;A0A024R1N1;A0A0U4BW16	Myosin-9	MYH9
21,429	P15153;A0A024R1P2;B1AH77;B1AH8	P15153;A0A024R1P2;B1AH77;B1AH	Ras-related C3 botulinum tc	RAC2
41,92	A0A024R1Z6;Q99536;B3KUF8;K7ESA3	A0A024R1Z6;Q99536;B3KUF8	Synaptic vesicle membrane	VAT1
42,1	A0A024R2B6;P36952;B4DWS9;C9JLN	A0A024R2B6;P36952;B4DWS9	Serpin B5	SERPINB5
21,768	P61586;A0A024R324;A0A024R0I3;P0	P61586;A0A024R324;A0A024R0I3;P	Transforming protein RhoA;	RHOA;hCG_2043376;RHOC
30,777	A0A024R3E3;P02647;F8W696;Q9Y35	A0A024R3E3;P02647;F8W696	Apolipoprotein A-I;Proapoli	APOA1
47,168	P06733;A0A024R4F1;E2DRY6;K7EM9(	P06733;A0A024R4F1;E2DRY6	Alpha-enolase;Enolase	ENO1
31,969	Q0QF37;Q75MT9;Q6FHZ0;A0A024R4K	Q0QF37;Q75MT9;Q6FHZ0;A0A024R4	Malate dehydrogenase;Mala	MDH2
16,142	A0A024R528;P18510	A0A024R528;P18510	Interleukin-1 receptor anta	IL1RN
51,026	A0A024R611;P31146;Q59G88;H3BRY	A0A024R611;P31146;Q59G88;H3BR	Coronin;Coronin-1A	CORO1A
13,897	Q86TY5;Q6FGL0;A0A024R693;Q59FR	Q86TY5;Q6FGL0;A0A024R693;Q59FF	Galectin;Galectin-3	LGALS3;hCG_22119
13,078	G3V2V8;J3KMY5;Q53HV6;A0A024R6C	G3V2V8;J3KMY5;Q53HV6;A0A024R6	Epididymal secretory protei	NPC2
55,52	A0A024R718;P43490;A0A0C4DFS8;Q(	A0A024R718;P43490	Nicotinamide phosphoribo	PBEF1;NAMPT
19,25	A0A024R8D7;P31025;Q5VSP4	A0A024R8D7;P31025	Lipocalin-1	LCN1
57,116	P07237;A0A024R8S5;H7BZ94;B4DUA	P07237;A0A024R8S5;H7BZ94;B4DU	Protein disulfide-isomerase	P4HB
36,748	A0A024R9G4;Q9NUQ9;Q68D08;E5RI1	A0A024R9G4;Q9NUQ9;Q68D08	Protein FAM49B	FAM49B;DKFZp686B04128
21,258	A0A024RAE4;B4E1U9;P60953;A0A024	A0A024RAE4;B4E1U9;P60953;A0A02	Cell division control proteir	CDC42;hCG_39634
47,371	P61158;A0A024RAI1;B4DXW1;Q59FV	P61158;A0A024RAI1;B4DXW1;Q59F	Actin-related protein 3	ACTR3
22,988	A0A024RAS5;P52566;B2R4F3;F5H6Q(	A0A024RAS5;P52566;B2R4F3;F5H6C	Rho GDP-dissociation inhib	ARHGDIB
107,53	P32926;A0A024RC30;Q7Z2Z5	P32926;A0A024RC30	Desmoglein-3	DSG3
49,416	A0A024RC87;A0A140VJT8;P13489;H(	A0A024RC87;A0A140VJT8;P13489;H	Ribonuclease inhibitor	RNH1
75,207	A0A024RDE1;Q14515;Q8N4S1;B7ZB6	A0A024RDE1;Q14515;Q8N4S1;B7ZB(	SPARC-like protein 1	SPARCL1
70,289	V9HWJ7;A0A024RDT4;P13796;Q53FI	V9HWJ7;A0A024RDT4;P13796;Q53F	Plastin-2	HEL-S-37;LCP1
50,222	V9HW75;A0A087WSV8;P80303;Q2L6	V9HW75;A0A087WSV8;P80303;Q2L(	Nucleobindin-2;Nesfatin-1	HEL-S-109;NUCB2;Nucb2
8,641	Q07654;A0A0A6YYJ4;X6R3S7	Q07654;A0A0A6YYJ4;X6R3S7	Trefoil factor 3	TFF3
19,6	A0A0C4DGN4;C3PTT6;G8H6I3;Q96DA	A0A0C4DGN4;C3PTT6;G8H6I3;Q96D	Zymogen granule protein 16	ZG16B;PAUF;EECP
70,051	P0DMV8;P0DMV9;A0A0G2JIW1;A8K5	P0DMV8;P0DMV9;A0A0G2JIW1;A8K	Heat shock 70 kDa protein 1	HSPA1A;HSPA1B;HEL-S-103
15,799	A0A0K0K1J1;P01034	A0A0K0K1J1;P01034	Cystatin-C	CST3

104,85	O43707;A0A0S2Z3G9;Q96BG6;F5GXS:	O43707;A0A0S2Z3G9;Q96BG6;F5GX:	Alpha-actinin-4	ACTN4
65,33	A0A0S2Z3Y1;Q08380;B4DVE1;B4DWA	A0A0S2Z3Y1;Q08380;B4DVE1;B4DW	Galectin-3-binding protein	LGALS3BP
57,838	B4DRR0;CON__P02538;A8K2I0;A0A0S	B4DRR0;CON__P02538;A8K2I0;A0A0	Keratin, type II cytoskeletal	KRT6A
29,032	A0A0S2Z4G4;A0A0S2Z4I4;B2RDE1;A0/	A0A0S2Z4G4;A0A0S2Z4I4;B2RDE1;A(	Tropomyosin alpha-3 chain;	TPM3;DKFZp686J1372;TPM
13,436	Q0ZCH9;A0A0X9USM3;Q0ZCH4;Q9UL!	Q0ZCH9;A0A0X9USM3;Q0ZCH4;Q9UI	Ig heavy chain V-III region JON	
13,525	A0A0X9UWK7;A0A0F7T891;A0A0F7TE	A0A0X9UWK7		
12,468	A0A0X9UWL5;A0A0X9TDD0;A0A125Q	A0A0X9UWL5;A0A0X9TDD0		
13,096	A0A125U0U7;A0A120HF66	A0A125U0U7		
60,673	A0A140VJI7;Q16610;C8CHS3;B7ZAS5	A0A140VJI7;Q16610;C8CHS3	Extracellular matrix protein	ECM1
49,496	A0A140VJJ6;P02679;C9JEU5;C9JC84;I	A0A140VJJ6;P02679;C9JEU5;C9JC84	Fibrinogen gamma chain	FGG;DKFZp779N0926
34,258	A0A140VK00;P25311;C9JEV0;A0JLQ0,	A0A140VK00;P25311;C9JEV0	Zinc-alpha-2-glycoprotein	AZGP1
69,284	A0A140VK27;P09960;B4DVZ8;Q59ES1	A0A140VK27;P09960;B4DVZ8	Leukotriene A-4 hydrolase;L	LTA4H
37,54	A0A140VK56;P37837;F2Z393;B4DID5	A0A140VK56;P37837;F2Z393;B4DID	Transaldolase	TALDO1
78,181	V9HWI4;A0A161I202;P02788;E7EQB2	V9HWI4;A0A161I202;P02788;E7EQB	Lactotransferrin;Lactoferric	HEL110;LTF
14,481	A0A193CHQ9;A0A0B4J1V0;Q0ZCJ6;A2	A0A193CHQ9;A0A0B4J1V0;Q0ZCJ6;A2N2G5		IGHV3-15;VH87-2
51,923	A0A1B0GUU9;P01871	A0A1B0GUU9;P01871	Ig mu chain C region	IGHM
44,552	P07339;V9HWI3;A0A1B0GWE8;A0A1I	P07339;V9HWI3;A0A1B0GWE8;A0A1	Cathepsin D;Cathepsin D lig	CTSD;HEL-S-130P
28,889	A0A1R3UCD2;P06870;A0A1L2BPJ2;A(	A0A1R3UCD2;P06870;A0A1L2BPJ2;A	Kallikrein-1	KLK1
15,257	P69905;A0A1S5UZ39;D1MGQ2;A0A1I	P69905;A0A1S5UZ39;D1MGQ2;A0A1	Hemoglobin subunit alpha	HBA1;HBA2
49,586	CON__P13646-1;A1A4E9;B4DL17;A8K	CON__P13646-1;A1A4E9;B4DL17;A8K2H9		KRT13
25,164	A2KBC4;A2KBC2;A2KBC3;A2KBC5;A2K	A2KBC4;A2KBC2;A2KBC3;A2KBC5;A2KBC6;A2KBC7;A2KBC8;A2KE		IGHV
10,392	A2MYE1;A2MYE2;P01599	A2MYE1;A2MYE2;P01599	Ig kappa chain V-I region Gal	A30
12,388	A2NB45;A0A125QYY8	A2NB45		
105,21	Q5T985;A2RTY6;D3DRR6;P19823;B4D	Q5T985;A2RTY6;D3DRR6;P19823;B4	Inter-alpha-trypsin inhibito	ITIH2
5,0526	A2VCK8;P62328;Q0P5T0;Q0P5U7;Q0I	A2VCK8;P62328;Q0P5T0;Q0P5U7;Q(	Thymosin beta-4;Hematopc	TMSB4X
122,2	A5PL27;P00450;E9PFZ2;B7Z5Q2;Q1L8	A5PL27;P00450;E9PFZ2;B7Z5Q2;Q1L	Ceruloplasmin	CP
223,02	A5YM51;P12883;Q59EV3;P13533;Q5(	A5YM51;P12883;Q59EV3;P13533;Q5	Myosin-7;Myosin-6	MYH7;MYH6
51,596	A8K008	A8K008		
99,949	A9X9L0;A8K2P8;B4DLJ5;Q02487;A9X!	A9X9L0;A8K2P8;B4DLJ5;Q02487;A9)	Desmocollin-2	DSC2;DKFZp686P18250;DI
161,1	A8K2U0;H0YGG5;Q6ZWK7;H0YH14;F5	A8K2U0;H0YGG5;Q6ZWK7	Alpha-2-macroglobulin-like	A2ML1
18,012	V9HWF5;A8K486;P62937;B2RE56;B4I	V9HWF5;A8K486;P62937;B2RE56;B4	Peptidyl-prolyl cis-trans isor	HEL-S-69p;PPIA
53,533	Q9UBG3;A8K5I6;B4DQ53	Q9UBG3;A8K5I6	Cornulin	CRNN
53,907	Q53GX6;A8K7Q1;Q02818;B4DZX0;H7	Q53GX6;A8K7Q1;Q02818;B4DZX0;H	Nucleobindin-1	NUCB1
59,256	P11413;A8K8D9;B4DYA7;Q2VF42;Q2(	P11413;A8K8D9;B4DYA7;Q2VF42;Q2	Glucose-6-phosphate 1-deh	G6PD

16,537	B2R4C5;P61626;F8VV32;A0A0B4J259	B2R4C5;P61626	Lysozyme;Lysozyme C	LYZ
11,367	B2R4R0;P62805;Q0VAS5;Q6B823	B2R4R0;P62805;Q0VAS5	Histone H4	HIST1H4H;HIST1H4A
46,659	V9HWJ2;B2R5M8;O75874;Q0QER2;V9	V9HWJ2;B2R5M8;O75874;Q0QER2;V	Isocitrate dehydrogenase [N	HEL-S-26;IDH1;HEL-216
30,656	B2R7T8;B1AK87;B1AK88;B4DWA6;P4	B2R7T8;B1AK87;B1AK88;B4DWA6;P4	F-actin-capping protein sub	CAPZB
27,566	V9HWG9;B2R983;P78417;Q5TA02;Q5	V9HWG9;B2R983;P78417;Q5TA02;Q	Glutathione S-transferase or	HEL-S-21;GSTO1
16,837	P0DP24;B4DJ51;H0Y7A7;P0DP25;B2F	P0DP24;B4DJ51;H0Y7A7;P0DP25;B2RDW0;P0DP23;Q9BRL5;E7E		CALM3;CALM2;CALM1
45,333	B3KNK9;Q8NBJ4;F8WAT9;C9JYM4;C9J	B3KNK9;Q8NBJ4	Golgi membrane protein 1	GOLPH2;GOLM1
50,151	P68363;B3KT06;B3KPS3;A8JZY9;Q9BC	P68363;B3KT06;B3KPS3;A8JZY9;Q9B	Tubulin alpha-1B chain;Tub	TUBA1B;TUBA1C
16,057	Q5H9A7;Q6FGX5;B3KQF4;P01033;H0Y	Q5H9A7;Q6FGX5;B3KQF4;P01033;H(	Metalloproteinase inhibitor	TIMP1
54,739	P55058;B3KUE5;Q53H91	P55058;B3KUE5;Q53H91	Phospholipid transfer prote	PLTP
143,66	B3KVV6	B3KVV6		
47,311	B3KXD3;P16870;H0YAM0;D6RF88;C9.	B3KXD3;P16870	Carboxypeptidase E	CPE
106,87	V9HWJ0;B4DJ30;Q14697;F5H6X6;B4I	V9HWJ0;B4DJ30;Q14697;F5H6X6;B4	Neutral alpha-glucosidase A	HEL-S-164nA;GANAB
56,852	B4DJI2;P28799;K7EKL3;K7EQ05;B4E1	B4DJI2;P28799;K7EKL3;K7EQ05;B4E	Granulins;Acrogranin;Parag	GRN
30,767	B4DL49;Q5HYG5	B4DL49		
50,663	Q6IAT1;B4DLV7;P50395;Q5SX87;Q8TI	Q6IAT1;B4DLV7;P50395;Q5SX87	Rab GDP dissociation inhibi	GDI2
46,496	B4DPP8;D3DNU8;B4E1C2;P01042;Q0	B4DPP8;D3DNU8;B4E1C2;P01042;Q(	Kininogen-1;Kininogen-1 he	KNG1
67,599	B4DPW9;P13797;Q53GY0;A0A0A0MS	B4DPW9;P13797;Q53GY0;A0A0A0M	Plastin-3	PLS3
35,789	B4DUH8;P23280;Q5FBW4;Q8N4G4	B4DUH8;P23280	Carbonic anhydrase 6	CA6
59,99	B4DVJ0;K7EIL4;K7ESF4;Q8N196	B4DVJ0	Glucose-6-phosphate isomerase	
74,831	B4E1B2	B4E1B2		
140,94	B4E1Z4;P00751;A0A1U9X7H2;A0A1U9	B4E1Z4;P00751;A0A1U9X7H2;A0A1U	Complement factor B;Comp	CFB
26,224	B5MDF5;P62826;J3KQE5;F5H018;H0Y	B5MDF5;P62826;J3KQE5;F5H018;H0	GTP-binding nuclear protein	RAN
71,553	B7Z507	B7Z507		
39,411	C9JV77;B7Z8Q2;P02765;B7Z556	C9JV77;B7Z8Q2;P02765;B7Z556	Alpha-2-HS-glycoprotein;Al	AHSG
78,831	B7Z992;A0A0A0MS51;B7Z9A0;B7Z6N2	B7Z992;A0A0A0MS51;B7Z9A0;B7Z6N2;A0A0A0MT01;B7Z2X4;B7		GSN
9,9355	B7ZLF8	B7ZLF8		
51,621	CON__P02533;P02533	CON__P02533;P02533	Keratin, type I cytoskeletal 1	KRT14
69,366	CON__P02768-1;P02768;F6KPG5;B2R	CON__P02768-1;P02768;F6KPG5;B2	Serum albumin	ALB
59,998	CON__P04259;B4DKV4	CON__P04259;B4DKV4		
51,267	CON__P08779;P08779;Q16195;K7EN	CON__P08779;P08779	Keratin, type I cytoskeletal 1	KRT16
59,51	CON__P13645;P13645;CON__Q148H6	CON__P13645;P13645	Keratin, type I cytoskeletal 1	KRT10
62,378	CON__P13647;P13647;F8W0C6;F8VV	CON__P13647;P13647	Keratin, type II cytoskeletal	KRT5
63,91	CON__P19013;P19013;B4DRW1;B4DI	CON__P19013;P19013;B4DRW1;B4D	Keratin, type II cytoskeletal	KRT4

65,432	CON__P35908v2;P35908	CON__P35908v2;P35908	Keratin, type II cytoskeletal	KRT2
27,745	D0PNI1;P63104;E7EX29;B0AZS6;B7Z2	D0PNI1;P63104;E7EX29;B0AZS6;B7Z	14-3-3 protein zeta/delta	YWHAZ
9,3804	Q5T123;Q86Z22;D3DPK5;Q9H299	Q5T123;Q86Z22;D3DPK5;Q9H299	SH3 domain-binding glutam	SH3BGRL3;HEL-S-297
51,672	D3DPU2;B2RDY9;Q01518;B4DNW7;B	D3DPU2;B2RDY9;Q01518;B4DNW7;I	Adenylyl cyclase-associated	CAP1
23,625	D6CHE9;U3KPS2;P24158	D6CHE9;U3KPS2;P24158	Myeloblastin	PRTN3
21,057	D9IAI1;P30086;A0A0K0K1J6;B4DRT4;	D9IAI1;P30086;A0A0K0K1J6;B4DRT4	Phosphatidylethanolamine	HEL-S-34;PEBP1
15,998	P68871;D9YZU5;Q14473;B3VL86;Q9E	P68871;D9YZU5;Q14473;B3VL86;Q9	Hemoglobin subunit beta;L	HBB
54,305	D9ZGG2;P04004;B7Z553;F5GX75;J7H	D9ZGG2;P04004	Vitronectin;Vitronectin V65	VTN
15,164	E7DVW5;Q01469;I6L8B7;A8MUU1	E7DVW5;Q01469;I6L8B7	Fatty acid-binding protein,	FABP5
69,412	P15311;E7EQR4;Q6NUR7;B2R6J2;V9H	P15311;E7EQR4;Q6NUR7;B2R6J2;V9	Ezrin;Tyrosine-protein kinas	EZR;HEL-S-105;EZR-ROS1
46,736	P01009;E9KL23;Q13747;Q9P173;Q3I	P01009;E9KL23;Q13747	Alpha-1-antitrypsin;Short p	SERPINA1
70,897	P11142;V9HW22;E9PKE3;Q53GZ6;Q9	P11142;V9HW22;E9PKE3;Q53GZ6;Q	Heat shock cognate 71 kDa	HSPA8;HEL-S-72p
103,28	P55786;E9PLK3;B7Z899;B7Z4B2;B3KU	P55786;E9PLK3;B7Z899;B7Z4B2;B3K	Puromycin-sensitive amino	NPEPPS
11,985	E9PNW4;Q6FHM9;E9PR17;P13987;H0	E9PNW4;Q6FHM9;E9PR17;P13987;H	CD59 glycoprotein	CD59
16,93	P60660;F8W1R7;G3V1V0;J3KND3;B7	P60660;F8W1R7;G3V1V0;J3KND3;B	Myosin light polypeptide 6	MYL6
21,058	F8WCF6;P59998;F8WDD7;A0A0A6YY	F8WCF6;P59998;F8WDD7;A0A0A6Y	Actin-related protein 2/3 co	ARPC4-TTLL3;ARPC4
8,4984	H0YLF3	H0YLF3		B2M
65,969	H6VRG0;H6VRF8;H6VRG1;P04264;H6	H6VRG0;H6VRF8;H6VRG1;P04264;H	Keratin, type II cytoskeletal	KRT1
11,737	H9ZYJ2;P10599	H9ZYJ2;P10599	Thioredoxin	TXN
19,591	J3KNB4;P49913	J3KNB4;P49913	Cathelicidin antimicrobial	CAMP
27,63	P54108;J3KPA1;I3L0A1	P54108;J3KPA1	Cysteine-rich secretory prot	CRISP3
84,659	K9JA46;P07900;Q2VPJ6;Q8TBA7;Q86	K9JA46;P07900;Q2VPJ6;Q8TBA7;Q8	Heat shock protein HSP 90-a	EL52;HSP90AA1
26,922	Q5SRT3;Q53FB0;O00299;A0A1U9X8Y	Q5SRT3;Q53FB0;O00299;A0A1U9X8Y	Chloride intracellular chann	CLIC1
82,577	O00391;A0A140VKE5;A8K4C2;A8K47	O00391;A0A140VKE5;A8K4C2;A8K47	Sulfhydryl oxidase 1;Sulfhyc	QSOX1;BPGF-1
34,333	Q53R19;O15144;Q9BXV5;A0A024R40	Q53R19;O15144;Q9BXV5	Actin-related protein 2/3 co	ARPC2
36,019	O60218;A4D1P0;C9JRZ8	O60218	Aldo-keto reductase family	AKR1B10
66,193	V9HWG7;O75083;Q53H17;Q53GN4;C	V9HWG7;O75083;Q53H17;Q53GN4;	WD repeat-containing prote	HEL-S-52;WDR1
36,688	P00338;V9HWB9;B4DJI1;F5GXY2;F5G	P00338;V9HWB9;B4DJI1;F5GXY2;F5	L-lactate dehydrogenase A c	LDHA;HEL-S-133P
32,118	V9HWH6;Q8N7G1;P00491;G3V5M2;C	V9HWH6;Q8N7G1;P00491;G3V5M2	Purine nucleoside phosphor	HEL-S-156an;PNP
44,614	V9HWF4;P00558;B4E1H9;B4DHB3;B4	V9HWF4;P00558;B4E1H9;B4DHB3;B	Phosphoglycerate kinase;Ph	HEL-S-68p;PGK1
45,205	P00738;H0Y300;J3QR68;A0A0C4DGL	P00738;H0Y300;J3QR68;A0A0C4DGI	Haptoglobin;Haptoglobin a	HP
163,29	P01023;H0YFH1;F8W7L3;Q9BQ22	P01023	Alpha-2-macroglobulin	A2M
187,15	V9HWA9;P01024;B4E216;M0QYC8;M	V9HWA9;P01024;B4E216	Complement C3;Compleme	HEL-S-62p;C3
16,214	P01036	P01036	Cystatin-S	CST4

16,387	P01037	P01037	Cystatin-SN	CST1
18,098	P01591;D6RD17;D6RHJ6;C9JA05	P01591;D6RD17;D6RHJ6	Immunoglobulin J chain	IGJ;JCHAIN
83,283	P01833	P01833	Polymeric immunoglobulin	PIGR
57,019	Q6N030;Q5EBM2;P01860;A0A286YES	Q6N030;Q5EBM2;P01860;A0A286YE	Ig gamma-3 chain C region	DKFZp686I15212;IGHG3;FI
94,972	P02671;A0A0S2Z3E8;Q3KRA7;Q6NSD8	P02671;A0A0S2Z3E8;Q3KRA7;Q6NSD	Fibrinogen alpha chain;Fibr	FGA
55,928	V9HVY1;P02675;B4E1D3;D3DP13;D6I	V9HVY1;P02675;B4E1D3;D3DP13;D6	Fibrinogen beta chain;Fibrir	HEL-S-78p;FGB
23,511	V9HWF6;P02763	V9HWF6;P02763	Alpha-1-acid glycoprotein;A	HEL-S-153w;ORM1
52,963	V9HWI6;P02774;D6RF35;D6RBJ7;D6F	V9HWI6;P02774;D6RF35;D6RBJ7	Vitamin D-binding protein	HEL-S-51;GC
51,676	P02790;Q9BS19;B7Z8Q4	P02790	Hemopexin	HPX
14,326	P03973	P03973	Antileukoproteinase	SLPI
59,755	P04040;B4DWK8;Q8TAK2;A0A1C9J7V	P04040;B4DWK8	Catalase	CAT
39,42	V9HWN7;P04075;H3BQN4;J3KPS3;H3	V9HWN7;P04075;H3BQN4;J3KPS3;H	Fructose-bisphosphate aldo	HEL-S-87p;ALDOA
11,139	Q76LA1;P04080;A0A1W2PS52;A0A1V	Q76LA1;P04080;A0A1W2PS52	Cystatin-B	CSTB
38,714	Q5TZZ9;P04083;B5BU38;Q5T3N1;B4D	Q5TZZ9;P04083;B5BU38;Q5T3N1	Annexin;Annexin A1	ANXA1
54,253	V9HWD8;P04217;B3KP79;M0R009;B3	V9HWD8;P04217;B3KP79;M0R009	Alpha-1B-glycoprotein	HEL-S-163pA;A1BG
36,053	V9HVZ4;P04406;E7EUT5;Q2TSD0;Q0Q	V9HVZ4;P04406;E7EUT5;Q2TSD0;Q0	Glyceraldehyde-3-phosphat	HEL-S-162eP;GAPDH
57,767	P04745;B7ZMD7;Q6NSB3;Q5T085;H7	P04745;B7ZMD7;Q6NSB3	Alpha-amylase 1;Alpha-amy	AMY1A
10,834	P05109	P05109	Protein S100-A8;Protein S1	S100A8
83,868	P05164;J3QSF7;P11678	P05164	Myeloperoxidase;Myeloper	MPO
15,603	Q53R15;P05976	Q53R15;P05976	Myosin light chain 1/3, skel	MYL1
13,242	P06702;B2R4M6	P06702;B2R4M6	Protein S100-A9;Protein S1	S100A9
97,147	P06737;E9PK47;B2R825;Q6P1L4;B4D	P06737;E9PK47;B2R825	Glycogen phosphorylase, liv	PYGL
10,044	P07108;B8ZWD1;A0A024RAF2;A0A0A	P07108;B8ZWD1;A0A024RAF2;A0A0	Acyl-CoA-binding protein	DBI
36,638	Q5U077;P07195;A8MW50;C9J7H8;F5	Q5U077;P07195;A8MW50;C9J7H8;F	L-lactate dehydrogenase;L-l	LDHB
68,478	P07476;B4DU44	P07476;B4DU44	Involucrin	IVL
15,054	P07737;K7EJ44;I3L3D5;CON__P0258	P07737;K7EJ44	Profilin-1	PFN1
28,518	P08246;B2MUD5	P08246;B2MUD5	Neutrophil elastase	ELANE;ELA2
28,837	P08311	P08311	Cathepsin G	CTSG
53,651	V9HWE1;P08670;B3KRK8;B0YJC4;Q53	V9HWE1;P08670;B3KRK8;B0YJC4;Q5	Vimentin	HEL113;VIM
23,356	V9HWE9;P09211;A8MX94;A0A087X2	V9HWE9;P09211;A8MX94	Glutathione S-transferase P	HEL-S-22;GSTP1
16,445	P09228	P09228	Cystatin-SA	CST2
192,78	P0C0L4	P0C0L4	Complement C4-A;Complen	C4A
48,462	P0DOX2	P0DOX2		
23,379	P0DOX7	P0DOX7		

22,83	P0DOX8;Q6PIK1	P0DOX8;Q6PIK1		IGL@
52,494	P10909;E7ERK6;H0YLK8;Q5ISQ2;E7ET	P10909;E7ERK6;H0YLK8;Q5ISQ2;E7E	Clusterin;Clusterin beta cha	CLU
72,332	V9HWB4;P11021;Q5IST7;B4DEF7	V9HWB4;P11021;Q5IST7	78 kDa glucose-regulated pr	HEL-S-89n;HSPA5
16,572	P12273	P12273	Prolactin-inducible protein	PIP
36,375	P12429;D6RA82;D6RFG5;D6RCA8;D6	P12429;D6RA82;D6RFG5	Annexin A3;Annexin	ANXA3
57,936	P14618;V9HWB8;B4DNK4;A0A024R5	P14618;V9HWB8;B4DNK4;A0A024R	Pyruvate kinase PKM;Pyruva	PKM;HEL-S-30;PKM2
28,82	Q6P6D7;Q6FHU2;Q53G35;P18669;Q0	Q6P6D7;Q6FHU2;Q53G35;P18669;Q	Phosphoglycerate mutase;P	PGAM1;hCG_2015269
108,33	P19021;B4DKE0;H7BYD9;D6RG20;Q1	P19021;B4DKE0;H7BYD9	Peptidyl-glycine alpha-amic	PAM
48,206	P20061	P20061	Transcobalamin-1	TCN1
46,872	P21128;B7Z3J4;B7Z7N4	P21128;B7Z3J4	Poly(U)-specific endoribonu	ENDOU
80,287	P22079;F5H386;B4E1M1;B4DUH9;J3	P22079;F5H386;B4E1M1	Lactoperoxidase	LPO
7,9053	P22532	P22532	Small proline-rich protein 2	SPRR2D
22,742	V9HWC6;P23284	V9HWC6;P23284	Peptidyl-prolyl cis-trans iso	HEL-S-39;PPIB
18,502	V9HWI5;P23528;G3V1A4;E9PP50;E9P	V9HWI5;P23528;G3V1A4;E9PP50;E9	Cofilin-1	HEL-S-15;CFL1
67,819	V9HWC0;P26038;Q6PJT4;B7Z4C7;B7Z	V9HWC0;P26038;Q6PJT4	Moesin	HEL70;MSN
16,891	P27482	P27482	Calmodulin-like protein 3	CALML3
16,08	P28325	P28325	Cystatin-D	CST5
15,693	P29373;Q5SYZ4	P29373;Q5SYZ4	Cellular retinoic acid-bindir	CRABP2
67,877	V9HWD9;P29401;Q53EM5;B4E022;B3	V9HWD9;P29401;Q53EM5;B4E022;B	Transketolase	HEL107;TKT
25,035	V9HWC7;P30041;A4UCS6;B4DUK1	V9HWC7;P30041	Peroxiredoxin-6	HEL-S-128m;PRDX6
17,031	V9HW35;P30044	V9HW35;P30044	Peroxiredoxin-5, mitochon	HEL-S-55;PRDX5
42,741	V9HWH1;P30740;B4E3A8;C9J7N5;B2	V9HWH1;P30740;B4E3A8	Leukocyte elastase inhibitor	HEL57;SERPINB1
57,509	Q6PKA6;P30838;E9PNN6;C9JMC5;I3L	Q6PKA6;P30838;E9PNN6;C9JMC5;I3	Aldehyde dehydrogenase, di	ALDH3A1
28,082	V9HWD6;P31946;B5BU24;Q4VY20;Q4	V9HWD6;P31946;B5BU24	14-3-3 protein beta/alpha;1	HEL-S-1;YWHAB
27,774	P31947;Q3YBA8	P31947	14-3-3 protein sigma	SFN
11,74	P31949;V9HWH9;B2R5H0	P31949;V9HWH9;B2R5H0	Protein S100-A11;Protein S	S100A11;HEL-S-43
21,892	V9HW12;P32119;B4DF70;A6NIW5;A0	V9HW12;P32119;B4DF70;A6NIW5	Peroxiredoxin-2	HEL-S-2a;PRDX2
62,064	P35527;CON__P35527;K7EQQ3	P35527;CON__P35527;K7EQQ3	Keratin, type I cytoskeletal 9	KRT9
61,448	P36871;B4DFP1;B7Z6C2;B4DDQ8;Q9	P36871;B4DFP1;B7Z6C2;B4DDQ8	Phosphoglucomutase-1	PGM1
22,391	P37802;X6RJP6;B7Z5A2	P37802;X6RJP6	Transgelin-2	TAGLN2
36,426	V9HWF2;P40925;B9A041;B8ZZ51;C9J	V9HWF2;P40925;B9A041;B8ZZ51;C9	Malate dehydrogenase;Mala	HEL-S-32;MDH1
53,139	P52209;B4E2U0;B4DL86;K7EM49;B4D	P52209;B4E2U0;B4DL86;K7EM49;B4	6-phosphogluconate dehyd	PGD
23,207	V9HWE8;P52565;J3KRE2;J3KTF8;J3Q	V9HWE8;P52565;J3KRE2;J3KTF8;J3C	Rho GDP-dissociation inhib	HEL-S-47e;ARHGDIA
10,245	Q6EZE9;P59666;P59665	Q6EZE9;P59666;P59665	Neutrophil defensin 3;HP 3-	DEFA3;DEFA1

26,669	V9HWK1;P60174;Q53HE2;B4DUI5;Q2	V9HWK1;P60174;Q53HE2;B4DUI5;Q	Triosephosphate isomerase	HEL-S-49;TPI1
44,76	P61160;Q8IY98;F5H6T1;B4DHK9;B4D	P61160;Q8IY98;F5H6T1;B4DHK9;B4I	Actin-related protein 2	ACTR2
29,174	V9HW98;P62258;G9K389;G9K388;Q5	V9HW98;P62258;G9K389;G9K388;C	14-3-3 protein epsilon	HEL2;YWHAE;YWHAE/FAM2
41,792	P63261;B4E3A4;B4DVQ0;I3L3I0;I3L1U	P63261;B4E3A4;B4DVQ0;I3L3I0;I3L1	Actin, cytoplasmic 2;Actin,	ACTG1
50,112	Q53G85;Q5VTE0;Q53GE9;Q6IPT9;Q6II	Q53G85;Q5VTE0;Q53GE9;Q6IPT9;Q6	Elongation factor 1-alpha;P	EEF1A1P5;EEF1A1;EEF1A1L
42,051	P68133;P68032;A8K3K1;B7Z6P1;P62	P68133;P68032;A8K3K1;B7Z6P1;P6	Actin, alpha skeletal muscle	ACTA1;ACTC1;ACTA2;ACTG
49,83	P68371;Q8IWP6;Q8IZ29;Q8N6N5;P04	P68371;Q8IWP6;Q8IZ29;Q8N6N5;P0	Tubulin beta-4B chain;Tubu	TUBB4B;TUBB2C;TUBB4A
22,693	P80723	P80723	Brain acid soluble protein 1	BASP1
113,75	Q02413	Q02413	Desmoglein-1	DSG1
22,11	Q06830;Q5IST6;A0A0A0MSI0;B2R4P2	Q06830;Q5IST6;A0A0A0MSI0;B2R4P	Peroxiredoxin-1	PRDX1
76,631	Q08188	Q08188	Protein-glutamine gamma-g	TGM3
15,945	Q14019;H3BT58	Q14019;H3BT58	Coactosin-like protein	COTL1
12,993	Q14508;A8K2M3	Q14508	WAP four-disulfide core dor	WFDC2
24,488	Q15907;H3BMH2;H3BSC1;A0A024R5	Q15907;H3BMH2;H3BSC1;A0A024R5	Ras-related protein Rab-11B	RAB11B;RAB11A
32,642	Q32Q12;Q6FHN3;J3KPD9;P22392;O6	Q32Q12;Q6FHN3;J3KPD9;P22392	Nucleoside diphosphate kin	NME1-NME2;NME2
53,113	Q86U78;Q53YY1;Q53GY3;Q59EP2;B4E	Q86U78;Q53YY1;Q53GY3;Q59EP2;B4	Angiotensinogen;Angiotens	AGT
51,098	Q6MZU6	Q6MZU6		DKFZp686C15213
37,926	Q6P5S2	Q6P5S2	Protein LEG1 homolog	LEG1
25,834	Q6PIL8;Q6PJF2	Q6PIL8;Q6PJF2		IGK@
60,54	Q6UWP8;K7ESC4	Q6UWP8	Suprabasin	SBSN
49,172	Q8N4F0;B4E209	Q8N4F0;B4E209	BPI fold-containing family B	BPIFB2
39,158	Q8TAX7;D6RHX1	Q8TAX7;D6RHX1	Mucin-7	MUC7
52,441	Q8TDL5	Q8TDL5	BPI fold-containing family B	BPIFB1
27,011	Q96DR5;A8K739	Q96DR5;A8K739	BPI fold-containing family A	BPIFA2
68,283	Q96G03;B4DN40;E7ENQ8;Q4W5D6;E	Q96G03;B4DN40	Phosphoglucomutase-2	PGM2
54,392	Q96HE7;G3V3E6;G3V5B3;G3V2H0;G3	Q96HE7	ERO1-like protein alpha	ERO1L
53,088	Q96K68	Q96K68		
596,33	Q9HC84;A7Y9J9;B4E1L4;O75372;Q93	Q9HC84;A7Y9J9	Mucin-5B	MUC5B;MUC5AC
44,786	Q9NPP6;A0A068LKQ8	Q9NPP6		
120,71	Q9NQ38;E7EWP9	Q9NQ38;E7EWP9	Serine protease inhibitor Ka	SPINK5
15,892	Q9NZT1;Q53H37	Q9NZT1;Q53H37	Calmodulin-like protein 5	CALML5
18,154	Q9UBC9;B1AN48	Q9UBC9;B1AN48	Small proline-rich protein 3	SPRR3
260,73	Q9UGM3;B6V682;B4E262	Q9UGM3	Deleted in malignant brain t	DMBT1
44,276	Q9UIV8;B7Z8T4;C9JL93;B7Z4X7;H7BZ	Q9UIV8;B7Z8T4	Serpin B13	SERPINB13

37,375	Q9UJ70;H7C3G9;C9JEV6;H7C286;H7(	Q9UJ70;H7C3G9;C9JEV6;H7C286	N-acetyl-D-glucosamine kin	NAGK
223,04	Q9UKX2;P13535;B4DRV3;B4E0I9;B4D	Q9UKX2;P13535	Myosin-2;Myosin-8	MYH2;MYH8
12,437	Q9UL90;A0A0J9YY99;A0A1C9J6Z4	Q9UL90;A0A0J9YY99		
21,627	Q9ULZ3;B2RAZ4;H3BP42	Q9ULZ3;B2RAZ4	Apoptosis-associated speck-	PYCARD
572,01	Q9Y6R7;A0A087WXI2;A0A087WUZ2;/	Q9Y6R7;A0A087WXI2	IgGFc-binding protein	FCGBP
24,823	S6BGD6	S6BGD6		

cluster
1
3
4
1
1
0
1
1
4
1
5
5
1
4
0
1
1
1
5
5
1
1
1
5
5
1
3
5
3
3
2
5
3

0
5
1
0
5
3
1
1
1
5
2
0
5
2
5
5
3
5
3
5
5
3
3
1
5
3
1
2
4
5
5
5
1
1

5
4
1
1
1
4
1
1
3
1
1
1
1
1
1
4
1
1
2
5
2
1
1
0
1
5
4
1
2
5
4
5
4
5

5
5
1
5
5
4
5
1
5
1
1
4
1
1
1
1
3
2
5
1
5
1
1
1
1
1
1
5
1
5
5
5
5
2

2
2
2
3
0
0
3
0
5
3
0
5
5
5
1
5
2
2
5
1
2
0
1
1
1
5
0
1
1
5
2
1
3
3

1
3
4
2
1
5
0
1
5
1
5
4
1
5
0
4
5
1
0
4
1
5
1
1
5
4
1
5
1
1
4
5
1
5

5
1
1
2
1
0
1
1
4
4
4
1
3
1
1
1
5
2
2
1
2
3
5
2
1
4
2
2
2
1
1
5
2
1

1
1
1
1
5
2
