# Supplementary material for: Salivary proteomic profile of young healthy subjects
Source: Front Mol Biosci. 2023 Nov 30;10:1327233. doi: 10.3389/fmolb.2023.1327233 (PMC10720708; doi:10.3389/fmolb.2023.1327233)
Supplement: Supplementary file 2 [file DataSheet1.PDF]

LFQ intensi	LFQ intensi	LFQ intensi	LFQ intensi	LFQ intensi	LFQ intensi	LFQ intensi	LFQ intensi	LFQ intensi
33,0193	33,0264	30,3544	30,3463	31,9541	31,9598	33,6724	33,7177	33,2035
33,7688	33,8073	33,7185	33,75	33,7575	33,8208	33,7615	33,771	33,9755
26,1431	26,0706	24,7158	24,7114	23,251	22,5798	27,0192	26,9795	29,1581
NaN	15,9436	21,6321	22,1505	NaN	NaN	17,4549	17,4925	17,7381
27,3857	27,346	27,8957	27,8715	26,9468	26,9997	26,8075	26,8035	27,3367
25,2595	25,2774	26,4676	26,5517	26,0329	25,9563	26,028	25,9896	25,733
NaN	NaN	22,5578	22,5439	NaN	NaN	19,6882	18,9781	20,7976
27,8316	27,8986	28,7702	28,799	28,4665	28,4737	28,3654	28,3972	28,2818
29,3412	29,2754	29,8731	29,8821	28,8626	28,8436	29,2424	29,2379	29,5997
29,3396	29,305	27,4761	27,5264	28,4641	28,445	28,2626	28,2919	29,8733
NaN	18,0987	20,5414	20,5875	NaN	NaN	NaN	NaN	NaN
35,778	35,7257	33,165	33,1787	36,4592	36,4553	35,644	35,6412	34,1033
32,983	32,9856	31,3507	31,3673	32,6555	32,6525	31,9841	32,0719	32,163
28,7706	28,7934	27,9579	27,9778	28,1259	28,167	29,6968	29,5607	29,7663
25,042	25,0697	26,2017	26,138	26,0864	26,0113	25,9721	25,8761	25,5071
28,1377	28,0681	27,3809	27,3832	26,1327	26,1288	27,3743	27,3165	26,4908
27,1622	27,1634	28,2471	28,2381	27,323	27,4146	26,7724	26,6804	27,1807
27,0975	27,078	26,2183	26,2329	26,4169	26,4677	26,9382	27,0181	27,0506
25,8883	25,7281	27,8422	27,8155	26,264	26,3544	26,4233	26,4113	26,7954
22,1936	20,99	24,2378	24,3852	23,2853	23,494	23,5572	23,3692	23,3914
27,1591	27,1527	27,5385	27,5442	26,9548	26,9763	27,3486	27,333	27,4651
25,4267	25,4124	26,5618	26,4836	25,1872	25,274	24,8837	24,8669	25,0716
28,7729	28,7728	26,6951	26,6802	28,6457	28,67	28,5287	28,5929	28,4032
25,7782	25,8147	27,1076	27,122	26,0623	26,2034	25,279	25,1745	25,9691
27,5988	27,6753	26,1796	26,1552	26,5707	26,5463	28,5978	28,6845	28,2703
25,8427	25,6995	26,3733	26,3254	25,1407	25,1709	25,6218	25,628	25,4921
25,9619	26,044	25,9169	25,8851	26,0707	26,2213	27,6093	27,6093	27,5725
21,298	NaN	24,509	24,3164	21,5612	23,3628	23,1897	23,0144	23,2931
26,1613	26,4258	25,7967	25,7115	25,7143	25,831	27,2324	27,2266	27,4987
24,7508	24,6545	26,9911	26,8923	25,9928	25,9795	25,7512	25,448	26,2662
26,6644	26,6772	27,8968	27,9157	25,6905	25,8748	25,4949	25,6081	25,6927
27,6646	27,4891	26,6192	26,8209	27,9171	27,7735	27,3591	27,3604	26,8822
27,0338	27,0967	28,8365	28,8069	26,8005	26,8069	26,3672	26,4411	26,2775
25,1512	24,9981	25,769	25,7573	25,2238	25,1407	26,0775	26,0638	24,4754
21,3107	21,3306	23,9974	23,9888	22,847	23,3065	23,3148	23,393	23,2843
26,9467	26,9312	27,7473	27,7342	26,8515	26,7981	26,6466	26,6521	26,5461
29,0249	29,0694	29,2163	29,1877	28,3526	28,4415	28,0886	28,086	28,0218
25,4321	25,4631	27,3801	27,3235	25,8134	25,7314	25,0483	25,0842	26,1563
25,6687	25,721	27,3926	27,4425	26,3367	26,2846	26,2516	26,1956	26,8336
27,2722	27,2643	28,024	27,9924	26,9116	26,8019	26,2555	26,2377	26,6542
27,7727	27,7195	27,4569	27,5137	27,3712	27,159	28,3839	28,4412	29,0787
26,4244	26,3579	26,8515	26,7977	26,0333	26,0626	26,2354	26,2473	25,5098
25,9343	25,9989	27,2906	27,263	25,5815	25,6092	26,7299	26,7688	26,0046
26,4098	26,482	27,3235	27,341	26,4381	26,579	25,3504	25,401	25,8722
24,7841	24,8396	25,7376	25,6981	25,0573	25,0264	24,5967	24,565	24,4464
26,856	26,7854	27,792	27,8781	26,7437	26,8564	26,5855	26,5871	26,6891
25,6466	25,7624	25,5792	25,5768	24,6556	24,7023	25,0302	24,9663	24,5298
23,2144	23,0972	24,9346	24,6949	23,2324	23,2478	23,529	23,3566	23,7489
29,9878	29,9163	28,6174	28,6373	31,2346	31,2671	30,3644	30,4097	30,7253
NaN	NaN	20,5266	21,0083	NaN	NaN	17,7273	18,5546	20,3146
24,6374	24,7109	24,3825	24,383	24,6908	24,7898	25,0443	25,0249	24,3986

25,8627	26,0328	27,3615	27,3043	26,4192	26,3609	25,8512	25,8784	26,2879
33,6522	33,704	32,0813	32,0285	33,1977	33,2052	32,6668	32,6671	32,6793
21,7409	23,2488	20,3261	20,6639	18,7234	NaN	18,5684	18,954	19,1367
25,7042	25,7225	26,6899	26,6315	25,4612	25,1535	24,7079	24,6159	25,1698
NaN	NaN	18,0872	18,0942	NaN	NaN	NaN	NaN	NaN
25,8682	26,034	26,4438	26,416	25,266	25,32	25,5095	25,465	24,9904
24,5223	24,5324	24,6839	24,6488	22,7426	22,7691	23,9183	23,6935	22,1058
27,1972	27,1575	27,6461	27,6039	26,7915	26,8127	26,4519	26,4354	26,2006
26,8011	26,8342	26,8927	26,8137	26,575	26,4251	27,5683	27,6007	27,3852
24,4023	24,3262	22,3383	22,3691	24,8239	24,6723	23,3403	23,2938	23,6941
21,7785	22,2063	24,2139	24,0206	23,14	22,9945	24,5057	24,5877	24,3342
24,9015	24,8411	24,0567	24,0829	23,4127	23,4614	23,8243	23,7605	23,1346
25,6626	25,8485	26,5131	26,6223	26,371	26,5901	27,9008	27,9431	27,4965
23,9232	23,8922	25,2555	25,3039	24,5054	24,7361	24,2151	24,387	24,0728
24,4181	24,3952	24,9396	25,0283	23,8128	23,6906	24,0714	23,9785	24,1925
23,7914	23,7662	25,0322	25,0388	23,7711	23,7491	24,7611	24,9593	24,9127
27,0986	27,1861	28,1456	28,2341	26,9037	26,7314	26,6393	26,6211	26,4877
20,9565	NaN	24,079	24,049	22,5021	22,5264	23,4485	23,6918	22,5452
29,6695	29,7329	31,445	31,4399	30,679	30,6629	30,0383	30,1342	30,1781
23,7383	23,9482	24,4813	24,5588	23,4114	23,4122	23,064	23,015	23,1056
22,4398	22,3173	23,7631	23,6342	22,8776	22,7881	22,8109	22,7286	22,6745
22,7607	23,1464	25,0066	24,7466	24,6189	24,7427	24,1558	24,149	24,0768
24,6092	25,015	25,428	25,4221	24,9506	24,9111	24,5311	24,6155	24,9801
29,3666	29,321	27,6091	27,5787	28,6702	28,6843	29,0259	29,0598	29,3308
25,243	25,2006	23,0905	23,0966	24,5784	24,6587	24,7559	24,7249	24,2201
27,8055	27,738	26,8862	27,0083	27,2947	27,3052	27,1547	27,1948	24,8529
23,6076	23,8012	25,0703	25,1037	24,3335	24,3637	24,4146	24,2888	23,5831
24,4821	24,5209	25,4179	25,4143	24,3175	24,3791	23,9194	23,8489	24,121
27,5523	27,5469	28,276	28,2851	27,0961	27,0574	26,5097	26,5098	26,7255
25,5869	25,4595	26,6193	26,6433	25,853	25,8258	25,597	25,6696	26,1595
27,0359	27,0917	25,9223	25,7892	26,8811	26,8697	26,4177	26,3929	26,4984
17,2637	16,5203	17,4151	18,2029	NaN	16,2192	16,8802	15,8936	17,1065
NaN	21,5806	23,8687	23,8737	23,2285	23,358	22,978	22,8511	24,0695
25,8877	25,8115	27,5128	27,5984	26,2256	26,3193	26,5532	26,4807	27,0887
24,2611	24,7858	22,9162	22,5211	24,0096	24,0734	24,7622	24,8206	24,6865
NaN	NaN	21,1608	21,4764	NaN	21,1732	22,5683	22,5365	22,108
27,4156	27,3934	29,439	29,3552	28,2038	28,1117	27,4143	27,3494	27,8981
20,9626	20,293	26,557	26,5367	24,9222	23,9975	24,9122	25,0825	24,5615
27,1286	26,926	25,8987	25,945	28,063	28,1244	29,2437	29,2621	28,02
21,4877	23,0223	22,7174	23,1861	20,2007	19,9232	22,9544	22,7783	23,9126
29,0175	28,9645	30,3338	30,3119	29,0069	29,0195	28,6464	28,6804	29,4294
NaN	NaN	22,3634	22,2356	NaN	NaN	NaN	NaN	20,4175
25,919	25,9491	24,7095	24,5032	26,0469	26,1419	25,5782	25,5263	25,6763
22,2324	22,6624	24,1684	24,0679	23,5426	23,1008	23,0753	22,9573	22,8058
26,3553	26,0919	24,7532	24,992	26,8427	26,8311	27,3288	27,2194	26,8853
26,3103	26,2757	26,7657	26,7647	25,4767	25,3312	26,068	26,0112	25,491
27,514	27,571	28,1954	28,1869	26,2592	26,183	27,2541	27,2445	28,0683
26,2881	26,1814	27,167	27,2135	25,9325	26,0423	26,3512	26,3431	26,8522
27,818	27,9253	28,2581	28,2291	27,316	27,2363	27,1764	27,1629	26,8635
32,2244	32,1293	30,6875	30,7137	31,5589	31,5643	31,305	31,2726	31,104
31,2739	31,2045	27,4038	27,3985	32,3327	32,3585	33,8288	33,9234	33,457
26,8593	26,8797	27,5977	27,6308	26,5291	26,5042	27,3988	27,3585	25,9114

23,7467	23,9885	24,292	24,2304	23,1975	23,2062	22,6892	22,6925	22,8059
NaN	19,1128	24,4413	24,3061	20,8947	22,0061	NaN	NaN	23,132
24,0315	24,0123	25,6175	25,3429	20,7709	20,1831	22,6081	22,2381	22,7539
23,0621	22,4699	24,671	24,599	23,879	23,6409	24,1999	24,0794	23,585
24,8798	24,849	24,7915	24,7922	25,06	25,0215	25,4548	25,4023	24,726
24,8291	24,9152	26,2138	26,1171	25,8509	25,8241	25,264	25,273	25,4262
22,9891	23,1038	25,1678	25,0676	24,0691	24,0976	23,7628	23,6766	23,938
26,2518	26,1776	24,143	24,0817	25,939	26,0694	26,1676	26,2273	26,2015
26,368	26,4139	27,2177	27,2936	26,009	26,0334	25,5677	25,5498	25,5837
27,2474	27,2646	27,2016	27,1413	27,6402	27,5632	26,5691	26,5699	28,1377
NaN	NaN	NaN	NaN	NaN	NaN	NaN	NaN	NaN
21,284	20,0815	23,1074	23,0779	23,5279	23,4643	24,8273	24,7901	25,092
27,0335	26,9787	27,5362	27,5089	26,2513	26,274	26,2529	26,1908	26,1255
25,2157	25,2203	26,078	25,9935	23,9703	24,0833	25,2348	25,2783	24,7989
23,775	23,9594	25,0907	25,0003	24,6935	24,7373	24,0481	24,1342	23,8984
27,9613	27,9471	25,3461	25,3305	27,0734	27,0957	27,0992	27,0482	27,0393
21,725	21,7692	23,4895	23,7703	21,6173	21,0147	21,8784	22,7565	22,0981
22,5185	22,5178	23,4478	23,5526	22,4096	22,8468	23,1903	23,1876	21,2296
NaN	20,3516	24,1648	24,1544	22,8778	22,9874	23,1026	23,1957	23,2961
22,67	22,5711	24,0189	23,9187	21,3052	22,6975	22,7468	22,9338	22,7183
22,2574	22,379	23,6185	23,4831	20,8285	22,3231	23,013	23,1508	22,5392
25,0261	25,0623	24,214	24,1776	25,117	25,1625	24,6074	24,4154	24,6272
NaN	NaN	19,6596	20,4183	NaN	NaN	NaN	NaN	NaN
26,151	26,1266	28,4305	28,4292	26,7439	26,7242	27,1283	26,9958	26,7676
23,6917	23,7728	25,3218	25,2932	21,8708	22,2923	22,6095	22,0714	22,8993
24,8155	24,7189	24,4686	24,4041	24,2005	24,2841	24,6009	24,7996	23,8601
25,9657	25,9484	27,6218	27,4994	26,6476	26,6768	26,1731	26,1912	26,5997
NaN	NaN	20,8487	19,843	NaN	NaN	NaN	NaN	NaN
23,6141	23,7896	23,1789	23,1719	21,2303	22,1294	22,2177	22,116	21,0307
22,8007	22,8114	25,7661	25,8245	23,7862	23,5826	24,3989	24,5443	24,8392
23,3809	23,3117	24,1044	24,0622	23,9116	23,9578	23,7635	23,736	23,5831
30,7829	30,7968	29,1531	29,2277	30,502	30,5094	29,9745	30,0522	30,0283
24,4043	24,2822	25,7851	25,7476	24,6854	24,6664	25,0504	24,8759	25,6486
25,3589	25,2677	26,5688	26,4706	25,6279	25,4248	25,3892	25,2429	24,9773
24,7952	24,625	25,1958	25,2374	24,3492	24,4631	24,8351	25,0304	23,8264
23,1544	22,5637	23,3746	23,514	23,5931	22,6234	23,3781	23,5147	22,8993
25,0971	25,089	24,7005	24,5954	24,2617	24,2516	24,7168	24,669	23,8844
29,8212	29,8004	28,8555	28,9116	29,8102	29,9331	30,4708	30,5497	30,4429
29,2225	29,2697	27,21	27,297	28,384	28,3479	30,101	30,1439	30,2582
NaN	NaN	21,254	21,7512	NaN	NaN	22,6235	22,8025	NaN
23,9976	23,9644	21,782	22,1889	23,2027	23,3485	25,5365	25,5088	25,6344
27,6867	27,7618	27,1716	27,1538	26,9549	26,981	30,2261	30,1208	25,415
25,6154	25,5401	22,4668	22,5535	24,2459	24,2549	24,2042	24,127	25,0992
22,9765	22,9677	24,6449	24,7255	23,6839	23,7326	23,1449	23,2339	23,2701
24,1471	24,0923	23,6576	23,7	24,3977	24,4087	23,0354	23,1632	23,8855
30,5008	30,4874	25,6328	25,3561	32,1168	32,1747	32,4693	32,3001	32,3866
23,5904	23,5012	23,877	23,8964	23,4383	23,4821	24,3318	24,459	24,5117
23,7207	23,7733	18,8618	NaN	20,6606	20,553	20,7724	20,6373	NaN
23,3609	23,4178	24,8552	25,069	21,8711	23,4251	23,6768	23,5591	23,4578
22,9032	23,2636	24,1908	24,0266	NaN	21,9959	22,393	22,6551	21,6764
25,6828	25,7007	23,8068	23,8048	23,7464	23,6447	24,6078	24,4669	22,3838
22,1265	21,394	24,1279	23,9875	23,0763	22,6198	22,7069	22,788	21,5987

21,8663	22,0686	23,6099	23,5311	21,6432	21,8592	21,9983	22,1198	21,2123
27,6431	27,4777	28,43	28,4017	28,3192	28,4885	28,8576	28,8225	29,2367
20,6292	NaN	21,1964	21,2513	20,6217	20,9592	20,6293	19,9635	20,3817
23,3225	23,1846	21,7868	21,8456	22,7202	22,9017	22,9225	23,1469	22,9392
22,514	22,5075	22,8603	22,6653	22,3275	22,2727	22,229	22,7638	22,3032
NaN	NaN	22,6706	22,5444	21,4272	21,295	21,1682	21,0115	21,5701
26,6938	26,6971	26,6757	26,5983	28,2919	28,3391	28,2055	28,2545	30,7101
26,6411	26,7698	27,1492	27,1602	26,6266	26,8945	27,1747	27,0963	26,2444
23,2674	23,3515	25,2105	25,3528	24,3479	24,4065	23,953	24,4966	24,8429
32,1232	32,1052	30,876	30,8513	32,5484	32,5843	33,4853	33,5516	33,4724
23,215	22,8561	20,9542	20,6004	22,7414	22,7344	23,4435	23,4457	23,5317
24,3344	24,1774	23,1108	23,2267	23,7497	23,7065	23,4907	23,8521	22,2967
29,0172	29,0361	25,6703	25,7206	30,0532	30,0599	30,9232	30,9546	30,9492
24,9372	24,8696	24,4543	24,4841	23,5881	23,9127	23,9277	23,8525	23,6991
24,0542	24,5943	21,7146	21,9421	23,4524	23,875	23,6651	23,7564	23,337
30,9789	31,0987	29,1686	29,2746	29,9666	29,9631	29,7567	29,7826	29,6946
23,2587	23,5867	24,4731	24,6284	23,9518	23,7892	23,4614	23,5491	23,8717
21,7872	21,359	23,6628	23,7407	22,952	22,8325	22,6141	22,602	22,3813
NaN	NaN	20,1003	20,4693	NaN	NaN	NaN	NaN	20,9245
NaN	NaN	19,4187	19,3431	NaN	NaN	NaN	NaN	NaN
21,7798	22,1239	23,6905	23,5484	NaN	20,181	NaN	NaN	NaN
21,7311	21,8048	23,3961	23,3541	21,675	21,1292	22,3263	22,1647	20,7139
22,5475	22,7098	24,367	24,2592	23,3992	23,561	23,0138	23,188	23,2918
20,431	21,1548	23,8533	23,9238	19,1624	21,0409	NaN	NaN	21,074
24,3275	24,0521	20,0297	20,3547	22,7922	23,0235	24,8059	24,8253	24,5726
24,0242	24,3848	23,7442	24,3535	24,8652	24,7631	24,1693	24,0668	24,754
22,0979	22,3378	24,3246	24,3738	23,098	23,297	23,8245	23,4769	23,4948
28,4737	28,3692	26,4235	26,5819	30,4055	30,5789	30,1482	30,1012	29,1452
23,5091	23,6952	24,7134	24,7477	24,408	24,3672	24,1625	24,0634	23,8541
22,4024	21,8534	23,9233	24,014	22,4123	22,2571	22,6037	23,0522	23,1294
25,9354	26,0672	30,6379	30,7054	29,6548	29,6871	27,5131	27,5575	29,7481
24,4511	24,6116	24,9732	25,0385	23,9901	23,7759	23,6146	23,5077	23,5352
26,835	26,6939	28,9083	28,9373	27,4623	27,5192	27,0757	27,1451	27,4607
27,2949	27,2071	28,2189	28,1502	27,1786	27,1613	27,4532	27,295	26,742
25,5054	25,6646	27,727	27,6353	26,885	26,9167	26,013	26,0639	26,6079
22,616	22,8945	24,2738	24,4124	23,7671	23,4205	23,056	22,8368	23,0605
21,7808	22,3665	24,4251	24,419	21,6694	NaN	21,8281	22,0161	21,6497
21,4342	22,4823	23,5473	23,4811	22,6584	22,0759	22,6023	22,8237	22,6034
22,0001	21,7642	24,1799	24,2748	22,3088	22,5011	22,5321	22,7928	22,5573
21,6668	22,1135	23,0825	23,1898	22,26	22,694	22,9231	22,7461	23,2266
22,1183	21,7964	25,4276	25,626	23,3329	23,1688	23,4775	23,2894	23,7218
23,2349	23,2143	27,6153	27,6294	26,0403	25,9611	20,8004	NaN	21,8061
25,8825	25,8896	25,3656	25,319	24,7435	24,9438	24,5928	24,704	24,4303
27,0213	27,1415	26,2407	26,2805	27,9299	27,8634	27,8608	27,9917	28,1174
22,7473	22,6734	24,9227	25,0574	22,2307	22,241	23,8359	23,715	20,5647
NaN	NaN	19,722	NaN	NaN	NaN	NaN	NaN	NaN
23,923	23,4004	24,9456	24,9786	23,2947	23,2905	23,4902	23,7274	24,0685
22,4554	22,1517	24,6757	24,7448	22,3858	23,0772	22,2054	22,3046	23,0618
NaN	NaN	19,684	19,8302	NaN	NaN	21,6793	20,7159	21,1642
25,8039	25,7616	24,0576	23,9521	26,1757	26,2029	25,4082	25,3356	25,7096
23,4118	23,4972	24,8332	24,7207	23,0652	23,1288	23,2432	23,2684	23,0518
20,7021	NaN	22,5986	22,6748	21,6838	20,8942	21,9334	22,3644	21,1133

23,8821	23,9707	23,3661	23,3582	22,3211	22,5735	22,49	22,3708	22,3739
21,3749	21,6242	24,2534	24,3219	22,5952	22,4453	NaN	21,7099	21,9651
23,9819	23,9714	22,2809	22,5229	21,8368	21,471	22,8383	23,0094	21,8238
21,8818	21,3089	23,8192	23,8156	22,7026	22,5732	22,1642	21,9355	22,6594
NaN	NaN	NaN	NaN	22,0939	22,4789	22,8849	23,1671	22,057
21,5314	21,9249	23,1864	23,1133	21,5477	20,8754	22,6796	22,4081	NaN
NaN	NaN	NaN	20,319	NaN	NaN	22,5367	22,9686	20,7507
NaN	NaN	21,6032	21,5067	20,9051	NaN	20,5316	21,1533	20,4935
31,2253	31,1877	29,6322	29,6502	31,2003	31,2864	33,0265	32,9253	33,0563
25,7527	25,7928	24,3482	24,4214	26,9273	26,9328	26,8767	26,8258	27,9014
NaN	NaN	23,1826	22,9417	21,9874	21,6652	21,8465	22,2916	22,0507
22,5995	22,7499	23,0022	22,9431	21,7158	21,0579	21,3015	21,3037	21,5102
26,4889	26,5075	27,9479	27,9664	26,5396	26,4851	26,5789	26,5545	26,2773
26,5411	26,5403	24,8525	24,8549	24,5012	24,7656	27,3558	27,353	26,1825
21,5643	21,4684	22,4548	22,6519	21,4287	21,0869	NaN	20,8328	21,1549
21,3104	NaN	23,2977	23,4611	NaN	NaN	22,1107	22,3378	22,0528
24,2918	24,4171	24,0595	24,0619	24,3097	24,4072	24,3945	24,3367	23,9572
23,0014	23,0087	23,878	23,9371	23,5101	23,0776	22,925	22,8378	22,7588
21,0664	NaN	21,7214	21,7441	NaN	NaN	NaN	NaN	NaN
21,1462	21,7276	NaN	18,1832	NaN	NaN	23,5322	23,4819	NaN
22,7535	22,7274	24,2184	24,3377	NaN	23,6716	24,5884	23,4663	23,6443
26,5338	26,6391	30,8285	30,8761	29,9246	30,0274	27,5659	27,6434	30,258
23,203	23,2198	23,4356	23,4175	24,6529	24,688	24,4116	24,4923	24,0033
25,5195	25,4775	25,0615	25,1225	24,8539	24,7079	25,1633	25,1561	24,1314
24,2055	24,0341	24,712	24,756	24,3745	24,4209	24,3452	24,2954	24,4088
16,5634	NaN	26,4187	26,599	17,9575	18,0695	NaN	16,8449	17,3534
21,5478	21,1849	21,6299	22,0802	NaN	NaN	NaN	NaN	NaN
25,2449	25,1949	25,4029	25,338	24,2423	24,0172	24,0648	24,263	23,918
22,7418	22,8266	24,8866	25,04	23,4598	23,4823	24,3844	24,5466	23,5285
22,1654	22,1837	23,966	23,7978	21,4522	21,8952	22,0169	22,1471	22,4189
24,6793	24,7476	24,9564	25,0262	23,6474	23,641	23,7024	23,5331	22,8111
21,9613	23,0595	23,9178	23,9238	22,5783	22,5281	22,5529	22,5233	22,325
21,6216	21,8709	21,5695	21,6261	21,8942	21,7984	21,8289	22,3523	22,3847
27,4679	27,5639	26,2412	26,1185	26,3022	26,2153	26,2367	26,1224	27,5349
20,3152	21,0489	25,6501	25,7651	NaN	NaN	22,2827	21,9695	20,9147
22,9757	22,7631	24,0871	24,2105	23,4676	23,113	22,699	22,593	23,0629
NaN	21,3673	23,5518	23,4477	21,7547	21,8977	22,3317	22,1844	22,604
23,4356	23,0575	21,9516	21,8911	23,6293	23,7964	23,7038	23,6872	23,2741
22,8728	22,8777	24,0869	24,0763	22,3929	22,5069	21,9944	22,0609	21,8411
22,4893	22,9857	24,1021	24,0854	23,0748	23,1141	NaN	NaN	23,0557
NaN	NaN	21,5686	21,184	NaN	20,8933	20,5195	20,9233	20,5499
21,7375	21,9192	22,2149	22,2048	22,3424	22,4626	22,5335	22,5823	22,1289
24,0519	23,9275	22,7731	22,7147	24,0115	23,7374	23,2555	23,0338	23,1651
21,6595	21,776	22,8371	22,9221	21,7744	22,1358	22,312	22,1806	22,6426
NaN	NaN	NaN	NaN	NaN	NaN	18,6136	19,3276	NaN
27,6541	27,6566	28,3601	28,2837	26,838	26,8555	27,1138	27,2414	26,5497
NaN	NaN	22,3556	22,3598	NaN	NaN	NaN	21,1521	20,4848
NaN	NaN	21,0368	20,9043	21,3386	21,5596	22,3696	22,5224	22,3322
30,2286	30,1945	23,4201	22,364	30,1955	30,2269	31,958	31,9377	31,097
28,0324	28,0319	26,5251	26,4867	27,3691	27,3474	27,8421	27,9196	28,0945
22,2949	22,297	22,9349	22,9112	21,7295	NaN	22,1093	22,2728	20,3252
23,7928	23,6375	23,5218	23,5728	22,2387	21,5211	23,438	23,4623	22,3383

21,5748	21,6649	23,7104	23,5633	23,1452	23,374	21,9091	22,475	23,0459
21,7011	21,7366	22,2793	22,2959	NaN	20,8737	21,0529	20,8411	20,6867
NaN	NaN	NaN	21,4949	21,7898	22,8615	23,3414	23,5198	23,5055
23,077	23,0945	25,4795	25,7358	22,1031	21,9253	23,1288	23,3224	22,9187
31,7061	31,7023	29,1032	29,0444	30,1701	30,2278	30,3069	30,4055	29,4187
21,5897	21,6977	23,3554	23,3105	NaN	21,7772	NaN	21,6089	21,9789
NaN	NaN	20,5289	20,293	NaN	NaN	NaN	NaN	NaN
22,4294	22,4257	23,3452	23,1801	22,7113	22,576	22,7368	23,002	22,0374
21,0254	NaN	23,2826	22,8237	NaN	NaN	NaN	20,694	21,5602
22,0265	21,9922	NaN	NaN	22,652	23,0946	22,3335	21,4445	22,1718
NaN	NaN	22,0613	22,5301	NaN	NaN	22,5296	21,5066	22,1505
22,5811	22,6338	22,9992	23,1888	22,2461	22,2425	22,2905	21,9653	21,6667
NaN	NaN	22,0598	22,2948	20,9389	21,015	NaN	NaN	NaN
NaN	21,2181	21,1284	20,798	21,2379	21,2432	20,7431	20,7846	21,1985
26,7769	26,7007	26,084	26,0526	26,2926	26,3689	29,6112	29,5486	24,4596
26,0251	25,9728	28,1078	28,2286	26,8321	26,9703	26,6081	26,6366	27,0154
NaN	NaN	26,7054	27,1191	NaN	NaN	23,1809	23,6126	23,6127
25,0063	24,888	23,9618	23,9136	25,0371	25,1112	26,0621	26,0118	26,1461
23,4221	23,1018	21,0175	21,3293	23,5401	23,6383	22,0486	21,9814	23,0491
22,8227	22,9612	22,7989	22,6802	23,9075	23,7475	22,5445	22,4832	22,1393
NaN	NaN	NaN	NaN	NaN	NaN	NaN	21,7187	NaN
NaN	NaN	21,2565	21,608	NaN	NaN	NaN	NaN	21,9482
22,3859	23,456	24,84	24,9127	23,9316	24,3221	25,4765	25,4762	26,0644
NaN	NaN	21,4494	21,2851	20,9061	21,1447	21,5093	21,6129	21,0812
23,4415	23,4964	24,5381	24,6463	NaN	21,2741	22,9626	23,4293	23,1989
21,1721	21,0928	22,0912	22,1704	23,1873	23,2616	22,3537	22,1288	24,1091
24,7409	24,7818	27,8262	27,8003	23,2784	23,5228	24,5134	24,4182	23,2411
22,4266	23,416	22,4102	22,5499	22,2829	21,4714	23,383	23,521	21,5434
23,9652	23,9995	24,1418	24,2049	23,7087	23,969	23,97	23,8777	23,3233
23,0483	NaN	23,1303	22,9864	22,3128	NaN	22,3269	22,1758	21,4735
23,4135	23,2663	25,0406	25,1533	23,9947	23,678	23,2297	22,9784	23,7084
NaN	NaN	21,5345	21,3217	NaN	NaN	NaN	NaN	NaN
24,1497	23,641	22,5239	22,2162	23,7362	23,3969	22,6284	23,1614	23,6172
21,7393	21,6016	21,7434	21,4154	21,6931	NaN	21,5792	21,1842	21,4942
22,4292	22,1413	23,3202	23,5316	22,7263	22,768	22,3592	22,3996	22,098
22,769	22,7677	23,7316	23,6178	NaN	21,0536	20,8851	21,2082	20,5806
22,0411	20,5059	22,6116	22,7289	21,9242	21,9472	22,0792	22,1668	22,0401
23,3445	23,3178	21,6208	21,3587	NaN	NaN	22,6	21,914	NaN
21,0233	20,9332	21,8922	22,1269	20,964	20,9053	20,8741	NaN	NaN
22,5504	22,3761	24,2024	24,2253	23,3248	23,3264	22,9498	22,9086	22,795
NaN	20,7056	20,8784	20,6692	NaN	NaN	NaN	20,2111	20,564
20,413	20,9097	24,5345	24,5668	NaN	NaN	19,9253	19,8475	NaN
23,348	23,0793	NaN	NaN	23,1873	22,9027	23,07	23,124	23,9203
NaN	NaN	21,8399	21,89	20,4613	21,1339	20,8073	20,7319	20,5623
NaN	NaN	20,6655	20,6706	21,254	20,8682	NaN	NaN	21,0309
21,2096	NaN	22,906	22,8815	22,7431	22,5563	21,7704	21,9634	22,4545
NaN	NaN	23,6042	23,6777	NaN	NaN	NaN	20,755	NaN
22,8606	23,0324	21,6019	21,4316	22,2135	22,1061	23,5034	23,5676	24,2267
21,2541	20,9106	22,6313	22,6016	21,8926	21,7334	21,3054	21,638	21,3695
22,5075	22,676	23,7933	23,7275	22,2809	22,4842	21,9865	21,418	22,3262
24,8956	25,013	25,379	25,2957	22,1917	22,1456	22,095	21,9601	22,3334
21,6567	NaN	22,2385	22,2322	21,937	21,7674	22,1502	22,1307	21,8413

NaN	NaN	22,3725	21,5057	NaN	NaN	NaN	NaN	NaN
26,3758	26,3574	26,5656	26,547	25,6366	25,6052	25,1243	24,9913	25,2317
NaN	NaN	20,302	20,3375	NaN	NaN	NaN	NaN	NaN
NaN	NaN	NaN	NaN	NaN	NaN	20,3258	20,2343	21,6821
NaN	NaN	23,5011	23,273	NaN	NaN	21,1014	21,0986	21,1141
21,9029	21,8563	22,9794	23,0122	22,9102	22,7055	22,6671	22,8296	22,3357
23,4581	23,3412	24,3523	24,295	24,0336	23,9332	24,4246	24,4244	23,9959
21,8204	NaN	23,4822	23,4499	22,4672	22,7137	22,5241	22,2518	22,8015
21,4044	NaN	22,3272	22,4008	20,8014	20,975	21,0545	21,1158	21,135
25,6538	25,6125	26,0674	26,1326	25,8454	25,9349	25,2882	25,2838	26,5543
23,3748	23,3838	21,7354	22,2949	25,6061	25,7799	25,811	26,0572	27,0559
25,4633	25,3072	25,6024	25,5479	22,538	22,7979	23,2995	23,1374	23,6123
22,8347	22,5519	24,1084	24,2827	23,5194	23,3181	25,3155	25,2393	25,6453
NaN	NaN	NaN	NaN	NaN	NaN	NaN	NaN	NaN
21,8462	21,4662	22,8231	22,9226	NaN	21,4566	22,78	22,5668	20,8664
26,6786	26,6547	24,7615	24,7512	27,7312	27,6223	29,4106	29,381	29,4088
23,5638	23,6246	24,7649	24,6732	24,1741	24,3144	24,0523	24,1242	23,881
21,8003	21,1695	21,7478	21,7673	21,5606	21,3492	21,3914	21,0571	21,5098
NaN	19,0129	23,2275	23,4216	19,4108	NaN	23,0664	22,7729	23,7473
NaN	NaN	NaN	NaN	NaN	NaN	20,977	20,8541	NaN
24,0028	23,7961	24,9253	25,0907	22,9484	23,8302	23,5499	23,4056	23,5689
NaN	NaN	NaN	NaN	NaN	NaN	21,6136	21,5394	21,4233
22,9245	23,1767	21,777	20,0946	22,9083	23,1261	21,4112	21,6248	22,7056
NaN	NaN	21,4059	21,3377	NaN	20,9877	21,114	21,2377	21,036
20,9091	NaN	22,6868	22,2062	20,1738	NaN	NaN	NaN	NaN
23,3992	23,5826	23,3437	23,2585	23,7423	23,3577	22,3354	22,2738	23,022
22,6783	21,9114	23,9084	23,8713	22,4739	22,1949	21,9988	22,3594	22,3337
NaN	NaN	21,736	21,8526	NaN	NaN	20,8726	NaN	21,1497
20,5055	20,4338	20,9093	20,9573	NaN	NaN	20,5253	20,3897	20,4706
22,9756	23,0217	24,8003	24,7911	23,9565	23,8182	23,9662	23,8914	23,7738
NaN	NaN	21,7416	21,8648	NaN	20,5764	20,705	NaN	20,9368
26,7636	26,7617	26,4137	26,3253	25,5185	25,4992	25,9055	25,9592	24,9571
20,5545	21,1749	21,0878	20,9586	21,3336	21,2075	20,6867	NaN	NaN
21,4589	21,3164	22,6598	22,5217	21,5368	21,6749	NaN	21,4347	21,3887
NaN	NaN	21,2786	21,4268	NaN	NaN	21,8249	21,696	21,5562
22,8834	22,8947	22,5502	22,4905	23,1857	23,4575	23,8657	23,803	24,2285
21,3655	NaN	22,8021	22,4101	NaN	NaN	21,1346	21,4128	21,1372
20,4717	NaN	22,4729	22,7369	21,1816	NaN	20,2476	NaN	20,8738
23,6548	24,1378	22,4527	22,4926	25,8679	25,4825	24,6269	24,6317	24,6549
NaN	NaN	21,7049	21,7545	NaN	NaN	NaN	NaN	NaN
NaN	NaN	NaN	20,4087	NaN	NaN	NaN	NaN	NaN
22,682	22,7284	24,0687	24,0687	22,7422	22,6734	22,5916	22,6218	22,704
23,0637	23,6427	24,1744	24,2651	23,7491	23,9317	23,7331	23,7836	24,1006
NaN	NaN	NaN	NaN	NaN	NaN	NaN	NaN	NaN
NaN	NaN	NaN	NaN	NaN	NaN	NaN	NaN	NaN
NaN	NaN	NaN	NaN	NaN	NaN	NaN	NaN	NaN
23,1611	23,3312	24,5185	24,4871	23,7419	23,565	23,3218	23,246	23,3423
NaN	NaN	22,9361	23,3184	NaN	NaN	20,2747	20,4245	21,0537
NaN	21,1379	19,719	20,2238	NaN	NaN	NaN	NaN	NaN
NaN	NaN	23,8511	24,1377	22,2085	21,9157	22,0118	22,0038	22,2368
20,3684	NaN	NaN	NaN	NaN	NaN	20,9763	21,2563	21,7658
NaN	NaN	NaN	NaN	21,5975	NaN	21,2333	21,517	NaN

NaN	20,1652	19,9915	19,8026	NaN	NaN	20,2154	20,2148	NaN
24,0181	23,7779	24,834	24,8375	25,3166	25,4349	23,9795	23,9939	24,8615
NaN	NaN	20,0242	20,246	NaN	NaN	NaN	NaN	NaN
NaN	21,8721	NaN	NaN	NaN	NaN	NaN	NaN	NaN
23,5009	23,4408	21,1939	21,3648	22,8457	22,9585	22,9659	23,0568	22,4802
NaN	NaN	20,646	20,8121	20,8134	20,5044	21,1408	NaN	21,2996
25,765	25,8648	23,9613	23,8356	24,7394	24,7281	24,2106	24,2901	23,7867
23,285	23,2008	20,5485	20,3975	22,9994	22,8189	21,1847	20,9803	20,9292
NaN	NaN	20,0398	20,0799	NaN	20,7088	20,732	20,6198	NaN
NaN	NaN	22,8331	22,8577	22,2387	21,9386	NaN	21,533	NaN
NaN	NaN	21,9633	21,9643	22,337	22,3171	22,8552	22,3749	23,3823
20,7097	21,2676	23,7786	23,5718	22,2546	22,0997	20,8975	21,0871	21,7091
NaN	NaN	21,9691	22,2705	NaN	NaN	21,6424	21,9433	21,3535
NaN	NaN	20,6425	21,3944	NaN	NaN	NaN	NaN	NaN
NaN	NaN	21,6444	21,5861	NaN	NaN	NaN	19,6862	NaN
NaN	NaN	20,1763	20,3353	NaN	NaN	NaN	NaN	NaN
23,0012	23,1459	24,7143	24,788	23,6082	23,6206	23,6913	23,6269	23,5563
NaN	NaN	NaN	NaN	NaN	NaN	NaN	NaN	NaN
26,7313	26,8782	25,0295	25,0574	26,1456	26,4321	25,8882	25,9376	25,8557
25,5729	25,5456	27,494	27,5153	26,803	26,5276	26,1936	26,3545	26,7354
NaN	NaN	22,4134	21,8633	NaN	NaN	NaN	NaN	NaN
NaN	NaN	20,7313	20,6789	NaN	NaN	NaN	NaN	NaN
25,5296	25,7266	22,6817	23,5545	25,8167	26,1177	26,5567	26,613	27,62
21,9223	21,7906	23,4777	23,6253	22,0482	21,9786	22,0666	22,2139	21,9317
NaN	NaN	18,7488	19,2075	NaN	NaN	20,8868	NaN	19,9482
NaN	NaN	21,1721	20,1468	NaN	NaN	NaN	NaN	NaN
23,0236	22,9572	21,4172	21,6177	22,4271	22,4379	22,2737	21,9538	22,5188
NaN	NaN	21,3279	20,8335	NaN	NaN	NaN	NaN	NaN
NaN	NaN	20,0346	20,6694	20,5271	20,8906	NaN	20,1034	19,9713
NaN	NaN	21,1535	21,2661	NaN	NaN	20,4803	NaN	NaN
NaN	NaN	20,6495	20,651	NaN	20,2923	20,0929	NaN	20,251
NaN	NaN	20,1129	20,3505	NaN	NaN	19,8221	NaN	19,7668
NaN	20,8143	NaN	NaN	21,8523	22,036	20,9361	21,1355	20,7792
NaN	NaN	19,907	19,905	NaN	NaN	NaN	NaN	NaN
NaN	NaN	20,8404	20,947	NaN	NaN	NaN	NaN	NaN
NaN	NaN	20,6116	20,3896	20,7706	20,6844	20,36	20,4143	20,5145
21,1863	21,2982	22,5431	22,387	NaN	21,7475	22,3081	21,9452	22,1096
21,8169	21,8854	21,0217	20,9025	21,2326	21,1265	21,0366	21,1809	20,98
NaN	NaN	20,8608	21,1239	NaN	NaN	NaN	NaN	NaN
22,1884	22,3888	22,5097	22,5864	21,3614	21,2895	20,8235	20,6235	20,921
20,9342	21,2173	NaN	NaN	20,2715	NaN	20,4964	20,0447	20,4261
21,0622	21,0558	22,5853	22,6155	20,5605	20,8424	20,1901	NaN	20,2731
NaN	21,0334	21,035	21,535	20,9674	21,546	21,2121	21,205	20,8672
21,8443	NaN	21,871	21,788	NaN	NaN	NaN	NaN	NaN
23,1308	23,3604	20,605	20,1309	20,9857	21,3356	21,5079	21,7067	21,3657
NaN	NaN	18,2242	20,3063	NaN	NaN	24,3172	24,3125	NaN
NaN	NaN	21,2292	21,5769	20,2922	NaN	NaN	NaN	20,6137
NaN	NaN	22,2041	22,2277	22,0663	22,287	21,9006	21,7229	21,6576
22,5112	22,9694	21,3149	21,3024	23,1915	23,0362	22,9006	22,6595	22,8796
NaN	NaN	20,874	20,2832	NaN	19,8587	NaN	NaN	NaN
22,026	21,9426	NaN	19,3503	22,233	21,8669	22,1876	22,4291	22,2353
NaN	NaN	19,9767	20,0636	NaN	NaN	NaN	NaN	NaN

NaN	NaN	22,4726	21,9491	NaN	NaN	NaN	NaN	21,6507
22,0893	22,3678	21,5307	21,4543	NaN	NaN	21,3272	21,2374	NaN
NaN	21,199	NaN	NaN	NaN	NaN	NaN	NaN	20,4327
25,1533	25,1877	26,3318	26,34	24,6346	24,6348	24,7796	24,7562	24,0023
NaN	NaN	NaN	NaN	NaN	NaN	NaN	NaN	NaN
22,0257	21,9923	20,848	20,7312	21,5378	21,9337	21,4298	21,4801	21,2873
24,6398	24,7191	27,7798	27,813	26,1747	26,0138	26,3552	26,4555	27,1959
21,2133	21,601	22,311	22,2282	21,7268	21,1985	21,8079	21,3004	21,3883
22,886	21,8677	23,1522	23,0561	21,6952	NaN	21,8705	21,8845	NaN
NaN	NaN	21,0509	20,7008	NaN	NaN	NaN	19,6497	NaN
20,7861	21,0196	21,8269	21,5086	NaN	NaN	21,0648	21,1884	21,469
28,3219	28,2665	27,1177	27,1147	25,383	25,4406	25,9193	26,0583	25,092
NaN	NaN	23,1149	22,9963	22,1903	22,5195	22,7807	22,9541	NaN
NaN	NaN	NaN	NaN	NaN	NaN	NaN	NaN	NaN
20,9536	20,7422	22,1744	22,2656	20,4159	NaN	20,5223	20,6374	NaN
21,1483	21,2137	21,3178	21,193	NaN	NaN	NaN	20,296	20,6444
21,3608	21,7601	23,5835	23,7329	NaN	21,2492	20,3542	NaN	21,6047
NaN	NaN	NaN	NaN	23,4359	22,4873	NaN	NaN	NaN
NaN	NaN	23,2199	22,7611	21,6877	22,4019	23,0202	22,5184	21,8185
20,7218	NaN	23,1804	23,0644	20,7694	NaN	20,2934	19,9781	20,8238
24,372	24,3992	25,9342	25,9301	24,4592	24,5045	23,453	23,413	24,3751
21,8436	21,4944	22,4222	22,7068	20,7002	20,9958	NaN	20,8873	NaN
26,5468	26,8082	26,9804	26,6305	26,7491	26,761	26,3412	26,3726	26,0084
NaN	NaN	24,3541	24,2996	25,5568	25,5479	24,2304	24,0827	25,2266
NaN	NaN	21,6652	21,3987	NaN	NaN	NaN	NaN	20,3223
21,4756	NaN	22,5517	22,403	22,9803	23,2693	23,7624	23,9277	22,5779
NaN	NaN	NaN	NaN	NaN	NaN	NaN	NaN	NaN
23,0142	23,1094	24,2142	23,8684	23,2562	NaN	23,1006	23,22	23,6765
27,0276	27,0128	22,4081	22,569	25,0422	24,9237	26,7108	26,75	26,1182
NaN	21,8826	22,7909	22,7024	NaN	21,5911	22,9343	22,8297	21,0113
NaN	NaN	NaN	NaN	NaN	NaN	NaN	NaN	NaN
25,387	25,3439	24,4439	24,3437	23,4858	23,5315	26,5	26,37	25,532
23,3652	23,3403	22,8976	22,8152	22,6193	22,5697	22,8442	22,783	22,0737
24,0404	24,2746	23,5283	23,5898	23,717	23,9946	23,7787	23,564	23,466
NaN	NaN	20,1818	20,8691	21,2795	NaN	20,8676	20,8858	NaN
NaN	NaN	22,346	21,4839	NaN	NaN	NaN	NaN	NaN
NaN	NaN	22,9987	23,2388	21,4788	21,9881	NaN	21,2698	21,9838
21,6467	21,3154	20,4615	20,5418	21,1544	20,6994	20,6601	20,7332	20,5184
20,1788	20,0095	22,9259	22,9534	21,0424	21,2842	20,7434	20,8569	21,5893
NaN	NaN	20,8864	20,9891	20,8678	21,3847	20,3295	20,3375	NaN
26,3827	26,4733	23,0608	23,1865	23,0055	22,8773	25,6522	25,7242	24,0586
NaN	NaN	22,4784	22,5545	NaN	NaN	21,2531	NaN	21,0665
NaN	NaN	21,5736	21,6602	NaN	NaN	NaN	NaN	NaN
NaN	NaN	21,4602	21,5014	NaN	NaN	20,6639	21,3724	NaN
21,084	21,1407	21,4333	22,417	NaN	NaN	NaN	NaN	NaN
NaN	NaN	21,1576	20,9176	NaN	20,3784	NaN	NaN	21,4187
NaN	NaN	21,4205	21,8389	NaN	NaN	NaN	NaN	NaN
NaN	NaN	21,3387	21,4236	NaN	NaN	NaN	20,78	20,5707
NaN	NaN	20,9302	20,6447	NaN	NaN	NaN	NaN	NaN
NaN	NaN	20,2576	20,101	NaN	NaN	NaN	NaN	NaN
23,2874	23,2727	23,2466	23,3098	22,6983	22,3159	22,6303	22,4501	21,3764
21,4074	21,3725	21,6278	21,1794	NaN	NaN	NaN	20,5135	20,4451

NaN	NaN	21,0993	20,8418	NaN	NaN	NaN	NaN	NaN
23,6377	NaN	24,4912	24,4666	24,0165	23,8723	23,7027	23,68	21,3743
27,7182	27,68	25,9416	25,8947	26,5129	26,4963	26,4741	26,4182	26,3756
26,1608	26,2002	24,7427	24,8942	25,4707	25,4824	24,8046	24,7052	24,1877
25,6668	27,5189	25,4697	25,4484	25,7333	26,0759	25,465	25,4691	25,1289
25,3673	25,2524	25,9814	25,9711	24,2186	24,0221	24,2282	24,3338	24,3799
NaN	NaN	NaN	20,7565	NaN	NaN	NaN	NaN	NaN
NaN	NaN	20,2402	20,0442	NaN	NaN	NaN	NaN	NaN
NaN	NaN	20,69	20,7326	NaN	NaN	NaN	NaN	NaN
NaN	19,9357	22,21	21,8819	20,7804	20,8171	NaN	NaN	20,2414
NaN	NaN	20,5968	20,8381	NaN	NaN	NaN	NaN	20,3113
NaN	NaN	21,2879	21,8267	NaN	NaN	NaN	NaN	NaN
NaN	20,8214	NaN	NaN	20,532	NaN	NaN	NaN	NaN
NaN	NaN	NaN	NaN	NaN	NaN	NaN	NaN	NaN
NaN	NaN	20,8651	20,7143	NaN	NaN	NaN	NaN	NaN
NaN	NaN	21,4067	21,1237	NaN	NaN	NaN	NaN	NaN
NaN	20,243	21,1391	21,2455	20,8073	20,5735	21,6514	21,8167	21,6823
NaN	NaN	NaN	NaN	NaN	NaN	NaN	NaN	NaN
NaN	NaN	NaN	NaN	NaN	NaN	NaN	NaN	NaN
21,1227	NaN	21,5776	21,6155	NaN	NaN	NaN	20,0721	NaN
22,4156	22,4426	21,1923	21,1675	NaN	NaN	21,8034	21,8783	20,5706
NaN	NaN	NaN	18,7353	NaN	NaN	NaN	NaN	NaN
NaN	NaN	22,6792	22,738	NaN	NaN	21,474	NaN	NaN
NaN	NaN	21,238	21,1848	NaN	NaN	NaN	NaN	21,334
22,13	22,1091	19,9401	20,871	21,2043	21,1819	21,7598	21,5276	21,4012
20,5451	NaN	21,6047	21,9203	20,6727	20,4856	20,6509	20,7402	20,7677
22,1041	22,3865	24,0444	24,0222	NaN	NaN	23,0647	22,942	22,333
NaN	NaN	21,1733	20,8784	NaN	NaN	NaN	NaN	NaN
24,2638	24,1609	24,1597	25,0628	24,4336	24,4471	23,7971	23,8528	24,098
NaN	20,6627	21,2161	21,3962	NaN	NaN	NaN	NaN	NaN
20,5304	20,3663	21,632	21,6468	NaN	NaN	21,1673	20,7352	NaN
21,9071	20,9053	23,6959	23,679	21,9897	22,4115	21,9952	22,1842	22,4899
NaN	NaN	21,3565	20,9538	NaN	NaN	NaN	NaN	NaN
NaN	NaN	21,7447	21,6953	NaN	NaN	20,8727	21,1674	20,3281
NaN	NaN	20,773	20,2806	NaN	NaN	NaN	20,5441	20,0528
NaN	NaN	21,0293	21,0282	NaN	NaN	20,6581	NaN	NaN
NaN	NaN	21,5515	21,1047	NaN	NaN	NaN	NaN	NaN
NaN	NaN	21,5842	22,4135	NaN	NaN	NaN	NaN	NaN
NaN	NaN	20,8925	21,0816	NaN	NaN	NaN	NaN	NaN
NaN	NaN	NaN	NaN	NaN	NaN	NaN	NaN	NaN
NaN	NaN	22,1097	21,0315	NaN	NaN	NaN	NaN	NaN
NaN	NaN	NaN	NaN	NaN	NaN	NaN	NaN	NaN
NaN	NaN	18,7993	18,0389	NaN	NaN	NaN	NaN	NaN
22,5581	22,5633	21,5872	21,5649	21,61	21,6327	22,1547	22,3576	22,3126
NaN	NaN	20,0593	20,4733	21,0192	21,0048	NaN	NaN	NaN
21,9345	NaN	22,2462	22,1066	NaN	NaN	22,3286	NaN	22,1908
21,7601	22,1066	22,9396	22,925	NaN	20,9716	20,851	21,0689	NaN
NaN	NaN	19,1669	19,9114	NaN	NaN	NaN	NaN	NaN
NaN	NaN	21,7808	21,8643	20,6606	NaN	20,6268	20,7263	20,6666
NaN	NaN	NaN	NaN	NaN	NaN	NaN	NaN	NaN
NaN	NaN	21,5896	21,5239	NaN	NaN	22,5711	22,1978	NaN
NaN	NaN	21,1373	21,3061	20,4023	20,6245	21,5281	21,555	21,5602

NaN	NaN	23,8728	23,7984	NaN	NaN	20,2045	20,6594	NaN
NaN	NaN	20,3951	20,4951	20,9224	NaN	20,5402	NaN	NaN
NaN	NaN	22,2706	22,3402	NaN	NaN	NaN	20,6489	20,8674
NaN	NaN	21,6863	21,7449	20,4622	NaN	20,0855	NaN	20,5222
NaN	NaN	22,4549	22,4751	20,8877	20,4395	20,6308	20,9765	20,4071
NaN	NaN	21,4201	21,1811	NaN	NaN	20,9644	20,6584	20,5033
NaN	NaN	20,8895	21,3084	NaN	NaN	NaN	NaN	NaN
NaN	NaN	21,5134	21,3337	NaN	NaN	21,9144	22,1708	21,414
NaN	NaN	20,1795	19,9809	NaN	NaN	NaN	NaN	NaN
NaN	NaN	20,5007	20,5397	NaN	NaN	NaN	20,5463	20,001
NaN	NaN	NaN	NaN	NaN	NaN	NaN	NaN	NaN
NaN	NaN	NaN	NaN	NaN	NaN	NaN	NaN	NaN
23,4664	23,4563	23,2797	23,2467	23,9348	23,9579	23,2784	23,2686	23,717
NaN	NaN	21,4266	NaN	NaN	NaN	NaN	NaN	NaN
NaN	NaN	NaN	NaN	NaN	NaN	NaN	NaN	NaN
20,5214	20,882	21,1526	21,0245	NaN	NaN	20,433	20,5025	20,4616
NaN	NaN	23,0321	23,0033	NaN	NaN	NaN	NaN	21,4885
26,5146	26,663	24,3617	24,1683	25,4537	25,3194	27,155	27,2904	27,1248
NaN	NaN	22,8435	22,5672	NaN	NaN	21,0389	21,7053	20,6541
23,1984	NaN	NaN	21,7977	24,0278	24,2104	23,4502	23,5335	23,5308
20,9187	NaN	21,0248	21,5944	NaN	NaN	NaN	NaN	NaN
NaN	NaN	NaN	NaN	NaN	NaN	NaN	20,6795	NaN
23,7464	23,2943	20,764	21,0689	NaN	NaN	20,2946	20,0367	21,4751
NaN	NaN	NaN	NaN	20,8329	21,1226	21,1864	21,2545	21,086
26,6859	26,5863	23,3948	23,526	24,6415	24,6829	24,1188	24,2181	23,8954
NaN	NaN	20,9436	21,6025	NaN	NaN	NaN	NaN	NaN
NaN	NaN	20,5118	20,7978	NaN	NaN	NaN	20,7598	NaN
NaN	NaN	22,0925	22,3052	NaN	NaN	NaN	NaN	NaN
NaN	NaN	20,0155	20,5197	NaN	NaN	NaN	NaN	NaN
NaN	NaN	20,3738	20,8278	21,5346	NaN	21,6014	NaN	NaN
20,7112	20,9549	22,201	21,9417	NaN	NaN	NaN	NaN	21,1041
20,3337	20,5611	22,1113	22,0902	20,6634	NaN	20,5005	20,4805	20,4901
NaN	NaN	20,8842	20,239	NaN	NaN	NaN	NaN	19,5302
19,3594	NaN	19,9393	19,6399	NaN	18,9333	NaN	NaN	18,3714
NaN	NaN	NaN	NaN	NaN	NaN	NaN	NaN	21,4097
21,6971	21,5487	NaN	20,5082	22,7539	22,6961	21,0288	21,3614	21,7636
NaN	NaN	20,364	20,5364	NaN	NaN	20,045	20,2915	NaN
NaN	NaN	20,7805	20,8229	NaN	NaN	NaN	NaN	NaN
NaN	NaN	20,8467	20,4432	NaN	NaN	NaN	NaN	NaN
NaN	NaN	NaN	NaN	21,2145	NaN	20,5223	NaN	21,8549
NaN	NaN	19,9869	19,6768	NaN	NaN	NaN	NaN	NaN
NaN	NaN	NaN	NaN	NaN	21,1666	21,981	21,8448	22,0633
NaN	NaN	20,6468	20,5076	NaN	NaN	NaN	NaN	20,8491
22,6201	22,6009	23,6576	23,6785	22,9988	22,4536	22,4989	22,6266	22,5341
NaN	NaN	21,7856	21,7866	NaN	NaN	20,4523	20,5624	NaN
NaN	NaN	21,4151	21,5871	NaN	20,7569	NaN	NaN	NaN
NaN	NaN	21,8985	21,4844	23,2805	23,4121	21,9727	21,6885	23,5967
NaN	NaN	20,0169	19,1536	NaN	NaN	NaN	NaN	NaN
NaN	NaN	21,1908	21,3488	NaN	NaN	20,5648	NaN	19,9086
NaN	NaN	20,3873	20,2579	NaN	NaN	NaN	NaN	NaN
NaN	NaN	22,1643	22,3259	22,6985	22,6233	21,6122	21,8022	20,933
24,8208	24,879	24,2623	24,3213	23,6091	23,1481	23,9849	24,2651	23,5238

NaN	NaN	NaN	NaN	NaN	NaN	NaN	NaN	NaN
NaN	NaN	18,9059	18,8831	19,8326	NaN	20,2815	20,3627	20,7685
NaN	NaN	19,1043	NaN	NaN	NaN	NaN	NaN	NaN
NaN	NaN	21,0977	21,0878	NaN	NaN	20,2074	20,2056	NaN
NaN	NaN	19,7908	19,5882	NaN	NaN	21,4457	20,9414	21,4736
NaN	NaN	NaN	NaN	NaN	NaN	NaN	NaN	NaN
NaN	NaN	NaN	NaN	NaN	NaN	NaN	NaN	NaN
NaN	NaN	NaN	NaN	NaN	NaN	NaN	NaN	NaN
23,3892	23,3264	NaN	NaN	NaN	NaN	NaN	NaN	NaN
24,2116	24,0352	25,0083	25,0177	23,2469	23,3062	23,5766	23,0875	23,6122
21,3156	NaN	20,3614	20,2834	20,8309	NaN	21,4692	21,4645	21,6372
23,3745	23,4	22,5626	22,4993	22,6064	22,7509	22,9374	22,9479	23,1706
NaN	NaN	NaN	NaN	NaN	NaN	20,06	NaN	NaN
26,1892	26,1996	23,5329	23,4328	25,6395	25,4522	25,7461	25,6993	25,6703
NaN	NaN	19,5081	19,1715	20,6387	20,1807	20,7314	20,0628	20,1841
NaN	20,6078	21,7739	21,7613	20,9828	20,6382	20,7441	20,6426	20,0649
21,4473	NaN	23,6492	23,6998	23,3312	23,6804	21,9431	22,2254	23,014
NaN	NaN	20,1371	19,816	NaN	NaN	NaN	NaN	NaN
NaN	NaN	NaN	NaN	NaN	NaN	20,9214	20,8988	25,7733
23,4828	23,536	24,8722	24,7882	23,4225	23,2697	22,8035	22,6796	23,8478
NaN	NaN	NaN	NaN	NaN	NaN	NaN	NaN	NaN
23,5286	22,9244	24,2074	24,2816	22,8465	22,9905	22,8948	23,0968	22,8276
NaN	NaN	NaN	NaN	22,0809	23,0966	NaN	NaN	21,577
NaN	NaN	20,1819	20,9814	NaN	NaN	NaN	NaN	NaN
NaN	NaN	NaN	NaN	NaN	NaN	NaN	NaN	NaN
21,9919	22,3028	23,9161	23,9472	22,6574	22,5661	22,0286	21,8306	23,335
23,6652	23,6641	23,8774	23,9919	NaN	23,2008	23,1701	23,1144	23,2707
NaN	NaN	21,5161	21,6073	NaN	NaN	NaN	NaN	NaN
19,9105	20,0113	19,277	19,6111	19,1802	19,0758	20,1911	NaN	19,6429
NaN	NaN	NaN	NaN	NaN	NaN	21,0847	NaN	19,9675
20,3066	NaN	21,8398	22,0006	19,969	19,8706	NaN	19,8938	NaN
21,3533	21,8995	23,2508	23,0336	20,9229	20,7429	22,2207	22,1518	21,3718
NaN	NaN	NaN	NaN	NaN	NaN	NaN	NaN	NaN
NaN	NaN	NaN	19,7253	23,065	22,3354	21,8568	21,8715	23,7417
NaN	NaN	24,6135	24,5684	NaN	NaN	21,6944	22,1363	NaN
NaN	NaN	NaN	NaN	NaN	NaN	NaN	NaN	23,0503
NaN	NaN	21,3077	NaN	NaN	NaN	NaN	NaN	NaN
NaN	NaN	21,2476	21,1715	20,721	20,505	20,6121	20,6582	20,3915
25,8044	25,9387	28,5897	28,611	29,1767	29,3253	29,1872	29,1841	29,6002
NaN	NaN	23,2628	23,0441	21,4032	NaN	21,0786	NaN	21,1334
NaN	NaN	20,6824	21,4933	NaN	NaN	20,4075	20,57	20,3529
22,9486	22,8911	20,4262	20,3685	NaN	NaN	22,8887	23,1425	23,2925
20,7238	20,6511	20,3759	21,0473	NaN	20,3979	NaN	NaN	20,9123
NaN	NaN	21,9176	22,0824	21,6802	NaN	21,5636	21,5027	21,259
NaN	NaN	20,3776	20,105	NaN	NaN	NaN	NaN	NaN
25,007	25,1052	NaN	22,426	23,2707	23,261	23,4096	23,6764	23,8639
21,7925	22,1633	22,6244	22,6842	NaN	NaN	NaN	NaN	NaN
NaN	NaN	NaN	NaN	NaN	NaN	NaN	NaN	21,2519
24,4504	24,1223	20,2876	20,4977	22,1469	22,1019	22,985	23,6677	NaN
NaN	NaN	NaN	NaN	23,2153	23,7712	23,4125	22,9744	24,9475
NaN	NaN	26,844	26,8775	29,1971	28,9636	28,1729	28,2878	NaN
NaN	NaN	NaN	NaN	NaN	NaN	NaN	NaN	NaN

NaN	NaN	20,916	21,1738	NaN	NaN	20,4733	NaN	20,6648
21,9854	21,9338	23,3281	23,4636	21,9417	21,9853	22,0288	21,7683	22,5879
NaN	NaN	23,2551	23,3277	NaN	NaN	NaN	NaN	NaN
NaN	NaN	19,1569	19,8082	NaN	NaN	NaN	NaN	NaN
NaN	NaN	NaN	NaN	NaN	NaN	NaN	NaN	NaN
22,6187	22,805	NaN	20,9001	NaN	NaN	22,0247	22,4324	NaN
NaN	NaN	19,18	19,6879	NaN	NaN	NaN	NaN	19,655
20,4429	20,4249	21,6396	21,7173	NaN	NaN	20,7924	NaN	20,6453
NaN	NaN	NaN	NaN	NaN	NaN	NaN	NaN	NaN
21,6477	21,2713	19,4585	NaN	21,1953	21,3133	21,2697	20,9637	NaN
NaN	NaN	19,9388	20,115	NaN	NaN	NaN	NaN	NaN
NaN	NaN	21,1342	21,019	NaN	NaN	19,9271	NaN	NaN
NaN	NaN	19,6095	19,8091	NaN	NaN	NaN	NaN	NaN
NaN	NaN	NaN	NaN	NaN	NaN	NaN	NaN	NaN
NaN	NaN	NaN	NaN	20,8872	20,8987	21,1095	20,7802	20,422
NaN	NaN	NaN	18,973	NaN	NaN	19,4672	NaN	NaN
NaN	NaN	18,5129	18,342	NaN	NaN	NaN	NaN	NaN
NaN	NaN	NaN	NaN	NaN	NaN	NaN	NaN	NaN
NaN	NaN	NaN	NaN	NaN	NaN	NaN	NaN	NaN
NaN	NaN	19,8906	19,9978	NaN	NaN	NaN	NaN	NaN
19,9559	NaN	20,1782	20,176	NaN	NaN	20,3482	NaN	19,8117
NaN	NaN	20,2117	19,8671	NaN	NaN	NaN	NaN	NaN
NaN	NaN	20,8583	20,334	NaN	NaN	NaN	NaN	NaN
NaN	NaN	NaN	NaN	NaN	NaN	NaN	NaN	NaN
NaN	NaN	NaN	NaN	NaN	NaN	NaN	NaN	NaN
NaN	NaN	19,534	20,0839	NaN	NaN	NaN	NaN	NaN
19,9446	NaN	21,726	21,5384	NaN	NaN	NaN	NaN	NaN
NaN	NaN	NaN	19,3954	NaN	NaN	NaN	NaN	NaN
21,6502	NaN	22,3563	22,489	NaN	21,9406	25,4932	25,5417	NaN
NaN	NaN	21,0179	20,5851	NaN	NaN	NaN	NaN	NaN
23,3586	23,605	21,8087	NaN	23,1856	23,1961	NaN	22,147	22,3011
22,4571	22,4231	20,8696	20,9381	21,525	21,6247	21,4392	21,8535	20,8989
NaN	NaN	NaN	NaN	NaN	NaN	NaN	NaN	NaN
21,726	22,0001	NaN	NaN	NaN	NaN	NaN	21,3485	21,2077
NaN	NaN	19,6183	20,0624	NaN	NaN	NaN	NaN	NaN
22,6356	22,401	25,5346	25,4765	22,9847	23,1665	22,0285	21,9218	23,7281
NaN	NaN	20,655	NaN	NaN	NaN	NaN	NaN	NaN
NaN	NaN	21,1501	21,439	NaN	NaN	NaN	NaN	NaN
26,1117	25,7977	NaN	23,577	NaN	NaN	NaN	NaN	NaN
23,2835	24,0646	NaN	21,1519	22,6936	22,3182	22,2332	22,7425	NaN
NaN	NaN	NaN	20,6702	NaN	NaN	NaN	NaN	NaN
26,5631	26,441	23,2215	23,209	24,7336	24,6196	23,5416	23,6805	24,6112
NaN	NaN	20,0942	19,9831	NaN	NaN	19,8623	NaN	NaN
NaN	NaN	NaN	NaN	NaN	NaN	NaN	NaN	NaN
NaN	NaN	NaN	19,9922	NaN	NaN	NaN	NaN	NaN
21,2437	21,4565	22,4358	21,8232	20,7662	NaN	NaN	NaN	20,676
22,9262	23,2627	NaN	NaN	22,6137	23,1317	NaN	NaN	NaN
NaN	NaN	20,431	20,3981	NaN	NaN	NaN	NaN	NaN
23,4948	23,4876	22,4162	22,3204	21,6084	NaN	22,2434	22,1057	21,4977
NaN	NaN	NaN	NaN	NaN	NaN	NaN	NaN	NaN
22,3549	22,4505	21,4103	21,3362	22,6729	22,7014	22,696	22,7815	21,8111
25,9935	26,0343	24,4646	24,2675	24,9523	25,1298	25,1583	24,8224	24,0833

NaN	NaN	21,272	21,3983	NaN	NaN	NaN	NaN	NaN
NaN	NaN	NaN	NaN	NaN	NaN	NaN	NaN	NaN
23,3226	23,0789	21,4061	21,4713	NaN	22,4173	22,9269	22,9478	22,1687
NaN	NaN	20,5893	21,2883	20,6887	20,3698	20,6772	NaN	NaN
NaN	NaN	NaN	NaN	NaN	NaN	NaN	NaN	NaN
NaN	NaN	20,8612	20,5331	NaN	NaN	NaN	NaN	NaN
NaN	NaN	19,4385	19,5534	NaN	NaN	NaN	NaN	NaN
NaN	NaN	21,3882	21,3366	NaN	NaN	NaN	NaN	NaN
NaN	NaN	20,2649	NaN	NaN	NaN	20,4046	NaN	NaN
NaN	NaN	19,5148	NaN	NaN	NaN	NaN	NaN	NaN
NaN	NaN	20,0953	19,8074	NaN	NaN	NaN	NaN	NaN
NaN	NaN	NaN	NaN	NaN	NaN	NaN	NaN	NaN
NaN	NaN	NaN	NaN	NaN	NaN	NaN	NaN	NaN
21,85	21,6479	21,9112	22,0061	NaN	NaN	NaN	20,915	21,2436
NaN	NaN	21,6281	21,8049	NaN	NaN	NaN	NaN	NaN
NaN	NaN	20,7698	20,5303	NaN	20,7729	NaN	NaN	NaN
NaN	NaN	21,6352	21,5985	NaN	NaN	NaN	NaN	NaN
NaN	NaN	20,9203	20,7345	20,8999	21,2266	20,8275	21,1817	21,1799
20,7492	20,837	19,6687	19,6469	NaN	NaN	20,6867	20,7418	NaN
22,5075	21,8515	22,8478	22,8473	NaN	NaN	NaN	NaN	NaN
NaN	20,8362	21,3562	20,828	21,2768	NaN	NaN	21,6877	21,0149
NaN	NaN	NaN	NaN	NaN	NaN	NaN	NaN	NaN
NaN	NaN	NaN	NaN	20,9163	20,778	21,6888	21,6476	21,8878
NaN	NaN	19,7718	20,3764	NaN	NaN	NaN	NaN	NaN
NaN	NaN	NaN	NaN	NaN	NaN	NaN	NaN	NaN
NaN	NaN	NaN	19,8731	NaN	NaN	NaN	NaN	NaN
NaN	NaN	19,7881	19,7983	NaN	NaN	NaN	NaN	NaN
NaN	NaN	NaN	NaN	NaN	NaN	NaN	NaN	NaN
NaN	NaN	20,3772	20,0764	NaN	NaN	NaN	NaN	NaN
NaN	NaN	NaN	NaN	NaN	NaN	NaN	NaN	20,5619
NaN	NaN	NaN	21,5369	NaN	NaN	NaN	NaN	NaN
NaN	NaN	22,0397	22,1739	22,2494	21,8739	21,5108	21,3138	21,9665
NaN	NaN	NaN	NaN	NaN	NaN	NaN	NaN	NaN
NaN	NaN	NaN	19,3357	NaN	NaN	NaN	NaN	NaN
NaN	NaN	21,2134	21,3864	NaN	NaN	NaN	NaN	21,1538
21,7988	21,5868	NaN	NaN	NaN	NaN	NaN	NaN	NaN
21,2766	NaN	22,1715	22,846	22,2791	21,55	23,9457	23,9129	23,1082
NaN	NaN	NaN	NaN	29,1943	29,8226	27,811	28,0848	30,1681
24,1022	NaN	24,712	24,6755	NaN	NaN	NaN	NaN	23,2148
NaN	NaN	NaN	19,2681	NaN	NaN	20,2081	NaN	NaN
20,0622	NaN	20,7262	20,2113	NaN	NaN	20,3063	NaN	NaN
25,8702	25,8032	24,202	24,2238	25,1663	25,3327	24,5896	24,5374	24,47
NaN	21,1482	23,1228	22,9796	21,6362	22,3635	NaN	NaN	22,2922
NaN	NaN	21,3155	20,9585	NaN	NaN	NaN	NaN	NaN
NaN	NaN	21,1951	21,1639	NaN	NaN	NaN	NaN	NaN
NaN	NaN	19,9662	NaN	NaN	NaN	NaN	NaN	NaN
NaN	NaN	NaN	NaN	NaN	NaN	NaN	NaN	NaN
NaN	NaN	NaN	21,7044	NaN	NaN	NaN	NaN	NaN
NaN	NaN	21,4623	21,8134	NaN	NaN	NaN	NaN	NaN
NaN	NaN	21,3688	21,5871	21,0284	21,355	21,1071	21,1321	21,1785
NaN	NaN	NaN	NaN	NaN	NaN	NaN	NaN	NaN
NaN	NaN	NaN	NaN	NaN	NaN	NaN	NaN	NaN

NaN	NaN	18,9121	19,0672	NaN	NaN	NaN	18,8906	NaN
NaN	NaN	NaN	23,2438	21,3776	21,7848	NaN	21,97	21,5121
NaN	NaN	19,5848	19,4325	19,8872	NaN	NaN	NaN	NaN
20,5234	20,5677	20,6659	20,4657	NaN	NaN	NaN	NaN	NaN
NaN	NaN	NaN	NaN	NaN	NaN	NaN	NaN	NaN
NaN	NaN	22,485	22,1723	22,6249	22,9814	22,2579	NaN	21,7215
22,2148	22,4867	21,728	NaN	23,2106	NaN	22,4225	22,4959	21,9265
25,4732	NaN	25,5405	25,6886	24,607	24,5997	25,3493	25,3623	25,3034
NaN	NaN	19,1269	NaN	NaN	NaN	NaN	NaN	NaN
NaN	NaN	NaN	NaN	NaN	NaN	NaN	NaN	NaN
24,5895	24,2484	22,3969	22,1866	NaN	NaN	22,2056	21,9469	23,2094
23,9119	NaN	22,4521	21,6999	NaN	NaN	23,6725	23,6499	22,5465
NaN	NaN	19,3277	19,6338	NaN	NaN	NaN	NaN	NaN
24,0182	NaN	22,8542	22,8511	NaN	NaN	NaN	NaN	23,1603
NaN	NaN	NaN	NaN	NaN	NaN	NaN	NaN	NaN
26,3092	26,2893	24,0577	23,8947	23,7669	23,6767	23,7435	23,6824	23,2628
NaN	NaN	NaN	NaN	NaN	NaN	NaN	NaN	NaN
NaN	NaN	NaN	NaN	NaN	NaN	NaN	20,4831	20,5629
NaN	20,4809	NaN	NaN	NaN	NaN	20,7902	20,8932	20,2853
NaN	25,7046	23,3001	23,1624	23,9687	24,0465	NaN	NaN	NaN
NaN	NaN	NaN	NaN	NaN	NaN	NaN	NaN	NaN
20,998	21,3851	21,5028	21,7738	20,4717	NaN	NaN	20,6563	20,5101
23,3836	23,5342	21,4522	21,6757	NaN	NaN	23,3627	NaN	NaN
NaN	NaN	24,4113	24,3398	NaN	NaN	NaN	NaN	NaN
NaN	NaN	NaN	NaN	NaN	NaN	NaN	NaN	22,0655
21,1637	21,2615	20,0197	19,9181	20,9942	20,9408	20,4671	20,7002	20,3008
23,2469	21,9327	NaN	20,9992	NaN	21,0594	20,9442	21,1615	NaN
23,7228	23,6751	21,5697	21,7699	23,8616	23,6321	22,7123	22,788	23,4975
NaN	NaN	22,1573	21,7376	NaN	NaN	NaN	NaN	NaN
NaN	NaN	NaN	19,5463	NaN	NaN	NaN	NaN	NaN
NaN	NaN	NaN	NaN	NaN	NaN	NaN	NaN	NaN
NaN	NaN	19,2233	19,3963	NaN	NaN	NaN	NaN	NaN
NaN	20,3091	NaN	NaN	NaN	NaN	NaN	NaN	NaN
NaN	NaN	20,7733	20,5974	NaN	NaN	20,8858	20,8229	20,7772
21,5286	22,1416	19,227	19,2573	NaN	NaN	NaN	21,668	21,0321
NaN	NaN	19,4596	19,4341	NaN	NaN	NaN	NaN	NaN
NaN	NaN	21,6624	21,6038	NaN	NaN	NaN	NaN	NaN
NaN	NaN	NaN	NaN	NaN	NaN	NaN	NaN	NaN
NaN	NaN	NaN	NaN	NaN	NaN	NaN	20,6637	NaN
NaN	NaN	19,7994	19,9611	NaN	NaN	NaN	NaN	NaN
NaN	NaN	NaN	NaN	NaN	NaN	NaN	NaN	NaN
NaN	NaN	NaN	NaN	NaN	NaN	NaN	NaN	NaN
NaN	NaN	NaN	NaN	NaN	NaN	NaN	NaN	NaN
NaN	NaN	NaN	NaN	NaN	NaN	NaN	NaN	NaN
22,2457	22,3187	20,3001	20,2659	21,5524	21,6205	22,2512	22,1593	21,4163
NaN	NaN	NaN	NaN	NaN	NaN	NaN	NaN	NaN
NaN	NaN	18,9138	18,9737	NaN	NaN	NaN	NaN	NaN
NaN	NaN	NaN	NaN	NaN	NaN	NaN	NaN	NaN
NaN	NaN	NaN	NaN	NaN	NaN	NaN	NaN	NaN
NaN	NaN	20,259	20,7934	NaN	NaN	NaN	NaN	NaN
NaN	NaN	21,0653	20,7965	NaN	NaN	NaN	NaN	NaN
NaN	NaN	23,1418	23,1767	NaN	NaN	NaN	21,6655	21,39

NaN	NaN	NaN	NaN	NaN	NaN	NaN	NaN	NaN
NaN	NaN	NaN	NaN	NaN	NaN	NaN	NaN	NaN
NaN	NaN	19,7243	19,8926	NaN	NaN	NaN	NaN	NaN
NaN	NaN	19,6547	19,6334	NaN	NaN	NaN	NaN	NaN
24,0678	24,6428	21,0457	NaN	NaN	23,4558	23,0244	22,8775	NaN
NaN	NaN	21,8268	20,6676	NaN	NaN	NaN	NaN	NaN
NaN	NaN	NaN	NaN	NaN	NaN	21,1257	21,2996	NaN
NaN	NaN	NaN	NaN	NaN	NaN	NaN	NaN	NaN
NaN	NaN	NaN	NaN	NaN	NaN	NaN	NaN	NaN
NaN	NaN	NaN	NaN	NaN	NaN	NaN	NaN	NaN
NaN	NaN	20,4178	20,08	NaN	NaN	NaN	NaN	NaN
NaN	NaN	NaN	NaN	NaN	NaN	NaN	NaN	NaN
NaN	NaN	19,9448	NaN	NaN	NaN	NaN	NaN	NaN
NaN	NaN	NaN	NaN	NaN	NaN	NaN	NaN	NaN
NaN	NaN	NaN	17,4505	NaN	NaN	NaN	NaN	NaN
NaN	NaN	NaN	NaN	NaN	NaN	NaN	NaN	NaN
NaN	NaN	19,4665	19,0272	NaN	NaN	NaN	NaN	NaN
23,7021	23,3545	22,2014	22,0366	23,1753	23,0526	NaN	NaN	22,6909
NaN	NaN	NaN	NaN	NaN	NaN	NaN	NaN	NaN
NaN	NaN	NaN	NaN	NaN	NaN	NaN	NaN	NaN
NaN	NaN	NaN	NaN	NaN	NaN	NaN	NaN	NaN
NaN	NaN	NaN	NaN	NaN	NaN	NaN	NaN	NaN
NaN	NaN	NaN	18,4467	NaN	NaN	NaN	NaN	NaN
NaN	NaN	NaN	NaN	NaN	NaN	NaN	NaN	NaN
NaN	NaN	NaN	20,9131	NaN	NaN	NaN	NaN	NaN
NaN	NaN	NaN	NaN	NaN	NaN	NaN	NaN	NaN
NaN	NaN	NaN	NaN	NaN	NaN	NaN	NaN	NaN
NaN	NaN	21,2588	21,0727	NaN	NaN	NaN	NaN	20,0009
NaN	NaN	19,7305	19,8478	NaN	NaN	NaN	NaN	NaN
NaN	NaN	NaN	NaN	NaN	NaN	NaN	NaN	NaN
NaN	NaN	NaN	NaN	NaN	NaN	NaN	NaN	NaN
NaN	NaN	NaN	NaN	NaN	NaN	NaN	NaN	NaN
21,096	20,9124	20,9465	20,9219	NaN	NaN	20,6226	20,6508	20,4417
NaN	NaN	20,8895	20,8115	NaN	NaN	NaN	NaN	NaN
NaN	NaN	NaN	NaN	NaN	NaN	19,4974	19,4196	20,6594
NaN	NaN	NaN	NaN	NaN	NaN	NaN	NaN	NaN
23,147	22,9643	NaN	NaN	22,8438	22,2184	23,1661	22,6915	21,2521
21,6047	21,296	NaN	NaN	NaN	NaN	NaN	NaN	NaN
NaN	NaN	20,6822	21,0314	NaN	NaN	NaN	NaN	NaN
NaN	NaN	NaN	NaN	NaN	NaN	NaN	NaN	20,1183
NaN	NaN	NaN	NaN	NaN	NaN	NaN	NaN	NaN
NaN	NaN	NaN	NaN	NaN	NaN	NaN	NaN	NaN
NaN	NaN	NaN	NaN	NaN	NaN	NaN	NaN	NaN
NaN	NaN	19,1048	NaN	NaN	NaN	NaN	NaN	NaN
NaN	NaN	NaN	NaN	NaN	NaN	NaN	NaN	NaN
NaN	NaN	NaN	NaN	NaN	NaN	NaN	NaN	NaN
25,6632	25,4698	23,5422	23,5463	24,0952	24,062	23,6388	23,5156	23,3669
22,2118	22,276	21,0719	21,0784	21,6007	21,4489	20,7585	20,8568	20,5394
NaN	NaN	NaN	NaN	NaN	NaN	NaN	NaN	NaN
22,7141	22,8526	21,7167	21,9568	23,0618	23,0163	21,8184	21,685	21,1664
23,1311	20,6105	19,293	19,1263	NaN	NaN	NaN	NaN	21,2206
NaN	NaN	NaN	NaN	NaN	NaN	NaN	NaN	NaN

NaN	NaN	NaN	NaN	NaN	NaN	NaN	NaN	NaN
NaN	NaN	NaN	20,1993	NaN	NaN	NaN	NaN	NaN
NaN	NaN	20,6927	NaN	NaN	NaN	NaN	NaN	NaN
NaN	NaN	20,389	20,4506	NaN	NaN	NaN	NaN	NaN
NaN	NaN	19,458	NaN	NaN	NaN	NaN	NaN	NaN
26,8181	26,96	25,4355	25,4337	26,5537	26,72	26,3086	26,2335	25,8547
NaN	NaN	20,7832	20,6954	NaN	NaN	NaN	NaN	NaN
NaN	NaN	NaN	NaN	NaN	NaN	NaN	NaN	NaN
NaN	NaN	NaN	NaN	NaN	NaN	NaN	NaN	NaN
NaN	NaN	NaN	NaN	NaN	NaN	NaN	NaN	NaN
NaN	NaN	NaN	NaN	NaN	NaN	NaN	NaN	NaN
NaN	NaN	19,6683	19,2957	NaN	NaN	NaN	NaN	NaN
NaN	NaN	NaN	19,6535	NaN	NaN	NaN	NaN	NaN
23,0867	23,1216	NaN	NaN	NaN	NaN	NaN	NaN	20,5562
24,6243	24,6889	23,8555	23,8389	23,1238	23,174	23,1504	23,2165	23,512
22,0506	21,1345	NaN	21,3177	NaN	NaN	NaN	NaN	NaN
NaN	21,5775	22,7715	22,7662	21,2448	21,4812	20,9826	20,9727	20,9146
NaN	NaN	NaN	NaN	NaN	NaN	NaN	NaN	NaN
NaN	NaN	NaN	22,7722	NaN	NaN	NaN	NaN	NaN
NaN	21,821	NaN	NaN	NaN	NaN	NaN	NaN	NaN
NaN	NaN	NaN	NaN	NaN	NaN	NaN	NaN	NaN
NaN	23,0616	NaN	NaN	NaN	NaN	NaN	NaN	NaN
21,7639	22,0267	NaN	NaN	NaN	NaN	NaN	NaN	NaN
23,5934	23,7592	NaN	NaN	NaN	NaN	NaN	NaN	21,7022
NaN	NaN	NaN	NaN	NaN	NaN	NaN	NaN	NaN
NaN	NaN	NaN	NaN	NaN	NaN	NaN	NaN	NaN
NaN	20,7739	NaN	NaN	NaN	NaN	NaN	NaN	NaN
21,2388	21,2565	19,9969	NaN	20,7905	NaN	NaN	NaN	NaN
NaN	NaN	19,5406	19,7895	NaN	NaN	20,2218	20,2344	22,3708
NaN	NaN	NaN	NaN	NaN	NaN	NaN	NaN	NaN
NaN	NaN	NaN	NaN	NaN	NaN	NaN	NaN	NaN
NaN	NaN	20,4592	NaN	NaN	NaN	NaN	NaN	NaN
NaN	NaN	NaN	NaN	NaN	NaN	NaN	NaN	NaN
NaN	NaN	NaN	NaN	NaN	NaN	NaN	NaN	NaN
NaN	NaN	NaN	NaN	NaN	NaN	NaN	NaN	NaN
NaN	NaN	18,99	NaN	NaN	NaN	NaN	NaN	NaN
NaN	NaN	NaN	NaN	NaN	NaN	NaN	NaN	NaN
NaN	NaN	NaN	NaN	NaN	NaN	NaN	NaN	NaN
NaN	NaN	NaN	NaN	NaN	NaN	NaN	NaN	NaN
NaN	NaN	NaN	NaN	NaN	NaN	NaN	NaN	NaN
NaN	NaN	NaN	NaN	NaN	NaN	NaN	NaN	NaN
NaN	NaN	NaN	NaN	NaN	NaN	NaN	NaN	NaN
NaN	NaN	NaN	NaN	NaN	NaN	NaN	NaN	NaN
NaN	NaN	NaN	NaN	NaN	NaN	NaN	NaN	NaN
NaN	NaN	NaN	NaN	NaN	NaN	NaN	NaN	NaN
NaN	NaN	NaN	20,3347	NaN	NaN	NaN	NaN	NaN
NaN	NaN	NaN	NaN	NaN	NaN	NaN	NaN	NaN
NaN	NaN	21,6453	21,3988	NaN	NaN	NaN	NaN	NaN
NaN	NaN	NaN	NaN	NaN	NaN	NaN	NaN	NaN
20,2274	20,1756	NaN	19,8037	NaN	NaN	NaN	NaN	NaN
NaN	NaN	NaN	NaN	NaN	NaN	NaN	NaN	NaN
NaN	NaN	NaN	NaN	NaN	NaN	NaN	NaN	NaN

NaN	NaN	NaN	NaN	NaN	NaN	NaN	NaN	NaN
NaN	NaN	NaN	NaN	NaN	NaN	NaN	NaN	NaN
NaN	NaN	21,9322	22,0975	NaN	NaN	NaN	NaN	NaN
NaN	NaN	NaN	NaN	NaN	NaN	NaN	NaN	NaN
NaN	NaN	NaN	NaN	NaN	NaN	NaN	NaN	NaN
NaN	NaN	19,5243	NaN	NaN	NaN	NaN	NaN	NaN
NaN	NaN	NaN	19,62	NaN	NaN	NaN	NaN	NaN
NaN	NaN	NaN	20,284	NaN	NaN	NaN	NaN	NaN
NaN	NaN	19,3397	19,1479	NaN	NaN	NaN	NaN	NaN
NaN	NaN	NaN	NaN	NaN	NaN	NaN	NaN	NaN
NaN	NaN	NaN	NaN	NaN	NaN	NaN	NaN	NaN
NaN	NaN	NaN	NaN	NaN	NaN	NaN	NaN	NaN
NaN	NaN	21,314	21,4534	NaN	NaN	NaN	NaN	NaN
NaN	NaN	NaN	17,5307	NaN	NaN	NaN	NaN	NaN
NaN	NaN	NaN	NaN	NaN	NaN	NaN	NaN	NaN
NaN	NaN	NaN	NaN	NaN	NaN	NaN	NaN	NaN
NaN	NaN	NaN	NaN	NaN	NaN	NaN	NaN	NaN
NaN	NaN	NaN	19,6922	NaN	NaN	NaN	NaN	NaN
NaN	NaN	NaN	19,9073	NaN	NaN	NaN	NaN	NaN
NaN	NaN	20,3658	20,0979	NaN	NaN	NaN	NaN	19,6726
NaN	NaN	NaN	NaN	NaN	NaN	NaN	NaN	NaN
22,1852	22,0824	NaN	NaN	NaN	NaN	NaN	NaN	NaN
NaN	NaN	NaN	NaN	NaN	NaN	NaN	NaN	NaN
NaN	NaN	NaN	NaN	NaN	NaN	NaN	NaN	NaN
NaN	NaN	NaN	NaN	NaN	NaN	NaN	NaN	NaN
NaN	NaN	NaN	NaN	NaN	NaN	NaN	NaN	NaN
NaN	NaN	23,1786	NaN	25,7032	25,83	NaN	NaN	23,5095
NaN	NaN	NaN	NaN	NaN	NaN	NaN	NaN	NaN
21,7013	NaN	23,0045	23,1572	NaN	NaN	NaN	NaN	21,0824
NaN	NaN	19,4963	19,4611	NaN	NaN	NaN	20,9	NaN
NaN	NaN	NaN	NaN	NaN	NaN	NaN	NaN	NaN
NaN	NaN	NaN	NaN	NaN	NaN	NaN	NaN	NaN
22,5594	22,5137	NaN	NaN	NaN	NaN	NaN	NaN	NaN
NaN	NaN	NaN	19,5224	NaN	NaN	NaN	NaN	NaN
NaN	NaN	NaN	NaN	NaN	NaN	NaN	NaN	NaN
NaN	NaN	NaN	NaN	NaN	NaN	NaN	NaN	NaN
NaN	NaN	21,8053	NaN	22,5727	22,4821	NaN	NaN	NaN
NaN	NaN	NaN	NaN	NaN	NaN	NaN	NaN	NaN
NaN	NaN	NaN	NaN	NaN	NaN	NaN	NaN	NaN
NaN	NaN	NaN	NaN	NaN	NaN	NaN	NaN	NaN
23,2717	23,304	NaN	NaN	25,586	25,5444	23,7726	23,9704	20,1587
NaN	NaN	19,9813	NaN	NaN	NaN	NaN	NaN	NaN
22,6923	22,9818	NaN	NaN	NaN	NaN	NaN	NaN	NaN
NaN	NaN	20,5841	20,5744	NaN	NaN	NaN	NaN	NaN
NaN	NaN	20,5188	20,319	NaN	NaN	NaN	NaN	20,1043
NaN	NaN	19,7334	NaN	NaN	NaN	NaN	NaN	NaN
NaN	NaN	NaN	NaN	NaN	NaN	NaN	NaN	NaN
NaN	NaN	NaN	NaN	NaN	NaN	NaN	NaN	NaN
NaN	NaN	19,9166	19,533	NaN	NaN	NaN	NaN	NaN
NaN	NaN	NaN	NaN	NaN	NaN	NaN	NaN	NaN
NaN	NaN	19,0715	19,3143	NaN	NaN	NaN	NaN	NaN
NaN	NaN	NaN	20,2265	NaN	NaN	NaN	NaN	NaN

NaN	NaN	20,7792	NaN	NaN	NaN	NaN	NaN	20,7103
NaN	NaN	NaN	NaN	NaN	NaN	NaN	NaN	NaN
NaN	NaN	26,4838	26,5789	NaN	NaN	NaN	NaN	NaN
22,5846	23,2112	28,9249	28,89	32,3253	32,2495	28,6086	28,5539	30,5886
NaN	NaN	20,2035	20,2126	20,5441	NaN	NaN	NaN	NaN
21,1381	20,9913	NaN	NaN	NaN	NaN	NaN	NaN	22,795
NaN	NaN	NaN	NaN	NaN	NaN	NaN	NaN	NaN
NaN	NaN	19,3765	19,5901	NaN	NaN	NaN	NaN	NaN
NaN	NaN	NaN	NaN	NaN	NaN	NaN	NaN	NaN
NaN	NaN	NaN	NaN	NaN	NaN	NaN	NaN	NaN
NaN	NaN	NaN	19,5086	NaN	NaN	NaN	NaN	NaN
NaN	NaN	19,1564	NaN	NaN	NaN	NaN	20,3619	20,0053
25,3222	25,567	23,2626	23,3391	24,2654	24,3064	24,0742	24,0713	23,7452
21,4884	22,3287	19,65	NaN	NaN	20,5606	20,4795	20,4398	NaN
NaN	NaN	NaN	NaN	NaN	NaN	NaN	NaN	NaN
NaN	NaN	NaN	NaN	28,9603	28,882	25,9204	26,1159	29,5455
NaN	NaN	25,5522	25,6506	NaN	NaN	22,9641	23,2628	NaN
NaN	NaN	NaN	NaN	NaN	NaN	NaN	NaN	NaN
NaN	NaN	18,6475	18,6308	NaN	NaN	NaN	NaN	NaN
NaN	20,4902	22,1369	22,0759	NaN	NaN	20,5221	20,628	20,2093
NaN	NaN	NaN	NaN	NaN	NaN	NaN	NaN	NaN
NaN	NaN	NaN	NaN	20,9381	20,9489	NaN	NaN	NaN
NaN	NaN	NaN	NaN	NaN	NaN	NaN	NaN	NaN
NaN	NaN	NaN	NaN	NaN	NaN	NaN	NaN	NaN
NaN	NaN	NaN	NaN	NaN	NaN	NaN	NaN	NaN
NaN	NaN	NaN	NaN	NaN	NaN	21,3527	NaN	NaN
NaN	NaN	NaN	NaN	NaN	NaN	NaN	NaN	NaN
NaN	NaN	NaN	NaN	NaN	NaN	NaN	NaN	NaN
NaN	NaN	NaN	18,2147	NaN	NaN	NaN	NaN	NaN
NaN	NaN	21,0807	21,2443	NaN	NaN	NaN	NaN	NaN
NaN	NaN	NaN	21,0898	NaN	NaN	NaN	NaN	NaN
NaN	NaN	NaN	NaN	NaN	NaN	NaN	NaN	NaN
25,912	25,9499	24,3266	24,2741	26,0123	26,0903	25,6572	25,622	25,6335
NaN	NaN	NaN	NaN	NaN	NaN	NaN	NaN	NaN
NaN	NaN	23,2977	23,2995	NaN	NaN	NaN	NaN	21,4731
NaN	NaN	NaN	NaN	NaN	NaN	NaN	NaN	NaN
NaN	NaN	NaN	28,6993	NaN	NaN	NaN	NaN	NaN
NaN	NaN	NaN	NaN	NaN	NaN	NaN	NaN	NaN
21,5014	21,0497	NaN	NaN	NaN	NaN	NaN	NaN	NaN
20,8641	20,6588	NaN	NaN	NaN	NaN	NaN	NaN	NaN
NaN	NaN	NaN	NaN	NaN	NaN	NaN	NaN	NaN
24,8212	24,6333	21,861	21,6801	23,3362	22,4425	22,1734	22,7566	22,3422
NaN	NaN	26,3045	26,2672	NaN	NaN	NaN	NaN	25,2228
NaN	NaN	NaN	NaN	NaN	NaN	NaN	NaN	NaN
NaN	NaN	19,3811	19,5036	NaN	NaN	NaN	NaN	NaN
NaN	NaN	NaN	NaN	NaN	NaN	NaN	NaN	NaN
26,0156	26,1877	NaN	24,4838	NaN	NaN	25,6932	25,6408	24,8102
NaN	NaN	NaN	NaN	NaN	NaN	NaN	NaN	NaN
NaN	NaN	NaN	NaN	NaN	NaN	NaN	NaN	NaN
24,1438	24,3129	NaN	NaN	21,5333	NaN	NaN	NaN	NaN
NaN	NaN	NaN	NaN	NaN	NaN	NaN	NaN	NaN
NaN	NaN	NaN	NaN	NaN	NaN	NaN	NaN	NaN

NaN	NaN	NaN	NaN	NaN	NaN	NaN	NaN	NaN
21,9777	22,0348	20,48	20,4633	22,0652	NaN	23,6638	23,8937	NaN
21,6763	21,62	22,2218	22,2854	23,0939	22,6725	21,4733	21,9483	21,3923
21,0294	20,9511	NaN	NaN	NaN	NaN	21,3808	21,3824	21,6643
NaN	NaN	NaN	NaN	NaN	NaN	NaN	NaN	NaN
NaN	NaN	21,8986	21,8771	NaN	NaN	NaN	NaN	NaN
NaN	NaN	NaN	19,7409	NaN	19,5249	NaN	22,9707	25,3401
NaN	NaN	23,3221	23,4481	21,5961	22,1282	21,6479	21,4085	NaN
NaN	NaN	NaN	NaN	NaN	NaN	NaN	NaN	NaN
NaN	NaN	20,313	19,878	NaN	NaN	NaN	NaN	NaN
NaN	NaN	NaN	NaN	NaN	NaN	NaN	NaN	NaN
26,861	26,939	25,6406	25,7016	26,4474	26,453	25,7071	25,7659	25,5183
NaN	NaN	NaN	NaN	NaN	NaN	NaN	NaN	NaN
NaN	NaN	NaN	21,8148	22,9064	NaN	NaN	NaN	22,6557
NaN	NaN	NaN	NaN	NaN	NaN	NaN	NaN	NaN
NaN	NaN	21,647	21,7848	NaN	NaN	NaN	NaN	NaN
23,4371	23,2971	NaN	NaN	22,7883	22,8352	22,6309	22,682	24,1061

LFQ intensi	LFQ intensi	LFQ intensi	LFQ intensi	LFQ intensi	LFQ intensi	LFQ intensi	LFQ intensi	LFQ intensi
33,2006	32,9135	32,9089	32,2506	32,2263	31,5489	31,5501	33,0077	32,9861
33,9226	33,0735	33,0669	33,4079	33,419	34,0825	34,0837	34,1996	34,1787
29,102	25,7909	25,9465	27,3665	27,4509	24,9506	25,0743	25,3188	25,2745
18,784	27,6709	27,7925	21,272	21,1925	17,2266	NaN	17,0169	17,428
27,4747	26,8148	26,8187	26,7227	26,7264	27,8957	27,9214	27,9141	27,8937
25,6633	26,1272	25,9558	25,5741	25,4963	25,4062	25,4434	25,8861	25,9148
20,7956	26,7089	26,6724	23,0053	22,8967	22,0051	21,1371	NaN	NaN
28,2595	27,8807	27,9134	27,2619	27,2388	28,211	28,1929	27,9864	27,9136
29,6303	29,1749	29,2051	28,7441	28,695	29,8291	29,8673	29,5365	29,6899
29,8845	28,9693	29,0217	29,1185	29,1129	29,0623	29,0757	28,5225	28,5438
16,8417	26,5597	26,491	19,9632	18,3952	15,9013	NaN	NaN	NaN
34,1327	34,8415	34,8729	34,9987	34,9684	34,4169	34,3995	35,6707	35,7251
32,145	31,0141	31,0117	33,0165	32,9316	32,5038	32,5246	31,4684	31,436
29,7523	30,3832	30,3771	30,0802	30,0291	28,4044	28,398	30,1273	30,1173
25,3116	26,2427	26,3189	25,9857	26,0568	25,5147	25,1938	25,76	25,796
26,4897	27,2099	27,2163	27,5492	27,5427	28,4843	28,4875	27,1957	27,0421
27,2262	26,4029	26,4056	27,4963	27,4443	27,6007	27,8053	28,0295	27,9641
26,9593	32,9334	32,8681	29,2801	29,1935	25,7302	25,6775	26,1465	26,0005
26,7672	30,8305	30,785	27,9691	28,0005	25,9497	25,9113	26,4564	26,4779
23,5525	23,6831	23,629	23,0493	22,9266	22,9052	22,746	23,7926	23,1453
27,519	32,6946	32,7292	29,5118	29,4502	26,271	26,1617	26,636	26,6542
25,1482	24,6566	24,651	25,4794	25,4989	25,438	25,4871	25,4912	25,4024
28,4166	27,5767	27,5869	28,0066	27,9746	27,7426	27,6875	28,5995	28,612
25,9043	25,1718	25,1424	26,2808	26,3014	26,1914	26,2084	25,9062	25,9518
28,2372	28,4265	28,3901	28,4933	28,4286	26,8644	26,9952	28,6618	28,685
25,5582	25,8479	25,8316	25,405	25,3214	25,9602	25,8971	25,416	25,4336
27,6325	27,5911	27,5868	27,9075	27,8519	26,3508	26,3565	28,0453	28,1058
23,4556	22,9147	23,2768	23,372	23,3602	23,1386	23,2819	23,3981	23,5513
27,494	27,2962	27,2188	26,8994	26,8286	25,0592	25,1465	27,332	27,3119
26,2215	28,5929	28,6048	27,1753	27,2036	25,3438	25,3383	25,774	25,8764
25,7898	25,6353	25,6511	26,7797	26,7604	26,478	26,5138	26,1548	26,1953
26,7081	28,2456	28,1814	27,327	27,32	28,2666	28,2581	27,3171	27,5958
26,3641	26,6678	26,5744	26,5513	26,5925	27,2061	27,2219	26,8865	26,8872
24,4499	27,2558	27,2959	26,0421	25,9105	25,2052	25,1542	24,8908	24,9774
23,4022	24,7557	24,7205	23,4142	23,6043	23,0676	23,3022	22,5232	22,65
26,4314	26,6211	26,6157	26,844	26,8235	27,3122	27,3041	26,5286	26,6726
27,9931	27,8965	27,8704	28,545	28,4957	29,0567	29,0842	28,121	28,1394
26,2177	25,5325	25,4868	26,705	26,7112	26,3193	26,3108	26,0445	25,8992
26,8471	26,3671	26,3448	25,7137	25,7728	26,5382	26,5151	25,9656	25,8779
26,5956	26,2109	26,1726	27,0427	26,9156	27,2194	27,1785	26,7886	26,9187
29,0657	28,4511	28,458	29,3527	29,3029	27,674	27,6785	29,4316	29,4444
25,4328	26,3363	26,2668	26,3977	26,4404	26,7864	26,7144	26,1711	26,0267
25,9428	25,9298	25,936	26,3713	26,439	26,5278	26,499	25,8664	25,864
25,8744	25,4905	25,4521	26,181	26,2289	26,5666	26,4444	26,3129	26,2906
24,5117	24,7297	24,689	25,1217	25,0668	25,4107	25,4782	25,0325	24,928
26,6129	26,189	26,2624	27,1352	27,0645	26,9365	26,9554	26,8545	26,858
24,5722	24,9695	25,0015	25,2884	25,2867	25,4502	25,4743	25,0728	24,8291
23,6476	23,472	23,5621	23,2219	22,8592	22,6141	22,6221	21,7233	22,8648
30,7304	28,8243	28,8065	29,9535	29,8944	29,9468	29,9935	29,6226	29,6469
19,7145	26,7206	26,7677	22,0101	22,19	NaN	NaN	20,0539	17,298
24,5226	23,982	24,0227	24,3218	24,2842	24,3745	24,3741	24,4811	24,5509

26,2673	25,8357	25,8515	26,5053	26,5503	26,4463	26,4138	26,259	26,3084
32,7001	31,4649	31,4635	33,6702	33,6217	33,3396	33,4065	32,0403	31,9665
21,1581	18,9031	20,7261	21,7796	21,8015	28,1532	28,2487	22,3004	22,4232
25,1425	24,29	24,3337	25,6842	25,6419	25,8269	25,8695	26,4164	26,3524
NaN	25,2945	25,7162	19,2453	18,2033	NaN	NaN	NaN	NaN
25,1709	25,3452	25,3026	25,7343	25,8228	26,0742	26,0492	25,3462	25,5156
23,1479	23,8524	23,9224	24,1356	24,0992	24,8279	24,67	23,3195	23,421
26,2318	26,2818	26,1984	25,9951	25,9037	27,0042	26,9795	26,8185	26,7128
27,468	30,8967	30,9496	27,7958	27,8284	26,5946	26,5894	25,9121	25,8533
23,781	25,0337	25,0714	25,8898	25,7766	23,4689	23,656	23,7185	23,6744
24,4095	25,0984	25,1283	23,787	23,9371	23,4777	23,6067	23,1474	23,2859
23,1437	24,0204	24,0303	25,2419	25,2359	24,9144	24,8565	23,4841	23,7427
27,5304	27,1793	27,2602	25,679	25,694	26,7075	26,6668	27,6707	27,6857
24,004	24,1149	23,9752	24,0115	23,8165	24,3483	24,4574	24,3548	24,4315
24,1309	24,1234	24,0322	24,2907	24,379	24,557	24,5213	24,2372	24,415
24,8923	28,5114	28,488	25,8675	25,8457	23,8995	23,9011	24,982	25,2743
26,4271	26,6503	26,7161	26,7562	26,7679	27,759	27,8292	26,8043	26,7617
22,4228	25,2445	25,008	23,0916	22,8638	21,6844	22,1104	20,8953	NaN
30,1799	29,979	29,9749	30,4711	30,5019	30,4096	30,4882	29,9254	29,9879
22,7423	23,2177	23,0236	23,6447	23,6751	24,252	24,4248	23,0655	22,7345
22,7107	23,0086	22,647	22,3782	22,7152	22,8391	22,6169	23,2176	22,9548
23,9222	23,9323	23,7011	24,3753	24,5776	23,8273	23,891	24,567	24,4763
25,0206	24,5857	24,5867	24,9742	24,9362	24,9571	25,1281	25,1297	25,1931
29,2856	27,8538	27,8728	28,8543	28,852	28,7482	28,818	28,8025	28,7321
24,2035	24,4191	24,2987	23,8796	24,008	24,217	24,3572	24,9985	25,0423
24,9674	28,7533	28,6592	26,9964	27,04	27,7474	27,772	27,2595	27,174
23,5983	24,6571	24,6163	24,2916	24,3047	24,2569	24,2118	23,9072	23,7167
24,0205	23,9012	23,9423	24,4269	24,4267	24,4694	24,6206	24,355	24,2925
26,7631	26,6569	26,5865	27,3138	27,1905	27,7138	27,6651	26,8831	26,91
26,1175	25,9156	25,9837	25,4	25,5017	25,8565	25,8797	25,6877	25,5622
26,472	26,5673	26,5948	27,1334	27,1196	27,3123	27,2999	26,5472	26,5183
16,9849	27,9344	27,7792	22,6559	23,4418	NaN	16,4872	17,1871	15,8524
23,8251	23,2832	23,2601	22,8175	22,7051	23,2698	23,1056	23,5312	23,6639
27,0294	26,7025	26,5882	25,9421	25,8875	26,8272	26,7624	26,1497	26,3145
24,7419	23,6801	23,6311	23,7655	24,1206	23,9802	24,0095	23,5718	23,7952
22,3781	21,357	21,3167	NaN	21,6138	22,7836	22,8098	21,7691	21,7673
27,9326	27,5131	27,4871	27,7533	27,7237	28,7974	28,7872	27,8143	27,8088
24,7265	24,6137	24,4331	25,6009	25,5685	25,4172	25,2503	24,7885	24,8668
28,0681	28,1952	28,1581	27,5486	27,514	23,3918	23,2845	28,3304	28,3654
24,0562	27,8006	27,8458	24,4054	24,5357	22,5882	22,6179	19,7389	19,2329
29,4804	28,468	28,4067	29,0532	29,0803	29,9646	29,9757	29,3768	29,3516
NaN	24,1908	23,77	21,3506	21,1695	NaN	NaN	NaN	NaN
25,7552	24,5362	24,4902	25,1685	25,1555	25,7678	25,664	25,1388	25,1962
22,9242	20,5935	21,0622	23,019	23,1248	23,4713	23,414	23,1959	23,0144
26,8747	25,4534	25,4659	25,7823	25,8421	26,0492	25,9627	26,5033	26,555
25,5173	25,3714	25,3371	26,1661	26,1794	26,2062	26,2478	25,5371	25,4521
28,1015	26,7648	26,6242	26,5258	26,5631	28,1002	28,0579	27,341	27,2832
26,7317	25,8024	25,8577	25,9522	25,9273	26,8606	26,9695	26,8628	26,8488
26,8132	26,7985	26,87	27,1776	27,1342	28,0734	28,0334	27,1408	27,0929
31,0854	30,0129	30,082	32,0748	32,1043	32,0172	32,1437	30,9972	30,9028
33,47	31,2771	31,3009	32,3094	32,3526	33,5309	33,5203	31,7852	31,8191
25,8907	27,4845	27,528	26,9086	27,0055	27,2728	27,2628	26,5704	26,6539

22,7518	22,4177	22,8743	23,3295	23,2518	23,8246	23,7471	23,8653	23,5355
22,7012	25,6855	25,6344	24,8696	24,9397	NaN	NaN	NaN	NaN
22,6045	20,458	20,4886	25,2057	25,05	NaN	NaN	23,473	23,7675
23,3467	23,9465	23,8222	23,4672	23,5135	24,0015	24,0683	23,4942	23,3801
24,7033	25,4058	25,245	24,7372	24,9491	25,8105	25,9664	24,5107	24,312
25,4429	24,8967	24,9118	25,4213	25,4272	25,3807	25,3033	25,3471	25,1925
24,0165	24,1134	23,9748	23,4925	23,5754	24,2008	24,1931	23,5846	23,6348
26,1591	24,2101	24,0901	25,9431	25,992	25,0247	24,9217	25,1278	25,1969
25,5542	25,3028	25,327	26,2713	26,332	26,549	26,5517	26,1276	26,0717
28,1582	27,7709	27,7755	28,8011	28,8442	29,2418	29,2245	27,2983	27,3813
NaN	NaN	NaN	NaN	NaN	24,9672	24,9044	NaN	NaN
24,9696	24,0292	24,0606	24,1305	24,0781	22,5221	23,0248	24,1338	24,2458
26,0565	25,8622	25,8006	26,7843	26,7434	27,193	27,2205	26,2302	26,247
24,7829	24,607	24,5012	25,3775	25,2791	25,595	25,6146	24,8274	24,8639
24,0419	24,2498	24,2229	24,1777	24,0666	23,8994	23,9669	24,1485	24,3816
26,9198	25,8343	25,7956	26,9539	26,9283	27,1179	27,0199	26,6181	26,6875
22,2652	23,2037	23,5337	22,7321	22,5504	21,9342	22,1711	22,1411	22,194
21,5224	23,8831	23,7232	23,1154	22,9983	24,0089	23,938	22,4487	20,9456
23,1959	23,3781	23,2864	22,2373	22,1629	22,6037	22,3235	21,5664	22,9567
22,6579	22,6584	22,7181	22,5434	22,1444	20,4468	20,321	20,7479	20,7975
22,3118	23,6217	23,6686	22,4538	22,7084	23,2897	23,1024	22,7915	23,0345
24,5353	23,8613	23,6821	24,6527	24,8478	25,2465	25,3082	24,3661	24,2653
NaN	24,4089	23,7631	18,4009	19,3572	NaN	NaN	NaN	NaN
26,7179	26,7777	26,6898	26,01	26,0321	28,0388	27,9751	27,2827	27,2783
22,8117	NaN	NaN	23,7625	23,6391	22,5114	22,8105	NaN	NaN
23,8468	25,2642	25,1713	24,6972	24,5961	25,2387	25,2483	23,9035	24,1595
26,6085	25,7886	25,6977	26,4165	26,4362	26,6558	26,7367	26,6962	26,8445
NaN	NaN	NaN	NaN	NaN	NaN	NaN	NaN	NaN
20,7284	23,2213	23,2068	22,3711	22,2141	23,3757	23,5725	22,5284	21,5197
24,8943	26,5011	26,7178	25,3073	25,2726	23,7692	23,7939	24,4991	24,1532
23,6202	23,8986	23,8222	23,9465	23,9011	24,3877	24,5133	22,8455	23,3022
29,9686	28,8129	28,732	30,8719	30,8592	30,816	30,7818	29,4494	29,4319
25,6137	25,2302	25,2366	24,4445	24,4748	25,322	25,3231	24,5589	24,4403
25,2221	25,0262	25,0618	25,2978	25,3082	25,604	25,3618	25,2447	25,4227
23,9774	24,906	24,832	24,1185	24,1079	25,587	25,4794	24,781	24,6955
23,007	24,714	24,729	23,5591	23,4558	22,805	22,9591	21,8718	21,6856
23,8969	25,4775	25,3962	24,3543	24,4451	25,3166	25,3269	24,7216	24,7169
30,4247	28,0224	27,9545	30,5825	30,5618	29,4318	29,4031	29,1952	29,208
30,1454	29,6799	29,6553	28,6228	28,5861	28,3798	28,4111	29,7937	29,8068
NaN	24,9304	25,0948	21,1782	20,7234	NaN	NaN	NaN	NaN
25,7808	24,5156	24,4906	23,82	23,9057	23,5807	23,4945	24,9686	24,9686
25,3363	26,5527	26,5322	25,6029	25,6788	29,3572	29,3678	33,7934	33,876
25,133	24,6419	24,6198	24,7059	24,4963	25,0671	25,1444	25,3879	25,4345
23,4078	23,5478	23,6681	23,3329	23,4653	23,4997	23,2198	23,6537	23,6049
23,9576	23,3019	23,372	23,7222	23,6642	24,3608	24,3717	24,5906	24,6291
32,2851	30,119	29,863	31,1087	31,1851	31,4478	31,84	30,9295	30,9968
24,5157	25,0932	24,8709	22,7587	22,688	23,3357	23,2203	23,2412	22,9829
NaN	20,9618	20,4898	23,0956	23,0562	21,0158	20,1126	21,1796	21,7679
23,2282	24,098	24,0124	24,1887	24,0124	23,1449	23,4983	23,7339	23,233
21,5104	21,6211	21,6886	23,4749	23,3126	22,6871	22,5988	NaN	21,7198
22,163	23,8023	23,826	24,5003	24,5867	24,16	24,1037	24,0972	23,8537
21,8003	23,0955	23,1567	23,2723	23,0539	22,4444	22,4886	21,7949	22,0328

21,6549	22,9674	22,6391	22,184	22,2301	22,8187	21,9778	22,0143	22,0707
29,2617	28,4977	28,6196	28,6956	28,5977	24,4415	24,2656	29,191	29,2785
20,1882	23,7527	23,4343	21,3271	20,8046	20,4647	20,2724	NaN	20,5442
23,0896	NaN	NaN	23,1447	23,1185	23,6561	23,6078	NaN	21,6557
22,4147	23,0682	23,0124	23,9	23,923	22,9084	23,3439	21,7048	21,8283
21,4905	21,1285	20,7144	20,5552	21,0329	21,3343	21,0596	20,9865	20,8208
30,7487	29,4017	29,3575	29,5924	29,6221	25,2022	25,429	27,0174	27,3217
26,4261	27,3133	27,3253	27,085	27,0749	27,1234	27,0768	26,6843	26,6594
24,9817	25,0014	24,8567	24,4239	24,4706	24,2862	24,1822	23,37	23,0964
33,517	31,6366	31,6873	32,5706	32,4563	32,4295	32,3035	32,3739	32,4431
23,3915	22,085	22,0276	23,1836	23,1621	22,2083	22,3394	23,1212	22,9433
22,3219	23,3541	23,4433	23,8	24,0107	24,2605	23,9562	22,915	23,0641
30,8989	29,5002	29,4826	28,1994	28,1551	29,2834	29,2621	30,6334	30,6567
23,8244	24,2217	24,3137	24,6051	24,7246	25,0063	25,0716	23,4849	23,5787
23,0147	23,3117	23,2763	22,5418	22,9383	22,7141	23,1447	23,8885	24,0924
29,6488	28,5089	28,5402	30,3972	30,3579	30,8424	30,7329	29,771	29,6816
23,7258	23,6585	23,622	23,6614	23,6866	23,9008	23,883	23,7698	23,5418
22,3467	22,1421	22,4149	22,4538	22,6173	22,6785	23,0146	22,7293	22,8342
21,3261	NaN	NaN	20,7547	NaN	21,4478	21,1669	22,0769	22,0766
NaN	25,0284	24,9712	19,9502	19,9202	NaN	NaN	NaN	NaN
20,1737	21,193	20,8893	21,8678	21,9623	22,5669	22,7536	20,4038	NaN
21,6496	21,89	22,3349	21,603	21,542	22,7002	22,8663	22,0248	21,9047
23,2755	23,1463	23,1389	23,473	23,5803	23,3265	23,362	23,6291	23,6594
NaN	22,1505	21,4447	20,8542	20,4134	22,2132	22,5144	NaN	NaN
24,6181	23,7465	23,5725	23,0619	22,9011	23,5694	23,4545	24,5265	24,7473
24,7616	23,7458	23,9706	24,1018	24,2182	24,0646	24,3182	24,2133	24,4023
23,5289	23,635	23,6977	22,11	22,3775	23,4248	23,5633	24,376	24,1758
29,0179	29,7719	29,6495	28,8336	28,8009	28,3619	28,2626	30,1425	30,0397
23,9614	24,4066	24,4146	24,061	24,1658	24,1231	24,209	24,0923	24,1829
23,6212	23,0383	23,0295	21,9714	22,3126	22,8943	22,8411	22,2773	22,7745
29,8127	28,2879	28,2565	29,7855	29,8053	26,3525	26,3751	27,5648	27,6504
23,4378	23,3918	23,3655	24,0184	23,9776	24,618	24,5238	24,2049	24,3504
27,5116	26,9125	26,9425	27,1713	27,1797	27,8349	27,8686	27,5735	27,4805
26,7057	26,9617	26,9094	27,3115	27,2195	28,3209	28,2335	27,0874	27,154
26,5793	26,0792	26,081	26,742	26,7584	26,2798	26,2603	26,4882	26,5855
23,2591	23,104	23,0717	23,0099	23,0676	23,176	23,1814	22,9901	23,3632
21,6674	22,9899	23,0813	22,7831	22,8504	23,2132	23,2133	20,2865	20,2259
22,4252	22,5326	22,5573	23,0513	22,9382	23,4148	23,1377	22,8259	21,9355
22,4502	22,6677	22,9102	22,2312	22,4973	23,2422	22,9824	21,9544	22,5558
23,1594	22,1713	22,5149	20,7721	20,92	22,754	22,8806	23,7361	23,5918
23,7629	23,0245	23,3184	23,1491	23,2857	24,3439	24,3496	23,5124	23,3087
21,9352	22,8899	22,8259	23,7947	23,7612	23,7459	23,5046	20,9516	20,7153
24,3803	24,1224	24,0078	25,3377	25,3922	26,0425	26,0073	24,6957	24,8231
28,1279	26,8098	26,8191	27,072	27,0844	27,2744	27,153	25,9036	25,9756
21,5155	23,1441	23,1334	22,8968	22,5789	23,3367	23,5658	22,5417	22,5002
NaN	23,9137	23,1585	NaN	NaN	NaN	NaN	NaN	NaN
23,8282	23,8171	23,8615	23,6463	23,3847	24,0833	24,1469	24,0634	24,1202
22,7494	22,1562	22,024	22,5919	22,8881	22,6256	22,7271	22,1046	22,9783
21,6594	24,4794	24,2577	21,6521	21,9034	19,0973	21,0643	NaN	NaN
25,7129	24,589	24,5555	24,4944	24,2768	25,8877	26,0037	25,309	25,3287
23,0582	23,472	23,3905	23,2459	23,4322	24,3611	24,0906	23,4154	23,5015
21,1385	23,1714	23,6344	21,4475	21,2094	20,1964	20,9355	21,8115	21,6041

22,3641	23,5933	23,7589	22,5312	22,5541	22,7949	22,9261	22,3682	22,5335
22,4399	21,444	20,3325	22,0396	22,162	22,5668	23,008	22,5249	22,019
22,0906	22,6517	22,4014	23,2259	23,303	24,0398	24,1031	22,8508	22,9492
22,6711	22,2575	21,7503	22,804	22,7061	22,909	23,0188	22,6173	22,689
22,3199	21,4175	21,2637	21,6248	21,6902	21,8199	22,2762	21,7774	21,53
21,8939	23,1782	23,2689	21,9794	22,3134	22,354	22,398	21,5527	21,7813
21,2248	22,123	23,1024	20,2958	NaN	22,9481	22,2061	NaN	NaN
21,1346	22,0797	21,8646	21,2014	21,6648	21,1551	21,3819	NaN	NaN
33,0295	31,6196	31,5266	31,1411	31,1575	31,0801	31,1709	31,5056	31,5276
27,8229	25,9409	25,8384	26,1438	26,282	26,8371	26,8252	27,2772	27,444
21,86	22,5696	22,3369	21,7842	21,9095	22,3584	22,637	21,3051	21,5979
21,4199	21,6122	21,3045	22,5131	22,6154	21,7382	22,2432	21,8192	22,1621
26,235	26,0954	26,0034	26,7369	26,7985	26,9346	26,9178	26,4832	26,4521
26,1077	28,2428	27,9973	26,3771	26,3369	24,1424	24,0621	25,6665	25,6641
20,935	21,2442	20,7647	21,8576	21,9504	21,6118	21,3357	21,4391	NaN
21,6993	21,8026	21,858	20,4482	NaN	22,4401	22,4796	22,696	22,5875
23,9465	25,087	25,0528	24,2613	24,34	24,1682	24,1259	23,8166	23,9578
22,6321	22,2228	22,3452	22,9637	22,9962	23,598	23,5304	23,51	23,3881
NaN	NaN	NaN	21,0407	20,7969	21,0817	21,2933	20,7021	NaN
NaN	NaN	NaN	NaN	NaN	23,4275	23,5527	27,8289	27,7713
23,8391	26,9796	26,8554	24,4946	24,3404	24,5942	24,7166	NaN	20,6372
30,3095	28,4099	28,2995	30,1706	30,1581	26,3329	26,5133	27,0855	26,9018
23,9702	24,2662	24,0984	24,4626	24,3853	23,6947	23,6475	24,2084	24,1223
24,3019	25,6298	25,6254	25,7236	25,6997	25,5244	25,468	24,5473	24,5006
24,24	24,2211	24,2084	23,9016	23,652	24,6076	24,6466	24,2531	24,4347
17,7127	NaN	NaN	17,9186	NaN	16,6665	NaN	16,994	17,0824
NaN	NaN	NaN	NaN	21,0603	21,261	21,2165	NaN	NaN
23,9602	23,9038	23,9675	24,6139	24,4506	25,629	25,5435	24,6441	24,6272
23,4333	26,1647	26,1711	23,8275	23,8518	23,2345	23,2215	23,0643	22,6696
22,4511	22,5496	22,5865	22,4769	22,7642	22,729	22,9219	22,4872	22,0649
23,1047	23,541	23,5136	23,4863	23,4616	24,3369	24,4449	23,7626	23,8669
22,3551	22,3993	22,5145	21,6483	21,549	22,8761	23,5291	NaN	NaN
22,4036	21,195	21,4452	21,6089	21,5219	22,0045	21,9377	22,1499	22,3134
27,422	25,9119	25,8682	27,9151	27,9972	27,885	28,0392	26,642	26,5392
21,7067	21,2905	21,4129	23,1377	23,4419	21,3004	21,5134	19,5201	19,1555
23,1349	22,5422	23,0023	22,8804	22,8177	22,7978	22,9693	23,2437	23,0545
22,3308	22,8886	22,635	22,5745	22,3706	NaN	NaN	20,9479	NaN
23,2326	22,744	22,4254	22,7646	23,0463	23,418	23,7994	23,2462	22,751
22,0086	22,084	21,8044	23,0386	23,1114	23,5949	22,8234	22,1987	22,4576
22,9474	22,8897	22,6878	22,9695	22,9668	23,0578	23,2393	23,14	23,1066
20,5504	NaN	21,2659	20,1489	19,6916	22,9879	22,7339	20,0188	19,9967
21,8421	21,7276	21,7406	21,8524	21,8748	21,7577	22,1458	21,9014	21,9884
23,0228	22,52	22,6766	24,0705	23,9213	23,6828	23,8403	23,3327	23,3992
22,4004	21,9201	21,6141	20,8106	NaN	21,7957	22,1997	23,009	22,7822
NaN	22,7248	22,7521	19,335	19,4361	NaN	NaN	NaN	NaN
26,4663	26,96	26,8651	27,1369	27,1306	28,2867	28,2798	26,8976	26,7959
NaN	21,7292	21,8753	20,6407	NaN	NaN	NaN	NaN	NaN
22,1925	NaN	20,8312	20,767	20,8424	21,9444	21,697	22,0803	21,8353
31,1128	28,3429	28,308	28,6691	28,6062	30,1032	30,1349	28,3682	28,3365
28,0252	26,5369	26,5573	27,547	27,514	27,3836	27,3726	26,8266	26,8341
21,1822	22,0619	21,8688	22,0882	22,1735	22,5907	22,3904	21,8408	22,2468
22,3919	23,5039	23,6379	22,4159	22,1468	23,5799	23,5968	22,6385	22,597

22,7337	21,7429	21,5607	23,0427	23,4098	23,0408	22,9062	23,0669	23,0556
20,7437	20,8777	21,139	21,4721	21,5666	21,2784	21,7909	NaN	21,034
23,2488	22,2325	21,7501	21,2837	22,2132	22,4271	22,4984	22,7248	22,65
23,154	24,6692	24,7076	22,5065	22,3541	23,9126	23,7087	23,6091	23,8093
29,4902	28,6761	28,5805	31,1039	31,097	31,4767	31,487	29,9457	29,9342
22,1316	21,4195	21,8228	22,2638	22,0303	21,7725	21,6632	22,1511	22,697
NaN	20,3491	20,2347	NaN	NaN	NaN	NaN	21,0472	NaN
22,9546	22,6848	22,7047	23,0264	22,9249	22,7135	23,0118	22,1708	22,1597
NaN	21,4596	NaN	NaN	NaN	NaN	20,7282	NaN	20,7564
22,1023	NaN	20,8539	22,0516	22,0344	21,8455	21,9905	22,759	23,027
22,2791	20,4376	20,9719	20,6348	20,3394	20,525	20,7931	NaN	NaN
21,8679	21,9966	21,834	22,5647	22,5446	22,0579	22,5092	22,0351	21,9488
NaN	NaN	20,2063	20,8445	20,856	21,2059	20,8307	NaN	NaN
21,2566	20,7271	20,6317	21,6426	21,7083	20,8133	20,6736	NaN	NaN
24,3286	25,6176	25,7397	24,7035	24,6549	28,6021	28,5909	33,0514	33,0397
26,9807	26,0601	26,0141	27,1247	26,897	27,1818	27,0621	26,8676	26,973
22,8805	25,3937	25,8368	24,0068	23,825	22,8152	22,989	NaN	NaN
26,2274	25,0856	25,1097	25,6537	25,5644	25,005	25,0062	25,8406	25,6025
22,8071	20,9759	21,513	22,4395	22,2555	22,3694	21,7258	23,1817	22,7115
21,9297	22,3728	22,3647	22,9352	23,0053	22,8291	22,6425	22,911	22,5535
NaN	22,0186	22,1571	NaN	21,7766	NaN	NaN	NaN	NaN
21,0876	20,5201	NaN	NaN	NaN	NaN	NaN	NaN	NaN
25,9962	26,0488	25,812	24,2059	24,2681	23,9538	24,0666	24,595	24,6215
20,6434	22,1092	21,9939	20,7832	20,4987	21,5495	21,5881	NaN	NaN
23,3291	24,0098	23,5735	23,2792	23,3318	NaN	22,4294	NaN	23,0813
24,1896	22,958	22,8766	21,8647	21,836	21,14	21,1887	22,0567	21,7613
23,146	26,1375	26,3112	24,5346	24,5086	25,2116	25,1051	23,8559	23,9556
21,6762	23,011	23,1395	22,2686	22,3308	23,7348	23,7289	25,569	25,4039
23,1627	24,023	23,9799	24,2134	23,8746	23,9173	23,9726	23,6823	23,7397
21,6775	22,0801	22,0727	22,6302	22,8709	23,2477	23,0642	22,0916	NaN
23,7324	22,4299	22,4874	23,9619	23,9774	23,7946	23,9786	23,9374	24,1954
NaN	21,9898	21,5984	21,4214	21,1257	NaN	NaN	NaN	NaN
23,5195	22,2658	21,9228	24,1316	24,0256	NaN	NaN	22,0792	22,2904
21,4787	21,6223	21,9583	21,413	21,655	22,261	22,2466	22,2332	22,3113
22,6498	22,5403	22,3007	22,6295	22,8117	23,2331	23,1915	22,6959	22,6076
20,7624	20,7596	20,7714	21,1854	21,8755	21,3736	21,5159	21,6251	21,0543
21,5993	22,442	23,0646	22,1264	21,9195	21,827	21,4936	NaN	21,7876
NaN	NaN	NaN	21,8503	NaN	22,5786	22,1998	22,3397	22,5522
20,2674	20,6304	NaN	20,3745	21,2222	20,8776	20,7225	21,6367	20,9269
22,8344	23,0127	22,8258	23,0572	22,9029	22,9665	23,3612	22,7984	22,584
NaN	21,827	22,0012	19,8527	NaN	NaN	NaN	NaN	NaN
NaN	NaN	20,0335	21,02	20,7544	20,4262	20,0858	NaN	NaN
24,1299	21,1704	21,1064	23,4775	23,3167	23,0199	23,043	22,0604	22,3326
20,6714	21,6163	21,6392	20,5727	20,6187	21,5483	21,5232	21,1091	20,9734
21,3725	20,5346	20,7799	22,4152	22,5638	NaN	NaN	20,7457	NaN
22,4676	22,5722	22,3953	22,7382	22,8951	22,4201	22,5096	22,929	22,9351
21,095	20,8051	20,7353	20,9295	20,9609	21,4481	21,231	NaN	NaN
24,1135	22,9643	22,7443	22,5976	22,6072	22,7674	22,6614	23,3542	23,4731
21,0711	21,3258	21,6597	21,3394	21,5343	22,6301	22,6964	21,9983	21,707
22,1794	21,6946	22,3962	21,7567	22,2186	22,2823	22,2349	22,0332	22,3483
21,9544	23,5022	23,4193	23,0207	22,672	23,2406	23,3012	22,4525	22,1488
22,3339	21,9277	21,5875	21,6012	21,6993	NaN	21,5894	22,3332	21,8097

19,7128	NaN	NaN	NaN	NaN	NaN	19,9777	NaN	NaN
25,4196	25,1627	25,2231	26,0537	26,0696	26,3567	26,1992	25,721	25,7161
NaN	23,6257	22,7312	NaN	NaN	NaN	NaN	NaN	NaN
21,8188	24,9256	24,8373	NaN	20,013	NaN	18,4234	27,4241	27,4765
21,0642	23,8652	23,8978	23,2829	22,913	21,2584	21,178	21,743	21,6413
22,557	23,1189	22,9846	22,0431	21,9303	22,4755	22,4269	22,377	22,4605
23,8933	24,5186	24,5834	24,2552	24,1021	23,7119	23,8255	23,7708	23,8086
22,7064	22,5162	22,3674	22,0434	22,3971	22,0292	22,0132	21,4178	22,0047
20,9781	21,3397	20,7754	21,5178	21,3298	21,5439	21,6385	NaN	NaN
26,5969	24,1481	24,1871	26,2559	26,2115	26,1168	26,094	25,7924	25,7937
27,2731	25,9583	25,8974	25,6123	25,572	23,3632	23,4702	23,5892	23,1462
23,7368	23,4315	23,4485	24,3609	24,7564	25,4521	25,2538	23,8534	23,9162
25,4839	25,8201	25,7676	23,3094	23,3766	22,3708	22,632	24,4854	24,3479
NaN	25,5533	24,5371	22,2179	22,2793	NaN	NaN	NaN	NaN
20,9981	21,9719	21,8487	22,0732	21,4749	22,8001	22,8935	NaN	NaN
29,4962	27,0966	27,024	27,0062	27,0331	28,5395	28,6755	29,267	29,2721
23,8202	24,0184	24,1429	24,0907	24,1174	24,3554	24,3058	23,9901	24,1216
21,6893	21,3894	21,5099	20,6483	20,5419	21,4858	21,5617	21,5441	22,092
23,8009	25,2268	25,268	21,9522	21,9268	NaN	21,1678	21,1699	21,2322
NaN	NaN	NaN	NaN	NaN	NaN	NaN	24,3395	24,192
23,8671	23,1163	23,1784	24,2332	24,1781	24,1692	24,0918	23,7989	23,5822
21,613	NaN	NaN	21,9085	22,0689	NaN	NaN	NaN	NaN
22,7486	21,9516	22,0338	22,7506	22,6562	23,5483	23,3187	22,0188	NaN
21,2607	21,6791	21,6329	21,182	21,189	21,4455	21,1226	NaN	NaN
NaN	NaN	19,9142	20,8378	20,6448	NaN	NaN	20,6363	20,3215
22,9106	22,5188	22,465	24,9321	25,0293	25,0803	25,0858	23,4574	23,4386
22,4362	22,4649	22,3192	22,7571	22,8663	22,9509	22,7779	22,0821	22,1412
21,0367	NaN	19,9753	20,5966	20,6876	20,8642	20,5785	20,7349	21,1547
20,6387	20,8026	20,6857	NaN	21,1541	21,028	21,6779	NaN	NaN
23,749	23,2967	23,4712	23,8399	23,8683	23,356	23,4749	23,1476	23,6096
20,9478	22,4511	22,4037	20,52	20,3041	NaN	NaN	NaN	NaN
24,8512	25,7055	25,7092	26,2241	26,2099	27,0964	27,1437	25,6588	25,7962
NaN	20,5259	20,6472	NaN	21,4519	NaN	20,7284	NaN	NaN
21,114	21,7457	21,2335	21,5718	21,8923	NaN	NaN	NaN	NaN
21,56	22,1638	22,1221	20,9758	21,0476	NaN	20,2683	20,9512	NaN
24,0853	23,1637	23,2527	22,8818	23,0813	22,6823	22,5754	23,9282	23,7403
21,5002	21,1758	20,5665	21,794	21,3143	21,5033	21,7456	20,5843	NaN
20,0471	19,6515	19,4915	20,9637	20,2787	19,9243	20,4532	20,4292	20,5063
24,6766	23,1881	23,1906	23,9562	23,9853	23,9372	23,8946	25,0694	24,7934
NaN	NaN	20,9346	NaN	21,3661	21,8335	NaN	NaN	NaN
NaN	22,1185	21,7928	NaN	NaN	NaN	NaN	NaN	NaN
22,6887	22,6606	22,6826	22,8671	22,831	23,3377	22,9909	23,1008	23,0513
24,181	23,5052	23,4896	23,2038	23,1813	23,9842	23,9831	24,8621	24,603
NaN	NaN	NaN	NaN	NaN	25,2671	25,2647	NaN	NaN
NaN	NaN	NaN	NaN	NaN	24,9596	24,934	NaN	NaN
NaN	NaN	NaN	NaN	NaN	23,901	23,923	NaN	NaN
23,2644	23,4244	23,3906	23,2562	23,3689	23,3902	23,5124	23,6174	23,6332
NaN	20,9457	20,7804	20,7479	20,9016	21,3612	21,9805	20,7149	21,6009
NaN	NaN	NaN	NaN	NaN	NaN	NaN	NaN	NaN
22,0013	NaN	21,8187	22,4543	22,832	22,904	23,0943	22,3821	22,538
21,8586	20,6379	NaN	20,6488	20,2838	NaN	NaN	NaN	NaN
NaN	21,5319	21,1207	NaN	NaN	NaN	NaN	NaN	NaN

NaN	18,4785	NaN	18,9517	18,5533	20,0234	20,486	25,3582	25,0601
24,8509	24,3356	24,3336	24,0995	23,9538	24,661	24,4482	24,6369	24,5195
NaN	21,5071	21,3924	NaN	20,6495	NaN	20,3076	NaN	NaN
NaN	22,7479	22,7009	NaN	NaN	NaN	NaN	NaN	NaN
22,1894	22,6153	22,8783	22,1742	22,0732	22,335	22,1914	23,2454	23,0302
21,0053	21,9083	22,059	20,7866	20,4717	NaN	NaN	20,0144	NaN
23,7758	23,8239	23,6629	25,83	25,2263	26,092	25,899	24	24,1081
21,1129	NaN	20,3777	21,4421	21,4087	21,4628	22,1512	21,1918	21,4588
NaN	20,788	20,1219	20,4341	19,7563	22,1873	22,2229	NaN	NaN
21,379	22,2522	22,147	NaN	21,7017	21,645	22,0018	NaN	NaN
23,1692	22,8758	23,3878	NaN	NaN	21,7084	22,5832	22,7045	23,0776
21,5875	21,0795	21,5166	22,1899	22,3424	21,8928	22,0088	21,2352	21,3451
21,1241	22,6134	22,7823	21,1911	21,5816	21,1552	NaN	NaN	NaN
NaN	21,0248	21,0283	NaN	NaN	NaN	NaN	NaN	NaN
19,9519	19,7713	19,9109	20,3445	20,5892	20,6674	20,4828	NaN	NaN
NaN	NaN	NaN	NaN	NaN	20,2055	NaN	NaN	NaN
23,6926	23,4862	23,342	23,6612	23,574	24,0872	24,0768	23,9572	23,9279
NaN	NaN	NaN	23,2557	23,5032	NaN	NaN	NaN	NaN
25,8905	25,1335	25,0571	26,5155	26,5696	26,6161	26,6633	25,5529	25,3051
26,6433	25,2667	25,2348	26,6169	26,5922	26,8413	26,8502	25,7914	25,8036
21,0738	21,3547	20,9713	21,8147	21,9652	NaN	NaN	NaN	NaN
21,0072	20,7594	20,9614	NaN	21,2354	NaN	NaN	NaN	NaN
27,5409	25,3191	25,2663	26,2452	26,1112	25,5517	25,1491	23,7667	24,6061
21,9904	23,0466	22,5075	22,1445	22,4761	22,097	22,5927	NaN	22,2303
19,7446	25,5123	25,1264	20,0803	NaN	NaN	NaN	NaN	NaN
NaN	23,0321	23,1301	NaN	21,721	NaN	NaN	NaN	NaN
22,6496	NaN	21,2313	23,1854	23,0595	23,6768	23,9616	21,9897	22,821
19,5002	20,576	20,6605	NaN	20,1181	21,0528	21,2509	NaN	NaN
20,692	20,7969	20,7996	21,0569	20,6076	NaN	NaN	NaN	NaN
NaN	21,241	21,5109	20,9902	20,9501	NaN	NaN	20,8589	NaN
20,5119	21,6962	21,8396	20,462	20,4004	20,5844	NaN	NaN	NaN
NaN	NaN	NaN	19,8011	19,8526	19,7326	19,9388	NaN	NaN
20,8318	19,9403	20,2745	20,4508	20,2934	20,8252	NaN	21,0213	20,9643
NaN	NaN	19,7611	NaN	NaN	NaN	19,9485	NaN	NaN
NaN	NaN	NaN	NaN	NaN	NaN	NaN	NaN	NaN
20,454	20,2103	19,935	20,274	20,343	NaN	20,1693	20,3464	20,1993
22,4666	22,1367	21,8336	20,94	20,8849	21,0154	21,11	20,7172	21,0162
21,1513	20,1518	NaN	20,662	20,7764	20,857	20,8135	21,234	21,0486
NaN	21,8128	21,4918	NaN	NaN	NaN	NaN	NaN	NaN
20,7521	21,0958	21,4567	21,917	21,6907	21,5092	21,4028	21,2703	21,3994
NaN	NaN	NaN	NaN	20,7153	20,1614	20,3939	NaN	NaN
20,4348	19,9902	20,2042	20,916	20,3549	21,0399	21,6298	21,6781	21,5018
20,595	21,5133	21,6278	20,8135	20,5513	21,6425	20,7639	20,9142	NaN
NaN	NaN	21,3277	21,3532	NaN	20,9562	NaN	NaN	NaN
21,1084	21,6888	21,7178	20,9146	21,8723	20,5772	20,9287	22,228	21,6981
NaN	18,8168	18,9694	NaN	NaN	20,661	21,4541	28,0723	27,9647
NaN	20,9011	20,6179	20,9783	20,8757	20,9164	20,3288	NaN	20,8248
21,228	21,325	21,6519	21,8918	21,4426	21,497	21,2017	NaN	NaN
22,8233	21,6759	21,7479	23,0708	23,2011	22,4707	22,4034	23,295	23,2284
NaN	19,8684	19,9951	NaN	NaN	NaN	NaN	NaN	NaN
22,36	22,3035	22,0687	22,4555	22,6604	21,3485	21,6397	22,4663	22,3378
NaN	NaN	NaN	20,1982	NaN	NaN	NaN	NaN	NaN

21,2681	20,5677	20,5777	21,3051	20,8513	20,3275	20,8063	NaN	NaN
NaN	22,5497	22,3421	21,8309	22,1436	21,6981	21,5199	NaN	NaN
NaN	NaN	NaN	20,5337	20,5844	NaN	NaN	NaN	NaN
23,8874	25,5337	25,5643	25,5841	25,4753	25,9489	25,8801	24,8836	24,8658
NaN	21,7888	21,8587	NaN	NaN	NaN	NaN	24,8956	24,9216
21,7136	20,9775	21,0031	21,0586	21,186	22,0864	22,0946	22,045	21,8614
27,286	26,3955	26,3535	25,8018	25,7175	26,0333	25,8895	25,8915	25,9691
21,8027	21,3407	NaN	21,9788	22,0111	22,5574	22,3237	NaN	NaN
NaN	21,3948	NaN	22,0029	22,2758	21,7772	21,9288	20,6272	NaN
19,997	18,7997	NaN	20,5345	21,2455	NaN	NaN	NaN	NaN
21,4324	21,2145	20,9009	NaN	21,0119	20,6046	21,1998	22,2253	21,5424
25,133	26,3405	26,2769	26,7469	26,669	27,9651	27,9828	26,1468	26,1968
NaN	NaN	NaN	22,408	22,7915	23,6516	23,2761	NaN	NaN
NaN	21,8375	21,925	NaN	NaN	NaN	20,01	NaN	NaN
NaN	20,2437	20,4379	20,6545	20,3062	21,1958	21,1276	NaN	NaN
20,2747	20,1289	20,0575	21,5525	21,7672	20,7326	20,6892	NaN	NaN
21,5914	20,8913	20,7342	23,766	23,6392	22,1301	21,9415	20,6101	NaN
NaN	23,3693	22,9694	22,4506	22,4272	NaN	NaN	NaN	NaN
21,8344	23,1806	23,5436	22,3185	23,0152	22,2383	22,6371	22,3749	22,0311
20,6768	20,4975	20,5094	20,942	21,4108	20,4133	21,7375	NaN	NaN
24,2762	25,0316	25,0058	24,7748	24,9135	24,9162	24,7189	24,3034	24,4628
NaN	21,3745	21,0642	21,7355	21,6014	21,8024	21,5565	21,2386	21,2827
26,0539	26,2415	26,0401	25,9803	26,0308	26,0608	26,0052	26,6077	26,5808
25,1878	24,5609	24,4755	25,6451	25,4866	NaN	NaN	22,8951	22,7874
20,0784	NaN	20,1391	20,8404	20,4603	20,3906	21,2089	20,4805	20,1739
22,5119	24,1554	24,0891	22,7448	23,1062	22,7762	22,8361	21,579	21,7601
NaN	20,9833	20,8033	NaN	NaN	NaN	NaN	NaN	NaN
23,0681	NaN	23,0876	24,167	23,5321	24,636	24,3503	22,6341	22,4805
25,9953	26,3941	26,3638	25,8127	25,5636	NaN	NaN	25,8247	25,756
20,7367	23,4154	23,3502	21,4889	21,3909	22,3322	22,4242	21,9573	21,8495
NaN	22,1146	21,8689	NaN	NaN	NaN	NaN	NaN	NaN
25,5226	27,2919	27,2889	25,3102	25,1973	22,3349	22,5347	24,015	24,1666
22,4526	23,0081	22,5804	22,3661	22,0449	23,5861	23,4371	22,5532	22,422
23,5679	23,5953	23,6338	24,0727	23,7898	23,9287	23,7876	23,5919	23,8104
21,4788	NaN	NaN	20,8225	20,6829	20,7072	NaN	NaN	20,6223
NaN	20,7771	NaN	20,9383	NaN	21,3962	21,5031	21,5814	NaN
20,9026	21,2984	20,988	22,7626	22,2735	21,7698	21,7774	NaN	21,1284
20,6446	20,5604	NaN	NaN	20,7302	20,6312	20,828	21,1039	20,8438
21,4468	20,9185	20,7485	20,7964	NaN	20,8333	21,2459	21,999	20,4571
20,5223	NaN	NaN	NaN	NaN	NaN	NaN	21,0849	21,1385
23,7369	26,5464	26,5772	25,04	25,1665	22,3168	22,6163	23,9026	23,7509
20,843	21,399	21,34	NaN	NaN	NaN	NaN	NaN	NaN
NaN	19,4495	19,8042	NaN	19,5455	19,7321	19,7656	NaN	NaN
21,7798	23,406	22,9134	20,9971	21,0408	20,7719	21,5757	NaN	NaN
NaN	NaN	21,0358	21,4119	21,4774	21,1478	21,0496	NaN	NaN
21,287	NaN	NaN	20,3184	19,7193	NaN	NaN	NaN	19,7496
NaN	21,5046	21,4107	NaN	NaN	NaN	NaN	NaN	NaN
20,5886	20,7068	20,8781	NaN	NaN	20,9858	21,114	NaN	NaN
NaN	NaN	NaN	NaN	NaN	NaN	NaN	NaN	NaN
NaN	NaN	NaN	NaN	NaN	NaN	20,5441	20,7579	NaN
21,4317	22,4496	22,5033	22,943	23,0163	23,3593	23,5311	22,4419	22,6525
20,2959	20,807	20,665	21,1981	21,2387	NaN	20,8639	NaN	21,1125

20,3967	NaN	NaN	20,1336	NaN	NaN	NaN	NaN	NaN
21,4913	23,9238	23,8819	23,045	22,9182	24,9136	24,8726	21,7303	21,742
26,2847	24,8181	24,7966	27,9413	27,7839	26,8158	26,7939	26,6811	26,7443
24,1483	22,7444	22,8553	25,4558	25,427	25,9093	25,9007	24,405	24,5503
25,4541	23,8067	23,8151	26,627	26,6443	26,7394	26,924	24,9067	NaN
24,3953	24,6423	24,3789	25,5928	25,3891	25,3026	25,4604	24,4415	24,3324
NaN	21,8883	21,7336	NaN	NaN	NaN	NaN	NaN	NaN
NaN	NaN	NaN	NaN	NaN	NaN	NaN	NaN	NaN
NaN	20,4021	20,7618	NaN	NaN	NaN	20,8852	NaN	NaN
NaN	20,5509	20,4854	20,8451	20,9636	20,8042	21,2718	NaN	20,8203
20,4124	20,5039	20,5836	NaN	NaN	NaN	20,6622	NaN	NaN
NaN	19,7281	NaN	19,9644	NaN	NaN	NaN	NaN	NaN
20,4107	23,2086	23,5858	NaN	NaN	NaN	20,5806	NaN	NaN
NaN	NaN	NaN	NaN	NaN	NaN	NaN	NaN	NaN
NaN	NaN	NaN	19,9016	NaN	NaN	NaN	NaN	NaN
NaN	20,9056	NaN	20,036	NaN	20,7573	20,7321	NaN	NaN
21,6046	21,7203	21,9457	21,0552	20,8119	20,6123	20,9068	20,8372	NaN
NaN	NaN	21,2997	NaN	NaN	NaN	NaN	NaN	NaN
NaN	NaN	NaN	NaN	NaN	NaN	NaN	NaN	NaN
20,5026	NaN	NaN	NaN	20,55	NaN	20,7214	NaN	NaN
20,4645	22,6196	22,6784	NaN	NaN	NaN	NaN	NaN	NaN
NaN	22,4247	21,2341	NaN	19,5863	NaN	NaN	NaN	NaN
21,0263	NaN	NaN	22,2632	21,7728	21,7446	21,725	21,8548	NaN
21,34	22,5539	NaN	NaN	NaN	21,7369	NaN	NaN	NaN
21,1226	21,5593	21,3793	21,314	20,7979	21,3597	21,5231	22,5418	22,319
20,8379	21,3661	21,6061	20,6216	20,735	20,5654	20,5091	21,1163	NaN
22,6074	23,7869	23,8244	22,2318	22,1428	NaN	NaN	NaN	NaN
NaN	NaN	NaN	NaN	20,7124	20,5403	20,1493	NaN	NaN
24,1899	23,9184	23,8897	24,391	23,6896	24,4315	24,3178	NaN	NaN
NaN	19,9872	NaN	20,2337	20,1124	20,6883	20,4231	NaN	NaN
NaN	20,0246	NaN	20,5884	NaN	21,6701	21,4498	23,9776	23,6044
22,2636	22,1098	21,9226	21,7218	21,779	22,4842	22,4033	22,2502	21,6937
NaN	20,8801	20,5697	NaN	21,1651	20,8397	21,3364	NaN	NaN
20,3835	20,4212	NaN	NaN	NaN	20,7359	NaN	NaN	NaN
NaN	20,7242	21,0841	NaN	NaN	NaN	NaN	NaN	NaN
NaN	21,1843	21,3615	20,3587	NaN	21,2186	20,8447	NaN	NaN
NaN	NaN	NaN	NaN	20,8638	NaN	NaN	NaN	NaN
NaN	20,9496	NaN	20,5844	20,8147	NaN	NaN	NaN	NaN
NaN	NaN	NaN	NaN	NaN	NaN	NaN	NaN	NaN
NaN	20,3097	20,1476	NaN	NaN	NaN	NaN	NaN	NaN
NaN	NaN	NaN	NaN	20,1865	20,7374	NaN	NaN	NaN
NaN	NaN	NaN	NaN	NaN	NaN	NaN	NaN	NaN
20,0867	24,6616	25,2691	21,2064	NaN	19,2291	19,2196	NaN	NaN
22,2272	20,7734	20,9162	22,2284	22,5596	NaN	21,6607	NaN	21,8727
20,3927	NaN	NaN	NaN	NaN	21,6554	21,8481	NaN	NaN
22,2013	23,1124	23,2893	21,6916	21,6952	21,8397	21,879	NaN	21,7832
NaN	NaN	21,1172	21,4835	21,8868	22,0537	22,1886	22,5231	NaN
NaN	NaN	NaN	19,1273	19,0554	NaN	NaN	NaN	NaN
20,8671	20,5544	20,3864	20,8861	20,5074	20,8906	21,1829	NaN	20,0312
NaN	21,418	21,5085	NaN	NaN	NaN	NaN	NaN	NaN
NaN	NaN	NaN	NaN	NaN	NaN	NaN	20,5985	21,2101
21,0832	21,2561	21,2934	20,3115	NaN	NaN	NaN	NaN	NaN

NaN	21,8306	21,7299	19,7906	19,3286	19,9389	NaN	NaN	NaN
20,6381	20,4402	20,4405	20,5413	20,6775	NaN	NaN	NaN	NaN
21,1763	NaN	20,2704	21,7425	22,183	21,1462	21,2235	NaN	NaN
20,283	NaN	NaN	20,0687	NaN	20,3547	20,1846	NaN	NaN
NaN	20,5462	20,6034	20,8014	NaN	22,0375	21,1071	NaN	NaN
20,6616	20,9064	21,1373	20,4748	20,6209	20,9533	20,7182	20,9906	NaN
NaN	20,7454	20,7315	NaN	NaN	NaN	NaN	NaN	NaN
21,6456	21,619	21,3116	20,4441	20,564	21,0071	21,024	NaN	NaN
20,6527	21,5237	21,7037	NaN	NaN	NaN	NaN	NaN	NaN
20,3656	21,5803	21,5079	20,055	19,9462	20,5587	20,5714	NaN	NaN
NaN	21,8601	21,6472	NaN	NaN	NaN	NaN	NaN	NaN
NaN	NaN	NaN	NaN	NaN	NaN	NaN	NaN	NaN
21,8588	22,6903	22,6023	24,0913	25,0178	24,047	23,9745	NaN	23,8931
NaN	NaN	NaN	NaN	NaN	NaN	NaN	NaN	NaN
NaN	21,0381	21,4136	NaN	NaN	NaN	NaN	NaN	NaN
20,3464	19,8419	NaN	21,2316	21,4008	20,2289	20,3103	NaN	20,1613
22,053	NaN	NaN	NaN	NaN	NaN	NaN	NaN	NaN
27,1144	26,5574	26,5151	26,0207	25,9245	25,4454	25,3245	26,7542	26,5835
20,7183	20,8771	20,9594	20,8642	20,8842	21,0819	21,0692	NaN	NaN
23,4753	22,4192	22,4295	22,9536	23,152	NaN	NaN	NaN	NaN
NaN	NaN	NaN	20,4299	NaN	NaN	NaN	NaN	NaN
NaN	NaN	NaN	NaN	NaN	NaN	NaN	NaN	NaN
21,1246	NaN	NaN	26,3506	26,3537	NaN	NaN	NaN	NaN
21,062	NaN	NaN	NaN	NaN	NaN	NaN	NaN	NaN
23,9849	23,2781	23,3015	25,7217	25,6157	24,5289	24,8228	24,6768	24,6629
NaN	20,6261	NaN	NaN	NaN	20,6176	20,7325	NaN	NaN
19,2824	20,5557	20,2482	20,3443	20,4592	21,5514	21,5499	NaN	NaN
NaN	NaN	NaN	NaN	NaN	NaN	NaN	NaN	NaN
NaN	NaN	19,9158	NaN	NaN	NaN	NaN	NaN	NaN
NaN	NaN	NaN	NaN	NaN	NaN	NaN	NaN	NaN
20,9269	20,7903	20,8025	21,2605	20,9622	20,9602	21,1456	NaN	NaN
21,0491	20,836	20,5805	20,0948	20,2118	20,5964	21,1536	21,2154	21,2129
NaN	NaN	NaN	NaN	19,8228	NaN	NaN	NaN	NaN
NaN	NaN	NaN	NaN	NaN	NaN	19,6974	NaN	NaN
20,8667	NaN	NaN	NaN	NaN	NaN	NaN	NaN	NaN
21,8611	20,4727	20,4576	22,2435	22,1634	22,0577	21,9355	22,2075	22,4497
NaN	20,6086	20,5831	NaN	NaN	NaN	NaN	NaN	NaN
NaN	NaN	NaN	NaN	NaN	NaN	NaN	NaN	NaN
NaN	NaN	NaN	NaN	NaN	NaN	NaN	NaN	NaN
NaN	20,92	21,5097	NaN	NaN	NaN	NaN	NaN	NaN
NaN	NaN	19,3922	NaN	19,6296	NaN	NaN	NaN	NaN
22,135	20,705	20,7105	NaN	NaN	NaN	NaN	NaN	NaN
NaN	20,0695	19,932	21,6257	21,3026	20,9565	20,2797	20,1929	NaN
22,5218	22,4841	22,784	21,8217	NaN	22,2617	22,345	22,385	22,7046
20,7569	20,6566	NaN	20,7383	21,5635	21,3095	20,859	21,2766	21,504
21,0889	21,319	21,1598	21,0939	NaN	21,4977	21,563	21,3174	NaN
23,587	21,1674	20,8691	22,9869	23,1376	21,1896	21,4546	22,3409	22,4161
NaN	NaN	NaN	NaN	NaN	NaN	NaN	NaN	NaN
20,2241	20,9044	20,8174	19,793	NaN	NaN	20,4682	NaN	20,5373
NaN	NaN	NaN	19,8767	NaN	20,1393	NaN	NaN	NaN
NaN	22,4119	21,8314	20,8878	NaN	22,1283	21,9787	21,5559	21,3277
23,4585	23,5216	23,7405	24,4385	24,2386	25,1465	25,2376	23,4082	23,4598

NaN	20,8733	NaN	NaN	NaN	NaN	NaN	NaN	NaN
20,0824	NaN	19,6719	NaN	NaN	19,4458	NaN	20,3239	20,2512
NaN	NaN	NaN	NaN	NaN	NaN	NaN	NaN	NaN
19,4923	20,6149	20,6172	19,4838	NaN	NaN	19,8019	NaN	NaN
21,4099	26,8515	26,9038	NaN	19,6598	NaN	19,9366	26,4298	26,1491
NaN	24,0733	23,6838	NaN	NaN	NaN	NaN	NaN	NaN
NaN	NaN	NaN	NaN	NaN	23,6976	23,7564	NaN	NaN
NaN	NaN	22,0037	NaN	NaN	NaN	NaN	NaN	NaN
NaN	NaN	NaN	NaN	NaN	NaN	NaN	NaN	NaN
23,4387	22,4976	22,6959	24,5702	24,2613	24,2671	24,7665	22,9411	23,9732
21,5411	NaN	NaN	NaN	NaN	21,2122	20,855	NaN	NaN
23,1451	22,8809	22,5424	23,0361	22,9349	23,3665	23,3001	22,4725	22,5275
NaN	NaN	NaN	NaN	NaN	NaN	NaN	NaN	NaN
25,9957	24,9516	24,9768	25,3668	25,3404	25,8562	25,7894	25,2215	25,2339
20,0182	19,6225	19,6444	19,769	19,8761	19,9295	19,8132	19,8644	NaN
20,436	NaN	NaN	NaN	20,7379	20,9128	20,9516	20,6252	20,7427
22,8678	22,6596	22,7287	22,4433	22,3384	22,2301	22,2383	22,2344	22,138
NaN	NaN	NaN	NaN	NaN	NaN	NaN	NaN	NaN
25,7047	NaN	20,8462	NaN	NaN	NaN	NaN	NaN	NaN
23,9932	22,7313	22,7046	24,2381	24,3385	23,6398	23,5057	23,5902	23,6857
NaN	22,1095	21,7968	NaN	NaN	NaN	NaN	NaN	NaN
21,9369	22,0166	22,1514	23,6808	23,5692	23,655	23,6508	23,0467	23,1058
NaN	NaN	NaN	NaN	NaN	NaN	21,613	NaN	NaN
19,4133	NaN	19,6196	NaN	NaN	19,784	19,7468	NaN	NaN
NaN	21,014	21,2816	NaN	NaN	NaN	NaN	NaN	NaN
23,2099	22,4337	22,6141	23,2259	23,6697	22,9181	23,0033	22,6279	22,6699
23,2897	22,3264	22,0693	23,734	23,8085	23,9874	23,6582	24,0261	24,1126
NaN	20,4566	20,4694	20,1088	19,8067	NaN	NaN	NaN	NaN
NaN	23,1313	23,1225	19,4243	20,5008	NaN	NaN	NaN	NaN
20,1854	NaN	NaN	NaN	NaN	20,7895	20,7439	20,5115	20,7273
NaN	NaN	19,4704	19,8554	19,7099	NaN	19,7877	NaN	NaN
21,1684	24,1084	24,1061	22,1466	22,0535	22,3659	22,3448	21,1393	21,1171
NaN	21,3526	21,0877	NaN	NaN	NaN	NaN	NaN	NaN
23,9196	22,3293	22,4864	21,5235	21,2937	NaN	NaN	NaN	21,2765
NaN	23,0154	22,8754	21,7399	21,8605	22,6284	22,0694	NaN	NaN
22,8618	NaN	NaN	20,7842	NaN	NaN	NaN	NaN	NaN
NaN	NaN	NaN	NaN	NaN	NaN	NaN	NaN	NaN
20,5529	20,6843	20,6219	20,2467	20,5991	NaN	20,5586	NaN	NaN
29,6372	28,8174	28,7364	27,6373	27,6518	26,2855	26,4197	28,4091	28,3952
21,2989	21,0513	21,0676	NaN	21,4009	21,5974	21,8015	NaN	NaN
20,183	22,7525	22,6301	20,9532	21,1226	20,3954	20,4002	NaN	NaN
23,2376	22,6937	22,6381	24,1329	24,2034	22,7486	21,5565	22,865	23,3961
20,9115	NaN	NaN	20,7622	20,8855	20,0957	20,2827	NaN	20,9228
21,4957	22,5525	22,649	21,4747	21,2613	20,7516	20,8303	21,5976	21,1528
NaN	19,9456	20,4455	NaN	NaN	NaN	NaN	NaN	NaN
23,979	NaN	NaN	24,1853	24,2183	24,0844	24,0945	23,3848	23,0712
NaN	NaN	NaN	21,4218	21,2503	NaN	NaN	NaN	NaN
20,8735	20,3598	20,5762	21,2688	21,5514	NaN	20,4202	NaN	NaN
20,4752	NaN	21,1855	21,53	23,5473	24,1349	23,6253	22,9563	22,9088
25,0128	21,8219	21,6876	20,7241	NaN	NaN	21,929	NaN	NaN
NaN	27,2126	27,3307	NaN	NaN	28,7413	28,727	NaN	NaN
NaN	NaN	NaN	NaN	NaN	NaN	NaN	NaN	NaN

21,2573	NaN	NaN	22,0548	NaN	NaN	NaN	NaN	21,4422
22,5174	21,9763	21,9486	22,1226	22,1471	22,2785	21,9036	22,6164	22,7723
NaN	NaN	NaN	NaN	NaN	NaN	NaN	NaN	NaN
NaN	NaN	NaN	NaN	NaN	NaN	NaN	NaN	NaN
NaN	NaN	NaN	NaN	NaN	NaN	NaN	NaN	NaN
21,7802	21,4711	21,3752	NaN	21,1888	NaN	NaN	21,5794	22,2934
NaN	NaN	19,8455	NaN	NaN	NaN	NaN	NaN	NaN
20,6857	20,9765	21,0078	NaN	NaN	NaN	21,3777	NaN	NaN
NaN	NaN	NaN	NaN	NaN	NaN	NaN	NaN	NaN
NaN	20,5542	20,9858	NaN	NaN	20,885	20,8719	21,6219	21,837
NaN	NaN	NaN	NaN	NaN	NaN	NaN	NaN	NaN
NaN	20,6613	21,0313	NaN	20,1043	19,6909	NaN	NaN	NaN
NaN	NaN	NaN	NaN	NaN	NaN	NaN	NaN	NaN
NaN	20,5691	20,7668	NaN	NaN	NaN	NaN	NaN	NaN
20,4158	NaN	20,2143	NaN	NaN	NaN	NaN	NaN	NaN
NaN	22,4884	22,3336	18,6309	NaN	NaN	NaN	NaN	NaN
NaN	NaN	NaN	NaN	NaN	NaN	NaN	NaN	NaN
NaN	NaN	NaN	NaN	NaN	NaN	NaN	NaN	NaN
NaN	20,9752	20,6162	NaN	NaN	NaN	NaN	NaN	NaN
NaN	NaN	NaN	NaN	NaN	NaN	NaN	NaN	NaN
20,2139	NaN	20,1998	NaN	20,0307	NaN	19,9631	NaN	NaN
NaN	20,1039	NaN	NaN	NaN	NaN	NaN	NaN	NaN
NaN	NaN	NaN	NaN	NaN	NaN	NaN	NaN	NaN
NaN	NaN	21,7514	NaN	NaN	NaN	NaN	NaN	NaN
NaN	NaN	20,4539	NaN	NaN	NaN	NaN	NaN	NaN
NaN	NaN	NaN	NaN	NaN	NaN	NaN	NaN	NaN
NaN	20,8036	NaN	20,1892	NaN	NaN	NaN	NaN	NaN
NaN	NaN	NaN	NaN	NaN	NaN	NaN	NaN	NaN
NaN	NaN	NaN	21,7073	21,8485	NaN	NaN	25,5339	25,3629
20,2703	NaN	NaN	20,5878	20,3104	NaN	NaN	NaN	NaN
NaN	21,9775	21,5405	23,2189	23,089	24,0748	24,024	22,5861	NaN
21,2675	NaN	NaN	22,2314	22,1936	22,4637	22,5672	NaN	NaN
NaN	NaN	NaN	NaN	NaN	NaN	NaN	NaN	NaN
21,1081	NaN	NaN	21,7227	21,8181	21,8253	21,1762	NaN	NaN
NaN	NaN	NaN	NaN	NaN	NaN	NaN	NaN	NaN
23,709	28,5292	28,6091	25,2595	25,3113	22,8449	22,8444	22,4484	23,0856
NaN	NaN	NaN	NaN	NaN	NaN	NaN	NaN	NaN
NaN	20,8917	NaN	NaN	NaN	NaN	NaN	NaN	NaN
NaN	NaN	NaN	24,3288	25,1962	24,7654	24,5516	NaN	NaN
NaN	NaN	NaN	23,7447	22,634	22,9052	NaN	NaN	NaN
20,7018	NaN	NaN	20,7233	NaN	NaN	NaN	NaN	NaN
24,5511	23,7158	23,665	25,3108	24,9899	24,8731	24,9283	23,3491	23,5463
NaN	NaN	NaN	NaN	NaN	20,2106	20,1104	NaN	NaN
NaN	NaN	NaN	NaN	NaN	NaN	NaN	NaN	NaN
NaN	NaN	20,2004	NaN	NaN	NaN	NaN	NaN	NaN
NaN	NaN	20,5906	21,1879	21,1772	NaN	NaN	NaN	NaN
NaN	NaN	NaN	22,2431	22,1008	NaN	23,1627	NaN	NaN
NaN	NaN	NaN	NaN	NaN	NaN	NaN	NaN	NaN
21,3047	22,4759	22,125	22,2706	22,3981	22,0935	22,1061	22,7119	22,0369
NaN	21,2218	21,2185	NaN	NaN	NaN	NaN	NaN	NaN
21,9108	22,4542	22,3127	22,2079	22,3964	21,8705	NaN	NaN	NaN
24,2124	24,1446	23,8403	24,4031	24,3631	23,3884	24,4232	21,2414	21,4519

NaN	20,423	NaN	NaN	NaN	NaN	NaN	NaN	NaN
NaN	NaN	NaN	NaN	NaN	NaN	NaN	NaN	NaN
22,4378	22,1443	21,763	22,0241	22,2563	21,6316	22,452	NaN	NaN
NaN	21,6662	21,9153	19,9991	20,1987	NaN	20,4402	NaN	NaN
NaN	NaN	NaN	21,3431	21,4456	NaN	21,9127	NaN	NaN
NaN	NaN	NaN	NaN	NaN	NaN	NaN	NaN	NaN
NaN	NaN	NaN	NaN	NaN	NaN	NaN	NaN	NaN
NaN	NaN	NaN	20,8296	20,5462	21,2361	21,1397	21,1902	21,2993
NaN	21,6145	NaN	NaN	NaN	NaN	NaN	NaN	NaN
NaN	20,5206	NaN	NaN	NaN	NaN	NaN	NaN	NaN
NaN	NaN	NaN	NaN	NaN	NaN	NaN	NaN	NaN
NaN	NaN	20,2235	NaN	NaN	NaN	NaN	NaN	NaN
NaN	21,0233	21,3171	NaN	NaN	NaN	NaN	NaN	NaN
20,9888	NaN	20,6811	21,5406	NaN	21,5328	21,3756	NaN	NaN
NaN	NaN	19,978	NaN	NaN	NaN	20,8837	NaN	NaN
NaN	NaN	NaN	20,9205	NaN	20,1999	NaN	NaN	NaN
NaN	NaN	NaN	NaN	NaN	NaN	NaN	NaN	NaN
20,9725	21,3335	21,3439	21,1163	20,9879	20,5039	20,633	20,7275	20,4719
20,2755	NaN	NaN	20,5393	20,5961	20,6579	21,0289	NaN	NaN
NaN	21,1763	NaN	22,0448	21,8718	21,8252	NaN	NaN	NaN
21,2263	20,867	20,9404	20,4427	20,5641	NaN	21,6357	22,5491	22,8276
NaN	NaN	NaN	NaN	21,3428	NaN	NaN	NaN	NaN
21,3896	NaN	20,0006	NaN	NaN	21,7892	NaN	NaN	NaN
NaN	NaN	NaN	NaN	NaN	NaN	NaN	NaN	NaN
NaN	21,5109	21,4969	NaN	NaN	NaN	NaN	NaN	NaN
NaN	NaN	NaN	NaN	NaN	NaN	NaN	NaN	NaN
NaN	NaN	NaN	NaN	NaN	NaN	NaN	NaN	NaN
NaN	20,2821	20,4408	NaN	NaN	NaN	NaN	NaN	NaN
NaN	20,0626	20,1628	19,7259	NaN	20,9226	NaN	NaN	NaN
20,3686	NaN	NaN	20,1877	NaN	NaN	NaN	NaN	NaN
NaN	NaN	NaN	NaN	NaN	NaN	NaN	NaN	NaN
21,6397	21,0915	20,9632	21,2222	21,437	21,7427	21,6125	21,7455	21,5292
NaN	NaN	NaN	NaN	NaN	20,9646	NaN	NaN	NaN
NaN	NaN	NaN	NaN	NaN	NaN	NaN	NaN	NaN
21,0234	20,3487	NaN	NaN	NaN	NaN	19,6738	21,4908	21,2067
NaN	NaN	NaN	NaN	21,1988	21,4627	22,0635	NaN	NaN
23,0288	22,5879	22,325	21,4465	21,565	22,6647	22,7616	22,5753	23,1167
30,1213	23,4078	23,1186	23,7517	23,7345	27,3218	27,2175	25,8599	NaN
23,1733	23,7499	23,6676	23,9662	23,5057	NaN	24,213	NaN	NaN
19,9435	NaN	NaN	NaN	NaN	20,2946	NaN	NaN	NaN
NaN	NaN	19,6215	NaN	19,6594	NaN	NaN	NaN	NaN
24,3953	23,1733	23,4752	25,7539	25,7758	25,3062	25,3585	24,2141	24,15
22,1482	20,9714	20,7397	23,1236	23,221	NaN	21,3782	21,9105	22,2189
NaN	NaN	NaN	NaN	NaN	NaN	NaN	NaN	NaN
20,1225	20,4843	NaN	NaN	NaN	19,9058	NaN	NaN	NaN
NaN	20,1034	20,2589	NaN	NaN	NaN	NaN	19,2158	NaN
NaN	19,8784	19,734	NaN	NaN	NaN	NaN	NaN	NaN
NaN	NaN	NaN	21,3863	21,4514	21,3876	NaN	NaN	NaN
NaN	NaN	NaN	NaN	NaN	NaN	NaN	NaN	NaN
20,9765	21,715	21,4804	21,5068	21,3394	NaN	21,3416	21,473	21,6321
NaN	20,8051	20,9227	NaN	19,4196	NaN	NaN	NaN	NaN
NaN	21,1444	21,1648	19,8593	NaN	NaN	NaN	NaN	NaN

NaN	21,676	21,592	NaN	NaN	NaN	NaN	NaN	NaN
NaN	20,9934	NaN	22,1646	NaN	NaN	NaN	NaN	NaN
NaN	NaN	NaN	NaN	NaN	NaN	NaN	NaN	NaN
NaN	19,9826	NaN	19,9201	NaN	20,7013	20,429	NaN	NaN
NaN	NaN	NaN	NaN	NaN	NaN	NaN	NaN	NaN
21,497	22,2506	22,2365	NaN	NaN	NaN	NaN	22,6861	NaN
22,0726	21,9783	21,6279	22,2424	22,4753	21,9872	22,1344	NaN	NaN
25,4005	25,5171	25,652	25,0026	24,9656	25,9549	25,8967	25,5927	25,8503
NaN	NaN	NaN	NaN	NaN	NaN	NaN	NaN	NaN
NaN	NaN	NaN	NaN	NaN	NaN	NaN	21,0213	21,0668
22,7195	21,5897	21,9975	22,2839	22,7666	23,1383	22,9711	NaN	NaN
22,6665	25,8906	25,6006	23,0439	NaN	NaN	NaN	NaN	NaN
NaN	NaN	NaN	NaN	NaN	NaN	NaN	NaN	NaN
23,2134	NaN	NaN	NaN	NaN	23,5122	23,6499	NaN	NaN
NaN	NaN	NaN	NaN	NaN	NaN	NaN	NaN	NaN
22,9803	22,4981	NaN	25,6754	25,747	25,4899	25,3713	23,4352	23,4574
NaN	NaN	NaN	20,111	20,1158	NaN	NaN	NaN	NaN
NaN	20,474	20,3979	19,9376	20,4109	20,2527	20,3008	NaN	NaN
NaN	NaN	NaN	NaN	20,0865	NaN	20,3995	20,1814	NaN
NaN	23,3467	23,4442	24,2241	24,6607	24,1335	24,5216	24,0582	24,0611
NaN	NaN	20,2168	NaN	NaN	NaN	NaN	NaN	NaN
20,6685	NaN	20,1541	20,9599	21,0461	20,9607	21,2632	20,764	NaN
23,0275	27,9881	24,8851	23,3423	23,3909	NaN	NaN	NaN	NaN
NaN	20,7572	NaN	NaN	NaN	21,1313	21,2286	NaN	NaN
22,3721	NaN	NaN	NaN	NaN	NaN	NaN	NaN	NaN
20,01	NaN	20,068	20,6425	20,4461	20,6697	20,4255	NaN	NaN
NaN	NaN	NaN	21,5282	21,9373	21,3137	21,6787	NaN	NaN
23,8515	23,2782	23,3791	24,7254	25,1555	25,059	24,366	23,7966	23,7408
NaN	20,7106	NaN	NaN	NaN	NaN	NaN	NaN	NaN
NaN	NaN	NaN	NaN	19,1856	NaN	20,3975	NaN	NaN
NaN	21,0057	21,0557	NaN	NaN	NaN	NaN	NaN	NaN
NaN	NaN	NaN	NaN	NaN	NaN	NaN	NaN	NaN
21,9118	NaN	NaN	20,9663	20,7022	NaN	NaN	20,6048	NaN
20,6723	20,6924	20,8418	NaN	NaN	NaN	NaN	NaN	NaN
21,424	21,1232	20,978	NaN	NaN	NaN	NaN	NaN	NaN
NaN	19,5234	19,6819	NaN	NaN	NaN	NaN	NaN	NaN
21,2168	NaN	NaN	NaN	NaN	NaN	NaN	NaN	NaN
NaN	20,3555	20,35	19,9042	19,797	NaN	NaN	NaN	NaN
NaN	NaN	NaN	NaN	NaN	NaN	20,8244	NaN	NaN
NaN	NaN	NaN	NaN	NaN	NaN	NaN	NaN	NaN
NaN	NaN	NaN	NaN	NaN	NaN	NaN	NaN	NaN
NaN	NaN	NaN	NaN	NaN	NaN	NaN	NaN	NaN
NaN	20,7848	20,7815	NaN	NaN	NaN	NaN	NaN	NaN
20,992	NaN	NaN	NaN	NaN	NaN	NaN	NaN	NaN
21,3358	21,2037	21,4196	20,9914	20,9373	21,2936	21,2659	22,2631	22,2926
NaN	NaN	NaN	NaN	NaN	NaN	NaN	NaN	NaN
NaN	NaN	NaN	NaN	NaN	NaN	NaN	NaN	NaN
NaN	NaN	NaN	NaN	NaN	NaN	NaN	NaN	NaN
NaN	NaN	NaN	NaN	NaN	NaN	NaN	NaN	NaN
NaN	NaN	20,4337	NaN	NaN	NaN	NaN	NaN	NaN
NaN	NaN	19,7407	NaN	NaN	NaN	NaN	NaN	NaN
21,2886	22,6037	22,4789	21,3778	21,5759	21,6741	NaN	NaN	NaN

NaN	20,0534	20,3397	NaN	NaN	NaN	NaN	NaN	NaN
NaN	22,7765	23,1801	NaN	NaN	NaN	NaN	NaN	NaN
NaN	NaN	NaN	NaN	NaN	NaN	NaN	NaN	NaN
NaN	NaN	NaN	NaN	NaN	NaN	NaN	NaN	NaN
23,0259	NaN	23,4022	NaN	22,1826	NaN	NaN	NaN	23,3878
NaN	NaN	20,141	20,0372	NaN	NaN	NaN	NaN	NaN
NaN	NaN	NaN	NaN	NaN	NaN	NaN	20,142	NaN
NaN	NaN	NaN	NaN	NaN	NaN	NaN	NaN	NaN
NaN	NaN	20,9254	NaN	NaN	NaN	NaN	NaN	NaN
NaN	NaN	NaN	NaN	NaN	NaN	NaN	NaN	NaN
NaN	NaN	NaN	NaN	NaN	NaN	NaN	NaN	NaN
NaN	NaN	NaN	NaN	NaN	NaN	NaN	NaN	NaN
NaN	NaN	NaN	NaN	NaN	NaN	NaN	NaN	NaN
NaN	NaN	NaN	NaN	NaN	NaN	NaN	NaN	NaN
NaN	NaN	NaN	NaN	NaN	NaN	NaN	NaN	NaN
NaN	NaN	NaN	NaN	NaN	NaN	NaN	NaN	NaN
NaN	NaN	NaN	NaN	NaN	NaN	NaN	NaN	NaN
NaN	NaN	NaN	NaN	NaN	21,4394	21,737	NaN	NaN
NaN	NaN	NaN	20,8548	NaN	NaN	20,7766	NaN	NaN
NaN	NaN	NaN	NaN	NaN	NaN	NaN	NaN	NaN
NaN	NaN	NaN	NaN	NaN	NaN	NaN	NaN	NaN
NaN	NaN	NaN	NaN	NaN	NaN	NaN	NaN	NaN
NaN	NaN	NaN	NaN	NaN	NaN	NaN	NaN	NaN
NaN	NaN	NaN	NaN	NaN	NaN	NaN	NaN	NaN
NaN	NaN	NaN	NaN	NaN	NaN	NaN	NaN	NaN
NaN	NaN	NaN	NaN	NaN	NaN	NaN	NaN	NaN
NaN	NaN	NaN	NaN	NaN	NaN	NaN	NaN	NaN
NaN	NaN	NaN	NaN	NaN	NaN	NaN	NaN	NaN
NaN	NaN	NaN	NaN	NaN	NaN	NaN	NaN	NaN
NaN	NaN	NaN	NaN	NaN	NaN	NaN	NaN	NaN
NaN	21,3436	21,2633	NaN	NaN	NaN	NaN	NaN	NaN
NaN	NaN	NaN	NaN	NaN	NaN	NaN	NaN	NaN
20,5047	20,2197	20,4043	NaN	NaN	22,0943	NaN	NaN	NaN
NaN	NaN	NaN	NaN	NaN	NaN	NaN	NaN	NaN
20,4072	18,9451	NaN	NaN	NaN	NaN	NaN	NaN	NaN
NaN	NaN	NaN	NaN	NaN	NaN	NaN	NaN	NaN
21,1259	21,4563	21,4766	22,8172	22,8396	22,385	22,7567	21,5307	21,7332
NaN	NaN	NaN	20,4765	NaN	19,4761	NaN	NaN	NaN
NaN	NaN	NaN	NaN	NaN	NaN	NaN	NaN	NaN
NaN	NaN	NaN	NaN	NaN	NaN	NaN	NaN	NaN
NaN	NaN	20,149	NaN	NaN	NaN	NaN	NaN	21,4377
NaN	NaN	NaN	NaN	NaN	NaN	NaN	NaN	NaN
NaN	20,984	20,4423	NaN	NaN	NaN	NaN	NaN	NaN
NaN	NaN	NaN	NaN	NaN	20,3428	19,8105	NaN	NaN
NaN	NaN	NaN	NaN	NaN	NaN	NaN	NaN	NaN
NaN	NaN	NaN	NaN	NaN	NaN	NaN	NaN	NaN
23,4339	22,5745	22,4631	24,8833	24,757	24,0795	23,9842	23,6799	23,6291
20,5983	19,8988	20,1957	22,8503	22,992	21,4583	21,899	22,0972	22,1396
NaN	NaN	NaN	21,696	NaN	20,829	NaN	NaN	NaN
22,1577	NaN	21,6834	23,0328	23,115	22,2313	23,7031	21,9361	21,8436
20,1071	NaN	NaN	19,6584	22,0231	NaN	20,9405	22,092	NaN
NaN	NaN	NaN	NaN	21,4302	NaN	NaN	NaN	NaN

NaN	NaN	NaN	NaN	NaN	NaN	NaN	NaN	NaN
NaN	NaN	NaN	NaN	NaN	NaN	NaN	NaN	NaN
NaN	NaN	NaN	NaN	20,1488	NaN	NaN	NaN	NaN
NaN	NaN	NaN	NaN	19,6433	NaN	NaN	NaN	NaN
NaN	NaN	NaN	NaN	NaN	NaN	NaN	NaN	NaN
25,7921	25,1126	25,1442	27,9545	27,912	27,0064	27,0951	26,2907	26,3815
21,1204	NaN	NaN	NaN	NaN	NaN	NaN	NaN	NaN
NaN	NaN	NaN	NaN	NaN	NaN	NaN	NaN	NaN
NaN	NaN	20,8675	NaN	NaN	NaN	NaN	NaN	NaN
NaN	NaN	NaN	NaN	NaN	NaN	NaN	NaN	NaN
NaN	NaN	NaN	NaN	NaN	NaN	NaN	NaN	NaN
NaN	NaN	NaN	NaN	NaN	NaN	NaN	NaN	NaN
NaN	NaN	NaN	NaN	NaN	NaN	NaN	NaN	NaN
20,3467	NaN	NaN	NaN	20,5838	21,0469	20,7163	NaN	NaN
23,5717	NaN	20,8653	23,4381	23,5837	23,2999	22,9692	22,3455	21,9437
NaN	NaN	NaN	NaN	NaN	20,8348	20,7208	NaN	NaN
21,1822	20,7815	20,7983	21,1702	21,4174	NaN	21,2566	20,8193	21,1411
NaN	NaN	NaN	NaN	NaN	NaN	NaN	NaN	NaN
NaN	NaN	NaN	23,2007	23,5912	NaN	NaN	NaN	NaN
NaN	NaN	NaN	NaN	NaN	NaN	NaN	NaN	NaN
NaN	NaN	NaN	NaN	NaN	NaN	NaN	NaN	NaN
22,6293	21,9604	NaN	23,3781	22,9276	23,1706	22,97	NaN	22,5722
NaN	NaN	NaN	NaN	21,3662	NaN	NaN	NaN	NaN
21,661	NaN	NaN	24,1066	24,0312	21,9156	22,1904	NaN	NaN
NaN	NaN	NaN	21,3473	21,5617	NaN	NaN	NaN	NaN
NaN	NaN	NaN	NaN	NaN	20,8363	20,9045	NaN	NaN
NaN	NaN	NaN	NaN	19,3519	NaN	NaN	NaN	NaN
NaN	NaN	NaN	21,7356	21,5677	NaN	NaN	NaN	NaN
21,6799	NaN	NaN	NaN	NaN	19,5246	19,7021	19,972	NaN
NaN	NaN	NaN	NaN	NaN	NaN	NaN	NaN	NaN
NaN	NaN	NaN	NaN	NaN	NaN	NaN	NaN	NaN
NaN	NaN	NaN	NaN	NaN	NaN	NaN	NaN	NaN
NaN	NaN	NaN	NaN	NaN	NaN	NaN	NaN	NaN
NaN	NaN	20,4565	NaN	NaN	NaN	NaN	NaN	NaN
NaN	NaN	NaN	NaN	NaN	NaN	NaN	NaN	NaN
NaN	NaN	NaN	NaN	NaN	NaN	NaN	NaN	NaN
NaN	NaN	NaN	NaN	NaN	NaN	NaN	NaN	NaN
20,29	NaN	NaN	NaN	NaN	NaN	NaN	NaN	NaN
NaN	NaN	NaN	NaN	NaN	NaN	NaN	NaN	NaN
NaN	NaN	NaN	NaN	NaN	NaN	NaN	NaN	NaN
NaN	NaN	NaN	NaN	21,392	NaN	NaN	NaN	NaN
NaN	20,5311	20,4893	NaN	NaN	NaN	NaN	NaN	NaN
NaN	20,3831	NaN	NaN	NaN	NaN	NaN	NaN	NaN
NaN	NaN	NaN	NaN	NaN	NaN	NaN	NaN	NaN
NaN	NaN	NaN	NaN	NaN	19,8033	NaN	NaN	NaN
NaN	NaN	NaN	NaN	NaN	NaN	NaN	NaN	NaN
NaN	NaN	NaN	NaN	NaN	NaN	NaN	NaN	NaN
NaN	23,7088	23,5584	NaN	NaN	NaN	NaN	NaN	NaN
NaN	NaN	NaN	NaN	NaN	NaN	NaN	NaN	NaN
NaN	NaN	NaN	20,2385	19,9818	19,959	20,1495	NaN	NaN
NaN	NaN	NaN	NaN	21,8681	NaN	NaN	NaN	NaN
NaN	NaN	NaN	NaN	NaN	NaN	NaN	NaN	NaN

NaN	NaN	NaN	NaN	NaN	NaN	NaN	NaN	NaN
NaN	NaN	NaN	NaN	NaN	NaN	NaN	NaN	NaN
NaN	NaN	NaN	NaN	NaN	NaN	NaN	NaN	NaN
NaN	NaN	19,3064	NaN	NaN	NaN	NaN	NaN	NaN
NaN	NaN	NaN	NaN	NaN	NaN	NaN	NaN	NaN
NaN	NaN	NaN	NaN	NaN	NaN	NaN	NaN	NaN
NaN	NaN	NaN	NaN	NaN	NaN	NaN	NaN	NaN
NaN	NaN	NaN	NaN	NaN	NaN	NaN	NaN	NaN
NaN	NaN	20,3018	NaN	NaN	NaN	NaN	NaN	NaN
NaN	NaN	NaN	NaN	NaN	NaN	NaN	NaN	NaN
NaN	NaN	NaN	NaN	NaN	NaN	NaN	NaN	NaN
NaN	20,5203	20,4513	NaN	NaN	NaN	NaN	NaN	NaN
NaN	NaN	NaN	NaN	NaN	NaN	NaN	NaN	NaN
NaN	NaN	NaN	NaN	NaN	NaN	NaN	NaN	NaN
NaN	NaN	21,7097	NaN	NaN	NaN	NaN	NaN	NaN
NaN	18,2097	NaN	NaN	NaN	NaN	NaN	NaN	NaN
NaN	NaN	NaN	23,4039	23,1706	NaN	NaN	NaN	NaN
NaN	NaN	NaN	NaN	NaN	NaN	NaN	NaN	NaN
NaN	NaN	NaN	NaN	NaN	NaN	NaN	NaN	NaN
NaN	20,2589	NaN	20,1019	20,0642	20,179	20,1715	NaN	NaN
NaN	NaN	NaN	NaN	NaN	25,4874	25,5019	NaN	NaN
NaN	NaN	NaN	NaN	NaN	NaN	NaN	NaN	NaN
NaN	23,0956	23,1856	NaN	NaN	NaN	NaN	NaN	NaN
NaN	27,5758	27,6135	NaN	NaN	NaN	NaN	NaN	NaN
NaN	NaN	NaN	NaN	NaN	NaN	NaN	NaN	NaN
NaN	21,8554	21,9042	NaN	NaN	NaN	NaN	NaN	NaN
23,5894	NaN	24,0554	25,211	25,137	23,5427	23,9602	26,9008	26,7451
NaN	NaN	NaN	NaN	NaN	20,4315	20,7915	NaN	NaN
NaN	NaN	NaN	21,7115	NaN	21,6287	21,8328	NaN	NaN
21,1683	NaN	NaN	21,7304	NaN	20,5151	NaN	NaN	21,4327
NaN	NaN	NaN	NaN	NaN	NaN	NaN	NaN	NaN
NaN	NaN	NaN	NaN	NaN	NaN	NaN	NaN	NaN
NaN	NaN	NaN	NaN	NaN	NaN	NaN	NaN	NaN
NaN	NaN	NaN	NaN	NaN	NaN	NaN	NaN	NaN
NaN	NaN	NaN	NaN	NaN	NaN	19,9229	NaN	NaN
NaN	NaN	NaN	NaN	NaN	NaN	NaN	NaN	NaN
NaN	NaN	NaN	NaN	22,7661	23,438	23,2988	NaN	NaN
NaN	NaN	NaN	NaN	NaN	NaN	NaN	NaN	NaN
NaN	NaN	NaN	NaN	NaN	NaN	NaN	NaN	NaN
NaN	21,0265	21,4843	NaN	NaN	NaN	NaN	NaN	NaN
20,0099	22,4349	22,6658	22,1729	22,2573	NaN	NaN	25,1395	25,3901
NaN	NaN	NaN	NaN	NaN	NaN	NaN	NaN	NaN
NaN	NaN	NaN	NaN	NaN	NaN	NaN	NaN	NaN
NaN	NaN	NaN	NaN	NaN	NaN	NaN	NaN	NaN
NaN	NaN	NaN	NaN	NaN	NaN	20,0834	NaN	NaN
NaN	NaN	NaN	NaN	NaN	NaN	NaN	NaN	NaN
NaN	20,4986	21,1504	NaN	NaN	NaN	NaN	NaN	NaN
NaN	NaN	NaN	NaN	NaN	NaN	NaN	NaN	NaN
NaN	NaN	NaN	NaN	NaN	NaN	NaN	NaN	NaN
NaN	NaN	NaN	NaN	NaN	NaN	NaN	NaN	NaN
NaN	NaN	NaN	NaN	NaN	NaN	NaN	NaN	NaN
NaN	NaN	NaN	NaN	NaN	NaN	NaN	NaN	NaN

20,628	NaN	NaN	21,4571	21,3183	21,5356	21,3678	NaN	NaN
NaN	NaN	NaN	NaN	NaN	NaN	NaN	NaN	NaN
NaN	24,4172	24,152	NaN	NaN	NaN	NaN	27,153	26,9487
30,6293	29,6554	29,6804	25,1454	24,7829	30,1912	30,1951	32,2835	32,2888
NaN	NaN	NaN	NaN	NaN	20,6477	20,386	NaN	NaN
22,9062	NaN	NaN	21,8781	22,0818	NaN	NaN	NaN	NaN
NaN	NaN	NaN	NaN	NaN	NaN	NaN	NaN	NaN
NaN	NaN	NaN	NaN	NaN	NaN	NaN	NaN	NaN
NaN	NaN	19,6278	NaN	NaN	NaN	NaN	NaN	NaN
NaN	21,2676	21,3113	NaN	NaN	NaN	NaN	NaN	NaN
NaN	NaN	NaN	NaN	NaN	NaN	NaN	NaN	NaN
20,0628	20,1575	NaN	NaN	NaN	NaN	20,6847	20,629	21,3141
23,7293	22,9138	23,0675	24,6302	24,6509	25,0156	25,0311	24,3882	24,5607
NaN	20,4922	NaN	19,8007	NaN	19,9225	20,2799	21,6646	NaN
NaN	NaN	NaN	NaN	NaN	NaN	NaN	NaN	21,9091
27,9939	24,2533	24,1556	24,8558	24,7981	24,1661	23,9445	24,7531	24,8075
NaN	22,9479	22,9462	23,0904	23,3044	23,9713	23,9195	NaN	NaN
NaN	NaN	NaN	NaN	NaN	NaN	NaN	NaN	NaN
NaN	20,6314	20,7863	NaN	NaN	NaN	NaN	NaN	NaN
20,6639	NaN	NaN	20,8849	21,2814	20,7083	21,0538	20,5054	20,8832
NaN	NaN	NaN	NaN	NaN	NaN	NaN	NaN	NaN
NaN	NaN	NaN	NaN	NaN	NaN	NaN	NaN	NaN
NaN	NaN	NaN	NaN	NaN	NaN	NaN	NaN	NaN
NaN	NaN	NaN	NaN	NaN	NaN	NaN	NaN	NaN
NaN	NaN	20,6675	NaN	NaN	NaN	NaN	NaN	NaN
NaN	20,1618	19,9545	NaN	NaN	NaN	NaN	NaN	NaN
NaN	NaN	NaN	NaN	NaN	NaN	NaN	NaN	NaN
NaN	21,7607	21,8737	NaN	NaN	NaN	NaN	NaN	NaN
NaN	NaN	NaN	NaN	NaN	NaN	NaN	NaN	NaN
NaN	NaN	NaN	NaN	NaN	NaN	NaN	NaN	NaN
NaN	NaN	NaN	NaN	NaN	NaN	NaN	NaN	NaN
NaN	NaN	NaN	NaN	NaN	NaN	NaN	NaN	NaN
25,9501	25,1336	25,2706	26,926	26,8708	26,5407	26,8368	NaN	NaN
NaN	NaN	NaN	NaN	NaN	NaN	NaN	NaN	NaN
21,3075	NaN	21,3516	NaN	22,2922	22,8986	22,9122	NaN	NaN
NaN	NaN	NaN	NaN	NaN	NaN	NaN	NaN	NaN
NaN	NaN	NaN	NaN	NaN	NaN	NaN	NaN	NaN
NaN	NaN	NaN	NaN	NaN	NaN	NaN	NaN	NaN
NaN	NaN	NaN	20,4886	20,6796	NaN	19,4398	NaN	NaN
NaN	NaN	NaN	NaN	NaN	NaN	NaN	NaN	NaN
NaN	NaN	NaN	NaN	NaN	NaN	NaN	NaN	NaN
22,6067	21,5106	22,3008	22,9277	23,0598	23,1895	23,2842	NaN	NaN
25,8783	NaN	NaN	25,6955	26,0821	26,2821	26,2239	NaN	NaN
NaN	NaN	NaN	NaN	NaN	NaN	NaN	NaN	NaN
NaN	NaN	NaN	NaN	NaN	NaN	NaN	NaN	NaN
NaN	21,5358	21,428	NaN	NaN	NaN	NaN	NaN	NaN
24,8287	24,0985	24,8771	25,7042	25,7955	26,1131	26,1621	NaN	NaN
NaN	NaN	NaN	NaN	NaN	NaN	NaN	NaN	NaN
NaN	NaN	NaN	NaN	NaN	NaN	NaN	NaN	NaN
NaN	NaN	NaN	21,7673	21,679	NaN	21,523	21,7725	NaN
NaN	NaN	NaN	NaN	NaN	NaN	NaN	NaN	NaN
NaN	NaN	NaN	NaN	NaN	NaN	NaN	NaN	NaN

NaN	NaN	NaN	20,4508	NaN	NaN	NaN	NaN	NaN
NaN	NaN	NaN	22,4869	22,4655	22,0027	21,9709	22,2485	NaN
21,9224	NaN	NaN	22,2257	22,0305	22,239	22,4224	21,8167	21,6702
21,6016	20,7862	20,476	NaN	NaN	NaN	NaN	NaN	NaN
NaN	19,8374	NaN	NaN	NaN	NaN	NaN	NaN	NaN
NaN	NaN	NaN	NaN	NaN	NaN	NaN	NaN	NaN
25,4326	NaN	NaN	NaN	NaN	NaN	NaN	20,7581	20,377
21,571	21,1331	21,2083	21,7379	21,8645	21,5064	21,8971	21,5689	21,7399
NaN	22,3583	22,7814	NaN	NaN	NaN	NaN	NaN	NaN
NaN	NaN	NaN	NaN	NaN	NaN	20,0709	NaN	NaN
NaN	NaN	NaN	NaN	NaN	18,1672	NaN	NaN	NaN
25,4561	24,7887	24,6766	27,1701	27,1326	26,8027	26,8137	25,5549	25,5321
NaN	NaN	NaN	NaN	NaN	NaN	NaN	NaN	NaN
22,3441	NaN	NaN	22,1139	21,6864	NaN	NaN	NaN	NaN
NaN	NaN	NaN	NaN	NaN	NaN	NaN	NaN	NaN
NaN	21,0549	21,171	NaN	NaN	NaN	NaN	NaN	NaN
24,0988	23,5523	23,514	NaN	NaN	NaN	NaN	24,1662	24,2821

LFQ intensi	LFQ intensi	LFQ intensi	LFQ intensi	LFQ intensi	LFQ intensi	LFQ intensi	LFQ intensi	LFQ intensi
32,1457	32,1251	32,2037	32,1912	33,3509	33,3362	33,5302	33,5863	31,2677
33,4815	33,449	33,9359	33,8994	34,7854	34,7841	34,3176	34,2963	33,9976
28,5903	28,5968	28,2119	28,1974	26,4131	26,5327	26,4021	26,3685	26,6613
17,9417	19,5194	20,9491	20,0603	18,2954	17,5693	NaN	NaN	21,2293
26,5213	26,4093	26,6262	26,5353	27,2626	27,3121	26,6164	26,7883	26,7436
24,4073	24,5081	26,5273	26,4786	24,5021	24,5221	26,3633	26,4031	25,9438
24,2564	24,0392	24,2343	24,2003	20,257	NaN	20,049	NaN	23,8538
27,6116	27,5913	27,972	28,0287	29,2003	29,2366	28,1618	28,1337	28,9418
29,0506	29,0879	29,0028	29,0074	29,7136	29,7652	29,4623	29,4764	29,214
27,2891	27,3356	27,8362	27,8418	29,3968	29,4207	28,3259	28,2731	28,2347
17,2848	NaN	20,4204	17,7762	NaN	NaN	NaN	NaN	20,1807
34,9827	34,9943	35,2256	35,2067	35,9901	36,0037	34,6261	34,5796	34,4214
31,9634	31,9671	32,0969	32,0904	32,7093	32,7006	32,4375	32,485	31,4422
27,9422	28,0224	28,9789	28,9742	29,6195	29,6824	27,4398	27,4534	29,4591
25,8143	25,8071	26,0098	26,0105	24,874	24,8491	26,472	26,3887	26,8123
28,8619	28,7927	28,1491	28,0654	26,5949	26,5721	26,6375	26,4908	28,1859
27,2627	27,3071	27,4572	27,5251	26,6974	26,6866	27,5743	27,6371	27,6255
26,8985	26,9159	28,0873	28,1234	25,6604	25,785	25,3598	25,388	24,4788
25,8484	25,807	26,8309	26,8517	24,8849	25,1336	24,3144	24,3744	25,8418
23,0324	23,0147	24,0984	24,1794	21,3247	20,664	24,7827	24,7852	24,0531
27,1706	27,2874	28,4635	28,439	26,0359	26,0635	25,2894	25,2589	25,8879
25,7392	25,6754	25,6524	25,6644	24,5795	24,6553	25,7923	25,747	25,9867
28,1173	28,0704	27,8194	27,734	28,6911	28,6253	27,636	27,5721	27,52
26,5413	26,4478	26,3532	26,4099	25,533	25,5185	26,7558	26,7078	26,4615
26,3389	26,3577	27,6952	27,7353	28,1686	28,276	25,4034	25,4769	28,1631
25,5955	25,6	26,0743	26,0516	26,2088	26,0665	25,4462	25,3596	25,4016
25,5902	25,4619	26,5921	26,4934	27,4185	27,4991	27,0291	26,9793	27,0834
23,4383	23,2092	23,853	23,737	22,363	22,3939	23,9318	23,9057	24,1774
25,6382	25,7494	27,8656	27,9108	26,23	26,2137	26,9271	26,7745	26,5658
24,9495	24,9819	25,0692	25,1795	25,0733	25,631	24,0469	24,0144	25,2533
26,4482	26,4037	26,861	26,87	25,4836	25,3786	26,5793	26,4976	26,2174
29,6474	29,6718	27,9884	27,9909	28,0597	27,9896	25,8631	25,9939	28,8196
26,4052	26,4922	26,5785	26,7103	25,9415	25,864	26,5326	26,4568	26,9749
26,2082	26,1889	25,715	25,7572	23,4929	23,9805	25,0867	24,9924	26,0658
22,5608	22,7853	22,9055	22,3368	21,7059	NaN	24,1995	24,055	23,7303
27,5775	27,5877	27,1376	27,208	26,5687	26,6455	27,3162	27,2762	26,7754
29,2054	29,274	28,9368	28,9351	28,2192	28,1405	28,8157	28,8259	28,9083
25,5941	25,5707	26,8231	26,77	25,9918	25,8469	26,8082	26,9054	25,7211
25,8383	25,9014	25,7792	25,7076	26,4275	26,3105	26,4831	26,464	26,0742
27,3354	27,3399	27,2189	27,3634	26,1354	26,1508	27,5294	27,5113	27,2887
26,5554	26,6816	27,3349	27,3481	28,3543	28,3695	27,1517	27,1375	28,5097
27,182	27,1765	26,7412	26,7543	25,6533	25,6786	25,6394	25,5327	26,7722
26,5274	26,5268	27,6813	27,7142	26,7519	26,8212	26,2109	26,1457	27,0103
26,3741	26,3747	26,3997	26,4461	25,6105	25,6341	26,405	26,496	26,4197
25,8357	25,8427	24,6375	24,6282	24,8771	24,8225	25,1293	25,0454	25,5072
26,7917	26,7803	27,0363	27,0704	26,4998	26,6421	27,4754	27,4587	27,3957
26,3139	26,3334	25,6568	25,6783	24,7497	24,8158	24,5213	24,5394	25,7343
23,2361	23,246	24,4148	24,4538	21,778	22,4112	23,6003	23,8307	23,5711
29,6701	29,6671	28,8877	28,9218	30,4667	30,4734	29,2395	29,2656	28,1413
16,6161	18,3336	20,2731	19,4303	NaN	NaN	NaN	NaN	16,6809
24,5748	24,6916	24,3592	24,1065	25,5377	25,4904	24,0841	24,2115	24,2869

26,7274	26,7371	26,622	26,7095	25,7022	25,7993	26,8719	27,0224	27,0251
32,7271	32,8223	32,277	32,3671	33,277	33,2289	33,1952	33,1875	32,2333
21,5009	21,6129	22,5742	22,8015	22,6618	22,4494	18,02	17,318	21,1272
26,1938	26,1485	25,9183	25,9812	24,714	24,8554	26,2697	26,1308	25,6558
NaN	NaN	NaN	NaN	NaN	NaN	NaN	NaN	NaN
26,3611	26,3872	25,9233	25,9788	25,2879	25,1683	25,6036	25,7101	26,2538
24,9772	25,1315	23,0116	23,1528	23,1012	23,1538	23,2953	23,1892	24,5906
26,7273	26,7349	26,8152	26,8202	25,9876	25,992	26,4916	26,5438	27,1145
27,3095	27,3292	28,2135	28,1716	26,0922	26,1947	27,3257	27,2333	26,9483
25,4051	25,3655	24,4423	24,4865	23,3991	23,3365	22,7278	22,5419	24,5095
24,0441	23,9087	26,3613	26,2417	20,3677	20,9077	25,4943	25,5797	24,9245
25,523	25,5444	24,6197	24,5595	23,9003	23,8013	23,9029	24,2432	24,4694
26,5271	26,5105	26,9339	26,8644	27,9532	27,9932	27,199	27,233	27,5811
24,8227	24,7663	23,224	23,488	23,7104	23,8608	24,8674	24,8132	25,1727
23,5621	23,4149	23,2663	22,8968	25,1074	24,9883	24,0148	24,0403	23,4551
23,6273	23,6989	24,2483	24,3826	24,2822	24,0549	23,4047	23,2698	24,2888
27,5885	27,5379	26,7288	26,775	26,638	26,5616	26,7739	26,8579	27,6769
23,0066	22,5082	22,0046	22,1895	21,0542	21,599	22,5382	22,6431	23,4793
30,1838	30,103	30,6611	30,6271	29,4706	29,5532	31,0773	31,0738	30,8982
24,231	24,137	24,2072	24,2602	23,052	23,3462	23,5326	23,6241	24,2465
23,3807	23,3232	23,8536	23,8411	22,3976	22,3542	23,353	23,3596	23,6935
24,1117	24,1025	24,7284	24,6465	23,1618	23,2221	25,286	25,1382	25,4653
24,6669	24,7076	25,1726	25,1397	23,886	23,9667	25,6208	25,5595	25,0777
28,7668	28,6944	28,053	28,1718	29,236	29,2468	28,2439	28,237	27,9106
23,8845	24,0815	24,0556	24,1539	24,4774	24,6971	23,8873	24,002	23,6237
29,202	29,2227	28,8043	28,8949	26,052	26,0449	26,2505	26,3848	28,8561
24,0582	24,2453	24,3518	24,3054	22,7568	23,2446	24,6507	24,6145	24,5202
24,2026	24,1908	23,9009	24,3191	23,9895	23,7619	24,8014	24,7404	24,9175
27,6328	27,6554	27,34	27,4311	26,5124	26,5286	26,8224	26,8294	27,6226
25,6334	25,6532	26,1111	26,1346	26,3073	26,271	26,0176	26,103	25,7933
27,6344	27,6644	25,7465	25,8001	26,3617	26,1698	27,1212	27,1313	26,5773
17,0143	17,7315	18,777	18,2873	18,2528	18,6105	17,5927	NaN	17,2991
21,6778	22,1011	NaN	20,6358	23,8277	23,9758	23,2676	23,4394	22,4437
25,9044	25,9137	25,9672	25,8964	26,6306	26,6724	26,7879	26,7548	26,1766
23,6317	23,9502	23,238	23,3594	24,947	24,8975	23,8719	23,5665	23,3865
21,6976	21,1296	20,8793	21,1234	22,474	22,9086	22,7277	22,5402	21,1057
28,5787	28,6658	28,3525	28,3395	27,4455	27,3253	28,5648	28,6727	28,601
24,2921	24,5333	25,6985	25,6648	24,6054	24,631	23,957	23,9278	25,1043
27,5443	27,6355	28,706	28,7615	29,0016	29,1534	28,1194	28,0702	27,0222
22,9267	23,2785	23,9949	23,9249	21,8304	21,6654	22,5507	22,6225	21,8309
28,7793	28,7196	29,2575	29,1047	29,7942	29,9756	29,0872	29,0332	28,629
NaN	NaN	19,489	19,4607	NaN	NaN	NaN	20,4714	20,9087
25,7677	25,7797	25,2889	25,1995	25,6774	25,6489	24,7988	24,8221	25,0531
22,8658	22,7549	22,9903	22,8789	22,607	22,6769	23,8134	23,7643	23,6392
25,7046	25,71	25,3753	25,4545	26,4824	26,3985	25,8375	25,7128	25,9347
26,4521	26,4319	27,0766	27,1195	26,4054	26,3295	25,5826	25,5944	26,4387
25,616	25,7213	26,3734	26,3861	26,8496	27,037	27,8355	27,9427	27,4297
26,1453	26,1413	25,5581	25,6121	26,9767	26,9461	26,3503	26,4485	26,097
27,8975	27,9087	26,8985	26,8498	26,7428	26,7103	26,9897	27,0544	28,1582
31,2835	31,316	30,7769	30,8077	31,9641	31,8841	31,5377	31,5796	30,9508
32,5728	32,5685	31,5806	31,5391	32,9269	32,9114	31,9506	32,0568	32,7766
27,4859	27,4583	27,4555	27,5854	26,0122	26,0984	25,8065	25,5228	27,6744

24,1356	24,1631	23,6767	23,773	23,2723	23,3192	23,552	23,3966	23,8627
18,8435	17,8521	NaN	NaN	NaN	NaN	NaN	NaN	NaN
22,2366	22,1878	24,8523	24,8314	21,7999	21,2461	23,9939	24,1216	19,7371
23,8502	23,7926	23,658	23,7642	23,0981	23,1574	24,2752	24,5265	24,3452
26,2271	26,1649	24,5802	24,8032	24,7851	24,6361	24,5183	24,5592	26,1322
25,3913	25,3372	25,6193	25,5766	24,6056	24,6756	26,3428	26,2467	25,9723
23,9608	23,9491	23,6829	23,5446	22,6217	22,907	24,0793	24,0555	24,3996
25,5107	25,5438	24,9829	24,9463	26,232	26,3903	24,8167	24,6545	24,0336
26,9686	26,9801	26,5545	26,5253	25,8172	25,6862	26,5037	26,4642	26,6133
28,4588	28,4572	26,5871	26,6979	28,5153	28,4645	27,7386	27,8686	28,9597
NaN	NaN	NaN	NaN	NaN	NaN	NaN	NaN	NaN
23,1251	23,0826	22,9066	22,8114	24,7076	24,6124	24,1615	24,1536	22,7943
27,3659	27,2976	27,0784	27,0212	26,3343	26,2048	26,4328	26,537	26,8894
26,0946	26,0889	26,3538	26,3369	25,4038	25,4254	24,3158	24,3601	25,5807
24,2577	24,129	24,2065	24,238	23,6635	23,7577	24,5546	24,5394	24,7248
26,5663	26,5243	26,6435	26,6132	27,1361	27,1157	26,2219	26,1557	26,0076
22,5092	22,5589	23,0899	23,1752	21,2034	21,3755	21,6476	21,4522	22,7681
24,4804	24,5369	22,3696	22,0561	22,4814	22,7457	21,2707	20,2919	24,258
22,0832	22,3679	22,4776	22,5075	22,8964	22,8641	22,5847	22,4928	22,6343
22,4329	22,0668	23,9123	24,156	20,8458	20,3541	23,2984	23,5209	23,1202
22,5359	22,6843	22,2668	22,5186	20,9342	22,0002	23,0674	22,8295	23,4458
24,7621	24,8156	23,9046	24,0056	25,0518	24,824	24,1239	24,0951	24,4218
NaN	NaN	18,9316	NaN	NaN	NaN	NaN	NaN	NaN
28,21	28,1825	27,9578	28,0432	27,2594	27,2366	25,8189	25,8551	28,2823
NaN	21,1885	23,2727	23,085	22,1323	21,9062	22,2845	21,966	22,4675
26,031	26,0106	24,2883	24,2302	24,2446	24,1021	23,7717	23,5949	25,4824
26,6079	26,6618	27,0288	26,9657	26,0863	25,9198	27,6378	27,6235	27,4724
NaN	NaN	22,5185	22,5648	NaN	NaN	NaN	NaN	NaN
24,3948	24,4832	22,8812	22,7112	21,9656	22,3121	21,266	21,5254	24,0716
22,1822	22,1325	23,9984	23,8444	23,2128	23,0604	22,3214	20,4505	23,8059
25,3473	25,3578	23,8567	24,0295	23,4902	23,7697	23,7091	23,7881	24,6087
29,781	29,8572	29,42	29,4621	30,1923	30,1287	30,2463	30,1621	29,7163
24,4063	24,4839	24,6428	24,6149	25,2746	25,272	25,1905	25,2419	24,8874
25,3994	25,418	25,6841	25,6781	24,8395	24,6678	25,8551	25,7359	25,6096
26,2304	26,3114	24,7494	24,8328	24,1975	24,2953	24,1962	24,307	25,4777
22,326	21,7897	21,6223	22,1541	22,5642	22,5283	23,2082	23,2006	22,6505
26,1813	26,1715	24,6172	24,4135	24,1326	24,1562	23,6584	23,7227	25,5914
29,6073	29,6181	29,5212	29,6249	30,9445	30,9825	29,4166	29,4335	28,5144
29,0626	28,9999	28,6363	28,5943	30,1831	30,1853	29,9786	29,9617	27,9923
NaN	NaN	NaN	NaN	NaN	NaN	NaN	NaN	NaN
23,7919	23,8246	23,6431	23,6797	25,3469	25,3365	25,0206	24,9339	22,691
26,8401	26,8142	25,4052	25,4363	27,0749	27,2189	27,4959	27,4348	24,6132
22,6506	22,7166	23,7925	23,6141	24,8117	24,7128	23,5844	23,1869	22,4155
24,0255	23,9321	23,7497	23,8645	22,4671	22,8735	24,12	24,1628	24,2372
24,6794	24,4448	24,4671	24,3254	22,9771	23,0028	24,5739	24,5451	24,4934
32,1711	32,281	30,0947	30,0268	31,5645	31,2385	30,8849	30,934	31,8356
23,8094	23,8357	25,3886	25,2712	22,7328	22,7365	25,2116	25,2513	24,2934
25,4421	24,9448	NaN	20,1138	21,1083	21,0871	22,1441	21,6402	24,3904
23,4039	23,2753	24,5209	24,6909	NaN	21,6484	23,4837	23,5191	23,7282
23,161	23,6456	21,637	21,4273	22,103	22,4097	22,341	NaN	22,7668
26,3062	26,2465	24,8229	24,7762	22,8521	23,2115	22,0008	20,743	24,7684
22,9232	22,8436	22,609	22,2369	21,4416	21,9127	23,0405	23,1454	23,4728

23,2057	23,2112	22,117	21,9651	NaN	NaN	22,2625	22,4343	23,4466
28,4001	28,4461	28,9132	28,9325	30,4197	30,3096	27,3061	27,674	27,4143
19,1034	19,4758	19,9175	21,1433	NaN	20,1471	21,907	21,5964	21,1706
22,9835	22,8366	22,3674	22,1064	23,4012	23,4726	22,9261	22,7416	22,055
22,9307	22,6886	23,7259	23,6288	22,4273	22,5798	22,79	22,1299	22,9642
21,1963	20,6071	21,5923	21,2572	20,6323	NaN	22,3165	22,2566	21,5264
26,9352	27,0403	25,7143	25,8281	29,6825	29,6022	28,5852	28,8377	24,7161
26,8362	26,7357	27,4605	27,4283	26,0172	26,0671	26,4975	26,5069	27,4271
24,5516	24,4011	25,6548	25,6647	23,1577	23,4668	24,6996	24,3984	24,2296
31,5503	31,6492	31,6407	31,6689	31,8579	31,8525	32,1725	32,2718	31,9322
22,9048	22,5444	22,0461	21,8517	23,7907	23,6375	22,5084	22,5051	22,1406
25,1064	25,0838	23,7177	23,7861	22,9319	23,288	22,6407	22,6287	24,7256
29,669	29,7526	28,5565	28,5204	29,565	29,5836	29,0205	29,0766	29,1674
25,8086	25,9248	23,7688	23,7531	24,1272	23,9978	22,9624	23,3553	24,9677
22,5546	22,4974	22,7007	23,1661	22,9429	23,4713	22,5009	22,6373	22,0846
29,6047	29,7031	29,2694	29,5302	30,4772	30,4607	30,1815	30,1037	29,4038
23,9974	24,0101	23,7421	23,8057	23,4025	23,1925	24,2622	24,3427	24,4865
23,2816	22,9701	23,2945	23,3809	21,8977	22,2268	23,0085	22,9968	22,8347
NaN	NaN	NaN	NaN	21,4839	21,5196	21,2835	21,1459	20,6057
NaN	NaN	NaN	19,8133	NaN	NaN	NaN	NaN	NaN
23,2191	23,4957	21,874	22,0877	NaN	NaN	NaN	20,0531	22,2857
22,8273	22,4449	21,57	21,6602	22,0025	21,7207	22,1574	21,7382	22,4105
23,9146	23,9933	23,7824	23,6112	23,2086	23,1383	24,0152	24,0345	24,2141
21,921	21,7078	21,2082	21,321	21,6317	21,4259	21,6672	21,0173	22,1021
22,9852	23,1881	22,8216	22,4395	24,3345	24,3903	24,1124	24,1149	22,5291
24,4164	24,5225	24,6642	24,4595	24,5652	24,6215	24,6984	24,544	24,0447
22,6689	22,6081	23,6765	23,4073	24,1875	24,4074	23,2035	23,2591	23,4223
28,6161	28,6977	29,2189	29,1626	29,766	29,7532	28,0067	27,9483	28,6774
23,7014	23,7814	23,6132	23,6845	23,7185	23,558	24,4729	24,5628	24,6246
22,1442	22,5314	23,4937	23,3047	22,0835	NaN	23,7064	23,4893	23,6168
30,6504	30,7313	26,5953	26,386	27,0692	27,166	31,3164	31,3927	30,084
24,6412	24,7268	24,0577	24,0605	23,4568	23,5544	23,978	24,0321	24,1893
27,5831	27,6644	27,5863	27,7063	26,9859	26,9532	27,7077	27,7778	27,8688
28,0586	28,0766	27,5621	27,5109	26,7763	26,9616	27,0153	26,9148	27,9403
26,1918	26,2896	26,3734	26,3412	25,5292	25,5366	27,1181	27,1762	27,0534
23,4773	23,5127	23,1194	23,1086	22,5194	22,8556	23,673	23,7196	23,7544
22,635	22,5212	23,9077	23,8986	21,8129	21,5164	21,9999	21,6112	23,0203
22,9833	23,058	22,6256	22,401	NaN	21,1499	23,5876	23,5007	23,7628
23,7325	23,9782	22,8283	22,5958	22,0198	22,3321	22,9269	22,8308	23,5458
21,789	21,3223	21,8925	21,9468	22,7403	22,4899	22,5946	22,6362	22,7796
22,8876	22,8975	24,0158	23,8433	23,5564	23,4033	24,3637	24,5421	24,1939
23,026	23,0562	25,0143	24,9653	23,935	23,765	25,8635	25,8324	26,8409
25,9736	26,0214	24,5853	24,6193	24,777	24,8119	24,5361	24,5135	25,4271
27,4789	27,4104	26,7011	26,6788	28,4008	28,2786	27,348	27,4857	26,2822
24,0028	24,0178	23,51	23,6796	22,2021	22,278	21,4944	20,4394	23,1752
NaN	NaN	19,6582	19,7833	NaN	NaN	NaN	NaN	21,5437
23,7872	23,7176	24,1797	24,1407	25,1366	24,9904	24,0246	24,1508	24,3884
23,1979	23,5204	22,7127	22,9317	NaN	21,2879	24,0362	23,6515	23,3917
20,9303	20,2679	21,5061	21,0909	NaN	19,6324	20,9632	20,4076	20,2566
24,8939	24,9461	24,6292	24,6091	25,3824	25,2708	25,2825	25,189	23,7672
24,2978	24,3932	24,3784	24,1911	23,3519	23,325	23,2485	22,9548	24,1512
20,5983	20,7613	22,0451	21,9731	NaN	NaN	21,402	21,6079	23,071

23,7926	23,8243	23,6233	23,7115	22,7983	22,8352	22,3424	22,2251	23,4391
22,2098	22,5702	22,5585	22,3708	21,9791	22,3104	23,1174	23,0792	22,5844
24,6129	24,3872	22,9678	22,924	22,6797	22,5161	22,5351	22,6388	23,7011
22,9517	23,0569	22,5424	22,6865	22,1835	21,4586	23,0646	23,2928	23,5285
21,5803	21,5788	NaN	NaN	22,6797	23,0977	22,1867	22,05	20,6428
21,9838	21,9967	21,9766	22,2185	NaN	NaN	22,4367	22,1693	22,2421
20,6193	21,538	21,4142	21,5848	NaN	NaN	20,0536	NaN	20,5742
22,4438	22,6264	21,9997	21,9954	NaN	NaN	NaN	21,0013	22,6479
31,7556	31,6814	32,8367	32,7184	31,8064	31,8171	30,8521	30,769	28,6959
25,5183	25,549	26,2961	26,2345	26,8729	26,9621	25,626	26,0475	24,9452
22,9358	22,9277	21,2254	21,4723	21,6777	21,9585	22,1412	22,1739	22,3821
22,4682	22,2025	22,0509	22,0115	20,5785	NaN	21,8172	21,8278	22,3642
26,7951	26,8238	26,4853	26,517	25,9194	25,9436	26,6784	26,7655	26,9043
24,8944	24,9104	25,989	25,95	24,1359	24,2994	26,4498	26,3271	26,684
21,3926	22,0751	21,7078	21,6397	NaN	NaN	22,0063	22,2491	21,7808
21,2839	21,5894	21,7983	21,9483	22,5889	22,4597	21,6808	21,9325	22,1928
23,1878	23,3827	23,3941	23,5989	23,3372	23,3558	24,1661	24,0934	23,6127
22,1898	21,8375	21,6584	21,7803	23,8908	23,7284	23,2985	23,3534	22,2185
21,1086	21,1253	20,8913	21,0746	NaN	NaN	21,0296	NaN	20,9279
21,4669	21,685	NaN	NaN	17,5969	17,8869	19,8264	19,712	NaN
24,0315	24,0613	24,9106	24,8886	23,3469	23,668	22,7987	22,6336	23,479
31,033	31,0568	26,4498	26,4908	27,1997	27,33	31,2963	31,1964	30,2635
23,9232	23,963	23,0952	22,9962	24,0162	24,1403	23,3934	23,4081	23,8145
25,9231	26,0257	25,9265	25,9461	24,4511	24,4702	23,9701	23,9348	25,9597
24,0604	23,9749	24,0404	24,0407	23,376	23,2637	24,6244	24,7421	24,8259
17,1884	NaN	NaN	NaN	NaN	17,2471	NaN	NaN	NaN
22,1292	21,9587	20,9158	21,0746	NaN	NaN	20,4227	20,7254	21,6762
25,38	25,3921	25,0272	24,9745	24,4407	24,4056	24,4422	24,6031	25,0918
23,4103	23,4779	23,776	23,799	22,7232	22,7638	23,5707	23,6066	23,6968
23,2288	22,9431	23,6168	23,768	22,0784	22,0015	22,6251	23,0496	23,0117
24,7271	24,7391	23,5833	23,7525	23,2661	23,4578	23,5575	23,4127	24,6737
23,5231	23,4603	22,795	22,561	22,5395	22,498	23,2387	22,9807	23,5711
20,8676	21,3196	21,715	21,6118	22,6125	22,6464	21,3674	22,1314	21,5892
27,9231	28,0085	26,6549	26,6603	26,449	26,4196	26,4254	26,4193	27,3845
22,8537	22,8087	20,7027	20,9633	21,3198	21,6559	21,6707	21,2688	22,6567
22,8465	23,0172	23,4512	23,016	22,3203	22,4984	23,4833	23,6812	23,3952
23,2982	23,3158	20,8525	20,8282	NaN	NaN	23,5392	23,6192	23,1809
22,6877	22,5986	22,4065	22,2272	24,0221	23,8365	22,8141	23,1356	22,8315
23,5773	23,583	23,1737	23,513	22,353	22,3389	23,4051	23,0127	23,5513
23,2082	23,4538	23,9093	23,9713	22,7618	22,6755	23,6687	23,8123	24,0132
20,3354	20,0752	20,5957	20,6818	NaN	NaN	21,2434	21,1799	20,3286
21,8981	21,9754	21,7877	21,521	21,5203	22,2444	21,4638	22,0978	21,8853
24,0017	23,842	22,4095	22,4415	23,2419	23,1584	23,3111	23,3568	23,0546
20,9274	21,9417	22,0913	21,7766	22,9874	23,0055	22,4193	22,5847	22,6558
NaN	NaN	19,0637	19,3782	NaN	NaN	NaN	NaN	18,9823
28,5866	28,4972	28,0621	27,9031	26,6479	26,5712	25,6494	25,695	27,9586
20,7684	NaN	20,9609	20,8067	NaN	NaN	21,2008	21,4244	21,1067
NaN	NaN	21,251	NaN	21,5654	21,4987	20,9494	21,4371	20,8487
29,2818	29,264	26,8092	26,767	29,1344	29,0638	29,4495	29,4776	30,719
27,2115	27,0939	27,0741	27,0053	27,9226	27,9242	27,0556	27,1088	26,5171
22,6249	22,659	21,6326	21,7448	20,01	NaN	21,0934	21,3755	23,237
24,1328	24,2024	23,9457	23,91	23,5181	23,5941	22,2948	22,2216	24,3942

21,8561	21,8438	22,607	22,64	NaN	21,7221	23,2416	23,3905	22,3009
22,1885	21,9749	21,4643	21,7793	20,9062	20,681	21,1005	20,954	22,1783
22,1823	22,4533	22,3077	22,4357	22,5352	22,444	21,5896	21,6726	21,2029
23,9945	23,9259	24,2142	24,295	23,9954	23,926	22,7499	22,6886	23,1534
30,3316	30,3673	29,2568	29,1703	30,8167	30,8708	30,2033	30,2404	30,0519
22,4638	21,9299	22,5121	22,5457	NaN	NaN	23,3865	22,6193	22,2588
NaN	NaN	NaN	NaN	NaN	NaN	NaN	NaN	NaN
22,5467	22,762	21,7609	22,01	22,0947	22,2261	23,4227	23,36	22,8174
NaN	NaN	22,7019	22,1732	NaN	NaN	NaN	NaN	20,752
21,8242	NaN	NaN	NaN	22,4472	NaN	NaN	NaN	20,928
21,0479	21,1586	20,661	NaN	NaN	NaN	20,658	NaN	20,5298
22,7948	22,8864	22,6272	22,7782	NaN	NaN	22,426	22,503	22,8992
21,2616	20,9813	NaN	NaN	NaN	NaN	20,4489	NaN	21,0005
21,1041	20,4085	NaN	NaN	21,301	21,4995	20,5374	20,6841	20,6486
26,4656	26,4985	24,4678	24,6291	26,4584	26,5012	26,616	26,5953	23,2641
27,2511	27,1153	28,0268	28,1563	26,5912	26,636	27,8588	27,7853	27,8914
23,8734	23,8572	22,5721	22,9064	NaN	22,9664	NaN	NaN	24,0832
24,8341	24,7502	24,1648	24,4079	26,0646	26,0131	25,9166	26,0095	22,7486
22,1569	21,8615	21,2843	21,4791	22,337	22,1048	NaN	NaN	NaN
23,1831	23,2753	22,3239	22,7136	23,4686	23,4828	21,7793	21,9405	22,5319
NaN	NaN	NaN	NaN	NaN	NaN	NaN	NaN	NaN
NaN	NaN	NaN	NaN	NaN	NaN	21,2153	21,3849	NaN
24,6993	24,6793	25,8228	25,8428	25,1892	25,1561	25,8173	25,6518	24,8754
20,797	21,0068	20,865	20,7415	NaN	NaN	NaN	NaN	20,4709
23,243	22,8522	23,7731	24,2382	22,1401	21,8088	24,2142	23,9621	24,3786
21,696	22,3816	23,1288	23,1364	21,0073	21,0723	20,7973	20,4531	21,796
25,9393	25,9231	24,7042	24,7632	24,1922	24,4327	22,9605	22,4577	25,4979
23,8965	23,7191	22,5253	22,4958	22,3226	22,1119	21,6758	21,9527	23,3361
23,6456	23,4035	24,1726	23,9524	22,2429	22,3411	23,2942	23,2277	24,2767
23,2358	23,0718	22,6554	22,8562	22,0233	NaN	21,7307	21,8516	22,221
23,7054	23,7299	23,7003	23,9093	22,9624	22,8836	24,2585	24,3048	24,3281
21,8918	21,9777	21,7273	NaN	NaN	NaN	NaN	NaN	21,5741
22,2754	21,9348	NaN	NaN	23,9748	24,3135	23,0072	22,869	23,0291
22,7284	22,7721	21,2894	21,7215	21,0662	20,9419	22,7176	22,5839	22,7761
23,334	23,2689	23,3843	23,4615	22,4996	NaN	23,0533	23,1993	23,3715
22,0563	21,0573	21,3428	21,3877	20,8852	NaN	21,7755	21,471	21,6534
21,937	22,1356	21,9105	21,7217	21,6605	NaN	21,2205	21,6046	21,766
22,1005	22,4713	22,0411	21,8025	NaN	21,8319	NaN	NaN	21,9849
20,5948	20,5915	21,2479	20,9271	NaN	NaN	21,0565	21,0869	21,3571
23,3066	23,313	22,9451	22,6804	22,4624	22,7663	23,6983	23,5218	23,4684
20,9571	21,2012	21,1034	21,6721	NaN	NaN	NaN	NaN	21,1222
20,0043	20,0479	NaN	18,362	NaN	20,2661	20,2006	19,5539	20,1708
20,3247	20,4675	NaN	NaN	23,0762	23,0694	21,3301	21,327	NaN
21,2955	21,3753	21,9099	22,0585	20,8524	20,9179	21,4128	21,2036	21,7322
20,5846	20,545	20,6713	20,5708	21,3511	21,3871	21,2254	21,3755	22,0671
22,499	22,1996	22,3614	22,1005	NaN	NaN	23,0394	22,8811	22,4059
21,139	21,081	21,0358	21,2372	NaN	NaN	21,2065	20,9737	21,6942
23,2142	23,1408	22,679	22,4214	24,0183	23,7311	23,2787	23,0902	22,1308
22,0888	22,1493	21,5547	21,5961	NaN	NaN	21,5179	21,5436	22,3187
22,4404	22,369	23,0789	22,9865	21,5068	21,6877	23,1908	23,2731	23,0706
24,0034	23,9523	25,0728	24,9926	22,3206	22,4533	NaN	21,5915	25,1305
21,6065	NaN	NaN	NaN	22,6483	22,563	22,4362	22,353	NaN

20,0254	20,0265	NaN	19,8613	NaN	NaN	20,1587	20,6265	20,6541
25,9661	25,9633	26,017	26,0356	24,9207	24,993	24,8878	24,8146	25,6948
NaN	NaN	NaN	NaN	NaN	NaN	NaN	NaN	NaN
NaN	NaN	NaN	NaN	NaN	NaN	NaN	NaN	NaN
NaN	21,0981	NaN	21,5	NaN	NaN	NaN	NaN	21,3269
22,577	22,5642	22,3891	22,2735	23,2211	23,1773	22,5091	22,3091	22,3854
23,515	23,348	24,6599	24,7107	23,029	23,1066	24,9609	24,9485	24,497
21,2659	21,3649	22,7855	22,9348	20,8781	21,0901	22,4685	22,5581	22,5243
21,9019	21,611	21,6318	21,6148	20,6261	NaN	21,6355	21,6763	21,76
24,4713	24,5127	24,5545	24,4251	25,7426	25,7291	25,2191	25,2979	24,944
24,29	24,0815	24,0356	24,0913	26,5607	26,7356	25,2518	25,1346	23,9946
25,3455	25,1578	24,3047	24,5203	23,3182	23,2442	22,045	22,4926	24,3001
23,5641	23,7036	25,7734	25,8451	23,8579	23,8547	24,5873	24,405	24,3058
NaN	NaN	NaN	NaN	NaN	NaN	NaN	NaN	NaN
21,6049	22,1229	21,3971	21,3377	NaN	NaN	21,5405	21,7756	21,9614
27,3023	27,321	27,5407	27,5682	27,852	27,8544	27,5015	27,4302	27,9195
23,9394	24,0247	24,2808	24,1825	23,63	23,2565	24,351	24,4415	24,5201
21,0757	20,9225	21,7345	21,8023	22,5695	22,614	21,7103	21,654	21,5946
20,9804	21,0442	23,85	24,0038	20,0823	19,4677	19,2744	NaN	20,4238
NaN	NaN	NaN	NaN	NaN	NaN	NaN	NaN	NaN
24,15	24,148	24,9027	24,9858	23,5775	23,6091	24,9378	24,9427	24,3413
NaN	NaN	NaN	NaN	NaN	NaN	NaN	NaN	NaN
22,2083	21,8829	21,0533	21,0393	23,8562	23,9731	22,3784	22,1888	21,8444
21,4663	21,5877	21,3834	20,9697	21,0827	20,9616	21,3258	21,4782	21,9792
20,5509	21,4602	21,8618	21,9976	NaN	NaN	21,9501	22,1324	20,7052
25,1467	25,1156	22,8906	22,9627	23,4458	23,4107	23,0787	23,2925	24,5822
22,9089	22,9843	22,7841	22,9465	22,0786	21,8752	23,2228	23,0925	23,4087
NaN	NaN	20,5306	20,3143	NaN	NaN	NaN	NaN	20,5808
20,2995	20,6481	NaN	20,7147	NaN	NaN	20,6431	21,1284	20,991
23,9517	23,9593	23,4837	23,2663	22,9306	22,4458	24,879	24,8187	23,9779
20,817	NaN	NaN	NaN	NaN	NaN	20,677	21,0343	20,3999
27,7246	27,6795	26,1978	26,106	25,7279	25,6119	25,1458	25,1503	26,6954
20,8618	20,7543	21,2104	20,8799	NaN	NaN	NaN	NaN	20,5438
20,7766	21,6357	22,7726	21,2497	21,3301	NaN	22,4287	22,0277	20,9849
20,6892	20,2851	21,5433	21,5865	21,3217	NaN	21,4211	21,6223	21,6203
22,8894	22,8853	21,9527	22,5464	23,8998	23,4275	23,7228	24	22,3287
21,4407	21,5045	21,8316	22,3361	21,7804	NaN	21,492	21,8075	21,9821
20,3704	21,4073	NaN	NaN	NaN	NaN	NaN	NaN	NaN
23,876	23,9925	24,0095	23,9264	25,0852	25,0281	23,2002	22,8992	23,346
21,8485	22,075	21,5405	21,5333	NaN	NaN	21,1094	NaN	21,8729
NaN	NaN	20,7188	NaN	NaN	NaN	20,7929	NaN	NaN
23,0868	22,9692	22,6737	22,7088	22,2521	22,5278	23,3577	23,42	23,2303
23,6589	23,611	23,8076	23,6839	24,2407	24,4241	23,844	23,7269	23,6733
NaN	NaN	NaN	NaN	NaN	NaN	NaN	NaN	NaN
NaN	NaN	NaN	NaN	NaN	NaN	NaN	NaN	NaN
NaN	NaN	NaN	NaN	NaN	NaN	NaN	NaN	NaN
23,5889	23,5531	23,3476	23,3399	22,4716	22,3985	23,8859	23,9005	24,1116
21,4711	21,1607	21,607	21,3606	NaN	20,6158	22,147	22,0387	21,8485
21,9574	21,8497	NaN	NaN	NaN	NaN	NaN	NaN	20,5291
23,3918	23,0756	23,5498	23,5099	22,1616	NaN	23,5071	23,7	23,7013
NaN	NaN	NaN	NaN	21,5216	20,8699	20,6655	20,669	20,1632
22,3923	22,4204	NaN	21,8348	NaN	NaN	NaN	NaN	21,8894

19,9919	19,9029	19,6963	19,9562	19,1731	18,9265	20,1783	19,2664	19,8688
24,3347	24,1787	25,1083	25,0198	26,1496	26,2686	24,7086	24,7774	23,4844
22,4495	22,5551	NaN	20,9155	NaN	NaN	NaN	NaN	21,3191
NaN	NaN	NaN	NaN	NaN	NaN	NaN	NaN	NaN
22,2853	22,3127	22,1489	22,0192	22,8419	22,167	22,2378	22,1069	21,7334
NaN	NaN	21,4856	21,0303	NaN	NaN	20,8688	20,877	20,4876
24,3695	24,2773	24,5724	24,3893	25,0937	25,1946	24,211	24,2075	23,3369
21,0092	21,0365	20,393	20,5409	23,2812	23,806	NaN	NaN	NaN
21,2023	21,0711	20,5286	20,6476	NaN	NaN	20,4113	NaN	20,4182
22,3465	22,3243	21,9871	22,0748	NaN	NaN	23,0068	22,1724	22,3441
21,6533	NaN	23,1744	23,031	23,0594	23,1548	22,3419	22,3198	21,894
21,2691	21,3249	22,0473	22,146	NaN	NaN	23,3498	23,2213	22,4617
21,3193	NaN	22,1313	21,7004	NaN	NaN	21,7608	21,9501	21,6755
NaN	NaN	21,0238	20,8609	NaN	NaN	NaN	NaN	NaN
20,668	20,4817	20,6829	20,8252	NaN	NaN	20,5858	20,1186	20,689
20,8418	21,4957	NaN	NaN	NaN	NaN	NaN	NaN	21,0839
24,1462	24,1774	23,9395	23,9657	23,4504	23,6837	24,4244	24,3704	24,2389
NaN	NaN	NaN	NaN	NaN	NaN	NaN	NaN	NaN
25,9286	25,9456	25,3362	25,3501	26,1348	26,3563	25,2248	25,3172	25,3917
25,8059	25,7671	27,4216	27,4173	28,0299	27,9822	26,39	26,3274	25,8712
21,1951	21,31	21,4133	NaN	NaN	NaN	20,6717	20,4912	20,1183
20,9805	NaN	20,9741	21,2052	NaN	NaN	21,2213	21,4845	21,2177
24,8115	24,6731	26,4629	26,4623	25,5398	25,6128	24,0494	23,7507	24,2522
22,4667	22,3358	23,5095	23,3342	22,0293	22,5586	22,6275	22,3949	23,1687
19,5889	NaN	20,111	20,4588	NaN	NaN	NaN	20,2983	19,9694
NaN	NaN	NaN	NaN	NaN	NaN	NaN	NaN	NaN
22,1242	21,9879	21,4643	21,1702	22,3112	22,4842	21,8898	21,3729	21,0986
21,2273	NaN	NaN	NaN	NaN	NaN	NaN	NaN	21,6674
NaN	NaN	NaN	20,3474	NaN	NaN	22,2877	21,8518	20,7317
20,4978	NaN	NaN	NaN	NaN	NaN	21,0058	21,2258	21,0571
22,2645	21,5738	20,3211	NaN	NaN	20,1571	20,862	20,4188	21,6565
20,3893	20,1639	21,3701	20,5621	NaN	20,2192	20,1444	NaN	19,6576
NaN	NaN	NaN	NaN	21,5413	NaN	NaN	NaN	NaN
NaN	NaN	19,9744	19,9556	NaN	NaN	20,0125	20,1303	20,0556
NaN	NaN	NaN	NaN	NaN	NaN	NaN	NaN	20,6608
NaN	20,2348	20,4934	20,5491	NaN	NaN	21,0486	20,7186	20,5525
21,0653	21,2401	22,923	22,8245	NaN	20,6103	22,0953	22,1301	21,8485
20,7603	20,8093	20,76	21,0618	21,2931	21,3736	21,084	20,9699	20,2092
NaN	NaN	NaN	NaN	NaN	NaN	NaN	NaN	NaN
22,0042	22,0331	22,2748	22,2154	NaN	NaN	21,8145	21,9384	22,1786
NaN	NaN	19,9984	NaN	20,4627	20,6781	21,4065	21,7059	20,0929
21,2757	21,0187	21,1047	20,7379	20,9288	NaN	21,0964	21,602	22,0875
21,0105	20,8642	20,9953	21,0423	20,9072	NaN	21,3722	21,4664	20,8242
21,5076	22,1385	21,9411	22,0166	NaN	NaN	21,18	22,255	22,0401
20,8505	NaN	NaN	20,7857	NaN	21,1885	20,516	NaN	NaN
NaN	19,0503	NaN	NaN	NaN	NaN	19,3714	19,239	NaN
20,961	20,6654	NaN	20,7003	NaN	NaN	21,6126	21,3357	21,5161
21,98	21,859	21,6782	21,253	NaN	NaN	22,2685	23,0304	22,7701
22,2828	22,5809	22,4949	22,4752	23,0823	23,0772	22,4383	22,8977	22,1295
19,4502	NaN	NaN	NaN	NaN	NaN	19,8245	NaN	19,9628
21,8359	21,7952	21,0701	21,0145	22,7722	23,0315	21,7291	21,9888	21,9216
NaN	NaN	19,8176	NaN	NaN	NaN	NaN	NaN	20,045

NaN	NaN	20,3717	20,3627	NaN	NaN	21,2373	21,7156	NaN
21,3057	21,9993	21,8654	22,3644	NaN	NaN	21,5699	21,1842	21,5331
20,6041	20,5435	NaN	NaN	21,0726	21,3712	20,4349	20,2348	NaN
26,8529	26,8377	25,3561	25,4586	24,9186	25,0654	23,4629	23,6291	26,172
NaN	NaN	NaN	NaN	NaN	NaN	NaN	NaN	NaN
21,1758	21,5109	21,5158	21,1955	22,257	21,8691	21,0763	21,3567	21,1145
26,9139	26,9682	27,4371	27,2865	25,6315	25,7612	26,8695	26,8839	27,08
22,4157	22,2656	22,3247	22,5659	21,6843	21,5727	22,6088	22,634	22,7478
21,3306	22,3457	21,8846	22,0286	NaN	NaN	21,7204	21,8665	22,6988
NaN	NaN	23,7904	23,7403	19,7278	NaN	NaN	20,1268	NaN
NaN	NaN	21,2317	21,2847	NaN	NaN	NaN	NaN	21,2405
27,5767	27,6169	27,0828	26,9838	25,4172	25,6419	24,5551	24,6348	26,8643
NaN	NaN	NaN	NaN	NaN	NaN	23,1376	22,6364	NaN
19,8194	NaN	20,5467	20,5995	NaN	NaN	19,938	19,6666	NaN
21,5064	21,0933	20,7732	20,7095	20,7049	NaN	20,4974	20,2131	21,2906
21,5252	21,1141	20,0577	NaN	NaN	NaN	NaN	NaN	20,4476
21,0143	21,0393	NaN	NaN	22,32	22,5663	NaN	NaN	20,8391
24,674	25,2084	24,3405	24,3783	22,3796	22,9242	22,6052	22,7797	22,1462
21,621	21,6656	21,3782	21,6353	20,6567	20,8721	21,5955	21,6702	22,2071
20,6647	20,6252	20,0659	20,9235	NaN	19,8215	22,0929	21,4175	21,2907
25,5462	25,5758	24,655	24,5483	24,2199	24,1802	24,1009	24,3249	25,3537
22,1959	21,6557	21,8461	21,4372	NaN	NaN	21,4297	21,6242	22,1378
25,6827	25,8053	26,4408	26,502	25,1263	25,3959	26,3715	26,4247	26,3435
24,1707	24,2905	24,0095	23,7168	22,902	23,9377	26,8039	26,3755	25,3272
20,2903	20,3617	20,1028	NaN	NaN	NaN	21,3088	20,6878	21,0742
24,5238	24,5553	22,3017	21,8984	23,0316	23,3242	22,0401	22,4419	23,8147
NaN	NaN	NaN	NaN	NaN	NaN	NaN	NaN	19,6157
24,0397	23,857	22,8956	22,9212	NaN	NaN	NaN	NaN	23,2368
24,6099	24,6478	23,2897	23,3549	25,3958	25,3532	24,6233	24,7753	24,4577
23,1362	23,0196	21,764	21,8876	22,0037	21,9022	20,6123	20,3234	22,4604
NaN	NaN	NaN	NaN	NaN	NaN	NaN	NaN	NaN
23,9232	23,8065	25,1746	25,2435	23,3724	23,0402	25,8904	25,7736	25,7233
23,7665	23,5743	23,0067	23,1557	22,5203	22,5211	23,278	22,9614	23,7238
23,8111	23,856	23,8495	23,7776	24,111	24,0846	24,1327	24,0189	23,6933
NaN	NaN	NaN	NaN	20,6687	NaN	20,6463	20,4114	20,5258
21,5856	21,7358	21,9115	22,0095	NaN	NaN	21,2532	NaN	21,5121
22,074	22,2774	22,8037	22,6565	20,6225	NaN	22,1552	22,3504	22,9675
20,7991	20,6667	20,9114	20,7125	21,6173	21,1613	21,7388	21,6546	19,8927
21,0999	21,0534	21,4372	21,6141	20,1038	20,4943	21,684	21,5788	21,7983
20,0846	NaN	NaN	NaN	20,8424	20,8263	NaN	NaN	20,1046
22,4297	22,8407	24,2998	24,0077	NaN	23,0213	25,2904	25,3886	24,4162
21,2762	20,7248	21,1304	21,065	NaN	NaN	21,0616	NaN	NaN
19,8353	20,0682	20,3762	20,7177	NaN	NaN	20,7297	20,6312	20,218
21,0908	NaN	NaN	22,9304	NaN	NaN	NaN	NaN	NaN
20,9231	20,9403	NaN	NaN	NaN	NaN	22,3285	22,4479	21,2658
19,4119	NaN	NaN	NaN	21,4243	NaN	19,9784	20,3812	20,5195
NaN	NaN	NaN	NaN	NaN	NaN	NaN	20,9502	21,4278
21,1402	21,2151	NaN	NaN	NaN	NaN	21,1795	21,0531	21,2963
NaN	NaN	NaN	20,8565	NaN	NaN	NaN	NaN	NaN
NaN	NaN	NaN	NaN	NaN	NaN	NaN	NaN	20,3244
23,9008	23,8974	22,0575	21,9626	22,5917	22,935	22,0359	22,0919	22,9981
21,5818	21,4487	21,1792	21,1716	NaN	NaN	20,976	NaN	NaN

NaN	NaN	NaN	NaN	NaN	NaN	NaN	NaN	NaN
23,9272	24,124	24,0741	24,0177	26,0259	25,8939	25,2123	25,2776	23,8033
26,9232	27,0081	26,4098	26,5361	27,4394	27,3831	27,0087	26,611	25,271
24,7908	24,7786	23,952	23,9214	25,0468	24,938	24,5755	24,6113	24,8468
25,176	25,2688	24,0246	24,0423	25,9814	25,9995	24,9198	24,8204	24,2903
25,9636	25,9362	25,541	25,6684	24,5097	24,5867	24,2028	24,1092	25,7352
NaN	NaN	NaN	NaN	NaN	NaN	NaN	NaN	NaN
NaN	NaN	NaN	NaN	NaN	NaN	NaN	NaN	NaN
20,9901	20,4843	NaN	NaN	NaN	NaN	20,6263	20,2574	21,1637
21,1608	21,4781	21,9168	21,4213	NaN	NaN	21,5519	21,3648	22,0254
NaN	NaN	20,5009	20,6128	20,3097	20,3749	NaN	19,7406	19,6964
NaN	NaN	NaN	NaN	NaN	NaN	NaN	NaN	NaN
NaN	20,2216	NaN	NaN	NaN	NaN	20,3336	NaN	NaN
NaN	NaN	NaN	NaN	NaN	NaN	NaN	NaN	NaN
NaN	NaN	NaN	NaN	NaN	NaN	21,002	21,7869	20,6472
NaN	20,2817	NaN	NaN	NaN	NaN	NaN	NaN	20,5667
20,6743	20,6987	22,0986	21,7586	NaN	19,7971	20,904	20,8859	20,6997
NaN	NaN	NaN	NaN	NaN	NaN	NaN	NaN	NaN
NaN	NaN	NaN	NaN	NaN	NaN	NaN	NaN	NaN
21,054	20,9679	20,6703	20,6548	NaN	NaN	20,6264	20,7708	20,9746
NaN	NaN	20,7374	NaN	NaN	NaN	NaN	NaN	NaN
NaN	NaN	NaN	NaN	NaN	NaN	NaN	NaN	NaN
21,6447	21,782	22,5449	22,681	21,3673	NaN	22,5744	22,5193	22,4908
NaN	NaN	NaN	NaN	NaN	NaN	NaN	NaN	NaN
21,1176	20,8657	21,499	21,6759	21,4946	21,5613	21,5801	21,4554	20,8091
20,5521	20,7569	20,7769	20,9844	20,6004	20,5159	20,4428	20,316	20,8962
NaN	NaN	NaN	NaN	NaN	NaN	23,0076	23,405	23,154
21,0302	20,3924	20,6879	20,9096	NaN	NaN	20,5247	20,8043	20,4627
24,5233	24,5176	24,7789	24,9833	23,6383	23,6144	23,818	23,8036	24,3977
20,9612	21,3314	20,6201	20,6654	NaN	NaN	NaN	NaN	20,7468
NaN	20,4629	NaN	NaN	NaN	NaN	NaN	19,8852	20,0242
22,3435	22,392	22,6201	22,5403	21,3802	21,5593	22,5998	22,8394	22,9221
NaN	20,3331	NaN	NaN	NaN	NaN	20,3087	NaN	20,9418
20,5854	20,8048	NaN	NaN	NaN	NaN	NaN	20,6706	21,4675
19,8983	NaN	20,4579	20,4962	NaN	NaN	20,7326	NaN	20,4643
NaN	21,3001	21,1485	20,7781	NaN	21,0345	NaN	NaN	21,422
21,601	21,2001	NaN	NaN	NaN	NaN	NaN	NaN	21,0067
NaN	NaN	NaN	NaN	NaN	NaN	NaN	NaN	NaN
NaN	NaN	NaN	NaN	NaN	NaN	20,8088	20,7258	NaN
NaN	NaN	NaN	NaN	NaN	NaN	NaN	NaN	20,3316
20,2572	20,3286	NaN	NaN	NaN	NaN	NaN	NaN	20,4476
NaN	NaN	NaN	NaN	NaN	NaN	NaN	NaN	NaN
19,2958	19,7045	20,2013	19,232	NaN	NaN	NaN	NaN	18,7425
21,4936	21,8488	21,9286	22,2359	22,2042	22,5103	21,8427	21,8576	21,2254
21,3276	22,0882	21,3592	21,5697	20,6293	20,987	22,1237	21,8878	NaN
21,3586	21,4946	22,5067	22,8386	NaN	NaN	22,3828	22,5018	21,5599
22,0439	22,035	20,9561	NaN	21,0291	21,2813	20,8285	NaN	21,8272
NaN	NaN	NaN	NaN	NaN	NaN	19,4384	NaN	19,2498
20,7312	20,9591	NaN	NaN	NaN	NaN	NaN	NaN	20,8307
NaN	NaN	NaN	NaN	NaN	NaN	NaN	NaN	NaN
21,46	21,5362	NaN	22,3077	NaN	NaN	NaN	NaN	NaN
21,6104	21,7243	22,14	22,0759	NaN	NaN	20,1116	20,0962	20,1518

22,3647	NaN	20,4398	21,0327	NaN	NaN	NaN	NaN	20,5802
NaN	NaN	NaN	NaN	NaN	NaN	20,6366	NaN	NaN
21,2687	21,523	21,4575	NaN	21,3237	NaN	20,7155	20,6013	21,1087
20,1117	20,4851	NaN	20,6971	NaN	NaN	20,7209	21,0453	20,9425
22,3023	22,041	21,3421	21,1151	NaN	NaN	NaN	20,8626	21,4503
20,5227	20,332	21,5996	21,4553	NaN	NaN	21,185	21,2026	21,0362
NaN	20,8792	NaN	NaN	NaN	NaN	NaN	NaN	20,8823
21,144	20,7191	22,1644	22,0691	NaN	NaN	21,7717	21,7343	21,1131
NaN	NaN	NaN	NaN	NaN	NaN	NaN	NaN	NaN
NaN	20,8428	NaN	NaN	NaN	NaN	NaN	NaN	20,6574
NaN	NaN	NaN	NaN	NaN	NaN	NaN	NaN	NaN
NaN	NaN	NaN	NaN	NaN	NaN	NaN	NaN	NaN
24,1383	23,9914	22,8936	22,87	24,7677	24,8244	NaN	25,1465	23,2177
NaN	NaN	22,2413	22,3579	NaN	NaN	NaN	NaN	NaN
NaN	NaN	NaN	NaN	NaN	NaN	NaN	NaN	22,4472
21,1464	21,0674	20,1515	20,2573	20,5769	20,6718	NaN	19,4236	21,1895
NaN	NaN	22,0414	NaN	NaN	NaN	21,7282	21,7357	22,151
26,0136	26,1309	26,0767	26,0829	26,7685	26,856	26,5027	26,4488	24,9961
21,6486	21,2447	21,1977	21,3376	20,5291	20,4504	20,6681	20,7172	22,0505
24,174	24,0452	24,1944	24,0475	24,1369	24,067	23,5878	23,7357	23,6157
NaN	NaN	20,9507	NaN	NaN	NaN	NaN	NaN	20,9075
NaN	NaN	NaN	NaN	NaN	NaN	NaN	NaN	NaN
NaN	NaN	NaN	NaN	22,6042	22,8093	NaN	NaN	NaN
NaN	NaN	20,6728	20,42	20,9611	NaN	NaN	19,9557	19,9422
24,1255	24,1071	23,8782	23,599	24,4444	24,3264	24,0483	24,3314	23,804
20,8378	20,6686	20,2471	20,3987	NaN	NaN	20,6182	20,5664	20,9326
21,5078	21,341	NaN	NaN	NaN	NaN	19,99	NaN	20,8289
NaN	NaN	NaN	NaN	NaN	NaN	NaN	NaN	NaN
20,5954	20,7036	NaN	NaN	NaN	NaN	NaN	NaN	NaN
NaN	NaN	21,4644	NaN	NaN	NaN	NaN	NaN	NaN
21,3412	20,9793	21,5904	20,9935	NaN	NaN	22,2815	22,5491	20,8969
NaN	20,3532	21,8991	22,069	NaN	NaN	21,3155	21,6345	21,3475
NaN	NaN	NaN	NaN	NaN	NaN	NaN	NaN	NaN
20,1672	20,0534	NaN	NaN	NaN	NaN	NaN	NaN	19,7862
NaN	NaN	NaN	NaN	NaN	NaN	NaN	NaN	NaN
21,2655	21,4161	21,0695	21,2056	22,1483	21,9578	21,3142	21,1886	20,4207
NaN	NaN	20,3927	20,1038	NaN	NaN	NaN	NaN	NaN
NaN	NaN	NaN	NaN	NaN	NaN	NaN	NaN	NaN
NaN	NaN	NaN	NaN	NaN	NaN	NaN	NaN	NaN
NaN	NaN	NaN	20,4331	NaN	NaN	22,0896	22,7652	20,6935
NaN	NaN	20,3494	19,6074	NaN	NaN	NaN	NaN	NaN
NaN	20,0296	20,3937	NaN	NaN	NaN	20,2551	NaN	NaN
20,1284	20,2666	NaN	19,9366	NaN	NaN	21,6271	21,9565	20,4043
22,8859	22,8912	22,4828	22,6979	21,7379	21,9294	22,5393	22,3675	23,2542
21,2341	21,1394	21,8446	21,845	NaN	NaN	21,5736	21,3439	21,4033
21,357	21,4027	21,2889	21,6044	20,9327	NaN	21,2011	21,5426	NaN
21,8036	21,5178	20,2255	20,4449	23,9224	23,6991	22,4146	22,6655	23,0203
NaN	NaN	NaN	NaN	NaN	NaN	NaN	NaN	NaN
19,8406	20,2246	20,2781	20,0335	NaN	NaN	21,0167	20,9541	21,4863
20,2017	20,6871	NaN	NaN	NaN	NaN	NaN	NaN	NaN
22,6777	23,4406	23,2031	23,2025	21,2845	21,1682	20,9479	20,9599	23,9542
25,6832	25,7084	25,0896	25,1388	22,8955	23,0002	23,4414	23,5914	25,013

NaN	NaN	NaN	20,9989	NaN	NaN	NaN	NaN	NaN
NaN	NaN	NaN	NaN	NaN	NaN	NaN	19,8399	19,6544
NaN	19,6357	NaN	NaN	NaN	NaN	NaN	NaN	19,5351
NaN	NaN	20,151	NaN	NaN	NaN	20,0884	20,1103	19,9822
18,3436	NaN	19,0296	NaN	NaN	NaN	NaN	NaN	NaN
NaN	NaN	NaN	NaN	NaN	NaN	NaN	NaN	NaN
NaN	NaN	NaN	NaN	NaN	NaN	NaN	NaN	NaN
NaN	NaN	NaN	NaN	NaN	NaN	NaN	NaN	NaN
NaN	NaN	NaN	NaN	NaN	NaN	NaN	NaN	NaN
24,0372	24,085	24,577	24,374	23,2251	23,4269	24,0043	23,9051	24,0073
NaN	NaN	NaN	NaN	21,4978	NaN	21,1314	20,9378	NaN
22,5069	22,4517	22,4307	22,417	23,18	23,2107	22,3833	22,4553	22,1126
NaN	NaN	NaN	NaN	NaN	NaN	NaN	NaN	NaN
24,8951	24,8807	24,6455	24,7889	25,5406	25,5728	24,4383	24,2787	24,6084
NaN	NaN	NaN	NaN	20,1704	21,1144	NaN	19,6651	19,3332
20,5328	20,5345	NaN	21,1265	NaN	NaN	NaN	20,6775	20,8378
22,7116	22,566	22,5235	22,3965	21,6091	21,3333	23,2235	23,2975	23,5748
NaN	NaN	NaN	NaN	NaN	NaN	NaN	NaN	NaN
NaN	NaN	NaN	NaN	NaN	NaN	NaN	NaN	NaN
23,3781	23,4385	24,2839	24,3142	23,2945	23,4549	24,5635	24,3987	24,0794
NaN	NaN	NaN	NaN	NaN	NaN	NaN	NaN	NaN
23,9389	24,136	23,2562	23,7128	23,4147	23,1302	23,7729	23,7028	24,5306
NaN	NaN	NaN	NaN	NaN	NaN	NaN	NaN	NaN
NaN	NaN	19,677	19,7012	NaN	NaN	NaN	NaN	NaN
NaN	NaN	NaN	NaN	NaN	NaN	NaN	NaN	NaN
21,8751	NaN	23,4897	23,9131	22,4643	23,0013	24,0786	24,0979	22,9465
21,8709	22,1404	22,6592	22,8022	23,7566	23,3343	23,6739	23,393	22,7798
NaN	20,2066	20,4476	20,4276	NaN	NaN	20,699	NaN	20,1311
19,062	NaN	21,1909	20,7282	NaN	NaN	NaN	18,8157	NaN
NaN	NaN	NaN	NaN	NaN	NaN	19,9651	20,7907	20,4383
NaN	NaN	20,045	20,1219	NaN	NaN	20,0383	20,0939	20,09
23,0697	23,0584	22,8744	22,9918	21,4849	21,2754	20,6172	NaN	22,4226
NaN	NaN	NaN	NaN	NaN	NaN	NaN	NaN	NaN
20,798	20,7173	NaN	NaN	22,2617	22,4047	21,8606	21,9474	NaN
23,2726	23,0352	22,2327	21,9591	NaN	NaN	NaN	NaN	23,0944
NaN	NaN	NaN	NaN	NaN	NaN	NaN	NaN	NaN
NaN	NaN	NaN	NaN	NaN	NaN	NaN	NaN	NaN
NaN	20,2111	NaN	NaN	NaN	NaN	20,8422	20,9362	20,6204
28,3105	28,3769	28,9391	28,9723	27,0606	27,0565	29,1762	29,0877	27,9465
22,6745	22,0678	22,2736	22,2065	NaN	NaN	NaN	21,5914	22,4862
20,1803	NaN	20,1129	20,4072	NaN	NaN	21,0735	20,6945	20,5599
NaN	NaN	23,1405	22,8123	22,9709	22,8544	NaN	21,1858	23,12
20,6247	NaN	20,4773	NaN	21,3694	NaN	NaN	NaN	21,2482
21,0916	21,441	21,1164	21,2	NaN	20,375	21,6543	21,4521	21,6497
NaN	NaN	NaN	NaN	NaN	NaN	NaN	NaN	NaN
22,893	23,045	NaN	NaN	23,087	23,5139	23,5584	23,4463	23,054
21,0785	NaN	NaN	20,4499	NaN	NaN	NaN	NaN	21,5367
NaN	NaN	NaN	20,712	NaN	21,3443	NaN	NaN	20,8372
21,3297	21,5025	22,973	22,9813	22,1203	21,0742	21,4531	NaN	22,3205
NaN	NaN	NaN	NaN	NaN	23,249	NaN	21,4728	22,3027
NaN	23,6754	27,6664	27,7019	NaN	NaN	NaN	NaN	27,4425
NaN	NaN	NaN	NaN	NaN	NaN	NaN	NaN	NaN

20,2823	20,497	20,4957	20,5097	21,7017	22,2664	NaN	20,7034	NaN
22,2161	21,9686	23,0753	23,2451	21,6704	21,7704	22,5238	22,5827	22,4665
NaN	NaN	NaN	NaN	NaN	NaN	NaN	NaN	NaN
NaN	NaN	NaN	NaN	NaN	NaN	NaN	NaN	NaN
NaN	NaN	NaN	NaN	NaN	NaN	NaN	NaN	NaN
NaN	21,2174	NaN	NaN	NaN	21,7304	NaN	NaN	NaN
NaN	NaN	NaN	NaN	NaN	NaN	19,2335	19,7048	NaN
NaN	20,801	22,3856	22,1026	NaN	NaN	20,7316	NaN	20,8311
NaN	NaN	NaN	NaN	NaN	NaN	NaN	NaN	NaN
20,8296	20,5012	NaN	20,5933	NaN	20,9511	NaN	NaN	20,1296
NaN	NaN	NaN	NaN	NaN	NaN	NaN	NaN	NaN
NaN	NaN	20,7588	NaN	NaN	NaN	NaN	20,0083	20,4321
NaN	NaN	NaN	NaN	NaN	NaN	NaN	NaN	NaN
NaN	NaN	NaN	NaN	NaN	NaN	NaN	NaN	NaN
NaN	NaN	NaN	NaN	NaN	20,6133	NaN	NaN	NaN
NaN	NaN	NaN	NaN	NaN	NaN	NaN	NaN	18,487
NaN	NaN	NaN	NaN	NaN	NaN	NaN	NaN	NaN
NaN	NaN	NaN	NaN	NaN	NaN	NaN	NaN	NaN
NaN	NaN	NaN	NaN	NaN	NaN	NaN	NaN	NaN
19,6027	NaN	NaN	NaN	NaN	NaN	NaN	NaN	NaN
20,226	NaN	20,0708	20,1859	NaN	NaN	20,0203	20,155	NaN
19,0853	NaN	NaN	NaN	NaN	NaN	NaN	NaN	19,4485
19,889	NaN	NaN	NaN	NaN	NaN	NaN	19,874	NaN
NaN	NaN	NaN	NaN	NaN	NaN	NaN	NaN	NaN
NaN	NaN	NaN	NaN	NaN	NaN	NaN	NaN	NaN
NaN	NaN	NaN	NaN	NaN	NaN	NaN	NaN	NaN
20,4908	20,4018	NaN	20,6671	20,0706	19,4913	20,2044	19,7956	21,0738
18,8749	NaN	NaN	NaN	NaN	NaN	NaN	NaN	NaN
NaN	NaN	23,6174	24,1397	26,1812	26,1773	22,7385	22,826	NaN
NaN	NaN	NaN	NaN	NaN	NaN	NaN	NaN	NaN
22,8007	22,9385	22,9247	22,3683	23,3789	23,5678	22,8386	23,0168	22,8849
21,97	22,7022	22,626	22,0422	21,6264	22,6548	21,7614	21,4059	21,1949
NaN	NaN	NaN	NaN	NaN	NaN	NaN	NaN	NaN
NaN	NaN	NaN	NaN	NaN	NaN	NaN	NaN	20,8012
NaN	NaN	NaN	NaN	NaN	NaN	NaN	NaN	19,8551
23,1137	22,8248	23,5087	23,4467	21,5629	21,7632	21,3156	21,147	21,9584
NaN	NaN	NaN	NaN	NaN	NaN	NaN	NaN	NaN
NaN	NaN	20,8421	NaN	NaN	NaN	NaN	NaN	20,8567
NaN	NaN	NaN	23,4679	24,4182	24,6504	24,2408	24,1683	23,4397
23,3403	23,4377	NaN	21,8564	23,0142	NaN	22,4193	22,5717	21,4733
NaN	NaN	NaN	NaN	NaN	NaN	NaN	NaN	NaN
24,0571	24,3403	23,4948	23,5958	25,655	25,3121	24,7188	24,392	23,3661
NaN	NaN	NaN	NaN	NaN	NaN	NaN	NaN	NaN
NaN	NaN	NaN	NaN	NaN	NaN	NaN	NaN	NaN
NaN	NaN	NaN	NaN	NaN	NaN	NaN	19,7733	NaN
NaN	21,5604	NaN	21,1946	NaN	NaN	NaN	NaN	21,5342
NaN	NaN	NaN	NaN	22,6056	NaN	NaN	NaN	NaN
NaN	NaN	NaN	NaN	NaN	NaN	NaN	NaN	NaN
21,427	21,5245	21,9909	22,091	21,1393	21,3373	20,8895	20,9243	21,5642
NaN	NaN	NaN	NaN	NaN	NaN	NaN	NaN	NaN
22,6628	22,5386	22,602	22,5564	23,2496	23,2031	22,878	22,8495	22,3339
25,3566	24,8914	24,3432	24,011	25,4192	25,6321	25,4952	25,0151	23,9898

NaN	22,3508	NaN	22,9857	NaN	NaN	22,6768	22,5962	22,9617
NaN	NaN	NaN	NaN	NaN	NaN	NaN	NaN	NaN
22,7187	22,4685	23,161	23,1009	23,0921	22,5017	22,846	22,7293	22,5976
19,8189	19,8197	NaN	20,197	NaN	NaN	20,7172	21,508	21,632
NaN	NaN	NaN	NaN	NaN	NaN	NaN	NaN	NaN
NaN	NaN	NaN	NaN	NaN	NaN	NaN	NaN	NaN
NaN	NaN	NaN	NaN	NaN	NaN	NaN	NaN	NaN
21,2205	21,4674	21,2991	NaN	NaN	NaN	NaN	NaN	21,1781
NaN	NaN	NaN	NaN	NaN	NaN	NaN	NaN	NaN
NaN	NaN	NaN	NaN	NaN	NaN	NaN	NaN	NaN
NaN	NaN	NaN	NaN	NaN	NaN	NaN	NaN	NaN
NaN	NaN	NaN	NaN	NaN	NaN	NaN	NaN	NaN
NaN	NaN	NaN	NaN	NaN	NaN	NaN	NaN	NaN
21,2803	NaN	NaN	NaN	NaN	NaN	NaN	NaN	NaN
NaN	NaN	NaN	NaN	NaN	NaN	NaN	NaN	NaN
20,1556	20,4684	NaN	NaN	NaN	NaN	NaN	NaN	19,9382
NaN	NaN	NaN	NaN	NaN	NaN	NaN	NaN	NaN
19,8557	NaN	19,4498	19,6865	19,7456	NaN	21,2992	21,1318	20,3074
19,8103	20,0943	NaN	NaN	NaN	20,7209	20,3243	20,2783	NaN
22,0035	21,2889	21,8611	21,9894	NaN	NaN	21,9429	21,8217	21,5871
21,2187	21,5381	21,5613	21,3072	22,0665	22,0792	21,2326	21,2538	21,783
NaN	NaN	NaN	NaN	NaN	NaN	NaN	NaN	NaN
NaN	NaN	NaN	NaN	NaN	19,8329	20,7895	20,3129	NaN
NaN	NaN	NaN	NaN	NaN	NaN	NaN	NaN	NaN
NaN	NaN	NaN	NaN	NaN	NaN	NaN	NaN	NaN
NaN	NaN	NaN	NaN	NaN	NaN	NaN	NaN	NaN
NaN	NaN	NaN	NaN	NaN	NaN	NaN	NaN	NaN
NaN	NaN	NaN	NaN	NaN	NaN	NaN	NaN	NaN
20,3134	20,508	NaN	NaN	NaN	NaN	NaN	NaN	20,7516
NaN	NaN	NaN	NaN	NaN	NaN	19,8271	NaN	NaN
NaN	NaN	NaN	NaN	NaN	NaN	NaN	NaN	NaN
NaN	21,3951	NaN	NaN	NaN	21,0144	22,3124	22,0215	21,6214
NaN	NaN	NaN	NaN	NaN	NaN	NaN	NaN	NaN
NaN	NaN	NaN	NaN	NaN	NaN	NaN	NaN	NaN
NaN	NaN	20,3991	20,4473	NaN	NaN	NaN	NaN	NaN
21,046	21,3142	NaN	20,9439	20,7711	21,0205	NaN	NaN	21,5518
21,1113	21,6605	22,4635	22,4244	23,0429	22,9224	21,969	22,1478	22,0764
NaN	NaN	25,2155	NaN	NaN	NaN	NaN	NaN	NaN
23,671	24,0654	24,0092	24,4383	NaN	23,5412	23,4345	23,0971	23,829
19,7019	19,6923	NaN	NaN	20,0523	NaN	NaN	NaN	NaN
NaN	NaN	19,7064	NaN	NaN	NaN	NaN	NaN	20,1407
24,1481	24,3875	24,0945	24,01	25,5961	25,4283	24,6395	24,7194	24,0321
21,207	21,0364	20,859	21,0911	21,9078	21,6509	22,6552	22,7164	21,7536
NaN	NaN	NaN	NaN	NaN	NaN	21,0454	20,9698	20,8584
20,5474	20,2936	NaN	20,3252	NaN	NaN	NaN	NaN	20,5029
19,5986	19,4662	20,4731	20,9519	NaN	NaN	19,4338	19,4131	NaN
NaN	NaN	NaN	NaN	NaN	NaN	NaN	NaN	NaN
21,5788	21,8579	21,4555	NaN	NaN	NaN	NaN	NaN	21,7147
21,227	21,8305	20,58	NaN	NaN	NaN	NaN	NaN	20,9831
20,8517	20,62	NaN	NaN	NaN	NaN	21,2094	21,0358	21,4146
NaN	NaN	NaN	NaN	NaN	NaN	NaN	NaN	NaN
NaN	NaN	NaN	NaN	NaN	NaN	NaN	NaN	NaN

NaN	NaN	NaN	NaN	NaN	NaN	NaN	NaN	NaN
NaN	NaN	23,9226	23,6007	NaN	NaN	23,0575	23,1577	23,1633
NaN	NaN	NaN	NaN	NaN	NaN	NaN	NaN	NaN
NaN	NaN	20,841	20,6455	NaN	NaN	NaN	NaN	20,448
NaN	NaN	NaN	NaN	NaN	NaN	NaN	NaN	NaN
23,879	23,7753	23,2677	23,1092	NaN	NaN	21,8513	21,8539	22,2063
21,9232	21,9641	21,7545	NaN	NaN	NaN	21,8433	22,3247	21,9631
25,2655	25,1854	24,9092	25,0828	26,0187	26,2479	26,136	25,9409	25,1168
NaN	NaN	NaN	NaN	NaN	NaN	NaN	NaN	19,2088
21,9929	22,274	NaN	NaN	NaN	NaN	NaN	NaN	NaN
22,0308	22,4356	21,7531	21,8404	22,7408	22,5684	22,3146	22,3703	21,4185
NaN	NaN	22,7841	NaN	NaN	NaN	23,8973	23,8579	23,4008
NaN	NaN	NaN	NaN	NaN	NaN	NaN	NaN	NaN
NaN	NaN	NaN	NaN	NaN	NaN	NaN	23,7769	NaN
NaN	NaN	NaN	NaN	NaN	NaN	NaN	NaN	20,1108
24,4714	23,4481	23,0555	22,9806	24,183	24,4961	23,7497	23,2291	23,7967
20,0568	20,1039	NaN	NaN	NaN	NaN	21,7075	NaN	NaN
20,2176	19,8469	NaN	NaN	NaN	20,4646	NaN	NaN	20,237
NaN	NaN	NaN	NaN	NaN	NaN	NaN	20,0819	NaN
NaN	24,3224	23,6958	23,8367	25,6596	24,6262	24,0743	24,0776	NaN
NaN	NaN	NaN	NaN	NaN	NaN	NaN	NaN	NaN
21,1454	21,1473	21,0393	21,0011	NaN	NaN	20,3269	19,8516	20,8564
23,0603	23,0601	23,2461	23,1539	NaN	NaN	NaN	NaN	NaN
NaN	NaN	NaN	NaN	NaN	NaN	NaN	NaN	NaN
NaN	NaN	NaN	NaN	NaN	NaN	NaN	NaN	NaN
20,6609	20,9174	NaN	20,2167	NaN	20,9086	20,4957	NaN	NaN
NaN	NaN	NaN	NaN	NaN	NaN	NaN	NaN	NaN
23,6564	23,4554	23,05	23,2083	24,0483	23,7905	22,8193	23,1121	23,0711
NaN	NaN	NaN	NaN	NaN	NaN	NaN	NaN	NaN
NaN	NaN	NaN	NaN	NaN	NaN	NaN	NaN	NaN
NaN	NaN	NaN	NaN	NaN	NaN	NaN	NaN	NaN
NaN	NaN	NaN	NaN	NaN	NaN	NaN	NaN	NaN
NaN	NaN	NaN	NaN	NaN	20,9238	NaN	20,3323	NaN
NaN	NaN	20,7218	20,833	NaN	NaN	20,4913	NaN	20,5835
NaN	NaN	NaN	NaN	NaN	NaN	NaN	NaN	NaN
NaN	NaN	NaN	19,4825	NaN	NaN	NaN	NaN	NaN
NaN	NaN	NaN	NaN	NaN	NaN	22,1014	21,8012	NaN
NaN	NaN	19,922	NaN	NaN	NaN	NaN	NaN	NaN
NaN	NaN	NaN	NaN	NaN	NaN	NaN	NaN	NaN
NaN	NaN	NaN	NaN	NaN	NaN	NaN	NaN	NaN
NaN	NaN	NaN	NaN	NaN	NaN	NaN	NaN	NaN
NaN	NaN	NaN	NaN	NaN	NaN	NaN	NaN	NaN
NaN	NaN	NaN	NaN	NaN	NaN	NaN	NaN	NaN
NaN	NaN	NaN	NaN	NaN	NaN	NaN	NaN	NaN
21,174	21,0751	21,4996	21,1982	21,9891	21,6656	21,2477	21,2128	NaN
NaN	18,866	NaN	NaN	NaN	NaN	NaN	NaN	NaN
NaN	NaN	NaN	NaN	NaN	NaN	NaN	NaN	NaN
NaN	NaN	NaN	NaN	NaN	NaN	NaN	NaN	NaN
NaN	NaN	NaN	NaN	NaN	NaN	NaN	NaN	NaN
20,2691	19,6783	NaN	NaN	NaN	NaN	20,3662	20,8632	NaN
NaN	NaN	NaN	NaN	NaN	NaN	NaN	NaN	NaN
21,7756	21,8045	21,9772	22,1585	NaN	NaN	NaN	NaN	21,5818

NaN	NaN	NaN	NaN	NaN	NaN	NaN	NaN	NaN
NaN	NaN	NaN	NaN	NaN	NaN	NaN	NaN	NaN
NaN	NaN	NaN	NaN	NaN	NaN	19,9476	NaN	NaN
NaN	NaN	NaN	19,4103	NaN	NaN	NaN	19,6801	NaN
NaN	NaN	NaN	NaN	NaN	NaN	NaN	NaN	NaN
NaN	20,0499	20,1121	NaN	NaN	NaN	NaN	NaN	20,3976
NaN	NaN	NaN	NaN	20,3081	NaN	NaN	NaN	NaN
NaN	NaN	NaN	NaN	NaN	NaN	NaN	NaN	NaN
NaN	NaN	NaN	NaN	NaN	NaN	NaN	NaN	NaN
NaN	NaN	NaN	NaN	NaN	NaN	NaN	NaN	NaN
NaN	NaN	NaN	NaN	NaN	NaN	NaN	20,2375	NaN
NaN	NaN	NaN	NaN	NaN	NaN	NaN	NaN	NaN
NaN	NaN	NaN	NaN	NaN	NaN	NaN	NaN	NaN
NaN	NaN	NaN	NaN	NaN	NaN	NaN	NaN	NaN
NaN	NaN	NaN	NaN	NaN	NaN	NaN	NaN	NaN
NaN	NaN	NaN	NaN	NaN	NaN	NaN	NaN	NaN
18,881	18,9922	NaN	NaN	NaN	NaN	NaN	18,7759	19,2301
NaN	NaN	NaN	NaN	NaN	NaN	NaN	23,4707	NaN
20,6023	20,5571	NaN	NaN	NaN	NaN	NaN	NaN	20,515
NaN	NaN	NaN	NaN	NaN	NaN	NaN	NaN	NaN
NaN	NaN	NaN	NaN	NaN	NaN	NaN	NaN	NaN
NaN	NaN	NaN	NaN	NaN	NaN	NaN	NaN	NaN
NaN	NaN	NaN	NaN	NaN	NaN	NaN	NaN	NaN
NaN	NaN	NaN	NaN	NaN	NaN	NaN	NaN	NaN
NaN	NaN	NaN	NaN	NaN	NaN	NaN	NaN	NaN
NaN	NaN	NaN	NaN	NaN	NaN	NaN	NaN	NaN
NaN	NaN	NaN	NaN	NaN	NaN	NaN	NaN	NaN
NaN	NaN	20,2787	20,2302	NaN	NaN	NaN	20,1183	NaN
NaN	NaN	NaN	NaN	NaN	NaN	NaN	NaN	NaN
NaN	NaN	NaN	NaN	NaN	NaN	NaN	NaN	NaN
NaN	NaN	NaN	NaN	NaN	NaN	NaN	NaN	NaN
19,8818	NaN	NaN	NaN	NaN	NaN	NaN	NaN	NaN
22,4787	22,5431	NaN	21,7744	NaN	NaN	NaN	NaN	22,0593
NaN	NaN	NaN	NaN	NaN	NaN	NaN	NaN	NaN
NaN	NaN	NaN	NaN	NaN	NaN	NaN	NaN	NaN
NaN	NaN	NaN	NaN	NaN	NaN	NaN	NaN	NaN
23,0715	23,0118	21,8144	21,992	23,6087	23,594	21,9445	21,8959	21,2492
NaN	NaN	NaN	NaN	NaN	NaN	NaN	NaN	NaN
NaN	NaN	NaN	NaN	NaN	NaN	NaN	NaN	NaN
NaN	NaN	NaN	NaN	NaN	NaN	NaN	NaN	NaN
NaN	NaN	NaN	19,8943	NaN	NaN	NaN	NaN	NaN
NaN	NaN	NaN	NaN	NaN	NaN	NaN	NaN	NaN
NaN	NaN	NaN	NaN	NaN	NaN	NaN	NaN	NaN
NaN	NaN	NaN	NaN	NaN	NaN	NaN	NaN	NaN
NaN	NaN	NaN	NaN	NaN	NaN	NaN	NaN	NaN
NaN	NaN	NaN	NaN	NaN	NaN	NaN	NaN	NaN
23,994	23,9803	23,7234	23,631	23,8179	23,8035	23,5326	23,6482	23,5086
20,5732	20,7027	NaN	NaN	20,751	20,714	21,1092	21,2743	21,4893
NaN	NaN	NaN	NaN	NaN	NaN	NaN	NaN	NaN
22,3343	22,0159	NaN	NaN	22,9917	22,933	21,8162	NaN	NaN
20,0079	21,0038	NaN	NaN	21,514	20,8858	20,5263	NaN	NaN
NaN	NaN	NaN	NaN	NaN	NaN	NaN	NaN	NaN

NaN	NaN	NaN	NaN	NaN	NaN	NaN	NaN	NaN
NaN	NaN	NaN	NaN	NaN	NaN	NaN	NaN	NaN
20,5854	NaN	20,1755	NaN	NaN	NaN	NaN	NaN	20,4207
21,0367	20,6608	NaN	NaN	NaN	NaN	NaN	NaN	NaN
NaN	NaN	NaN	NaN	NaN	NaN	NaN	NaN	NaN
26,2118	26,0552	25,3382	25,2039	26,9557	26,9069	25,7577	25,6157	25,3974
NaN	NaN	NaN	NaN	NaN	NaN	NaN	NaN	NaN
NaN	NaN	NaN	NaN	NaN	NaN	NaN	NaN	NaN
NaN	NaN	NaN	NaN	NaN	NaN	NaN	NaN	NaN
NaN	NaN	NaN	NaN	NaN	NaN	NaN	NaN	NaN
NaN	NaN	NaN	NaN	NaN	NaN	NaN	NaN	NaN
NaN	NaN	NaN	NaN	NaN	NaN	NaN	NaN	NaN
NaN	NaN	NaN	NaN	NaN	NaN	NaN	NaN	NaN
NaN	NaN	NaN	NaN	NaN	NaN	NaN	NaN	NaN
23,438	23,0933	24,8266	23,4964	25,0204	24,7337	23,1015	23,2836	23,7927
NaN	NaN	NaN	NaN	NaN	NaN	NaN	NaN	NaN
21,1847	20,8473	21,3047	21,3342	21,7691	21,5667	20,9117	21,6523	20,8057
NaN	NaN	NaN	NaN	NaN	NaN	NaN	NaN	NaN
22,3217	22,356	21,8589	21,8793	22,4602	22,4369	21,9043	22,0234	NaN
NaN	NaN	NaN	NaN	NaN	NaN	NaN	NaN	NaN
NaN	NaN	NaN	NaN	NaN	NaN	NaN	NaN	NaN
22,5489	NaN	NaN	22,0901	NaN	NaN	NaN	NaN	NaN
NaN	21,8545	NaN	NaN	NaN	NaN	NaN	NaN	21,7612
NaN	NaN	NaN	NaN	NaN	NaN	NaN	NaN	NaN
NaN	NaN	NaN	NaN	NaN	NaN	NaN	NaN	NaN
NaN	NaN	NaN	NaN	NaN	NaN	NaN	NaN	NaN
NaN	NaN	NaN	NaN	NaN	NaN	NaN	NaN	NaN
NaN	NaN	NaN	NaN	NaN	NaN	NaN	NaN	NaN
NaN	NaN	21,1212	21,5352	NaN	NaN	NaN	NaN	20,4974
NaN	NaN	NaN	NaN	NaN	NaN	NaN	18,5943	NaN
NaN	NaN	NaN	NaN	NaN	NaN	NaN	NaN	NaN
NaN	NaN	NaN	NaN	NaN	NaN	NaN	NaN	NaN
NaN	NaN	NaN	NaN	NaN	NaN	NaN	NaN	NaN
NaN	NaN	NaN	NaN	NaN	NaN	NaN	NaN	NaN
NaN	NaN	NaN	NaN	NaN	NaN	NaN	NaN	NaN
NaN	NaN	NaN	NaN	NaN	NaN	NaN	NaN	NaN
NaN	NaN	NaN	NaN	NaN	NaN	NaN	NaN	NaN
NaN	NaN	NaN	NaN	NaN	NaN	NaN	NaN	NaN
NaN	NaN	NaN	NaN	NaN	NaN	NaN	NaN	NaN
NaN	NaN	NaN	NaN	NaN	NaN	NaN	NaN	NaN
NaN	NaN	NaN	NaN	NaN	NaN	NaN	NaN	NaN
NaN	NaN	NaN	NaN	NaN	NaN	NaN	NaN	NaN
NaN	NaN	NaN	NaN	NaN	NaN	NaN	NaN	NaN
NaN	NaN	NaN	NaN	NaN	NaN	NaN	NaN	NaN
NaN	NaN	NaN	NaN	NaN	NaN	NaN	NaN	NaN
NaN	NaN	NaN	NaN	NaN	NaN	NaN	NaN	NaN
NaN	NaN	NaN	NaN	NaN	NaN	NaN	NaN	NaN
NaN	NaN	NaN	NaN	NaN	NaN	NaN	NaN	NaN
NaN	NaN	NaN	NaN	NaN	NaN	NaN	NaN	NaN
20,1587	NaN	19,8526	NaN	NaN	NaN	NaN	19,6465	19,883
NaN	NaN	NaN	NaN	NaN	NaN	NaN	NaN	NaN
NaN	NaN	NaN	NaN	NaN	NaN	NaN	NaN	NaN

NaN	21,0105	NaN	NaN	NaN	NaN	NaN	NaN	NaN
NaN	NaN	NaN	NaN	NaN	NaN	NaN	NaN	NaN
NaN	NaN	21,2044	NaN	NaN	NaN	20,5331	NaN	NaN
NaN	NaN	NaN	NaN	NaN	NaN	NaN	NaN	NaN
NaN	NaN	NaN	NaN	NaN	NaN	NaN	NaN	NaN
NaN	NaN	NaN	NaN	NaN	NaN	NaN	NaN	NaN
NaN	NaN	NaN	NaN	NaN	NaN	NaN	NaN	NaN
NaN	NaN	NaN	NaN	NaN	NaN	NaN	NaN	NaN
NaN	NaN	NaN	NaN	NaN	NaN	NaN	NaN	NaN
NaN	NaN	NaN	NaN	NaN	NaN	NaN	NaN	NaN
NaN	NaN	NaN	NaN	NaN	NaN	19,7896	NaN	NaN
NaN	NaN	NaN	NaN	NaN	NaN	NaN	NaN	NaN
NaN	NaN	NaN	NaN	NaN	NaN	NaN	NaN	NaN
NaN	NaN	NaN	NaN	NaN	NaN	NaN	NaN	NaN
NaN	NaN	NaN	NaN	NaN	NaN	NaN	NaN	NaN
NaN	NaN	NaN	NaN	NaN	NaN	NaN	NaN	NaN
NaN	NaN	NaN	NaN	NaN	NaN	20,827	21,0162	NaN
NaN	NaN	NaN	NaN	NaN	NaN	NaN	NaN	NaN
NaN	NaN	NaN	NaN	NaN	NaN	NaN	NaN	NaN
NaN	20,0232	20,7326	20,6941	NaN	NaN	NaN	NaN	20,2328
NaN	NaN	NaN	NaN	NaN	NaN	NaN	NaN	NaN
NaN	NaN	NaN	NaN	NaN	NaN	NaN	NaN	NaN
NaN	NaN	NaN	NaN	NaN	NaN	NaN	NaN	NaN
NaN	NaN	NaN	NaN	NaN	NaN	NaN	NaN	NaN
NaN	NaN	NaN	NaN	NaN	NaN	NaN	NaN	NaN
NaN	NaN	NaN	NaN	NaN	NaN	NaN	NaN	NaN
NaN	23,8467	25,2001	25,2338	24,6514	24,8175	NaN	NaN	23,9209
NaN	NaN	NaN	NaN	NaN	NaN	NaN	NaN	NaN
NaN	21,9996	22,2751	NaN	NaN	NaN	23,3903	22,325	NaN
NaN	NaN	NaN	19,9036	NaN	20,9762	19,8097	19,7322	20,9076
NaN	NaN	NaN	NaN	NaN	NaN	NaN	NaN	NaN
NaN	NaN	NaN	NaN	NaN	NaN	NaN	NaN	NaN
NaN	NaN	NaN	NaN	NaN	NaN	NaN	NaN	NaN
NaN	NaN	NaN	NaN	NaN	NaN	NaN	NaN	NaN
NaN	NaN	NaN	NaN	NaN	NaN	NaN	NaN	19,6656
NaN	NaN	NaN	NaN	NaN	NaN	NaN	NaN	NaN
NaN	22,8356	NaN	NaN	NaN	NaN	NaN	NaN	NaN
NaN	NaN	NaN	NaN	NaN	NaN	NaN	NaN	NaN
NaN	NaN	NaN	NaN	NaN	NaN	NaN	NaN	NaN
NaN	NaN	NaN	NaN	NaN	NaN	NaN	NaN	NaN
23,4211	23,8479	22,298	22,1379	23,7768	23,2114	21,072	NaN	23,9215
NaN	NaN	NaN	NaN	NaN	22,2142	NaN	NaN	NaN
NaN	NaN	NaN	NaN	NaN	NaN	NaN	NaN	NaN
NaN	NaN	NaN	NaN	NaN	NaN	NaN	NaN	NaN
NaN	NaN	NaN	NaN	NaN	NaN	NaN	NaN	NaN
NaN	NaN	NaN	NaN	NaN	NaN	NaN	NaN	NaN
NaN	NaN	NaN	NaN	NaN	NaN	NaN	NaN	NaN
23,6914	23,8882	NaN	NaN	NaN	23,4196	NaN	NaN	23,1646
NaN	NaN	NaN	NaN	NaN	NaN	NaN	NaN	NaN
NaN	NaN	NaN	NaN	NaN	NaN	NaN	NaN	NaN
NaN	NaN	NaN	NaN	NaN	NaN	NaN	NaN	NaN
NaN	NaN	NaN	NaN	NaN	NaN	NaN	NaN	NaN

21,2933	21,4043	NaN	20,5591	NaN	NaN	NaN	NaN	20,8893
NaN	NaN	NaN	NaN	NaN	NaN	NaN	NaN	NaN
NaN	NaN	22,919	NaN	25,8425	25,934	NaN	NaN	23,5039
27,0454	27,0272	30,8502	30,9678	31,4909	31,2221	30,4291	30,4613	31,3069
NaN	NaN	NaN	NaN	NaN	20,6586	20,221	NaN	NaN
20,6176	NaN	NaN	NaN	NaN	NaN	NaN	NaN	NaN
NaN	NaN	NaN	NaN	NaN	NaN	NaN	NaN	NaN
NaN	NaN	NaN	NaN	NaN	NaN	NaN	NaN	NaN
NaN	NaN	NaN	NaN	NaN	NaN	NaN	NaN	NaN
NaN	NaN	NaN	NaN	NaN	NaN	NaN	NaN	NaN
NaN	NaN	NaN	NaN	NaN	NaN	NaN	NaN	NaN
19,9774	NaN	NaN	NaN	19,9725	NaN	NaN	20,1862	NaN
24,0225	24,043	23,3226	23,7627	25,4448	25,3386	24,5105	24,4641	23,4684
19,8195	NaN	NaN	NaN	NaN	19,8471	NaN	NaN	NaN
NaN	NaN	NaN	NaN	NaN	NaN	NaN	NaN	NaN
NaN	NaN	23,855	23,8006	24,5826	24,2535	NaN	NaN	NaN
25,0959	25,7012	NaN	NaN	NaN	NaN	NaN	NaN	24,1511
NaN	NaN	NaN	NaN	NaN	NaN	NaN	NaN	NaN
NaN	NaN	NaN	NaN	NaN	NaN	NaN	NaN	19,1343
21,0047	21,0861	20,1892	20,7121	NaN	NaN	NaN	20,14	21,4644
NaN	NaN	NaN	NaN	NaN	NaN	NaN	19,9287	NaN
20,4923	NaN	NaN	NaN	NaN	NaN	NaN	NaN	NaN
22,204	21,7075	NaN	NaN	NaN	NaN	NaN	NaN	NaN
NaN	NaN	NaN	NaN	NaN	NaN	NaN	NaN	NaN
NaN	NaN	NaN	NaN	NaN	NaN	NaN	NaN	NaN
20,2266	NaN	NaN	NaN	NaN	20,4572	NaN	NaN	NaN
NaN	NaN	NaN	NaN	NaN	NaN	NaN	NaN	NaN
NaN	NaN	NaN	NaN	NaN	NaN	NaN	NaN	NaN
NaN	NaN	NaN	NaN	NaN	NaN	NaN	NaN	NaN
NaN	NaN	20,8263	20,8238	NaN	NaN	NaN	NaN	NaN
NaN	NaN	NaN	NaN	NaN	NaN	NaN	NaN	NaN
NaN	NaN	NaN	NaN	NaN	NaN	NaN	NaN	NaN
26,2108	26,0492	25,6753	25,612	27,077	26,8332	26,1927	26,5092	25,6908
NaN	NaN	NaN	NaN	NaN	NaN	NaN	NaN	NaN
22,6672	23,2255	22,0975	22,1536	NaN	NaN	22,1458	22,0925	22,7597
NaN	NaN	NaN	NaN	NaN	NaN	NaN	NaN	NaN
NaN	NaN	NaN	NaN	NaN	NaN	NaN	NaN	NaN
NaN	NaN	20,8345	NaN	NaN	NaN	21,5581	21,4194	NaN
NaN	NaN	NaN	NaN	NaN	NaN	20,2197	20,6246	20,2535
NaN	NaN	NaN	NaN	NaN	NaN	NaN	NaN	NaN
NaN	NaN	NaN	NaN	NaN	NaN	NaN	NaN	NaN
NaN	22,8752	NaN	21,5444	22,9893	22,9968	23,153	23,0814	22,4188
NaN	NaN	26,117	26,1466	27,0739	26,8889	25,5416	25,6605	NaN
NaN	NaN	NaN	NaN	NaN	NaN	NaN	NaN	NaN
NaN	NaN	NaN	NaN	NaN	NaN	NaN	NaN	NaN
NaN	NaN	NaN	NaN	NaN	NaN	NaN	NaN	NaN
24,922	NaN	NaN	NaN	25,4106	25,6624	25,1163	25,4146	24,5189
NaN	NaN	NaN	NaN	NaN	NaN	NaN	NaN	NaN
NaN	NaN	NaN	NaN	NaN	NaN	NaN	NaN	NaN
NaN	21,4285	NaN	NaN	NaN	NaN	NaN	NaN	21,5736
NaN	NaN	NaN	NaN	NaN	NaN	NaN	NaN	NaN
NaN	NaN	NaN	NaN	NaN	NaN	NaN	NaN	NaN

NaN	NaN	NaN	NaN	NaN	NaN	NaN	NaN	NaN
22,0922	21,9748	22,1239	NaN	22,9296	22,6708	23,269	23,2957	NaN
21,1111	21,2881	20,9872	21,2897	21,7737	NaN	21,8502	22,2784	21,8039
NaN	NaN	NaN	NaN	NaN	21,1803	21,4954	21,4719	NaN
NaN	NaN	NaN	NaN	NaN	NaN	NaN	NaN	NaN
NaN	NaN	NaN	NaN	NaN	NaN	NaN	NaN	NaN
NaN	NaN	NaN	NaN	NaN	NaN	22,2233	NaN	NaN
22,6448	22,2239	22,5055	22,5187	NaN	20,791	22,4366	22,2819	23,4695
NaN	NaN	NaN	NaN	NaN	NaN	NaN	NaN	NaN
NaN	NaN	NaN	NaN	NaN	NaN	NaN	NaN	NaN
NaN	NaN	NaN	NaN	NaN	NaN	NaN	NaN	NaN
26,0397	26,1206	25,7847	25,7447	26,5467	26,5093	25,8978	25,7736	NaN
NaN	NaN	NaN	NaN	NaN	NaN	NaN	NaN	21,389
21,4738	21,9804	NaN	NaN	NaN	NaN	NaN	NaN	NaN
NaN	NaN	NaN	NaN	NaN	NaN	NaN	NaN	NaN
21,4125	21,5993	23,0018	23,27	NaN	NaN	21,7848	21,9083	21,4758
21,7133	21,3914	23,6731	23,5815	25,3872	25,0779	22,7576	22,8617	23,789

LFQ intensi	LFQ intensi	LFQ intensi	LFQ intensi	LFQ intensi	LFQ intensi	LFQ intensi	LFQ intensi	LFQ intensi
31,2952	31,9014	31,9794	32,4354	32,431	31,8726	31,878	31,5618	31,573
34,0363	34,9122	34,9678	32,8421	32,8778	33,5837	33,6699	34,0274	34,0322
26,7666	23,9299	23,9719	25,3439	25,5144	28,5549	28,6345	28,1915	28,1429
21,273	20,0455	16,994	16,2947	16,932	16,7926	18,4989	16,4178	NaN
26,6994	27,5366	27,6044	26,5601	26,5972	27,0879	27,1115	27,2146	27,2149
25,9697	24,4611	24,6312	22,871	22,8968	25,6816	25,6492	26,1495	26,1611
23,8023	NaN	20,011	23,3435	23,6405	24,2298	23,6792	22,1372	18,6544
28,9587	29,0001	29,0757	26,9176	26,8328	27,506	27,5584	28,4184	28,3623
29,3175	30,0742	30,1339	28,8238	28,7895	29,6068	29,5178	29,2223	29,1611
28,2016	28,5278	28,5068	27,5664	27,7077	28,6853	28,7295	28,6084	28,583
18,9314	NaN	NaN	19,1568	17,616	18,9829	17,8216	18,0494	NaN
34,3475	34,6219	34,4488	33,4476	33,4632	34,2248	34,3025	35,5842	35,542
31,5072	32,0661	31,9863	32,0715	32,087	32,5668	32,6159	32,5556	32,5484
29,5409	30,5723	30,5317	27,899	27,8171	28,0044	27,9963	28,284	28,3078
26,8884	23,6581	23,5973	25,5455	25,5607	26,2635	26,2967	26,5202	26,4724
28,2627	25,8622	25,9663	29,6241	29,5477	27,5039	27,4849	27,869	27,7918
27,6245	26,7661	26,9154	24,985	24,7231	27,2867	27,3818	27,8774	27,7854
25,2204	26,6148	26,7116	27,7459	27,6364	28,1646	28,2677	26,5143	26,4239
25,7955	25,9696	26,2249	26,2184	26,0746	26,6779	26,7406	25,895	25,7435
23,9586	19,7399	21,1804	NaN	NaN	23,5023	23,3977	23,8801	23,944
25,7438	27,2112	27,1702	27,6552	27,7035	28,4866	28,4624	26,6126	26,7339
26,1201	24,0501	24,1688	24,4923	24,5366	25,3648	25,4961	25,6429	25,6575
27,5797	27,6635	27,7898	28,6953	28,71	27,0425	26,7563	28,2276	28,1731
26,5191	24,9294	24,9296	24,4826	24,713	26,9644	26,9163	26,3996	26,4425
28,1344	29,226	29,1102	26,6514	26,6398	26,2759	26,2212	26,4918	26,506
25,5044	26,1852	26,2458	25,6374	25,3882	26,5442	26,6893	25,6471	25,5363
27,137	28,2489	28,2841	25,9982	25,9435	26,0205	25,9293	26,7406	26,7681
24,117	21,0098	22,0296	NaN	NaN	23,1412	23,078	23,9653	23,9753
26,5583	27,0685	27,003	24,5193	24,3929	27,186	27,152	26,6886	26,649
25,2821	26,0052	26,1783	24,3129	24,4026	25,5483	25,5377	25,2335	25,3615
26,3136	25,7944	25,911	24,716	24,7128	26,4506	26,5569	26,4774	26,431
28,8969	27,7858	27,8923	29,7281	29,8071	29,3616	29,2371	27,2904	27,2113
26,9933	26,5448	26,5197	26,036	26,0773	26,0789	25,9272	26,8751	26,828
26,1007	24,7493	24,4318	27,0459	26,8377	25,4634	25,4474	25,6029	25,593
23,8609	NaN	NaN	21,9537	21,5309	23,3193	23,3822	23,71	23,8402
26,8339	26,0901	26,2668	27,4637	27,4037	27,3829	27,5372	27,1147	27,0965
28,791	27,6514	27,8008	28,8958	28,9046	29,1442	29,1546	28,3942	28,5724
25,7109	25,6003	25,7303	23,3843	23,4656	26,19	26,2169	26,3936	26,5487
26,204	26,6845	26,6651	24,4013	24,5629	25,5347	25,4109	26,4636	26,4909
27,3873	25,7911	25,9517	25,8381	25,8485	27,4554	27,2813	26,9876	26,9704
28,4844	29,0949	29,1628	26,7436	26,8529	27,4266	27,4143	27,8126	27,7849
26,851	25,4898	25,4678	27,295	27,2621	26,9636	26,8002	26,5009	26,5314
26,948	25,8672	25,9338	28,0696	28,0555	27,385	27,3903	26,1095	26,196
26,4846	25,6053	25,6601	25,085	25,1083	26,5357	26,4109	26,2394	26,1664
25,3696	24,4305	24,3056	25,541	25,4617	25,5584	25,4379	25,104	25,1592
27,3322	26,359	26,4065	25,8751	25,7555	27,4163	27,4242	27,2335	27,3508
25,7224	24,6827	24,7631	26,4039	26,4773	25,9536	26,0618	25,2244	25,2053
23,6163	22,2052	21,5275	NaN	19,9649	23,3518	23,6318	23,9025	23,8267
28,2299	30,5958	30,6001	29,4176	29,419	29,9011	29,8464	29,8826	29,8485
19,9661	21,2239	21,8503	19,6985	19,6813	16,4626	20,8018	NaN	16,4144
24,3761	24,3797	24,2445	23,0743	23,0431	24,4007	23,8739	24,5369	24,5328

27,1213	25,5269	25,5666	25,6058	25,5461	26,8198	26,9682	26,8292	26,7103
32,3633	32,6462	32,7168	32,9522	33,033	33,4878	33,4072	33,7467	33,6441
20,8103	22,7004	23,8652	21,026	18,7288	21,4845	NaN	26,0581	25,8781
25,7323	25,8376	25,8364	22,0318	20,7957	26,0026	26,067	25,913	25,6518
NaN	NaN	NaN	NaN	NaN	17,772	NaN	NaN	NaN
26,295	25,1774	25,2586	26,4402	26,3625	26,4181	26,453	25,4813	25,3301
24,5925	21,6776	23,0844	24,9688	24,802	24,5582	24,3638	23,8937	24,0088
27,06	26,0799	26,1311	26,6855	26,8061	26,8823	26,844	27,0334	27,0969
26,9785	26,7442	26,6709	29,4123	29,4124	27,839	27,9183	27,3169	27,1907
24,4488	22,6116	22,6852	25,2666	25,2729	24,8858	24,7523	23,2811	23,211
24,8795	22,5647	22,7995	21,762	21,4674	23,9088	23,7717	24,4574	24,5449
24,439	24,5719	24,6262	25,3683	25,2659	24,5195	24,3632	23,7951	23,8401
27,5592	29,1041	29,1432	26,5194	26,612	26,8212	26,5757	27,6466	27,6601
24,8925	23,0678	23,2042	23,5104	22,8445	24,5091	24,2299	24,9531	24,6798
23,4516	24,6762	24,6622	23,4238	23,2459	23,9061	24,0676	23,4608	23,6067
24,1344	25,3669	25,4154	23,6389	23,5763	24,4218	24,3072	23,9498	23,7662
27,7672	26,5906	26,5976	27,5358	27,4624	27,321	27,3277	27,3644	27,291
23,5591	21,6984	21,4692	23,5746	22,7362	22,2106	21,5331	22,8172	22,466
31,0215	29,2043	29,2142	28,9085	29,0056	29,8629	29,8494	30,557	30,6354
24,2361	22,8763	22,641	24,2079	24,1079	23,8389	23,6594	23,7719	23,4713
23,6856	22,1277	21,8597	21,9925	22,2243	23,6971	23,3878	23,1984	23,2424
25,4973	20,4843	20,4478	21,6235	NaN	24,6293	24,5026	24,9527	25,0311
25,0473	23,532	23,1042	23,774	23,4197	24,9525	25,0972	24,9964	24,817
27,9454	28,3585	28,3905	28,8556	28,8073	28,524	28,3901	28,7543	28,7081
23,3799	24,5934	24,7454	23,8731	23,7969	23,6413	23,9218	23,5816	23,3719
28,8313	26,5195	26,4102	29,2825	29,2326	27,968	27,8041	27,3101	27,1995
24,5587	22,9177	22,9637	22,8305	23,3973	24,7448	24,4218	24,5737	24,4649
24,8984	23,574	23,4556	22,4991	22,5975	24,2353	24,2053	24,8136	24,8707
27,6163	26,2293	26,3132	27,1619	27,219	27,6385	27,5728	27,1746	27,2302
25,9349	26,2751	26,2428	25,5037	25,5254	26,4052	26,5617	26,0851	26,1081
26,7104	26,0589	26,0532	27,9107	27,8422	26,6728	26,9423	26,6033	26,6382
17,0109	18,5553	17,5811	NaN	NaN	18,4942	18,3356	16,627	NaN
22,0186	23,7321	23,5827	22,3775	21,9695	22,1386	21,7784	23,3969	23,0021
26,3122	27,0098	26,8957	24,7441	24,8749	26,1226	26,197	26,7035	26,4837
23,3257	24,0764	24,2258	23,9652	23,9171	23,228	23,4022	23,3864	23,1825
21,3741	24,3865	24,5758	22,0203	20,8849	21,8679	22,1252	21,6897	21,8017
28,6817	27,293	27,2834	27,7707	27,7406	28,1931	28,0119	27,7556	27,6358
25,3088	24,646	24,6488	26,1182	26,2456	23,1782	23,1137	22,8076	22,6454
27,087	28,3668	28,4022	29	29,1425	26,5832	26,666	28,0632	28,1259
22,4668	23,0518	23,2554	26,4034	26,3205	24,0561	24,5552	22,6867	22,5567
28,6569	30,1778	30,2514	28,8044	28,8204	29,7126	29,5041	29,0343	28,8474
21,3328	NaN	20,2007	19,0698	NaN	20,6899	19,8536	21,1613	NaN
24,9008	25,3359	25,379	25,0115	25,0484	25,0314	24,9237	25,4053	25,3321
23,7866	22,6574	22,518	NaN	NaN	22,2051	22,5106	23,756	23,8471
25,9745	26,1167	26,1636	26,729	26,84	24,414	24,4895	25,8393	25,7549
26,4617	25,3648	25,3918	27,1178	27,2656	26,9992	26,9228	26,0412	25,931
27,4764	27,9115	28,1606	25,3809	25,2801	27,1094	27,1384	26,5737	26,5035
26,1089	27,5296	27,5596	26,0521	25,9629	26,3584	26,4091	26,317	26,261
28,1402	26,1823	26,1452	27,6855	27,5646	27,4563	27,4144	27,6947	27,6657
30,8874	31,5518	31,4839	31,8154	31,8943	31,9035	32,0035	31,8838	31,9414
32,8784	31,5959	31,6575	33,9292	33,866	30,0289	29,9959	33,3128	33,2275
27,9237	25,9615	25,7922	28,3137	28,2627	26,7133	26,8541	27,1717	27,1372

23,7757	22,6101	22,4813	22,6309	22,7521	23,7077	23,6128	23,5686	23,8048
NaN	19,9408	NaN	NaN	NaN	23,7052	22,7194	22,449	22,1967
19,902	22,8127	23,026	NaN	NaN	23,1653	23,2568	24,2765	24,376
24,351	22,607	22,3106	23,7984	23,8262	23,7634	23,7488	24,0669	24,148
26,2149	25,34	25,4819	27,3339	27,3758	25,8757	25,7126	24,8905	24,9136
26,1446	24,7026	24,8447	23,0717	22,841	25,6781	25,7922	26,1274	26,0778
24,3525	22,4174	22,6702	22,5989	22,6862	23,846	23,6463	24,3907	24,089
24,0334	24,9435	24,9299	24,5189	24,7469	25,1359	25,1445	25,2522	25,2862
26,6647	25,246	25,3468	25,8761	26,0704	27,1582	27,0503	26,3818	26,4358
28,9583	28,0891	28,024	26,7142	26,5406	28,0216	27,9474	29,1769	29,1043
NaN	NaN	NaN	NaN	NaN	NaN	NaN	NaN	NaN
22,8638	23,5935	23,7076	23,7743	23,7726	22,9342	22,856	23,0535	23,1196
26,9062	26,0553	26,0199	27,3911	27,2183	27,1816	27,1614	26,4968	26,5381
25,7093	24,1526	24,2042	26,4522	26,4705	25,7891	25,7048	25,2082	25,3258
24,8791	23,4109	23,1803	23,4117	23,3013	23,9533	23,9137	24,6784	24,8116
26,0063	26,4301	26,5012	26,5089	26,5409	26,1035	26,3084	26,7841	26,8301
22,8042	22,1187	22,0364	21,7648	21,5262	22,6258	22,7795	22,2717	NaN
24,3992	21,6233	21,5245	25,248	25,2088	23,9324	23,7267	23,4638	23,5491
22,6084	23,343	22,3634	22,0436	21,8973	23,0495	22,6906	23,3301	23,2838
23,1615	21,0362	21,2339	NaN	NaN	22,936	22,7175	23,6524	23,4425
23,468	22,1166	22,2901	22,5836	22,8498	21,7038	21,4665	22,7661	22,7941
24,4303	24,734	24,6099	24,3649	24,2351	24,046	23,963	25,0195	24,9872
19,347	NaN	19,8753	NaN	18,8523	18,9698	NaN	NaN	NaN
28,417	28,0387	28,0751	28,8138	28,7819	27,2835	27,2913	26,4579	26,4878
22,3031	21,8829	21,5464	NaN	NaN	22,8396	23,2952	NaN	NaN
25,6183	23,7465	23,7627	26,5353	26,5259	24,787	24,9066	24,8516	25,0004
27,461	26,0024	25,8511	24,1842	23,9657	26,6815	26,9054	26,9923	26,9293
NaN	NaN	NaN	19,1977	20,1494	23,2267	23,2289	NaN	NaN
23,8761	20,8376	22,0911	23,7781	24,0073	23,4405	23,5551	23,406	22,9431
23,6051	24,946	24,7656	23,6531	23,5724	23,6416	23,3153	23,4162	23,5159
24,6702	23,3998	23,1766	26,0553	26,1775	24,8223	24,7549	24,1338	23,9447
29,5534	30,0955	29,9598	30,286	30,2826	30,5627	30,4414	30,6467	30,4581
24,6223	25,7925	25,6113	23,4981	23,5148	24,8608	24,3715	25,0318	24,9596
25,6692	25,0451	24,8716	25,0316	25,2781	25,3043	25,1814	25,4883	25,6287
25,6946	24,3307	24,3947	25,7616	25,8009	25,0008	24,9236	25,2905	24,9234
22,7138	22,1613	21,7164	21,1311	21,5724	22,3102	21,8421	22,7093	22,0637
25,5814	24,5042	24,4731	26,2008	26,4731	25,2433	25,3099	24,6278	24,3495
28,5486	29,0472	29,1839	29,3009	29,3716	29,6575	29,6804	29,1783	29,3549
27,9072	28,6119	28,6083	29,1413	29,1604	27,442	27,6047	28,6598	28,57
NaN	NaN	NaN	23,1043	23,1353	19,4646	NaN	21,1024	21,0078
23,0461	24,6336	24,6129	24,4565	24,634	22,2912	22,0376	22,9287	23,2686
24,3271	33,8322	33,4757	25,7765	25,8372	26,3342	26,12	24,9783	24,8097
22,3204	25,8372	26,023	24,8916	24,8132	23,4974	23,3864	23,5922	24,1049
24,3258	22,5094	22,643	22,8562	22,8843	24,14	24,0362	24,1264	23,8436
24,494	23,2819	23,3624	23,7415	23,623	24,6137	24,3186	24,4036	24,5472
31,829	30,7775	30,8658	32,7396	32,61	30,5857	30,7975	31,7503	31,8734
24,2824	23,4556	23,1423	23,1179	22,9779	24,4068	24,3343	23,945	23,8881
24,7349	20,976	19,9472	23,6263	23,5043	24,487	24,4185	22,3116	22,1472
23,8896	21,8873	21,9907	22,2561	22,4746	24,6148	24,5309	23,6361	23,6742
23,554	22,1967	22,6655	23,7358	24,005	22,7796	23,6856	20,9935	22,1216
24,7436	23,4779	23,137	24,8099	24,7942	24,648	24,6013	23,6276	23,8642
23,457	20,6847	21,3321	22,9004	22,8962	22,7422	22,926	22,9705	23,5162

23,2647	21,5269	21,2391	23,3724	23,543	23,4768	22,7783	22,735	22,7052
27,3769	29,9232	29,939	29,4482	29,5031	28,3681	28,3284	29,5646	29,558
21,5971	NaN	NaN	NaN	NaN	20,2762	20,5107	NaN	20,8448
21,7289	23,6469	23,5996	22,2025	22,0066	23,1018	23,0804	22,7635	22,9986
23,1558	22,085	21,7825	22,8417	22,6835	23,0347	23,2022	23,1182	22,9826
21,3109	NaN	NaN	21,7551	21,2374	21,9097	22,2204	21,2435	21,5914
24,5716	29,8637	30,0025	26,0711	26,0789	26,5779	26,9339	27,4697	27,5144
27,3694	25,4126	25,4572	27,6094	27,6039	26,7348	26,6914	27,6012	27,2817
24,1125	24,0305	23,8614	22,1776	22,3074	25,0762	25,3652	24,1943	24,4063
31,8467	31,6787	31,8775	32,9936	32,9638	31,3564	31,3866	32,7795	32,9717
22,4277	22,3678	22,6044	23,0148	22,9851	21,9561	21,6844	22,935	22,9103
24,8506	22,7787	22,6281	25,1969	25,3815	24,5381	24,7686	24,3225	23,6873
29,1076	30,3836	30,3259	30,1977	30,2059	27,7908	27,6805	28,933	28,9548
25,0054	23,5992	23,1909	25,1754	25,124	24,3604	24,3049	24,1199	24,0402
21,3831	23,3386	23,5301	22,5267	22,3727	21,9432	22,1817	22,0299	22,6236
29,3936	29,9879	30,0828	29,1774	29,327	30,5029	30,4547	30,5981	30,5153
24,5037	23,2839	23,1127	23,2167	23,3406	24,2777	24,1156	24,0423	24,0358
22,8701	22,6151	22,389	21,5001	21,5543	21,9762	22,1502	22,5978	23,2874
20,9748	NaN	NaN	NaN	NaN	NaN	NaN	21,3277	NaN
NaN	NaN	NaN	NaN	NaN	18,5162	NaN	NaN	NaN
22,0922	NaN	20,2276	22,9993	22,7593	22,5247	22,2046	20,4298	NaN
22,4725	20,4623	21,0079	22,8423	22,3483	21,1969	21,5414	22,4042	22,4962
24,2561	22,2974	22,6278	22,9594	22,9036	23,8915	23,9631	24,1723	24,2201
22,1632	NaN	NaN	21,948	21,8498	22,5227	22,1292	21,5012	21,981
22,4232	23,2002	23,1964	23,1844	23,434	21,7718	22,0017	22,4577	22,5173
24,066	23,5783	23,6107	23,6966	23,5906	24,1787	24,2703	24,0051	24,2431
23,1028	24,0769	24,4886	22,073	22,271	22,8158	23,271	22,8261	22,5273
28,6053	29,4744	29,4298	28,69	28,6991	27,3588	27,3583	27,8148	27,6958
24,495	23,5068	23,385	23,8306	23,7791	23,6205	23,4641	24,67	24,5438
22,9703	NaN	NaN	22,435	NaN	22,831	23,0096	23,2568	22,6088
30,1308	24,7982	24,9086	29,0169	29,1987	30,5579	30,6209	30,2519	30,2177
24,3024	23,6836	24,0218	23,9375	23,5946	24,423	24,3748	23,9235	24,0265
27,9902	26,9786	27,0394	25,8856	25,9993	27,509	27,5023	27,7654	27,9691
27,9463	26,4261	26,6464	28,4506	28,4574	27,537	27,3699	27,3003	27,4115
27,1808	25,9767	26,2323	25,9513	25,8214	26,0667	26,245	26,8045	26,7624
23,924	22,2439	22,1543	21,5217	22,4732	23,0139	23,3962	23,9662	24,0871
22,9773	21,3528	21,098	23,862	23,6944	22,3742	22,5382	23,4158	22,5201
23,6913	NaN	21,9946	23,2516	23,0295	23,2528	23,0818	22,8476	23,208
23,6875	21,2791	21,5603	23,5258	23,3264	23,4135	23,2433	23,5179	22,6384
22,7656	23,7861	23,312	21,2197	21,2648	21,7977	21,1083	22,6119	23,0221
24,2878	22,7914	22,6859	20,265	NaN	23,7215	24,0463	24,0221	23,6973
26,8014	24,6242	24,8227	23,939	24,1429	24,9753	25,1255	25,9826	26,0876
25,4583	24,8401	24,744	26,2262	26,0477	25,6096	25,6236	24,7169	24,7424
26,4242	27,0619	27,1892	27,2407	27,3229	26,1013	26,2481	27,2267	27,2255
23,366	23,5153	23,2823	24,1822	24,1042	23,5516	23,5409	22,7876	21,7482
21,2808	NaN	NaN	NaN	NaN	NaN	NaN	NaN	NaN
24,4761	24,5837	24,4727	23,5218	23,4785	24,0803	23,8309	24,4922	24,2468
23,7661	21,923	22,0278	NaN	NaN	23,2676	23,2009	23,6422	23,4975
20,549	20,6576	NaN	19,4951	19,63	21,0694	20,6436	NaN	NaN
23,8179	26,3035	26,1812	24,0589	24,3949	25,1396	25,3257	25,09	24,8859
24,2517	23,4022	23,4807	24,7575	24,6432	23,9177	24,0678	23,5805	23,3577
22,9362	NaN	NaN	21,1127	21,036	21,3202	21,6582	22,6909	22,4454

23,5323	22,5024	22,6581	23,8972	23,8585	23,2154	23,4471	22,208	23,1277
23,0494	NaN	NaN	NaN	NaN	22,4073	21,882	22,785	23,2306
23,707	22,5917	22,1543	24,8206	24,8832	23,5946	23,4224	23,4017	23,3512
23,3491	21,3549	20,718	21,0871	20,7768	22,22	22,355	23,2058	23,2704
20,7521	22,2667	NaN	22,5657	23,0728	20,0833	20,7152	23,2587	23,107
22,185	NaN	NaN	21,7079	21,8378	21,8754	22,306	21,9434	21,3109
21,3487	21,4911	21,2325	23,4416	23,7182	20,2391	20,8945	21,6452	21,8095
22,4206	NaN	NaN	23,1018	23,0993	21,8789	21,8591	21,7762	21,811
28,7569	32,0326	32,1096	31,6177	31,5873	29,9725	29,8928	31,4658	31,4507
25,0819	27,1693	27,0461	26,7003	26,6345	23,6543	23,83	26,5423	26,3082
22,4536	21,2903	21,2421	22,4326	21,7112	22,0661	22,0189	NaN	21,4148
22,4885	20,8371	21,2833	21,7461	21,589	22,8017	22,9355	21,9716	21,9752
27,0057	26,1539	26,0296	27,0294	27,0367	26,7892	26,7028	26,7272	26,736
26,7146	25,5416	25,5092	25,0207	25,4034	26,6855	26,8735	26,6588	26,6698
22,1512	NaN	NaN	NaN	21,5387	21,4918	21,5739	NaN	21,676
21,7404	22,6186	22,7172	20,4779	20,6574	20,3996	21,4254	21,9131	21,5557
23,5811	23,5092	22,9541	23,0277	23,2406	23,3547	23,0793	23,7583	23,8589
22,525	24,1981	24,0677	23,0409	22,9264	22,0233	21,8351	22,9702	22,7303
21,0098	20,3334	NaN	21,3299	20,9616	21,198	20,8571	NaN	20,7833
NaN	27,3694	27,4753	NaN	NaN	20,1101	19,3625	NaN	NaN
23,5715	23,1987	22,9961	24,8719	24,7425	23,7238	23,7909	23,0133	22,4333
30,5579	24,8832	25,1987	29,3504	29,5891	30,7683	30,7678	30,4475	30,3196
23,9843	24,2522	24,1884	25,2141	25,0704	22,4212	22,8761	24,172	24,1632
25,9805	24,2517	24,1769	27,5557	27,4532	25,4767	25,381	24,8685	24,9221
24,9216	24,1566	24,2282	25,1184	24,973	23,7982	23,7378	24,3867	24,3951
16,8424	NaN	NaN	NaN	NaN	17,2829	NaN	NaN	NaN
22,0924	NaN	NaN	22,156	22,2394	21,4967	21,1405	21,1925	20,8551
25,1389	24,1345	24,121	25,1103	25,4482	25,5155	25,4802	24,7206	24,953
23,4599	23,5014	23,4756	24,4072	24,4596	23,8064	23,3019	23,5039	23,2512
23,1098	21,7451	21,129	22,2062	22,3497	23,0754	22,9696	22,2941	22,3928
24,7096	23,3389	23,4978	24,8349	24,7655	24,3046	24,393	24,5132	24,3595
23,3732	NaN	22,5967	23,7159	23,7665	23,3303	23,3996	22,4005	22,6739
22,0173	22,8824	22,6039	21,0792	21,2403	NaN	NaN	22,2368	22,1607
27,4712	26,4562	26,6168	28,2735	28,42	25,6901	26,0973	27,7046	27,6115
22,6909	19,1525	20,5115	23,5503	23,3476	21,0453	21,2465	20,4766	20,5149
23,5145	22,1506	21,9564	NaN	NaN	22,9206	22,754	23,5748	24,2946
23,0815	21,1822	21,4058	21,0926	NaN	22,6424	22,5279	22,7514	23,0208
22,6499	23,2321	23,0666	23,619	23,3465	21,5478	21,986	23,1229	23,0754
23,52	22,1325	22,6008	22,5138	22,2452	23,8561	23,9548	23,2092	23,0764
24,0438	22,8678	22,8206	23,1234	23,3782	23,6337	23,9829	24,1197	23,9759
19,8326	20,4645	20,06	20,6226	NaN	19,8411	NaN	NaN	NaN
22,0739	21,8879	21,7149	22,5904	22,3561	21,9902	21,6578	22,3168	22,0528
22,7621	22,5572	22,6913	23,3354	23,5653	22,3139	22,5444	23,5366	23,554
22,5518	23,1586	22,9588	21,9353	21,6542	21,6665	21,8895	21,9351	22,0107
NaN	NaN	NaN	20,5882	19,2276	17,9729	NaN	NaN	NaN
27,8218	27,3465	27,4519	28,7901	28,74	27,982	27,7218	27,123	26,8198
21,5594	NaN	NaN	NaN	NaN	20,9831	21,3711	21,5533	21,35
21,5338	22,9223	22,7489	20,684	20,4967	NaN	NaN	NaN	20,7525
30,7828	28,4459	28,3581	31,6401	31,7374	26,9553	26,8553	29,3715	29,459
26,5318	27,1571	27,1696	27,1964	27,2715	26,9937	26,9243	27,9189	27,8144
23,1604	21,3868	21,2214	22,7331	22,8003	21,6811	21,6349	21,9754	22,1408
24,2567	23,765	24,0142	25,2738	25,3814	23,0094	23,2998	22,8724	22,7466

22,4317	NaN	NaN	NaN	NaN	21,6315	21,7364	22,683	22,9134
21,7699	21,4106	NaN	22,2401	22,0452	22,4744	22,5909	NaN	NaN
NaN	22,4118	22,3135	NaN	20,9584	NaN	21,8441	22,1563	21,7853
22,823	24,0266	23,9014	23,4275	23,3897	24,5938	24,5219	23,8321	23,6601
30,1031	29,7698	29,9874	30,5641	30,4683	30,3161	30,5794	30,2539	30,2746
22,3417	NaN	NaN	NaN	NaN	21,532	22,3204	22,5128	22,3375
20,6104	NaN	NaN	NaN	NaN	NaN	NaN	NaN	NaN
22,6687	21,589	21,5227	22,2468	22,185	21,7102	21,8843	22,7181	23,1053
21,0697	NaN	NaN	21,3371	22,1031	21,6613	21,9064	NaN	NaN
21,0253	NaN	NaN	22,651	22,1841	NaN	NaN	21,3399	21,5278
20,6108	21,0982	20,9449	NaN	21,8806	20,8217	21,1331	21,1398	20,7643
23,0706	21,2388	NaN	22,2205	22,482	23,3872	23,4018	22,2626	22,9391
21,1625	NaN	NaN	NaN	NaN	21,5219	NaN	21,6059	NaN
NaN	21,3164	22,0987	21,0219	NaN	NaN	20,6118	NaN	NaN
23,2876	32,9058	32,7556	24,8065	24,9408	25,2452	25,6813	23,7143	23,719
27,7303	26,5343	26,7058	25,4739	25,6415	26,8796	26,9528	27,2889	27,1677
24,4955	22,1175	NaN	23,4777	23,5997	23,5104	23,0214	23,3248	23,3935
23,8441	25,1701	25,1663	24,4741	24,4725	23,628	23,6949	25,0619	24,702
21,7275	22,134	22,0296	22,0414	22,807	NaN	21,9434	21,849	21,5747
22,8566	21,3485	21,0904	22,2025	22,2413	22,5291	22,4601	23,3095	23,288
NaN	NaN	NaN	NaN	NaN	NaN	NaN	NaN	NaN
20,6422	NaN	21,4014	NaN	NaN	NaN	NaN	NaN	NaN
24,9372	25,3816	25,379	23,8312	23,726	25,3626	25,4229	24,8505	24,5364
20,6304	20,9411	21,1757	21,6435	21,3413	20,4603	20,5934	21,1363	21,0456
24,1651	23,6437	23,5496	NaN	NaN	24,2196	24,219	23,2222	23,2755
22,1938	22,4759	22,9235	21,0806	NaN	23,4947	23,2653	22,8346	23,0195
25,6188	24,1715	24,2442	25,3138	25,2443	25,1795	25,2448	23,8312	23,6437
23,2402	25,0164	25,0741	23,9404	23,702	22,9665	23,0468	22,1627	21,9027
24,4503	22,6696	22,9887	24,0554	23,9851	23,5268	23,2964	24,5305	24,6149
23,0451	21,3427	21,7059	23,5605	23,5486	23,0062	23,392	22,6491	22,3104
24,4955	22,7599	22,6861	21,1971	20,6448	24,1512	24,1538	24,3807	24,2891
21,5115	NaN	NaN	22,3365	22,5726	21,5259	21,4275	NaN	NaN
22,8939	23,2565	23,1772	23,1002	22,6846	23,3986	23,5277	23,8569	23,9664
23,0197	21,5027	21,1727	22,5023	22,1013	21,928	22,1592	22,2313	22,4922
23,6646	22,7437	22,9132	22,7993	22,729	23,6834	23,3705	23,1083	22,9293
22,6868	20,8937	21,173	21,0417	20,838	22,2363	22,2874	22,2718	21,8819
21,8189	21,9436	21,6972	22,0532	22,4688	21,4322	21,7096	21,919	21,9187
21,2457	NaN	NaN	22,5659	22,5183	NaN	NaN	22,0322	NaN
21,1476	20,5829	NaN	NaN	NaN	21,1638	NaN	21,0042	20,9936
23,6415	22,4888	22,4631	21,9787	21,5939	22,6554	22,7802	23,5292	23,5953
20,926	NaN	NaN	NaN	NaN	20,8917	21,3492	21,0022	NaN
20,1239	19,686	NaN	NaN	NaN	19,0887	18,2324	20,0983	20,9752
NaN	22,6543	23,5592	23,4663	23,4308	NaN	NaN	21,743	21,4594
22,0382	20,5417	20,948	21,5388	21,946	21,9297	21,6658	21,0136	21,0427
20,9096	NaN	NaN	NaN	NaN	20,7609	20,8525	20,8365	21,6498
22,9299	NaN	NaN	NaN	NaN	22,652	21,9996	22,696	23,1062
22,5449	NaN	NaN	21,5676	22,3843	21,4071	20,7162	20,9835	20,392
22,217	22,6919	22,6946	23,3709	23,1539	21,103	21,6014	23,654	23,4289
22,2388	21,6464	21,2917	22,8999	22,1777	22,0437	21,6133	22,5754	22,4494
23,1359	21,5296	21,3769	21,355	21,2004	22,3374	22,3518	23,0702	22,8929
24,9784	21,9714	21,7962	24,5194	24,6494	23,7105	23,7032	23,1843	23,117
NaN	NaN	NaN	21,9229	22,0906	NaN	20,6086	21,2161	NaN

20,5736	NaN	NaN	21,1856	NaN	20,3703	NaN	NaN	19,8453
25,6638	24,7704	25,0007	25,9908	26,1147	26,6367	26,549	25,7633	25,4448
NaN	NaN	NaN	22,2174	22,128	NaN	NaN	NaN	NaN
NaN	NaN	NaN	NaN	NaN	NaN	NaN	NaN	NaN
NaN	21,7766	22,2356	NaN	NaN	20,7292	NaN	NaN	NaN
22,4241	22,7288	22,6954	21,8969	22,1516	22,9443	22,9137	22,757	22,8942
24,5246	22,9131	23,1101	22,6291	22,5619	23,8537	23,9935	24,5399	24,7134
22,4529	NaN	NaN	20,9291	NaN	22,1266	22,4535	22,6651	22,6155
21,9018	20,8011	21,1148	NaN	NaN	22,1866	21,6277	21,6244	21,3974
25,0118	26,4877	26,3746	23,9903	24,0191	24,5908	24,7396	25,661	25,5358
24,3432	26,8755	26,8978	24,9934	25,044	25,3055	25,5645	24,7402	24,7775
24,4501	23,6306	23,9258	25,6528	25,7324	25,1053	25,3449	23,7432	23,6567
24,2441	24,9716	24,8564	23,8095	23,7122	24,8574	24,9064	24,3412	24,5271
NaN	NaN	NaN	NaN	NaN	NaN	NaN	NaN	NaN
22,0294	NaN	NaN	22,9998	22,9193	21,7334	21,4219	22,8517	22,3684
28,3446	29,4809	29,3483	28,8839	29,215	25,8541	25,6501	28,5352	28,6653
24,4903	22,9739	23,0424	23,4827	23,3185	23,7915	23,6382	24,3912	24,2709
21,5232	22,2392	22,5394	20,9866	20,6085	22,1139	22,0602	21,5475	22,0928
20,27	18,7542	19,674	25,5583	25,4799	22,7597	22,64	21,6845	22,0508
NaN	23,4246	24,4415	NaN	NaN	NaN	NaN	NaN	NaN
24,3045	23,0752	23,2116	22,4926	22,539	24,972	24,8815	24,0655	24,0297
NaN	NaN	NaN	21,9099	22,0934	NaN	NaN	NaN	NaN
21,3166	22,4888	22,3864	22,55	22,9213	22,5764	22,1541	23,9035	23,9986
21,3382	20,9914	21,0228	NaN	20,9124	20,9001	21,981	20,9631	21,0132
21,3804	NaN	NaN	20,4272	NaN	19,9759	NaN	20,8848	NaN
24,5824	23,0319	23,1379	25,3998	25,3727	25,3072	25,1923	23,4947	23,2736
23,5445	21,4157	21,3072	21,959	22,2838	22,9848	23,2691	23,2631	23,4424
20,3053	NaN	NaN	NaN	NaN	19,9355	20,916	NaN	NaN
20,6097	NaN	NaN	NaN	NaN	NaN	NaN	NaN	NaN
24,0926	22,411	22,7761	23,453	24,1937	23,402	23,697	24,3636	24,1856
21,4236	NaN	NaN	NaN	NaN	21,1029	NaN	NaN	NaN
26,5842	25,6245	25,8213	27,9985	28,1165	27,1028	27,3302	25,4475	25,4289
20,7987	NaN	NaN	21,2709	NaN	21,2669	20,9011	21,4528	20,9499
21,1102	NaN	NaN	NaN	NaN	NaN	20,8779	21,6232	22,2998
21,4034	21,0399	NaN	NaN	NaN	21,3891	21,7878	21,313	20,7985
22,1228	23,243	22,1225	23,1833	23,328	21,5766	21,4623	22,2165	21,9426
22,0758	20,3358	20,5088	NaN	NaN	22,0386	21,8272	22,4163	21,8538
21,0585	NaN	NaN	20,1229	NaN	20,4283	21,3936	NaN	20,621
23,3749	24,6812	24,8352	24,5824	24,3866	22,969	23,0081	24,9068	24,7584
21,6297	NaN	NaN	21,6259	21,7041	22,3071	22,007	NaN	NaN
NaN	NaN	NaN	NaN	NaN	NaN	NaN	NaN	NaN
23,3644	22,7013	23,4609	23,1957	22,9749	23,3568	23,0187	23,196	23,2977
23,4183	25,2472	25,2537	23,0626	22,9782	23,8785	23,8596	23,5723	23,6641
NaN	NaN	NaN	NaN	NaN	NaN	NaN	NaN	NaN
NaN	NaN	NaN	NaN	NaN	NaN	NaN	NaN	NaN
NaN	NaN	NaN	NaN	NaN	NaN	NaN	NaN	NaN
24,0692	22,4088	22,4793	22,7353	22,7693	23,771	23,7613	23,787	24,0193
22,0216	NaN	NaN	NaN	NaN	21,9181	NaN	21,8435	21,5961
20,5763	NaN	NaN	20,3564	20,6337	NaN	20,3776	20,3814	NaN
23,5716	22,2919	22,1495	22,8509	22,4578	23,5557	23,619	22,4859	22,6085
NaN	NaN	NaN	21,2152	21,1734	NaN	NaN	NaN	NaN
21,533	NaN	NaN	21,1408	21,1888	NaN	21,1292	21,628	NaN

18,8358	25,3956	24,8973	20,1188	19,2506	20,2139	NaN	20,259	NaN
23,3753	25,6012	24,9038	24,324	24,408	24,6706	24,7445	24,2478	24,3539
21,3864	NaN	NaN	21,8683	22,1119	21,7015	21,7414	20,0855	20,7548
NaN	NaN	NaN	NaN	NaN	NaN	NaN	NaN	NaN
21,5427	23,1822	23,1023	NaN	22,8011	22,5075	22,3245	NaN	NaN
20,3244	20,2193	19,8811	19,8015	NaN	NaN	NaN	NaN	19,7718
23,5522	24,3515	24,6927	24,5388	24,6914	24,3969	24,0628	25,1135	25,2115
NaN	NaN	NaN	NaN	NaN	NaN	NaN	22,363	NaN
21,3304	NaN	NaN	20,7432	21,7576	20,3938	NaN	20,6595	NaN
22,3376	NaN	NaN	NaN	NaN	NaN	NaN	NaN	22,1572
22,8367	23,2037	23,2633	21,4346	21,7846	23,5087	22,9154	22,2751	22,3342
22,5892	NaN	NaN	NaN	NaN	22,4105	22,5864	22,6627	22,3916
22,0728	NaN	NaN	NaN	NaN	NaN	21,6186	22,0121	21,6035
NaN	NaN	NaN	NaN	NaN	NaN	NaN	NaN	NaN
20,7685	NaN	NaN	NaN	NaN	20,2334	21,1635	20,2912	NaN
21,1478	NaN	NaN	21,1723	20,9248	20,6545	20,7222	NaN	NaN
24,4595	23,318	23,5559	23,9456	24,312	24,2248	24,1914	24,4674	24,2775
NaN	NaN	NaN	NaN	NaN	NaN	NaN	NaN	NaN
25,6522	26,1122	26,0511	25,4275	25,2994	25,3378	25,3936	25,8939	25,9812
25,7717	26,822	26,8906	25,9459	25,6939	26,5234	26,6799	26,4321	26,2945
20,6553	20,2335	NaN	NaN	NaN	21,4845	21,2859	22,433	21,6052
21,1266	NaN	NaN	NaN	NaN	NaN	NaN	NaN	NaN
23,9369	26,4614	26,2655	24,1451	24,6155	24,7769	24,6643	25,5415	25,527
23,1095	21,6684	22,1269	NaN	NaN	22,3036	22,8407	22,0029	21,9442
19,7356	19,7795	20,1635	20,4258	20,9219	20,6996	NaN	NaN	NaN
NaN	NaN	NaN	NaN	NaN	NaN	NaN	NaN	NaN
21,0748	22,8323	22,7904	21,9467	21,7876	21,5351	21,5105	23,1611	22,9624
20,686	20,6848	NaN	22,1032	22,1189	20,8315	20,8976	NaN	NaN
20,8561	NaN	NaN	NaN	NaN	NaN	NaN	20,8198	20,7248
21,259	NaN	NaN	NaN	NaN	21,1502	21,2515	21,3738	21,2269
21,007	NaN	NaN	20,897	NaN	21,8155	22,0809	NaN	20,7597
19,6789	NaN	19,7011	NaN	NaN	20,5911	20,3471	20,3754	20,0765
20,216	21,0509	20,8525	20,8496	21,2381	NaN	NaN	NaN	20,6569
19,9197	NaN	NaN	NaN	NaN	19,7822	19,5226	NaN	NaN
20,6244	NaN	NaN	NaN	NaN	20,1515	20,2123	NaN	NaN
20,5258	NaN	20,3766	NaN	NaN	20,5961	20,0317	20,4161	20,4714
21,99	21,0698	19,892	NaN	NaN	21,4532	21,5961	22,1923	21,8616
20,3871	20,8363	20,6084	20,6787	20,7784	20,5793	20,8154	20,8723	21,2505
NaN	NaN	NaN	NaN	NaN	NaN	NaN	NaN	NaN
22,195	20,651	NaN	21,3723	21,3105	22,6563	22,6965	22,2862	21,8647
20,0906	NaN	NaN	NaN	NaN	NaN	NaN	NaN	20,7052
21,5055	NaN	NaN	NaN	NaN	21,7628	21,3925	21,5428	20,8796
21,1888	NaN	NaN	NaN	NaN	NaN	NaN	21,6297	20,9378
22,3192	NaN	NaN	21,1481	21,0832	22,6912	22,5139	23,0798	21,1087
NaN	20,8207	20,7509	NaN	NaN	NaN	NaN	NaN	NaN
NaN	27,8051	27,8627	NaN	NaN	NaN	NaN	NaN	NaN
21,3652	NaN	20,2342	20,827	NaN	20,7494	20,7087	20,9065	NaN
22,9563	NaN	NaN	NaN	NaN	22,027	21,9511	23,2768	22,7388
22,1448	22,5247	22,5295	22,537	22,3331	22,4286	22,5265	23,1051	22,9735
20,0446	NaN	NaN	NaN	NaN	NaN	NaN	NaN	NaN
21,6789	20,8956	21,1376	21,5353	22,115	21,4493	21,2871	NaN	20,9051
20,0603	NaN	NaN	NaN	NaN	19,7567	NaN	NaN	NaN

20,2805	NaN	NaN	NaN	NaN	NaN	NaN	NaN	NaN
21,2739	NaN	NaN	22,2207	22,5023	21,9341	22,1988	21,0153	21,4216
20,7963	NaN	20,3146	NaN	NaN	20,2915	20,6852	20,9034	21,1169
26,1261	24,9277	24,926	26,7196	26,5596	26,4868	26,6644	24,6007	24,5121
NaN	NaN	NaN	NaN	NaN	NaN	NaN	NaN	NaN
20,6191	NaN	20,8088	21,395	21,6314	NaN	21,4927	21,7951	21,6568
27,111	26,0086	26,1993	25,2994	25,4526	27,1828	27,4972	27,1651	27,2458
22,5588	22,0625	21,7908	21,9511	22,4348	22,0673	22,2695	21,981	22,2251
22,6273	NaN	NaN	21,852	NaN	22,6779	22,3548	21,7507	22,1659
19,1082	NaN	NaN	NaN	NaN	20,8603	21,4165	19,3956	NaN
21,6186	21,6037	21,2279	20,7673	20,7791	21,0373	20,7168	20,9062	NaN
26,7369	26,3747	26,3781	27,9393	27,9159	26,742	26,7222	26,0871	26,1331
NaN	22,1852	21,667	NaN	21,7757	23,5737	23,0826	NaN	NaN
19,5502	NaN	NaN	22,5637	22,7324	NaN	NaN	NaN	NaN
21,7193	NaN	NaN	21,3385	21,364	21,0556	21,0651	20,539	20,6894
20,7792	NaN	NaN	21,0432	20,7104	NaN	20,3561	NaN	NaN
20,8516	21,1572	21,2068	NaN	NaN	NaN	NaN	NaN	NaN
23,1151	NaN	NaN	NaN	NaN	21,8209	22,4548	22,6362	23,1447
22,368	NaN	NaN	21,9751	22,2578	21,4486	21,6283	22,5242	22,6145
21,5799	NaN	NaN	NaN	NaN	20,7452	20,5367	20,8783	20,6159
25,2269	23,5585	23,478	25,0282	25,0905	25,2933	25,4125	24,6065	24,7098
22,0281	20,3416	NaN	21,4553	21,4874	22,7642	21,9184	22,693	22,0551
26,2002	25,2189	25,4144	24,737	24,9791	25,5326	25,6804	26,0916	26,2935
25,5192	NaN	20,5538	22,4955	22,3173	24,9105	25,0299	24,6325	24,8148
21,0201	NaN	NaN	NaN	NaN	NaN	20,5418	NaN	20,7498
23,6827	22,222	22,5908	25,1722	25,0065	23,0828	22,7417	23,3254	23,2248
NaN	NaN	NaN	22,4742	22,2169	NaN	NaN	NaN	NaN
23,1199	23,2377	22,63	24,0976	24,0261	23,0392	23,1381	22,4837	22,0454
24,3215	27,5886	27,3484	25,9847	26,1098	24,0578	24,2341	25,4157	25,343
22,4582	22,3035	22,5014	22,2463	22,7637	22,1	21,7279	22,4482	22,4987
NaN	NaN	NaN	NaN	NaN	NaN	NaN	NaN	NaN
25,8181	24,7543	24,5977	24,2689	24,082	25,7221	25,7412	25,8081	25,5782
23,7303	21,8205	21,7264	24,2743	24,1236	23,9558	24,0709	23,4425	23,606
23,4233	23,4328	23,3372	23,2623	23,265	24,0869	24,4942	23,6046	23,6402
NaN	21,3912	21,4016	NaN	NaN	21,66	20,5058	NaN	20,9687
21,3143	NaN	NaN	21,1904	NaN	21,5634	21,0856	NaN	NaN
23,1496	NaN	NaN	22,3143	21,7896	22,3243	22,9393	NaN	NaN
19,8761	NaN	20,1162	NaN	20,4843	NaN	20,5165	21,0364	20,708
21,6215	NaN	NaN	NaN	NaN	21,2084	21,5936	20,4071	21,2638
20,3454	NaN	20,1696	NaN	NaN	20,0776	NaN	NaN	NaN
24,4889	23,9241	24,0857	23,5201	23,6475	24,8872	24,978	24,7133	24,8752
NaN	NaN	NaN	20,7898	NaN	NaN	NaN	NaN	NaN
20,1593	NaN	NaN	NaN	NaN	20,2463	19,8782	19,7514	NaN
20,9623	NaN	NaN	20,7551	21,0449	NaN	NaN	NaN	NaN
21,5301	NaN	NaN	NaN	NaN	21,2206	20,938	21,661	21,524
NaN	21,9071	21,8949	20,1345	NaN	20,1461	20,3451	20,703	20,0319
21,0206	NaN	NaN	NaN	NaN	NaN	NaN	NaN	20,7078
21,5188	NaN	NaN	NaN	NaN	NaN	20,4836	20,6412	20,9574
NaN	NaN	NaN	NaN	NaN	20,7899	NaN	NaN	NaN
20,3195	NaN	NaN	NaN	NaN	20,3886	NaN	NaN	NaN
23,2929	NaN	22,3942	23,5229	23,1671	23,5601	23,791	22,644	23,096
21,3288	NaN	NaN	20,8746	20,9184	21,7513	22,013	21,0302	21,1009

19,9469	NaN	NaN	NaN	NaN	NaN	NaN	NaN	NaN
23,8024	25,3793	25,3377	24,6794	24,7098	25,1232	24,7167	24,7511	25,1067
25,1999	25,9888	26,2775	27,1447	26,6979	26,8267	27,2566	26,1772	26,2255
24,9452	24,6477	24,8472	25,3346	25,0902	25,1284	25,0881	25,432	26,0961
24,7466	25,9955	25,8748	23,6897	NaN	26,818	26,7574	26,4032	26,0776
25,6918	24,5655	24,5774	26,3297	26,2396	26,1217	26,1578	24,493	24,3943
NaN	NaN	NaN	NaN	NaN	NaN	NaN	NaN	NaN
NaN	NaN	NaN	NaN	NaN	NaN	NaN	NaN	NaN
20,855	NaN	NaN	NaN	NaN	20,9047	20,7388	20,673	20,917
21,8556	NaN	NaN	NaN	NaN	21,2684	21,6348	21,4223	21,8504
NaN	20,7474	20,5725	NaN	NaN	21,0647	20,3512	NaN	19,9413
NaN	NaN	NaN	NaN	NaN	NaN	NaN	NaN	NaN
NaN	NaN	NaN	NaN	NaN	19,2402	20,2567	NaN	NaN
NaN	NaN	NaN	NaN	20,1758	NaN	NaN	NaN	NaN
NaN	NaN	NaN	NaN	NaN	NaN	20,46	NaN	20,6383
NaN	NaN	NaN	NaN	NaN	19,9884	NaN	NaN	NaN
21,208	NaN	NaN	NaN	NaN	21,4406	21,2115	21,3745	20,9537
NaN	NaN	NaN	NaN	NaN	NaN	NaN	NaN	NaN
NaN	NaN	NaN	NaN	NaN	NaN	NaN	NaN	NaN
NaN	NaN	NaN	NaN	NaN	NaN	NaN	21,0798	NaN
NaN	NaN	NaN	NaN	NaN	NaN	NaN	NaN	NaN
NaN	NaN	NaN	NaN	NaN	NaN	NaN	NaN	NaN
22,4556	NaN	NaN	NaN	NaN	21,7362	21,8351	NaN	21,7487
NaN	NaN	NaN	NaN	NaN	NaN	NaN	NaN	NaN
20,6911	21,6487	21,8462	20,9942	21,1155	20,9218	21,1368	21,0325	21,2922
20,5208	NaN	NaN	20,5719	20,2362	20,5981	20,8149	20,5934	NaN
22,8973	22,8764	22,7854	NaN	NaN	23,5336	23,2624	NaN	NaN
20,6957	NaN	NaN	NaN	NaN	20,6069	21,184	NaN	NaN
24,3414	23,5331	23,6675	NaN	21,802	24,9657	24,6664	24,6568	24,7704
20,5793	NaN	NaN	20,8251	20,8046	20,2171	20,4249	20,3394	20,1311
19,696	22,8826	23,4446	19,6794	19,7005	NaN	NaN	NaN	19,8341
23,0043	21,9114	21,9609	20,9157	21,273	22,6146	21,8918	23,3484	23,016
20,6858	NaN	NaN	NaN	NaN	NaN	NaN	NaN	21,3341
21,5232	NaN	NaN	21,7773	21,6016	20,6521	20,3847	NaN	21,3506
NaN	NaN	NaN	NaN	NaN	NaN	NaN	NaN	20,1408
21,6084	NaN	NaN	21,191	21,08	NaN	NaN	NaN	NaN
21,0035	NaN	NaN	21,3097	21,4178	NaN	NaN	NaN	NaN
NaN	NaN	NaN	NaN	NaN	NaN	NaN	NaN	NaN
NaN	NaN	NaN	NaN	NaN	NaN	NaN	NaN	20,4791
NaN	NaN	NaN	NaN	NaN	NaN	NaN	NaN	NaN
20,8642	NaN	NaN	NaN	NaN	NaN	NaN	NaN	NaN
NaN	NaN	NaN	NaN	NaN	NaN	NaN	NaN	NaN
19,3801	NaN	NaN	20,9615	22,3375	NaN	NaN	19,0287	18,8178
21,0944	21,2579	21,8865	NaN	20,5623	21,8262	22,0337	22,4851	22,1891
19,2654	NaN	NaN	NaN	NaN	22,0717	22,1488	20,3567	20,7152
21,7205	21,2134	NaN	21,1343	21,7312	NaN	NaN	NaN	NaN
21,9108	NaN	21,6061	22,0276	21,8505	NaN	21,5922	21,2631	21,6121
NaN	NaN	NaN	NaN	NaN	NaN	NaN	NaN	NaN
20,2838	NaN	NaN	NaN	21,1314	20,7301	20,6865	NaN	20,9616
NaN	NaN	NaN	NaN	NaN	NaN	NaN	NaN	NaN
NaN	NaN	NaN	22,8731	22,8933	NaN	NaN	NaN	NaN
20,1625	20,9741	20,4406	22,2602	22,071	20,2759	20,2107	19,8678	20,2246

21,1512	NaN	NaN	22,443	22,8713	21,4178	21,4017	NaN	NaN
NaN	NaN	NaN	NaN	NaN	NaN	NaN	NaN	NaN
21,2503	NaN	20,9805	NaN	NaN	21,3978	21,2511	NaN	NaN
NaN	NaN	NaN	NaN	NaN	20,6859	NaN	NaN	NaN
21,9028	21,446	20,7384	21,8593	21,2518	21,2441	NaN	NaN	21,1974
21,0755	NaN	20,1969	NaN	NaN	20,6746	20,8737	NaN	NaN
NaN	NaN	NaN	NaN	NaN	NaN	NaN	NaN	NaN
21,187	20,7281	20,9543	NaN	NaN	21,2703	20,8817	21,1925	20,9441
NaN	NaN	NaN	NaN	NaN	20,2533	NaN	NaN	NaN
20,6146	NaN	NaN	22,5355	21,6756	NaN	NaN	NaN	NaN
NaN	NaN	NaN	NaN	NaN	NaN	NaN	NaN	NaN
NaN	NaN	NaN	NaN	NaN	NaN	NaN	NaN	NaN
23,7948	NaN	24,4113	NaN	NaN	25,8639	25,6553	23,6289	23,2679
NaN	NaN	NaN	NaN	NaN	NaN	NaN	NaN	NaN
21,9349	NaN	NaN	NaN	NaN	NaN	NaN	NaN	NaN
21,6683	20,8445	NaN	NaN	20,9603	NaN	NaN	NaN	NaN
22,3626	NaN	NaN	NaN	NaN	NaN	NaN	22,1485	21,9374
25,0378	25,6075	25,8975	26,1834	26,0703	25,8821	25,7741	25,0586	24,8699
21,9352	NaN	NaN	21,5942	21,246	NaN	NaN	NaN	NaN
23,5937	23,6113	NaN	24,372	24,2241	25,1829	25,2829	23,8044	24,0454
NaN	NaN	NaN	NaN	NaN	NaN	NaN	NaN	NaN
NaN	NaN	22,1457	NaN	NaN	NaN	NaN	NaN	NaN
NaN	24,8553	24,7609	NaN	NaN	21,6627	21,3959	21,102	21,0188
NaN	20,9901	NaN	21,0057	21,0532	19,7439	NaN	NaN	NaN
23,8425	25,0925	25,1139	25,1509	25,034	25,4281	25,2355	25,3277	25,1331
20,9257	20,2098	NaN	NaN	NaN	20,385	20,5055	20,408	NaN
20,8858	NaN	NaN	21,3943	21,4773	20,458	20,0383	19,9714	20,3287
NaN	NaN	NaN	NaN	NaN	NaN	NaN	NaN	NaN
NaN	NaN	NaN	NaN	NaN	NaN	NaN	NaN	NaN
NaN	NaN	NaN	NaN	NaN	NaN	NaN	NaN	NaN
21,5624	NaN	NaN	NaN	NaN	NaN	NaN	NaN	NaN
21,5062	NaN	20,1733	NaN	NaN	20,753	20,4525	20,5888	20,3121
NaN	NaN	NaN	NaN	NaN	NaN	NaN	NaN	NaN
19,7836	NaN	NaN	19,9124	20,4433	20,8976	NaN	NaN	19,7938
NaN	NaN	NaN	NaN	NaN	NaN	NaN	NaN	NaN
20,2769	23,3953	22,8471	21,3035	21,0083	21,1996	21,3417	21,431	21,3286
19,963	NaN	NaN	NaN	NaN	NaN	NaN	NaN	NaN
19,141	20,3004	19,9744	NaN	NaN	NaN	19,2986	19,5921	19,3277
19,8838	NaN	NaN	NaN	NaN	NaN	NaN	NaN	NaN
NaN	NaN	NaN	NaN	NaN	NaN	NaN	21,123	NaN
NaN	NaN	NaN	NaN	NaN	NaN	NaN	NaN	NaN
20,1353	NaN	21,0769	20,9329	21,2303	NaN	NaN	20,8162	20,0612
20,4226	NaN	NaN	NaN	NaN	NaN	20,0847	20,4649	20,005
23,1244	NaN	NaN	20,9671	21,3376	22,5327	22,4978	23,2761	23,0278
21,4112	20,6189	20,6685	NaN	NaN	NaN	19,6241	20,911	NaN
22,0304	21,3576	21,2471	22,7168	NaN	21,0009	21,5596	NaN	21,2094
21,908	NaN	21,7397	21,2913	21,3165	20,7712	20,6199	22,3998	22,4318
NaN	NaN	NaN	NaN	NaN	NaN	NaN	NaN	NaN
21,204	NaN	NaN	NaN	NaN	20,2583	20,3855	20,5443	20,5097
NaN	NaN	NaN	NaN	NaN	NaN	NaN	NaN	NaN
23,9939	21,9262	21,7193	24,0754	23,9638	21,6395	21,8046	20,729	NaN
24,9765	22,5758	22,9186	26,5273	26,6339	24,3047	24,2321	24,1738	24,3586

NaN	NaN	NaN	NaN	NaN	20,6505	20,546	20,7676	NaN
NaN	21,4224	20,3298	NaN	NaN	NaN	NaN	NaN	NaN
NaN	NaN	NaN	NaN	NaN	NaN	NaN	NaN	NaN
19,8002	NaN	19,4792	NaN	NaN	NaN	NaN	19,6617	NaN
NaN	NaN	NaN	21,0274	20,7292	NaN	NaN	NaN	NaN
NaN	NaN	NaN	NaN	NaN	NaN	NaN	NaN	NaN
NaN	NaN	NaN	NaN	NaN	NaN	NaN	NaN	NaN
NaN	NaN	NaN	NaN	NaN	NaN	NaN	NaN	NaN
NaN	NaN	NaN	NaN	NaN	NaN	NaN	NaN	NaN
24,2717	23,4397	23,829	23,9848	24,2398	24,2934	24,4198	23,2694	22,7129
20,9797	NaN	NaN	NaN	NaN	NaN	NaN	21,016	21,012
22,7418	22,9042	23,0267	23,4366	23,3911	22,8814	22,6221	22,7987	22,675
NaN	NaN	NaN	20,8416	NaN	NaN	NaN	NaN	NaN
24,6425	25,4032	25,4691	25,7109	25,3392	25,5882	25,7184	25,5994	25,4235
NaN	NaN	NaN	19,9451	19,9621	NaN	NaN	NaN	NaN
20,7413	20,5211	NaN	20,7193	NaN	20,5257	NaN	NaN	NaN
23,4328	21,845	21,7019	21,5602	21,3637	22,6451	22,3023	22,7805	22,6056
NaN	NaN	NaN	NaN	NaN	NaN	NaN	NaN	NaN
NaN	21,3423	21,3446	NaN	NaN	19,9649	20,1706	NaN	NaN
24,2007	23,1255	22,9554	20,8579	20,9989	24,2777	24,2234	24,274	24,5599
NaN	NaN	NaN	NaN	NaN	NaN	NaN	NaN	NaN
24,6797	21,9323	22,1579	24,1984	24,0986	24,0996	24,7438	23,3568	22,9976
NaN	NaN	NaN	21,5128	NaN	NaN	NaN	NaN	NaN
NaN	NaN	NaN	NaN	NaN	NaN	NaN	NaN	NaN
NaN	NaN	NaN	NaN	NaN	NaN	NaN	NaN	NaN
23,0756	21,8412	21,8601	NaN	NaN	23,6729	23,1683	23,6616	23,7701
23,4064	23,9361	24,2625	NaN	NaN	23,0749	23,0097	NaN	NaN
20,4146	NaN	NaN	NaN	NaN	20,5926	20,6903	NaN	NaN
NaN	NaN	19,2444	NaN	NaN	NaN	NaN	NaN	NaN
19,9627	21,1904	21,5107	NaN	NaN	NaN	NaN	NaN	NaN
20,1401	NaN	NaN	NaN	NaN	20,2508	20,1507	20,1597	19,8994
23,0857	21,6015	21,448	23,5873	23,5066	22,9004	22,2874	21,9117	22,0265
NaN	NaN	NaN	NaN	NaN	NaN	NaN	NaN	NaN
NaN	21,9273	22,2244	21,0279	NaN	NaN	NaN	21,2019	NaN
23,5626	NaN	NaN	22,8868	22,6836	23,5905	21,7573	NaN	NaN
NaN	21,4551	21,8561	21,1068	21,3542	NaN	NaN	NaN	NaN
NaN	NaN	NaN	NaN	NaN	NaN	NaN	NaN	NaN
21,383	NaN	NaN	20,6218	NaN	NaN	NaN	20,744	20,6953
27,6149	27,9032	27,9388	26,2879	26,1501	28,558	28,4541	28,1342	28,2049
21,9223	21,3571	NaN	NaN	21,8244	21,9794	21,6861	22,0335	21,6552
20,5488	NaN	NaN	NaN	NaN	NaN	19,9363	20,7982	21,023
23,2353	22,4647	22,5345	22,1267	22,071	NaN	21,7624	NaN	NaN
20,9331	NaN	NaN	NaN	20,6581	20,2243	20,3777	21,0885	20,794
21,4405	20,4443	NaN	21,3025	21,4045	20,4549	20,9861	20,8838	20,7788
NaN	NaN	NaN	NaN	NaN	NaN	NaN	NaN	NaN
23,2374	23,5657	23,4858	NaN	NaN	24,0162	23,7207	23,5552	23,7801
21,2043	NaN	NaN	21,4456	NaN	NaN	NaN	NaN	NaN
20,7661	21,8815	22,3619	NaN	NaN	NaN	NaN	20,3066	20,6226
21,4478	22,5714	22,6544	23,744	24,043	24,3257	24,493	22,727	23,8107
21,7876	22,8153	22,4764	23,7702	23,4403	NaN	NaN	NaN	NaN
27,5095	NaN	NaN	NaN	NaN	NaN	NaN	NaN	NaN
NaN	NaN	NaN	NaN	NaN	NaN	NaN	NaN	NaN

20,5659	NaN	NaN	21,9398	22,2534	21,1912	22,5221	20,8385	20,2532
22,6163	21,7492	21,8536	NaN	NaN	22,5031	22,4375	21,7853	21,8815
NaN	NaN	NaN	NaN	NaN	NaN	NaN	NaN	NaN
NaN	NaN	NaN	NaN	NaN	NaN	NaN	NaN	NaN
NaN	NaN	NaN	NaN	NaN	NaN	NaN	NaN	NaN
NaN	NaN	21,0147	NaN	NaN	NaN	NaN	NaN	NaN
NaN	20,1335	NaN	NaN	NaN	NaN	NaN	NaN	NaN
20,9828	NaN	20,3592	21,8587	21,2939	21,2327	20,9722	NaN	NaN
NaN	NaN	NaN	NaN	NaN	NaN	NaN	NaN	NaN
NaN	21,0137	20,9439	20,8594	20,5671	20,3102	20,4434	NaN	NaN
NaN	NaN	NaN	NaN	NaN	NaN	NaN	NaN	NaN
20,4011	NaN	NaN	20,1796	NaN	NaN	NaN	20,8451	NaN
NaN	NaN	NaN	NaN	NaN	NaN	NaN	NaN	NaN
NaN	NaN	NaN	NaN	NaN	NaN	NaN	NaN	NaN
NaN	NaN	NaN	NaN	NaN	NaN	NaN	NaN	NaN
NaN	NaN	NaN	NaN	19,5096	NaN	NaN	18,8248	NaN
NaN	NaN	NaN	NaN	NaN	NaN	NaN	NaN	NaN
NaN	NaN	NaN	NaN	NaN	NaN	NaN	NaN	NaN
NaN	NaN	NaN	NaN	NaN	NaN	NaN	NaN	NaN
19,5512	NaN	NaN	NaN	NaN	NaN	NaN	NaN	NaN
20,183	NaN	20,3497	NaN	NaN	20,2793	19,9428	NaN	NaN
19,5746	NaN	NaN	NaN	NaN	NaN	NaN	NaN	NaN
NaN	NaN	NaN	NaN	NaN	NaN	NaN	NaN	NaN
NaN	NaN	NaN	NaN	NaN	NaN	NaN	NaN	NaN
NaN	NaN	NaN	NaN	NaN	NaN	NaN	NaN	NaN
NaN	NaN	NaN	NaN	NaN	NaN	NaN	NaN	NaN
20,7663	NaN	NaN	20,1816	NaN	NaN	NaN	21,4964	NaN
NaN	NaN	NaN	NaN	NaN	NaN	NaN	NaN	NaN
NaN	23,3291	23,0438	25,6006	25,6252	NaN	NaN	23,442	23,4697
NaN	NaN	NaN	21,0049	NaN	NaN	20,1322	NaN	NaN
23,142	23,2572	22,7157	22,9419	23,1947	23,3483	NaN	24,1249	24,1647
NaN	NaN	NaN	NaN	21,4522	23,5634	23,4538	22,5157	21,4088
NaN	NaN	NaN	NaN	20,9923	NaN	NaN	NaN	NaN
NaN	NaN	NaN	NaN	NaN	NaN	NaN	21,785	21,8301
NaN	NaN	NaN	NaN	NaN	NaN	NaN	NaN	NaN
22,1163	22,4029	22,8075	22,5441	22,5637	23,2565	23,3294	21,9385	22,1106
NaN	NaN	NaN	NaN	NaN	NaN	NaN	NaN	NaN
20,8595	NaN	NaN	NaN	NaN	NaN	NaN	20,6941	20,8404
23,7367	24,3901	24,0636	24,7252	24,4233	24,3376	24,3421	25,0497	25,0636
22,2991	22,1549	NaN	22,8766	22,7095	23,312	22,6633	NaN	23,7379
NaN	NaN	NaN	NaN	NaN	NaN	NaN	21,3283	NaN
23,1508	23,9098	23,9127	23,7991	24,072	24,8641	24,7389	24,0259	24,1976
NaN	NaN	NaN	NaN	NaN	NaN	NaN	NaN	NaN
20,0539	NaN	NaN	NaN	NaN	20,3638	NaN	NaN	NaN
19,9516	NaN	NaN	NaN	NaN	NaN	NaN	NaN	NaN
21,5463	NaN	NaN	NaN	NaN	NaN	NaN	NaN	NaN
NaN	22,0464	NaN	22,1152	22,1527	22,807	22,9203	NaN	NaN
NaN	NaN	NaN	NaN	NaN	NaN	NaN	NaN	NaN
21,5805	22,3488	22,053	21,4107	20,8469	21,5381	21,0431	20,7466	20,5731
NaN	NaN	NaN	NaN	NaN	NaN	NaN	NaN	NaN
22,4193	22,3435	22,3188	22,7806	22,7778	24,0069	23,0839	22,7625	22,5727
23,9576	24,3564	24,435	25,6178	25,5101	25,3407	25,651	25,4116	25,2906

23,1584	NaN	NaN	NaN	NaN	NaN	23,4085	NaN	NaN
NaN	NaN	NaN	22,0072	NaN	NaN	NaN	NaN	NaN
22,3078	NaN	NaN	23,7129	24,0043	22,8648	23,1738	22,8962	NaN
21,3227	NaN	NaN	NaN	NaN	NaN	19,6502	20,3873	20,6114
NaN	NaN	NaN	NaN	NaN	NaN	NaN	NaN	NaN
NaN	NaN	NaN	NaN	NaN	NaN	NaN	NaN	NaN
NaN	NaN	NaN	NaN	NaN	NaN	NaN	NaN	NaN
20,8241	NaN	NaN	21,0513	NaN	21,0714	NaN	NaN	NaN
NaN	NaN	NaN	NaN	NaN	NaN	NaN	NaN	NaN
NaN	NaN	NaN	NaN	NaN	NaN	NaN	NaN	NaN
NaN	NaN	NaN	NaN	NaN	NaN	NaN	NaN	NaN
NaN	NaN	NaN	NaN	NaN	NaN	NaN	NaN	NaN
NaN	NaN	NaN	NaN	NaN	NaN	NaN	NaN	NaN
NaN	NaN	NaN	NaN	NaN	NaN	NaN	NaN	NaN
21,2677	NaN	NaN	NaN	NaN	NaN	NaN	NaN	NaN
NaN	NaN	NaN	NaN	NaN	NaN	NaN	NaN	NaN
NaN	NaN	NaN	NaN	NaN	NaN	NaN	NaN	NaN
20,4815	NaN	NaN	NaN	NaN	NaN	NaN	20,5687	20,4921
NaN	NaN	NaN	NaN	NaN	NaN	NaN	NaN	20,2725
21,5813	NaN	NaN	NaN	22,1635	21,6095	21,8683	21,4073	NaN
21,7913	22,0621	22,2529	20,4221	NaN	20,5958	20,6903	21,3189	21,033
NaN	NaN	NaN	NaN	NaN	NaN	NaN	NaN	NaN
19,8983	22,1072	21,9656	21,2783	20,9025	NaN	NaN	20,2091	NaN
NaN	NaN	NaN	NaN	NaN	NaN	NaN	NaN	NaN
NaN	NaN	NaN	NaN	NaN	NaN	NaN	NaN	NaN
NaN	NaN	NaN	NaN	NaN	NaN	NaN	NaN	NaN
NaN	NaN	NaN	NaN	NaN	NaN	NaN	NaN	NaN
NaN	NaN	NaN	NaN	NaN	NaN	NaN	NaN	NaN
20,7431	NaN	NaN	20,6342	20,2182	NaN	NaN	NaN	NaN
NaN	NaN	NaN	NaN	NaN	NaN	NaN	NaN	NaN
NaN	NaN	NaN	NaN	NaN	NaN	NaN	NaN	NaN
21,7316	NaN	NaN	NaN	NaN	NaN	21,1174	22,0072	NaN
NaN	NaN	NaN	NaN	NaN	NaN	NaN	NaN	NaN
NaN	NaN	NaN	NaN	NaN	NaN	NaN	NaN	NaN
NaN	NaN	NaN	NaN	NaN	NaN	NaN	NaN	NaN
21,7016	21,1238	21,2349	22,1708	22,1878	NaN	20,6244	NaN	NaN
22,2943	25,3182	25,3308	22,3531	22,1681	22,324	22,2303	22,6341	22,9337
NaN	NaN	NaN	NaN	NaN	NaN	NaN	NaN	NaN
23,851	NaN	NaN	23,9876	24,0402	24,6312	24,5551	23,4761	23,5115
NaN	NaN	NaN	19,7609	NaN	NaN	NaN	19,798	NaN
NaN	NaN	NaN	NaN	NaN	NaN	NaN	NaN	NaN
24,2003	24,6539	24,6336	24,6057	24,5954	24,978	24,9711	25,2148	25,5153
22,2648	NaN	NaN	NaN	NaN	21,825	22,0548	22,5953	22,6231
21,1537	NaN	NaN	NaN	NaN	21,1834	20,8025	NaN	NaN
NaN	NaN	NaN	20,7798	20,9794	20,8514	NaN	NaN	NaN
19,4202	NaN	NaN	NaN	NaN	20,4157	20,2182	19,1473	NaN
NaN	NaN	NaN	NaN	NaN	NaN	NaN	NaN	NaN
21,8942	NaN	NaN	NaN	NaN	NaN	NaN	NaN	NaN
20,8438	20,4972	NaN	NaN	21,4639	20,7045	NaN	NaN	NaN
21,6093	NaN	NaN	NaN	NaN	NaN	NaN	21,4819	21,5223
NaN	NaN	NaN	NaN	NaN	NaN	NaN	NaN	NaN
NaN	NaN	NaN	NaN	NaN	NaN	NaN	NaN	NaN

NaN	NaN	NaN	NaN	NaN	NaN	NaN	NaN	NaN
23,3391	NaN	NaN	22,4603	NaN	21,428	21,3252	NaN	21,3971
NaN	NaN	NaN	NaN	NaN	NaN	NaN	NaN	NaN
20,6775	NaN	NaN	NaN	NaN	NaN	NaN	NaN	NaN
NaN	NaN	NaN	23,1145	22,9057	NaN	NaN	NaN	NaN
NaN	22,4693	22,3873	23,0204	23,99	NaN	NaN	NaN	NaN
21,9346	22,1435	21,4998	21,9036	21,9699	21,8343	21,8718	22,0304	22,1932
24,72	25,5379	25,5235	25,004	25,2371	24,445	24,492	25,3618	25,363
NaN	NaN	NaN	NaN	NaN	NaN	NaN	NaN	NaN
NaN	NaN	NaN	NaN	NaN	NaN	NaN	NaN	NaN
21,9083	22,3172	22,3647	22,5608	22,2988	22,7205	22,568	NaN	22,6002
22,8683	NaN	NaN	NaN	NaN	24,1075	24,7368	NaN	23,5512
NaN	NaN	NaN	NaN	NaN	NaN	NaN	NaN	NaN
23,0895	NaN	NaN	NaN	NaN	NaN	NaN	NaN	NaN
NaN	NaN	20,6545	NaN	NaN	NaN	NaN	NaN	20,7366
23,2915	24,0865	24,6538	25,1152	25,1525	24,9508	25,1737	23,937	24,2994
NaN	NaN	NaN	NaN	NaN	NaN	NaN	NaN	NaN
19,8389	NaN	NaN	21,3996	20,7112	NaN	NaN	NaN	NaN
NaN	NaN	NaN	NaN	NaN	NaN	NaN	NaN	NaN
24,3017	NaN	NaN	NaN	NaN	NaN	23,8232	NaN	NaN
NaN	NaN	NaN	NaN	NaN	NaN	NaN	NaN	NaN
NaN	NaN	NaN	21,1544	NaN	NaN	NaN	20,5121	20,3811
NaN	NaN	NaN	22,8924	22,8637	NaN	23,1871	NaN	22,6591
NaN	NaN	NaN	NaN	NaN	NaN	NaN	NaN	NaN
NaN	NaN	NaN	NaN	NaN	NaN	NaN	NaN	NaN
NaN	NaN	20,5744	NaN	NaN	NaN	NaN	NaN	NaN
NaN	NaN	NaN	NaN	NaN	NaN	NaN	NaN	NaN
23,0468	23,3401	23,2078	23,5273	23,3123	24,2557	24,2726	23,3207	23,759
NaN	NaN	NaN	NaN	NaN	NaN	NaN	NaN	NaN
NaN	NaN	NaN	NaN	NaN	NaN	NaN	NaN	NaN
NaN	NaN	NaN	NaN	NaN	NaN	NaN	NaN	NaN
NaN	NaN	NaN	NaN	NaN	NaN	NaN	NaN	NaN
NaN	NaN	NaN	20,8045	20,6975	NaN	NaN	20,0942	20,2742
20,5051	NaN	22,4276	NaN	NaN	NaN	NaN	NaN	20,5073
NaN	NaN	NaN	NaN	NaN	NaN	NaN	NaN	NaN
NaN	NaN	NaN	NaN	NaN	NaN	NaN	NaN	NaN
NaN	NaN	NaN	NaN	NaN	NaN	NaN	NaN	NaN
NaN	NaN	NaN	NaN	NaN	NaN	NaN	NaN	NaN
NaN	NaN	NaN	21,2048	NaN	NaN	NaN	NaN	NaN
NaN	NaN	NaN	NaN	NaN	NaN	NaN	NaN	NaN
NaN	NaN	NaN	NaN	NaN	NaN	NaN	NaN	NaN
NaN	NaN	NaN	NaN	NaN	NaN	NaN	NaN	NaN
NaN	NaN	NaN	NaN	NaN	NaN	NaN	NaN	NaN
NaN	NaN	NaN	NaN	NaN	NaN	NaN	NaN	NaN
NaN	21,8628	21,8472	NaN	21,1299	NaN	NaN	20,9531	NaN
NaN	NaN	NaN	NaN	NaN	NaN	NaN	NaN	NaN
NaN	NaN	NaN	NaN	NaN	NaN	NaN	NaN	NaN
NaN	NaN	NaN	NaN	NaN	NaN	20,1121	NaN	NaN
NaN	NaN	NaN	NaN	NaN	NaN	NaN	NaN	NaN
19,9542	NaN	NaN	NaN	NaN	21,1042	20,1761	20,5389	NaN
NaN	NaN	NaN	NaN	NaN	NaN	NaN	NaN	NaN
21,9258	NaN	NaN	21,6104	21,4669	21,7864	21,6511	NaN	NaN

NaN	NaN	NaN	NaN	NaN	NaN	NaN	NaN	NaN
NaN	NaN	NaN	NaN	NaN	NaN	NaN	NaN	NaN
NaN	NaN	NaN	NaN	NaN	NaN	NaN	NaN	NaN
NaN	NaN	NaN	NaN	NaN	NaN	NaN	NaN	NaN
NaN	21,2645	21,8391	NaN	NaN	NaN	NaN	NaN	NaN
NaN	NaN	NaN	NaN	NaN	20,0121	20,1023	20,2463	NaN
NaN	NaN	NaN	NaN	NaN	NaN	NaN	NaN	NaN
NaN	NaN	NaN	NaN	NaN	20,1975	NaN	NaN	NaN
NaN	NaN	NaN	NaN	NaN	NaN	NaN	NaN	NaN
NaN	NaN	NaN	NaN	NaN	NaN	NaN	NaN	NaN
19,5236	NaN	NaN	NaN	NaN	NaN	NaN	NaN	19,5512
NaN	NaN	NaN	NaN	20,4226	NaN	NaN	NaN	NaN
NaN	NaN	NaN	NaN	NaN	NaN	NaN	NaN	NaN
NaN	NaN	NaN	NaN	NaN	NaN	NaN	NaN	NaN
NaN	NaN	NaN	NaN	NaN	NaN	NaN	NaN	NaN
NaN	NaN	NaN	NaN	NaN	NaN	NaN	NaN	NaN
19,1493	NaN	NaN	NaN	NaN	NaN	NaN	NaN	NaN
NaN	22,7615	22,9041	NaN	NaN	NaN	NaN	NaN	NaN
NaN	NaN	NaN	21,7397	21,8362	NaN	NaN	NaN	NaN
22,4292	NaN	NaN	NaN	NaN	23,4432	23,6475	NaN	22,7625
NaN	NaN	NaN	NaN	NaN	NaN	NaN	NaN	NaN
NaN	NaN	NaN	NaN	NaN	NaN	NaN	NaN	NaN
NaN	NaN	NaN	NaN	NaN	NaN	NaN	NaN	NaN
NaN	NaN	NaN	NaN	NaN	NaN	NaN	NaN	NaN
NaN	NaN	NaN	NaN	NaN	NaN	NaN	NaN	NaN
NaN	NaN	NaN	NaN	NaN	NaN	NaN	NaN	NaN
19,3729	NaN	NaN	NaN	NaN	NaN	NaN	NaN	NaN
NaN	NaN	NaN	20,5758	NaN	NaN	NaN	NaN	NaN
NaN	NaN	NaN	NaN	NaN	NaN	NaN	NaN	NaN
NaN	NaN	NaN	NaN	NaN	NaN	NaN	NaN	NaN
NaN	NaN	NaN	NaN	NaN	NaN	NaN	NaN	NaN
NaN	NaN	NaN	NaN	NaN	NaN	NaN	NaN	NaN
NaN	NaN	NaN	22,1478	22,4812	21,853	22,5534	NaN	NaN
NaN	NaN	NaN	NaN	NaN	NaN	NaN	NaN	NaN
NaN	NaN	NaN	NaN	NaN	NaN	NaN	NaN	NaN
NaN	NaN	NaN	NaN	NaN	NaN	NaN	NaN	NaN
22,2573	22,0061	21,972	21,7041	21,6714	23,3797	22,9424	23,238	22,8035
NaN	20,6651	NaN	NaN	NaN	NaN	NaN	NaN	NaN
NaN	NaN	NaN	21,0556	20,9163	21,0327	NaN	NaN	NaN
NaN	NaN	NaN	NaN	NaN	NaN	NaN	NaN	NaN
NaN	NaN	NaN	NaN	NaN	NaN	NaN	NaN	NaN
NaN	NaN	NaN	NaN	NaN	NaN	NaN	NaN	NaN
18,806	NaN	NaN	NaN	NaN	NaN	19,0401	NaN	NaN
NaN	NaN	NaN	NaN	NaN	NaN	NaN	NaN	NaN
NaN	NaN	NaN	NaN	NaN	NaN	NaN	NaN	NaN
NaN	NaN	NaN	NaN	NaN	NaN	NaN	NaN	NaN
23,3636	23,4362	23,5862	23,1603	23,1909	24,7358	24,6924	23,8705	23,9845
21,5562	21,8216	21,7913	NaN	21,2994	22,0558	21,5322	21,9162	22,2881
NaN	NaN	NaN	21,5141	NaN	22,5649	21,6273	NaN	NaN
21,7855	NaN	22,1954	22,0777	21,9614	22,9475	NaN	22,3008	NaN
NaN	NaN	20,528	20,6573	20,2838	21,6658	21,4274	20,4352	19,7356
NaN	NaN	NaN	NaN	NaN	NaN	NaN	21,3072	NaN

NaN	NaN	NaN	NaN	NaN	NaN	NaN	NaN	NaN
NaN	NaN	NaN	NaN	NaN	NaN	NaN	NaN	NaN
20,9134	NaN	NaN	NaN	NaN	20,6146	NaN	NaN	20,3027
20,601	NaN	NaN	21,2161	NaN	NaN	20,8444	NaN	NaN
NaN	NaN	NaN	NaN	NaN	NaN	NaN	NaN	NaN
25,7306	26,0766	25,8844	26,0428	25,9201	26,6535	26,7125	26,5563	26,7976
NaN	NaN	NaN	NaN	NaN	NaN	NaN	NaN	NaN
NaN	NaN	NaN	NaN	NaN	NaN	NaN	NaN	22,7347
NaN	NaN	NaN	NaN	NaN	NaN	NaN	NaN	NaN
NaN	NaN	NaN	NaN	NaN	20,1795	NaN	NaN	NaN
NaN	NaN	NaN	NaN	NaN	NaN	NaN	20,3871	NaN
NaN	NaN	NaN	NaN	NaN	NaN	NaN	NaN	NaN
NaN	NaN	NaN	NaN	NaN	NaN	NaN	NaN	NaN
20,4679	NaN	NaN	NaN	NaN	20,8602	20,7553	NaN	20,2228
23,5475	24,4889	24,7472	23,3022	23,2763	24,6311	24,6345	23,9044	23,9193
NaN	NaN	NaN	NaN	NaN	NaN	NaN	NaN	NaN
21,6706	21,0431	NaN	21,7758	22,0624	21,1939	21,0695	21,0064	21,0821
NaN	NaN	NaN	NaN	NaN	NaN	NaN	NaN	NaN
NaN	NaN	NaN	NaN	22,1727	23,0109	23,0268	24,2763	22,7759
NaN	NaN	NaN	NaN	NaN	22,6693	23,3991	NaN	NaN
NaN	NaN	NaN	NaN	NaN	NaN	NaN	NaN	NaN
NaN	NaN	NaN	NaN	NaN	23,663	23,106	NaN	NaN
22,4634	NaN	NaN	NaN	NaN	23,1674	23,6844	NaN	21,8934
NaN	NaN	NaN	NaN	NaN	22,4388	21,9977	NaN	NaN
NaN	NaN	NaN	NaN	NaN	21,4411	21,3937	NaN	NaN
NaN	NaN	NaN	NaN	NaN	NaN	NaN	NaN	20,9136
NaN	NaN	NaN	NaN	NaN	20,2946	NaN	20,3302	NaN
NaN	NaN	NaN	NaN	NaN	21,9098	21,883	NaN	NaN
20,1823	20,3878	21,0045	19,74	NaN	NaN	NaN	NaN	NaN
NaN	NaN	NaN	NaN	NaN	NaN	NaN	NaN	NaN
NaN	NaN	NaN	NaN	NaN	NaN	NaN	NaN	18,2971
NaN	NaN	NaN	NaN	NaN	20,2155	NaN	NaN	NaN
NaN	NaN	NaN	NaN	NaN	NaN	NaN	NaN	NaN
NaN	NaN	NaN	NaN	NaN	NaN	NaN	NaN	NaN
NaN	NaN	NaN	NaN	NaN	NaN	18,1252	NaN	NaN
NaN	NaN	NaN	NaN	NaN	NaN	NaN	NaN	NaN
NaN	NaN	NaN	NaN	NaN	NaN	NaN	NaN	NaN
NaN	NaN	NaN	NaN	NaN	NaN	NaN	NaN	NaN
19,2672	NaN	NaN	NaN	NaN	NaN	NaN	NaN	NaN
NaN	NaN	NaN	NaN	NaN	NaN	NaN	NaN	NaN
NaN	NaN	NaN	NaN	NaN	NaN	NaN	NaN	NaN
NaN	NaN	NaN	NaN	NaN	NaN	NaN	NaN	NaN
NaN	NaN	NaN	NaN	NaN	NaN	NaN	NaN	NaN
NaN	NaN	NaN	NaN	NaN	NaN	NaN	NaN	NaN
NaN	NaN	NaN	NaN	NaN	NaN	NaN	NaN	NaN
NaN	NaN	NaN	NaN	NaN	NaN	NaN	NaN	NaN
NaN	NaN	NaN	NaN	19,3952	NaN	NaN	NaN	NaN
NaN	NaN	NaN	NaN	NaN	NaN	NaN	NaN	NaN
NaN	NaN	NaN	NaN	NaN	20,1286	19,8103	NaN	NaN
NaN	NaN	NaN	20,479	20,899	NaN	20,0558	19,645	NaN
NaN	NaN	NaN	NaN	NaN	NaN	NaN	NaN	NaN
NaN	NaN	NaN	NaN	NaN	NaN	NaN	NaN	NaN

19,8632	NaN	NaN	NaN	21,5892	NaN	NaN	NaN	NaN
NaN	NaN	NaN	NaN	NaN	NaN	NaN	NaN	NaN
21,024	NaN	NaN	NaN	NaN	NaN	21,6783	NaN	NaN
NaN	NaN	NaN	NaN	NaN	NaN	NaN	NaN	NaN
NaN	NaN	NaN	NaN	NaN	NaN	NaN	NaN	NaN
NaN	NaN	NaN	NaN	NaN	NaN	NaN	NaN	NaN
NaN	NaN	NaN	NaN	NaN	NaN	NaN	NaN	NaN
NaN	NaN	NaN	NaN	NaN	NaN	NaN	NaN	NaN
NaN	NaN	NaN	NaN	NaN	NaN	NaN	NaN	NaN
NaN	NaN	NaN	NaN	NaN	NaN	18,9012	NaN	NaN
NaN	NaN	NaN	NaN	NaN	NaN	NaN	19,6589	19,4484
NaN	NaN	NaN	NaN	NaN	NaN	NaN	NaN	NaN
NaN	NaN	NaN	NaN	NaN	NaN	NaN	NaN	NaN
NaN	NaN	NaN	NaN	NaN	NaN	NaN	NaN	NaN
NaN	NaN	NaN	NaN	NaN	NaN	NaN	NaN	NaN
NaN	NaN	NaN	NaN	NaN	NaN	NaN	NaN	NaN
NaN	NaN	NaN	NaN	NaN	NaN	NaN	NaN	NaN
NaN	NaN	NaN	NaN	NaN	NaN	NaN	NaN	NaN
NaN	NaN	NaN	NaN	NaN	NaN	NaN	NaN	NaN
20,2467	NaN	20,1467	20,8405	21,6107	NaN	NaN	NaN	NaN
NaN	NaN	NaN	NaN	NaN	NaN	NaN	NaN	NaN
NaN	NaN	NaN	NaN	NaN	NaN	NaN	NaN	NaN
NaN	NaN	NaN	NaN	NaN	NaN	NaN	NaN	NaN
NaN	NaN	NaN	NaN	NaN	NaN	NaN	NaN	NaN
NaN	NaN	NaN	NaN	NaN	NaN	NaN	NaN	NaN
NaN	NaN	NaN	NaN	NaN	NaN	NaN	NaN	NaN
23,686	24,4979	24,1185	NaN	NaN	24,3506	24,6991	25,3321	25,7048
NaN	NaN	NaN	NaN	NaN	NaN	NaN	NaN	NaN
22,6298	NaN	NaN	NaN	NaN	21,7886	NaN	NaN	22,1953
20,816	21,6453	21,4899	NaN	NaN	19,3588	20,2201	20,4001	20,5702
NaN	NaN	NaN	NaN	NaN	NaN	NaN	NaN	NaN
NaN	NaN	NaN	NaN	NaN	NaN	NaN	NaN	NaN
NaN	NaN	NaN	NaN	NaN	NaN	NaN	NaN	NaN
NaN	NaN	NaN	NaN	NaN	NaN	NaN	NaN	NaN
NaN	NaN	NaN	22,1555	21,9771	NaN	NaN	NaN	NaN
17,6443	NaN	NaN	NaN	NaN	NaN	NaN	NaN	NaN
NaN	NaN	NaN	23,3372	24,0926	NaN	NaN	NaN	NaN
NaN	NaN	NaN	NaN	NaN	NaN	NaN	NaN	NaN
NaN	NaN	NaN	NaN	NaN	NaN	18,5047	NaN	NaN
NaN	NaN	NaN	NaN	NaN	NaN	NaN	NaN	NaN
23,8868	24,0157	NaN	24,9427	24,9066	22,4887	22,9745	NaN	NaN
NaN	NaN	NaN	NaN	NaN	NaN	NaN	NaN	NaN
NaN	NaN	NaN	NaN	NaN	NaN	NaN	NaN	NaN
NaN	NaN	NaN	NaN	NaN	NaN	NaN	NaN	NaN
NaN	NaN	NaN	NaN	NaN	NaN	NaN	NaN	NaN
NaN	NaN	NaN	NaN	NaN	NaN	NaN	NaN	NaN
19,7804	NaN	NaN	NaN	NaN	NaN	NaN	NaN	NaN
22,6661	NaN	NaN	NaN	NaN	NaN	NaN	NaN	NaN
NaN	NaN	NaN	NaN	NaN	NaN	NaN	NaN	NaN
NaN	NaN	NaN	NaN	NaN	NaN	NaN	NaN	NaN
NaN	NaN	NaN	NaN	NaN	NaN	NaN	NaN	NaN
NaN	NaN	NaN	NaN	NaN	NaN	NaN	NaN	NaN

20,5293	NaN	NaN	NaN	20,9487	NaN	NaN	NaN	21,1917
NaN	NaN	NaN	NaN	NaN	NaN	NaN	NaN	NaN
23,5998	NaN	NaN	26,7752	26,6806	NaN	NaN	NaN	23,4057
31,3583	25,9361	25,8676	30,0646	31,482	23,5978	NaN	27,5214	28,0905
NaN	NaN	NaN	NaN	NaN	NaN	NaN	NaN	NaN
NaN	21,2991	NaN	NaN	NaN	NaN	NaN	NaN	NaN
NaN	NaN	NaN	NaN	NaN	NaN	NaN	NaN	NaN
NaN	NaN	NaN	NaN	NaN	NaN	NaN	NaN	NaN
NaN	NaN	NaN	NaN	NaN	NaN	NaN	NaN	NaN
NaN	NaN	NaN	NaN	NaN	NaN	NaN	NaN	NaN
NaN	NaN	NaN	NaN	NaN	NaN	NaN	NaN	NaN
NaN	NaN	20,4092	NaN	NaN	NaN	NaN	NaN	NaN
23,8006	24,4843	24,3765	25,0352	25,138	24,9281	24,9983	24,7998	25,2547
NaN	NaN	NaN	NaN	NaN	NaN	NaN	NaN	NaN
NaN	NaN	NaN	NaN	NaN	NaN	NaN	NaN	NaN
24,3527	25,2094	25,1187	25,759	NaN	NaN	NaN	NaN	NaN
24,5959	23,5714	NaN	NaN	25,4373	24,3697	NaN	NaN	NaN
NaN	NaN	NaN	NaN	NaN	NaN	19,8148	NaN	NaN
NaN	NaN	NaN	NaN	NaN	NaN	NaN	NaN	NaN
21,515	NaN	20,3616	19,909	NaN	20,3299	20,4315	21,7754	21,3854
20,5791	NaN	NaN	NaN	NaN	NaN	20,8169	NaN	NaN
NaN	NaN	NaN	NaN	NaN	NaN	NaN	NaN	NaN
NaN	NaN	NaN	NaN	NaN	NaN	NaN	NaN	NaN
NaN	NaN	NaN	17,6262	NaN	NaN	NaN	NaN	NaN
NaN	NaN	NaN	NaN	NaN	NaN	NaN	NaN	NaN
NaN	NaN	NaN	NaN	NaN	NaN	NaN	NaN	NaN
18,6114	NaN	NaN	NaN	NaN	NaN	NaN	NaN	NaN
NaN	NaN	NaN	NaN	NaN	NaN	NaN	NaN	NaN
NaN	NaN	NaN	NaN	NaN	NaN	NaN	NaN	NaN
19,5732	NaN	NaN	NaN	NaN	NaN	NaN	NaN	NaN
NaN	NaN	NaN	NaN	NaN	NaN	NaN	NaN	NaN
NaN	NaN	NaN	NaN	NaN	NaN	NaN	NaN	NaN
25,5504	26,0116	25,9224	26,9687	26,7817	27,1892	27,481	26,6475	26,6546
NaN	NaN	NaN	NaN	18,7357	NaN	NaN	NaN	NaN
22,938	21,31	21,758	22,9735	23,1031	22,9266	22,5539	22,1839	NaN
NaN	NaN	NaN	NaN	NaN	NaN	NaN	NaN	NaN
NaN	NaN	NaN	NaN	NaN	NaN	NaN	NaN	NaN
21,1949	NaN	NaN	21,4129	21,071	21,8136	21,7822	21,5662	NaN
NaN	NaN	NaN	NaN	NaN	NaN	NaN	NaN	NaN
NaN	NaN	NaN	NaN	NaN	NaN	NaN	NaN	NaN
NaN	NaN	NaN	NaN	NaN	NaN	NaN	NaN	20,433
21,9114	NaN	22,5972	23,0363	22,7017	23,5005	23,1161	NaN	23,0523
NaN	26,1272	25,5934	NaN	24,9199	26,3614	25,8583	26,4613	25,7348
NaN	NaN	NaN	NaN	NaN	NaN	NaN	NaN	NaN
NaN	NaN	NaN	NaN	NaN	NaN	NaN	NaN	NaN
NaN	NaN	NaN	NaN	NaN	NaN	NaN	NaN	NaN
NaN	NaN	NaN	NaN	NaN	26,5666	26,4923	25,9978	25,5191
NaN	NaN	NaN	NaN	NaN	NaN	19,3558	NaN	NaN
NaN	NaN	NaN	NaN	NaN	NaN	NaN	NaN	NaN
NaN	NaN	NaN	NaN	NaN	21,6796	NaN	22,2582	NaN
NaN	NaN	NaN	NaN	NaN	NaN	NaN	NaN	NaN
19,8163	NaN	NaN	NaN	NaN	NaN	NaN	NaN	NaN

NaN	NaN	NaN	NaN	NaN	NaN	NaN	NaN	NaN
21,8156	24,4366	24,7031	22,3581	22,4496	NaN	NaN	NaN	NaN
21,703	22,0132	22,2787	22,3315	22,2079	22,711	22,3209	22,0791	21,8251
NaN	NaN	NaN	NaN	NaN	19,5334	20,1526	NaN	NaN
NaN	NaN	NaN	NaN	NaN	NaN	NaN	NaN	NaN
NaN	NaN	NaN	NaN	NaN	NaN	NaN	NaN	NaN
NaN	NaN	NaN	NaN	NaN	NaN	NaN	NaN	NaN
23,0412	21,131	21,0804	21,121	21,383	22,1792	22,4356	23,1239	23,0324
NaN	NaN	NaN	NaN	NaN	NaN	NaN	NaN	NaN
19,961	NaN	NaN	NaN	NaN	NaN	NaN	NaN	NaN
NaN	NaN	NaN	NaN	NaN	NaN	NaN	NaN	NaN
NaN	26,1296	26,1305	25,9251	26,0054	26,8799	26,7798	26,0416	26,1447
NaN	NaN	NaN	NaN	NaN	NaN	NaN	NaN	NaN
21,8865	NaN	NaN	23,1866	NaN	NaN	NaN	NaN	NaN
NaN	NaN	NaN	NaN	NaN	NaN	24,6504	NaN	NaN
21,6403	NaN	NaN	NaN	NaN	21,3893	21,5366	NaN	NaN
23,7803	NaN	NaN	22,8353	22,7196	NaN	NaN	24,3312	24,4788

LFQ intensi	LFQ intensi	LFQ intensi	LFQ intensi	N: Razor + u	N: Mol. wei	T: Protein I	T: Majority	T: Protein n
31,9748	31,8973	32,6499	32,5921	109	596,33	Q9HC84;A7	Q9HC84;A7	Mucin-5B
34,1907	34,2089	32,4538	32,6138	90	69,366	CON__P02	CON__P02	Serum albu
26,243	26,1283	27,7247	27,7568	90	572,01	Q9Y6R7;A0	Q9Y6R7;A0	IgGFc-bindi
16,617	16,8323	28,6095	28,5137	89	331,77	P15924;Q4	P15924;Q4	Desmoplak
28,0723	28,0897	26,889	26,8034	88	187,15	V9HWA9;P	V9HWA9;P	Complemer
25,3532	25,3647	26,7834	26,7563	78	226,53	P35579;A0	P35579;A0	Myosin-9
21,451	21,7565	27,6918	27,7578	71	629,09	Q09666;Q6	Q09666	Neuroblast
27,6463	27,6705	27,3349	27,2906	67	163,29	P01023;H0	P01023	Alpha-2-ma
29,9693	30,0388	27,5515	27,4545	62	74,831	B4E1B2	B4E1B2	
29,6585	29,6557	26,5493	26,5973	55	78,181	V9HWI4;A0	V9HWI4;A0	Lactotransf
NaN	NaN	27,4276	27,1978	55	204,49	K7EKI8;O6	K7EKI8;O6	Periplakin
33,1143	33,2541	31,5472	31,4142	54	57,767	P04745;B7	P04745;B7	Alpha-amyl
32,7594	32,6754	31,5433	31,4954	52	83,283	P01833	P01833	Polymeric i
28,9346	29,0044	30,8218	30,8555	51	65,969	H6VRG0;H	H6VRG0;H	Keratin, typ
25,3994	25,4369	26,9071	26,7516	49	104,85	O43707;A0	O43707;A0	Alpha-actir
26,3606	26,4183	28,1062	28,1048	49	161,1	A8K2U0;H0	A8K2U0;H0	Alpha-2-ma
28,0629	28,0419	26,96	27,0281	48	70,289	V9HWJ7;A0	V9HWJ7;A0	Plastin-2
26,0029	26,0721	33,3969	33,1768	48	63,91	CON__P19	CON__P19	Keratin, typ
26,1623	26,0905	32,0474	32,0742	43	59,998	CON__P04	CON__P04259;B4DKV4	
23,372	23,477	24,3747	24,3017	43	280,74	P21333;Q6	P21333;Q6	Filamin-A
26,7803	26,5759	33,6443	33,5596	42	49,586	CON__P13	CON__P13646-1;A1A4	
25,6227	25,6824	24,977	25,0844	40	97,147	P06737;E9	P06737;E9	Glycogen p
28,219	28,2469	25,6369	25,394	38	80,287	P22079;F5	P22079;F5	Lactoperox
26,2175	26,2381	25,6346	25,6493	38	67,819	V9HWC0;P	V9HWC0;P	Moesin
27,2765	27,3179	28,1857	28,3044	38	62,064	P35527;C0	P35527;C0	Keratin, typ
26,7708	26,7933	25,2746	25,8171	37	122,2	A5PL27;P0	A5PL27;P0	Ceruloplasr
26,9235	26,8585	28,1761	28,2366	37	65,432	CON__P35	CON__P35	Keratin, typ
24,1427	24,1149	23,0225	22,6256	36	271,42	A0A1S5UZ0	A0A1S5UZ0	Talin-1
27,2491	27,1601	27,0999	27,1875	36	83,868	P05164;J3	P05164	Myeloperox
24,832	24,992	30,0811	30,0345	35	51,267	CON__P08	CON__P08	Keratin, typ
26,5482	26,5466	25,0241	25,1453	35	67,877	V9HWD9;P	V9HWD9;P	Transketola
26,4594	26,6826	27,5995	27,614	35	18,154	Q9UBC9;B1	Q9UBC9;B1	Small prolir
26,8803	26,8781	26,389	26,6527	34	57,936	P14618;V9	P14618;V9	Pyruvate ki
24,263	24,2155	27,6028	27,8022	34	76,631	Q08188	Q08188	Protein-glu
22,737	23,8518	25,0213	24,9541	33	189,28	A4QPB0;A0	A4QPB0;A0	Ras GTPase-
26,8877	26,7873	27,1996	27,0649	33	70,051	P0DMV8;P	P0DMV8;P	Heat shock
28,1526	28,1401	27,7636	27,88	32	47,168	P06733;A0	P06733;A0	Alpha-enola
26,5033	26,4786	25,4964	25,5533	32	71,553	B7Z507	B7Z507	
28,1308	28,0809	25,4789	25,5524	32	55,928	V9HVY1;P0	V9HVY1;P0	Fibrinogen
26,8789	26,954	25,8429	26,043	31	59,99	B4DVJ0;K7	B4DVJ0	Glucose-6-p
28,301	28,4507	29,5842	29,6575	31	59,51	CON__P13	CON__P13	Keratin, typ
25,6571	25,6914	27,2863	27,3643	30	57,116	P07237;A0	P07237;A0	Protein disu
25,8005	25,753	25,3296	25,5388	30	107,53	P32926;A0	P32926;A0	Desmoglein
26,3345	26,333	25,3734	25,137	30	69,284	A0A140VK	A0A140VK	Leukotrien
25,1076	25,0678	24,526	24,755	30	50,663	Q6IAT1;B4	Q6IAT1;B4	Rab GDP di
27,1324	27,3211	26,7512	26,5335	30	78,831	B7Z992;A0	B7Z992;A0A0A0MS51;	
24,5017	24,6945	25,6611	25,4883	30	72,332	V9HWB4;P	V9HWB4;P	78 kDa gluc
22,6663	23,0444	24,3975	24,5023	30	127,18	P11215;B3	P11215	Integrin alp
29,7366	29,846	29,0221	29,2287	30	260,73	Q9UGM3;B	Q9UGM3	Deleted in r
16,8516	16,5753	26,6664	27,1226	29	81,744	A0A0S2Z48	A0A0S2Z48	Junction pl
25,6149	25,6323	24,4593	24,6407	29	192,78	P0C0L4	P0C0L4	Complemer

26,6898	26,709	25,9955	26,0663	28	51,672	D3DPU2;B2	D3DPU2;B2	Adenylyl cy
33,2579	33,1127	31,6675	31,5426	28	53,088	Q96K68	Q96K68	
22,3713	21,8504	22,8305	22,839	27	69,293	CON__P027	CON__P02769	
25,3159	25,2055	24,8136	25,2452	27	59,755	P04040;B4	P04040;B4	Catalase
NaN	NaN	24,4066	25,206	26	231,63	A0A024R8I	A0A024R8I	Envoplakin
25,2796	25,5132	25,8777	25,9742	26	70,897	P11142;V9	P11142;V9	Heat shock
22,1123	22,5282	24,548	24,4144	26	103,28	P55786;E9	P55786;E9	Puromycin
26,6116	26,7227	25,9591	25,9472	26	44,614	V9HWF4;P(	V9HWF4;P(	Phosphogly
25,5144	25,6453	30,9489	30,7181	26	38,714	Q5TZZ9;P0	Q5TZZ9;P0	Annexin;Ar
22,9998	23,2368	25,9877	25,7587	26	68,478	P07476;B4	P07476;B4	Involucrin
21,8186	22,5175	25,3206	25,4184	26	53,651	V9HWE1;P(	V9HWE1;P(	Vimentin
23,2853	23,1025	24,1513	24,2664	26	120,71	Q9NQ38;E7	Q9NQ38;E7	Serine prot
27,8878	27,8849	26,555	26,5049	25	30,777	A0A024R3I	A0A024R3I	Apolipopro
24,7223	24,6915	23,8033	24,6369	25	59,256	P11413;A8	P11413;A8	Glucose-6-p
24,9278	24,8633	23,6729	23,8169	25	140,94	B4E1Z4;P0(	B4E1Z4;P0(	Complemer
24,1016	24,2253	28,9347	28,9473	25	62,378	CON__P13(	CON__P13(	Keratin, typ
26,884	26,8123	26,5183	26,3418	25	39,42	V9HWN7;P	V9HWN7;P	Fructose-bi
22,4767	22,5302	25,6229	25,2321	25	95,337	P13639;Q8	P13639;Q8	Elongation
30,3628	30,5859	29,3137	29,5881	25	41,792	P63261;B4	P63261;B4	Actin, cyto
23,0162	23,2009	23,6814	23,4606	25	68,283	Q96G03;B4	Q96G03;B4	Phosphoglu
23,5691	23,1291	22,8271	23,1515	24	116,72	A0A024QZI	A0A024QZI	Vinculin
24,2129	23,8296	24,6176	24,412	24	103,06	P12814;A0	P12814;A0	Alpha-actir
25,0429	24,9296	24,356	24,7665	24	29,032	A0A0S2Z4C	A0A0S2Z4C	Tropomyos
28,7559	28,7038	27,2394	27,2848	24	34,258	A0A140VK(	A0A140VK(	Zinc-alpha-
23,6794	23,7981	23,5434	23,7586	24	223,02	A5YM51;P1	A5YM51;P1	Myosin-7;M
26,7857	26,6449	29,2958	29,2172	24	53,533	Q9UBG3;A8	Q9UBG3;A8	Cornulin
24,2492	24,2899	25,6448	25,622	24	84,659	K9JA46;P0	K9JA46;P0	Heat shock
24,772	24,7631	23,2253	23,2342	24	66,193	V9HWG7;C	V9HWG7;C	WD repeat-
26,9068	26,9636	26,6402	26,6756	24	36,688	P00338;V9	P00338;V9	L-lactate de
26,5	26,5064	25,0106	25,1068	24	52,963	V9HWI6;P(	V9HWI6;P(	Vitamin D-l
26,4752	26,5112	26,4886	26,5229	24	42,741	V9HWH1;P	V9HWH1;P	Leukocyte e
NaN	NaN	28,1533	28,3568	24	56,865	Q8N1N4;C(	Q8N1N4;C(	Keratin, typ
24,291	24,151	22,1959	22,8474	23	138,95	A8K5T0;A0	A8K5T0;A0	Complemer
28,2611	28,4092	26,308	25,9252	23	49,496	A0A140VJJ	A0A140VJJ	Fibrinogen
24,0719	24,0707	22,4713	22,663	23	53,907	Q53GX6;A8	Q53GX6;A8	Nucleobinc
NaN	NaN	24,4338	24,5065	23	515,55	C0JYY2;P04	C0JYY2;P04	Apolipopro
28,0822	28,0154	25,5906	25,7772	23	36,053	V9HVZ4;P0	V9HVZ4;P0	Glyceraldeh
24,1378	24,2062	24,0567	24,2787	23	60,54	Q6UWP8;K	Q6UWP8	Suprabasin
27,7462	27,7696	27,7792	28,0377	23	52,441	Q8TDL5	Q8TDL5	BPI fold-cor
19,9855	NaN	28,3296	28,3046	22	38,604	P07355;A0	P07355;A0	Annexin A2
28,7976	28,9584	27,2135	27,7375	22	51,596	A8K008	A8K008	
NaN	NaN	26,2097	25,9241	22	74,139	P02545;W8	P02545;W8	Prelamin-A
26,0686	25,9541	23,888	24,0971	21	58,112	A0A024QZ(	A0A024QZ(	Prosaposin
23,1847	23,5686	22,1876	22,4029	21	99,024	A0A024R7I	A0A024R7I	Hexokinase
26,3334	26,2962	24,6637	24,4771	21	50,222	V9HW75;A	V9HW75;A	Nucleobinc
25,4319	25,5331	25,6636	25,4742	21	99,949	A9X9L0;A8	A9X9L0;A8	Desmocolli
28,6832	28,7111	26,8126	26,9362	21	45,205	P00738;HC	P00738;HC	Haptoglobi
27,1419	27,2371	25,1914	25,4377	21	51,676	P02790;Q9	P02790	Hemopexir
27,1116	27,2495	27,1715	26,9164	21	53,139	P52209;B4	P52209;B4	6-phosphog
31,8639	31,8501	30,0817	29,9855	21	25,834	Q6PIL8;Q6I	Q6PIL8;Q6PJF2	
32,4702	32,5819	28,791	28,7057	20	16,214	P01036	P01036	Cystatin-S
25,7863	25,6885	28,0654	28,2023	20	27,774	P31947;Q3	P31947	14-3-3 prot

23,2352	23,143	22,7519	22,7798	20	61,448	P36871;B4	P36871;B4	Phosphoglu
NaN	NaN	28,4924	28,4281	20	65,84	Q01546	Q01546	Keratin, typ
25,1696	25,2533	22,8331	21,5702	19	57,279	Q6UX06;A(	Q6UX06;A(	Olfactomed
23,6533	23,9215	24,2556	24,0375	19	117,85	A0A024R1/	A0A024R1/	Ubiquitin-l
24,0842	24,1328	25,7437	25,8494	19	42,1	A0A024R2I	A0A024R2I	Serpin B5
25,8859	25,9222	25,2965	24,9487	19	51,026	A0A024R6:	A0A024R6:	Coronin;Cc
24,544	24,3462	23,4573	23,4731	19	55,52	A0A024R7:	A0A024R7:	Nicotinami
26,0719	25,8342	23,7739	23,6121	19	75,207	A0A024RDI	A0A024RDI	SPARC-like
26,0775	26,0443	25,875	25,8958	19	37,54	A0A140VK!	A0A140VK!	Transaldola
28,8046	28,8239	26,2102	26,3761	19	51,923	A0A1B0GU	A0A1B0GU	Ig mu chair
NaN	NaN	NaN	NaN	19	187,37	CON__Q2U	CON__Q2UVX4	
23,8759	23,8493	23,7641	24,2439	19	82,577	O00391;A(	O00391;A(	Sulfhydryl c
25,9336	26,0591	25,7943	25,6763	19	26,669	V9HWK1;P	V9HWK1;P	Triosephos
24,3215	24,3892	24,9927	24,9036	19	113,75	Q02413	Q02413	Desmoglein
24,3395	24,3947	24,3035	23,9125	18	47,371	P61158;A0	P61158;A0	Actin-relate
26,8701	26,7641	25,2428	25,5388	18	65,33	A0A0S2Z3Y	A0A0S2Z3Y	Galectin-3-
NaN	22,2115	23,9912	23,4471	18	54,102	B3KQT9;V9	B3KQT9;V9	Protein dis
21,6281	21,1977	24,7658	24,6018	18	67,599	B4DPW9;P	B4DPW9;P	Plastin-3
24,6389	24,5279	22,3401	22,3107	18	90,568	P00747;B2	P00747;B2	Plasminoge
21,5975	21,5756	24,2394	24,5615	18	84,781	P05107;D3	P05107;D3	Integrin be
22,3906	22,9445	23,51	23,7234	17	49,416	A0A024RC8	A0A024RC8	Ribonuclea
25,1063	25,0786	23,6583	23,7816	17	44,552	P07339;V9	P07339;V9	Cathepsin D
NaN	NaN	25,4995	25,5813	17	89,798	B0AZN7;A8	B0AZN7;A8	Protein-glu
25,9151	25,8428	27,5969	27,4147	17	15,164	E7DVW5;Q	E7DVW5;Q	Fatty acid-b
21,2671	NaN	19,9013	NaN	17	51,681	Q13231;D6	Q13231;D6	Chitotriosi
23,1954	23,1226	25,2117	25,3901	17	54,392	Q96HE7;G3	Q96HE7	ERO1-like p
26,892	26,9545	25,5174	25,4707	16	22,988	A0A024RA9	A0A024RA9	Rho GDP-di
24,8547	24,7676	NaN	18,4669	16	163,87	B2R950;P2	B2R950;P2	Pregnancy z
NaN	NaN	24,2684	24,1458	16	56,291	B9EKV4;P4	B9EKV4;P4	4-trimethyl
23,7807	23,8846	26,6653	27,0536	16	51,621	CON__P02!	CON__P02!	Keratin, typ
23,0582	23,1787	24,038	24,1017	16	69,412	P15311;E7	P15311;E7	Ezrin;Tyrosi
30,6995	30,8283	30,258	29,6674	16	18,098	P01591;D6	P01591;D6	Immunoglo
26,911	26,8691	24,6383	24,2872	16	94,972	P02671;A0	P02671;A0	Fibrinogen
25,2947	25,1204	24,9313	25,0783	16	28,82	Q6P6D7;Q6	Q6P6D7;Q6	Phosphogly
24,1161	24,227	25,3801	25,3492	16	25,035	V9HWC7;P	V9HWC7;P	Peroxiredo
22,8809	22,5723	24,1294	24,0556	16	49,83	P68371;Q8	P68371;Q8	Tubulin bet
23,4276	23,3656	26,4054	26,1405	16	22,11	Q06830;Q5	Q06830;Q5	Peroxiredo
30,1844	30,2307	28,1	27,9979	16	37,926	Q6P5S2	Q6P5S2	Protein LEG
29,1028	29,005	28,0968	27,9226	16	49,172	Q8N4F0;B4	Q8N4F0;B4	BPI fold-cor
NaN	NaN	24,1918	24,2638	15	72,364	A8K2N8;B4	A8K2N8;B4DRX0;B4DR	
24,0613	24,2777	23,6259	23,8912	15	45,333	B3KNK9;Q8	B3KNK9;Q8	Golgi meml
26,8859	26,8669	25,1284	25,0747	15	15,998	P68871;D9	P68871;D9	Hemoglobi
24,6065	24,3532	24,2028	24,2345	15	46,736	P01009;E9	P01009;E9	Alpha-1-an
23,7417	23,8892	23,4536	23,9213	15	34,333	Q53R19;O1	Q53R19;O1	Actin-relate
24,2536	24,233	23,2815	23,4979	15	32,118	V9HWH6;C	V9HWH6;C	Purine nucl
31,6363	31,5123	27,9749	27,7352	15	16,387	P01037	P01037	Cystatin-SN
22,1498	21,6637	26,0209	26,071	15	36,375	P12429;D6	P12429;D6	Annexin A3
NaN	NaN	20,8691	20,5989	15	62,52	P23141;B7	P23141;B7	Liver carbo
NaN	23,047	25,1658	24,9186	15	46,918	Q53G71;V9	Q53G71;V9	Calreticulir
21,2063	21,771	23,7914	23,9282	15	44,564	P29508	P29508	Serpin B3
23,656	23,0039	24,3342	24,4299	15	57,509	Q6PKA6;P3	Q6PKA6;P3	Aldehyde d
22,4214	22,358	23,0895	22,6794	15	37,375	Q9UJ70;H7	Q9UJ70;H7	N-acetyl-D-

22,3284	21,7621	23,2975	23,1644	14	81,467	A0A024R5/	A0A024R5/	Calpain-1 c
28,0616	27,7988	27,8003	27,5932	14	19,25	A0A024R8I	A0A024R8I	Lipocalin-1
NaN	NaN	24,2869	23,7471	14	192,06	A0A087WV	A0A087WV	Clathrin he
NaN	NaN	NaN	NaN	14	96,19	B3KQS1;B3	B3KQS1;B3	Prominin-1
21,7957	21,6113	23,4361	23,6517	14	60,673	A0A140VJI	A0A140VJI	Extracellula
21,5145	22,297	21,5048	21,1144	14	68,168	A6NKB8;Q7	A6NKB8;Q7	Aminopept
29,6853	29,7141	27,3603	27,9589	14	16,537	B2R4C5;P6	B2R4C5;P6	Lysozyme;L
26,3441	26,5083	27,7977	27,5563	14	27,745	D0PNI1;P6	D0PNI1;P6	14-3-3 prot
24,2415	24,1585	25,7129	25,5569	14	19,591	J3KNB4;P4	J3KNB4;P4	Cathelicidi
32,8931	32,6378	29,7021	29,6071	14	16,572	P12273	P12273	Prolactin-ir
23,8292	23,8615	22,3172	21,8733	14	108,33	P19021;B4	P19021;B4	Peptidyl-gl
23,4863	22,4854	24,2394	24,3714	14	46,872	P21128;B7	P21128;B7	Poly(U)-spe
30,105	30,0503	29,4574	29,5143	14	27,011	Q96DR5;A8	Q96DR5;A8	BPI fold-co
23,1378	23,0509	24,7428	24,6296	14	44,276	Q9UIV8;B7	Q9UIV8;B7	Serpin B13
22,2909	23,1758	22,1495	22,4463	14	223,04	Q9UKX2;P1	Q9UKX2;P1	Myosin-2;M
29,9212	29,8814	28,9164	29,1085	14	24,823	S6BGD6	S6BGD6	
24,1686	23,8671	23,5551	23,1695	13	32,922	P52907;A0	P52907;A0	F-actin-cap
23,3589	23,4315	22,151	21,9637	13	41,92	A0A024R1	A0A024R1	Synaptic ve
22,2663	22,3449	20,8535	NaN	13	262,62	P02751;A0	P02751;A0	Fibronectir
NaN	NaN	24,2184	24,5737	13	80,496	A0A024R9	A0A024R9	Plakophilir
21,1396	NaN	NaN	NaN	13	46,247	A0A140VK	A0A140VK	Aspartate a
20,9194	21,8021	20,8021	20,8683	13	46,659	V9HWJ2;B	V9HWJ2;B	Isocitrate d
23,8772	23,8139	23,7687	23,8367	13	30,656	B2R7T8;B1	B2R7T8;B1	F-actin-cap
NaN	20,7555	21,5393	21,2524	13	46,087	C9JGI3;E5K	C9JGI3;E5K	Thymidine
23,3316	23,5694	23,0314	23,752	13	47,311	B3KXD3;P1	B3KXD3;P1	Carboxype
24,8319	24,4634	23,1999	22,6082	13	56,852	B4DJI2;P2	B4DJI2;P2	Granulins;
24,935	24,857	20,0485	21,1373	13	46,496	B4DPP8;D	B4DPP8;D	Kininogen-
28,5314	28,6987	26,8148	26,8062	13	35,789	B4DUH8;P	B4DUH8;P	Carbonic ar
24,0595	24,0987	24,5596	24,3391	13	26,922	Q5SRT3;Q5	Q5SRT3;Q5	Chloride in
22,8784	22,6219	23,6954	23,9066	13	40,45	P04899;B3	P04899;B3	Guanine nu
29,9798	29,905	29,418	29,5668	13	10,834	P05109	P05109	Protein S1C
23,9662	24,0651	23,4308	23,2634	13	36,638	Q5U077;P0	Q5U077;P0	L-lactate de
27,7912	27,8007	26,177	26,0882	13	15,054	P07737;K7	P07737;K7	Profilin-1
26,9553	26,9598	26,9619	26,4483	13	23,356	V9HWE9;P	V9HWE9;P	Glutathion
26,7197	26,7798	25,6935	25,759	13	18,502	V9HWI5;P2	V9HWI5;P2	Cofilin-1
23,2538	23,0387	22,9932	23,4748	13	44,76	P61160;Q8	P61160;Q8	Actin-relate
NaN	NaN	22,4747	22,3064	13	101,28	Q14CN2;Q	Q14CN2	Calcium-ac
22,9687	22,8718	23,5398	23,4959	12	56,94	Q16851;B4	Q16851;B4	UTP--gluco
22,9908	23,2686	22,1026	NaN	12	28,723	A0A0K0K1I	A0A0K0K1I	Proteasom
23,2547	22,9856	21,9709	21,5454	12	105,21	Q5T985;A2	Q5T985;A2	Inter-alpha
24,5916	25,103	22,9331	22,4651	12	53,467	A8K9E4;P2	A8K9E4;P2	Neutrophil
26,045	25,9286	23,8301	23,5024	12	22,788	X6R8F3;B2	X6R8F3;B2	Neutrophil
24,1162	24,0341	24,3511	24,1471	12	21,057	D9IAI1;P30	D9IAI1;P30	Phosphatic
27,3205	27,0746	25,8385	25,7926	12	27,63	P54108;J3I	P54108;J3I	Cysteine-ric
21,7845	21,5101	23,3496	23,4661	12	36,019	O60218;A4	O60218	Aldo-keto r
NaN	NaN	24,8364	24,5587	12	77,551	O95171	O95171	Sciellin
24,5732	24,4288	22,4247	21,9135	12	54,253	V9HWD8;P	V9HWD8;P	Alpha-1B-g
23,2807	23,767	20,5164	21,8571	12	34,735	P05089	P05089	Arginase-1
19,1243	NaN	24,0564	23,9797	12	48,113	Q0QEN7;V	Q0QEN7;V	ATP syntha
25,9614	25,8615	24,9179	25,087	12	52,494	P10909;E7	P10909;E7	Clusterin;C
23,3696	23,67	24,2458	23,9926	12	22,391	P37802;X6	P37802;X6	Transgelin-
NaN	NaN	23,8385	23,5433	12	89,321	V9HW80;P	V9HW80;P	Transitiona

21,1037	21,3963	23,5996	24,3994	11	31,969	Q0QF37;Q7	Q0QF37;Q7	Malate deh
21,7623	23,3905	20,1842	20,2897	11	50,097	P30520;A0	P30520;A0	Adenylosuc
20,5564	NaN	24,0542	23,6613	11	68,898	A0A024R6X	A0A024R6X	Carboxylic
22,9931	23,0347	21,6323	21,247	11	36,748	A0A024R9(	A0A024R9(	Protein FAM
21,4411	21,3139	23,3192	22,9428	11	129,35	A0A024R9(	A0A024R9(	Thrombosp
21,7347	22,1536	23,3221	23,2357	11	83,263	A0A024RD8	A0A024RD8	Heat shock
20,7464	20,4105	20,909	21,3961	11	40,005	A0A0C4DG	A0A0C4DG	Alcohol deh
21,6437	NaN	22,1332	22,127	11	76,572	P20810;A0	P20810;A0	Calpastatin
32,3985	32,3548	31,6968	32,0503	11	19,6	A0A0C4DG	A0A0C4DG	Zymogen gr
27,2854	27,1035	23,8815	23,9252	11	15,799	A0A0K0K1J	A0A0K0K1J	Cystatin-C
21,4958	21,6584	22,0053	22,1895	11	46,946	A0A140VJN	A0A140VJN	Perilipin-3
21,742	21,5767	22,5828	21,2964	11	27,399	A0A140VK4	A0A140VK4	Proteasome
26,2786	26,214	25,6433	25,8562	11	18,012	V9HWF5;A8	V9HWF5;A8	Peptidyl-pr
24,1925	23,9909	28,5032	28,5843	11	11,367	B2R4R0;P6	B2R4R0;P6	Histone H4
21,729	21,3619	21,3723	21,0151	11	26,805	B2RDE8;P5	B2RDE8;P5	Hepatoma-
23,2667	23,5199	NaN	NaN	11	79,952	H7C0L5;B7	H7C0L5;B7	Inter-alpha
24,0193	23,9749	25,451	25,4183	11	50,151	P68363;B3	P68363;B3	Tubulin alp
24,1893	24,4691	NaN	21,4488	11	36,254	D9IWP9;P0	D9IWP9;P0	Beta-2-glyc
20,4402	20,7698	NaN	NaN	11	84,304	G3V1D3;G3	G3V1D3;G3	Dipeptidyl
NaN	NaN	NaN	NaN	11	28,87	V9HWE3;P0	V9HWE3;P0	Carbonic an
21,8106	21,8351	27,6523	27,5826	11	22,782	V9HW43;P	V9HW43;P	Heat shock
29,8186	29,647	29,2989	29,7029	11	13,242	P06702;B2	P06702;B2	Protein S10
24,0627	24,1865	24,1632	22,9734	11	22,742	V9HWC6;P	V9HWC6;P	Peptidyl-pr
23,7674	23,8591	26,5802	26,6174	11	16,891	P27482	P27482	Calmodulin
24,3802	24,1468	23,6914	23,8414	11	17,031	V9HW35;P	V9HW35;P	Peroxiredo
17,9482	NaN	18,122	NaN	11	11,471	P31151	P31151	Protein S10
21,1914	21,1438	NaN	NaN	11	94,33	P34932;V9	P34932;V9	Heat shock
24,4952	24,3853	24,277	24,1006	11	36,426	V9HWF2;P4	V9HWF2;P4	Malate deh
23,1384	23,3435	26,6168	26,614	11	50,112	Q53G85;Q5	Q53G85;Q5	Elongation
22,7094	22,8236	23,4796	23,0671	11	24,488	Q15907;H3	Q15907;H3	Ras-related
23,5541	24,0127	23,6668	24,1806	11	32,642	Q32Q12;Q6	Q32Q12;Q6	Nucleoside
NaN	21,9007	NaN	NaN	10	39,455	A0A024QZ6	A0A024QZ6	Fructose-bi
22,4373	21,7184	21,5632	21,5063	10	52,602	A0A024R94	A0A024R94	Antithromb
27,5879	27,4564	27,2549	27,0393	10	28,889	A0A1R3UCI	A0A1R3UCI	Kallikrein-1
NaN	NaN	21,5354	21,9515	10	30,57	A0A1R3UCI	A0A1R3UCI	Kallikrein-1
23,2798	24,0927	22,1654	22,31	10	75,872	A0A1W2PN	A0A1W2PNV4	
22,4938	22,0267	23,2938	23,6978	10	45,836	Q5VUU6;B2	Q5VUU6;B2	Myeloid cel
23,9609	23,8644	NaN	NaN	10	40,058	F1C4A7;B2	F1C4A7;B2	Monocyte d
22,8249	23,2591	22,3092	22,1224	10	27,566	V9HWG9;B	V9HWG9;B	Glutathion
23,4628	23,4775	23,0243	23,2443	10	38,498	V9HW69;B	V9HW69;B	Macrophag
21,9319	22,5784	NaN	NaN	10	45,315	B2RC45;Q4	B2RC45;Q4VAX6	
21,9894	22,1095	22,1455	22,2658	10	106,87	V9HWJ0;B4	V9HWJ0;B4	Neutral alp
23,6138	23,6164	23,148	22,998	10	30,767	B4DL49;Q5	B4DL49	
23,4913	23,4243	NaN	21,5054	10	72,119	B7Z539;B7	B7Z539;B7	Inter-alpha
NaN	NaN	26,2589	26,4605	10	52,247	CON__O76(	CON__O76(	Keratin, typ
26,7442	26,5122	26,6943	26,3178	10	11,737	H9ZYJ2;P10	H9ZYJ2;P10	Thioredoxin
NaN	NaN	21,8952	20,9581	10	39,617	O75367;Q5	O75367;Q5	Core histor
21,9391	21,7192	20,2809	NaN	10	45,398	P06727;Q1	P06727;Q1	Apolipopro
30,7624	30,6695	25,8611	26,3389	10	16,445	P09228	P09228	Cystatin-SA
27,894	28,0421	25,2712	24,8614	10	48,206	P20061	P20061	Transcobala
20,4355	21,1962	21,6964	21,5875	10	47,716	P23526;Q1	P23526;Q1	Adenosylho
22,8682	22,7158	24,5697	24,7003	10	15,693	P29373;Q5	P29373;Q5	Cellular ret

23,4387	23,233	21,0989	21,6434	10	37,191	P33241;A8	P33241;A8	Lymphocyt
21,4697	NaN	21,8854	20,9965	10	52,384	V9HWJ1;P4	V9HWJ1;P4	Glutathion
22,0903	22,2643	21,993	NaN	10	68,985	Q13421;H3	Q13421;H3	Mesothelin
25,377	25,4536	21,6134	20,8451	10	53,113	Q86U78;Q5	Q86U78;Q5	Angiotensin
30,7319	30,727	30,0716	30,4424	10	44,786	Q9NPP6;A0	Q9NPP6	
22,5326	22,4646	20,3165	NaN	9	35,427	A0A0S2Z4N	A0A0S2Z4N	Vasodilator
20,6	NaN	21,1786	21,2986	9	282,25	A0A024R88	A0A024R88	Spectrin alp
22,4705	22,7913	22,5964	22,4415	9	21,258	A0A024RAI	A0A024RAI	Cell division
NaN	NaN	NaN	NaN	9	99,978	A8K6T3;A0	A8K6T3;A0	Desmocolli
21,8211	21,8013	NaN	NaN	9	86,677	A0A024RC7	A0A024RC7	Furin
NaN	NaN	21,1336	21,2222	9	83,822	B3GN61;D3	B3GN61;D3	Cadherin-1
22,1497	21,9024	22,7606	22,8342	9	27,887	A0A0K0K1I	A0A0K0K1I	Proteasome
20,5274	20,595	NaN	NaN	9	50,582	P60028;A0	P60028;A0	Rab GDP di
21,8319	21,6135	NaN	NaN	9	46,312	A0A140VKI	A0A140VKI	Pigment ep
26,0077	26,0621	24,4029	24,3365	9	15,257	P69905;A0	P69905;A0	Hemoglobi
27,2727	27,5277	25,8076	25,4914	9	5,0526	A2VCK8;P6	A2VCK8;P6	Thymosin b
NaN	NaN	24,8248	24,4847	9	42,064	A8YXX4;P1	A8YXX4;P1	Glutamine
25,3789	25,3538	24,9437	25,1107	9	16,057	Q5H9A7;Q6	Q5H9A7;Q6	Metallopro
23,0372	22,8873	21,5778	NaN	9	37,106	Q6IAW5;B3	Q6IAW5;B3	Calumenin
22,5354	22,6972	NaN	21,5996	9	50,151	B4DJQ8;P5	B4DJQ8;P5	Dipeptidyl
NaN	NaN	22,6593	22,4138	9	35,076	P63244;E9	P63244;E9	Guanine nu
21,0451	21,2122	NaN	NaN	9	65,408	E9PIT3;P00	E9PIT3;P00	Prothromb
24,652	24,4081	26,8443	26,7509	9	28,518	P08246;B2	P08246;B2	Neutrophil
NaN	NaN	22,2967	21,7927	9	30,375	P16152;E9	P16152;E9	Carbonyl re
NaN	NaN	23,882	24,179	9	21,364	P16403	P16403	Histone H1
22,6288	22,9189	21,9175	21,6384	9	53,899	P17213;B4	P17213;B4	Bactericida
23,6128	23,5818	26,1537	26,3894	9	7,9053	P22532	P22532	Small prolin
20,9423	21,5223	23,2932	22,8355	9	21,892	V9HW12;P	V9HW12;P	Peroxiredo
23,0822	23,1781	24,2881	23,9372	9	29,174	V9HW98;P	V9HW98;P	14-3-3 prot
NaN	NaN	22,8322	22,6866	9	24,029	Q05DB4;Q9	Q05DB4;Q9	Heme-bind
24,0922	24,0032	22,9039	22,9033	9	15,945	Q14019;H3	Q14019;H3	Coactosin-l
NaN	21,3352	22,4808	21,9631	9	48,121	Q15084	Q15084	Protein disu
24,1013	24,0619	21,5772	NaN	9	38,167	Q68CK4	Q68CK4	
22,0721	21,7875	21,3848	21,2099	9	57,578	Q6XQN6;G5	Q6XQN6;G5	Nicotinate
22,9314	22,9851	22,1936	21,0765	9	19,891	V9HWC2;Q	V9HWC2;Q	Protein deg
20,9464	21,3793	20,7506	20,4784	9	35,548	Q9BRF8;B3	Q9BRF8	Serine/thre
22,2588	22,1331	22,4815	22,302	8	19,56	Q5ISS1;A0A	Q5ISS1;A0A	ADP-ribosy
NaN	NaN	21,7918	21,6508	8	35,885	A0A024R17	A0A024R17	Prostagland
NaN	NaN	NaN	NaN	8	81,224	A0A024R2U	A0A024R2U	Acylamino-
22,8016	23,1338	22,3034	22,6604	8	21,768	P61586;A0	P61586;A0	Transformin
NaN	NaN	22,4151	22,3695	8	60,047	B7Z597;B3	B7Z597;B3	60 kDa hea
NaN	NaN	NaN	NaN	8	26,855	A0A024R4J	A0A024R4J	Kallikrein-6
23,8461	22,9425	NaN	NaN	8	47,65	A0A024R6I	A0A024R6I	Alpha-1-ant
21,3158	NaN	22,1181	21,6635	8	23,545	A0A024R7V	A0A024R7V	Ras-related
22,1647	22,2938	20,6638	20,9464	8	42,625	A0A024R96	A0A024R96	Chitinase-3
23,2768	22,816	22,3579	22,6134	8	28,412	I6L957;A0A	I6L957;A0A	Heterogene
21,0116	21,3567	NaN	NaN	8	27,401	Q86SZ7;Q2	Q86SZ7;Q2	Proteasome
23,3081	23,2646	21,3278	NaN	8	24,963	A0A0A8K9I	A0A0A8K9I	Protein FAM
22,0927	22,0674	20,7574	20,763	8	27,547	A0A0K0K1I	A0A0K0K1I	6-phosphog
22,7867	22,7987	NaN	NaN	8	23,489	A0A158RFU	A0A158RFU	Ras-related
23,8153	23,9506	24,2052	23,9802	8	32,933	B4DL57;A8	B4DL57;A8	Transmemb
22,5386	21,9634	21,735	NaN	8	42,16	Q9P1W6;B	Q9P1W6;B	Galactoside

NaN	NaN	NaN	NaN	8	80,733	Q9UM02;B	Q9UM02;B	Prolyl endo
25,5586	25,619	25,8371	26,3095	8	16,837	P0DP24;B4	P0DP24;B4DJ51;H0Y7	
NaN	NaN	23,5987	24,5437	8	43,855	B4DQE1;P1	B4DQE1;P1	Annexin;An
NaN	NaN	26,5271	26,4873	8	18,281	CON__P02	CON__P02754	
NaN	NaN	26,2578	26,29	8	48,105	CON__Q04	CON__Q04	Keratin, typ
23,6244	23,2984	22,8483	22,7783	8	54,305	D9ZGG2;P	D9ZGG2;P	Vitronectin
23,7437	23,801	25,3772	25,195	8	16,93	P60660;F8	P60660;F8	Myosin ligh
22,135	22,0332	22,7997	23,0385	8	20,457	J3QRS3;P1	J3QRS3;P1	Myosin regu
NaN	21,1733	NaN	21,0607	8	56,256	V9HW90;P	V9HW90;P	Glutathion
26,4674	26,9994	23,7632	24,1793	8	23,511	V9HWF6;P	V9HWF6;P	Alpha-1-aci
26,3137	26,0664	26,0271	25,9625	8	14,326	P03973	P03973	Antileukop
23,4261	23,6434	23,3629	24,6283	8	10,044	P07108;B8	P07108;B8	Acyl-CoA-bi
23,5927	23,8207	26,6384	26,3472	8	28,837	P08311	P08311	Cathepsin G
NaN	NaN	25,1853	26,6709	8	64,416	P12035;CC	P12035;CC	Keratin, typ
21,4317	21,0736	22,1482	NaN	8	36,573	V9HWI0;P1	V9HWI0;P1	Alcohol deh
29,2213	29,1828	25,1197	24,7777	8	16,08	P28325	P28325	Cystatin-D
24,2183	24,1984	23,6536	23,7574	8	28,082	V9HWD6;P	V9HWD6;P	14-3-3 prot
22,0218	22,2055	NaN	20,8241	8	69,068	P43652	P43652	Afamin
NaN	NaN	26,9299	27,0805	8	15,075	P47929	P47929	Galectin-7
NaN	NaN	NaN	NaN	8	16,14	P69891;P6	P69891;P6	Hemoglobi
24,1592	24,2827	23,817	23,6799	8	22,693	P80723	P80723	Brain acid s
NaN	NaN	NaN	NaN	8	193,51	Q05707;A8	Q05707;A8	Collagen alp
21,9732	22,2168	22,0008	21,9185	8	42,12	Q53FH2;Q1	Q53FH2;Q1	Chitinase-3
20,912	21,4127	22,2524	21,9782	8	96,022	Q8WUM4;	Q8WUM4;	Programme
19,9184	NaN	20,0502	NaN	8	30,608	V9HW91;Q	V9HW91;Q	Omega-ami
22,1911	22,1558	25,422	25,4807	8	15,892	Q9NZT1;Q5	Q9NZT1;Q5	Calmodulin
23,1059	23,2232	21,4417	21,4689	8	21,627	Q9ULZ3;B2	Q9ULZ3;B2	Apoptosis-a
NaN	21,5448	NaN	NaN	7	26,697	A0A024QZ	A0A024QZ	EF-hand do
NaN	NaN	21,2647	NaN	7	28,218	A0A024R1	A0A024R1	14-3-3 prot
24,5288	24,5665	23,8088	23,9126	7	21,429	P15153;A0	P15153;A0	Ras-related
NaN	NaN	22,9028	22,946	7	48,51	B4DUQ1;A	B4DUQ1;A	Heterogene
24,6859	24,5598	25,4261	25,4527	7	16,142	A0A024R5	A0A024R5	Interleukin
21,1198	NaN	20,9754	21,2295	7	59,296	A0A024R7	A0A024R7	Glucosidase
21,777	21,3838	21,856	21,7013	7	20,825	A0A024RB	A0A024RB	Ras-related
NaN	NaN	23,2117	22,8594	7	75,276	A0A0S2Z37	A0A0S2Z37	Annexin;An
22,9098	22,6274	NaN	21,341	7	27,465	A0A1R3UD	A0A1R3UD	Kallikrein-1
NaN	NaN	NaN	20,8232	7	37,482	A6NC48;Q1	A6NC48;Q1	ADP-ribosy
21,1296	NaN	NaN	20,3907	7	35,964	Q92820;A8	Q92820;A8	Gamma-glu
24,455	24,5401	24,0433	24,7491	7	54,739	P55058;B3	P55058;B3	Phospholip
NaN	NaN	21,8843	NaN	7	42,309	B4DEA3;P5	B4DEA3;P5	UV excision
NaN	NaN	NaN	21,837	7	45,597	B4DPJ2;Q5	B4DPJ2;Q5	Annexin;An
22,6519	22,8247	23,1385	23,4461	7	26,224	B5MDF5;P	B5MDF5;P	GTP-bindin
25,243	25,3072	23,2846	23,8797	7	39,411	C9JV77;B7	C9JV77;B7	Alpha-2-HS
NaN	NaN	NaN	NaN	7	164,34	CON__ENSE	CON__ENSEMBL:ENSBT	
NaN	NaN	NaN	NaN	7	30,276	CON__P15	CON__P15497	
NaN	NaN	NaN	NaN	7	75,829	CON__Q29	CON__Q29443;CON__	
23,614	23,8601	24,0816	23,7889	7	21,058	F8WCF6;P	F8WCF6;P	Actin-relate
21,2668	21,8088	20,8245	20,6943	7	19,997	H0YN26;Q	H0YN26;Q	Acidic leuci
NaN	NaN	NaN	NaN	7	55,023	O60701;Q	O60701	UDP-glucos
22,5637	22,8364	NaN	NaN	7	12,774	V9HW48;O	V9HW48;O	SH3 domain
NaN	NaN	NaN	NaN	7	152,93	O75976;B7	O75976	Carboxypep
NaN	NaN	21,3671	NaN	7	54,861	V9HW83;V	V9HW83;V	Retinal deh

NaN	NaN	NaN	NaN	7	29,246	V9HW21;P	V9HW21;P	Carbonic ar
24,3905	24,3045	24,3895	24,4257	7	57,019	Q6N030;Q5	Q6N030;Q5	Ig gamma-3
NaN	NaN	21,9726	21,8221	7	46,596	P05120;B2	P05120;B2	Plasminoge
NaN	NaN	22,5605	22,6569	7	35,293	Q6NVC0;P(	Q6NVC0;P(	ADP/ATP tr
22,4534	22,5041	22,1646	22,0832	7	43,101	P06732;B2	P06732;B2	Creatine kir
NaN	NaN	22,915	22,8064	7	35,936	V9HWE0;P(	V9HWE0;P(	Annexin;Ar
24,2943	24,8711	25,0172	24,9739	7	48,462	P0DOX2	P0DOX2	
NaN	21,4689	NaN	NaN	7	56,224	P0DOX3;Q8	P0DOX3;Q8NF20	
NaN	NaN	20,6541	20,5381	7	96,695	P11216;B4	P11216;B4	Glycogen p
21,4198	21,803	21,5042	22,1224	7	85,018	P17858;B3	P17858;B3	ATP-depend
22,3898	22,5326	23,0452	23,1868	7	26,885	P20160;Q8	P20160;Q8	Azurocidin
23,0486	22,5988	24,0144	22,1838	7	24,033	P26583;Q5	P26583;Q5	High mobil
20,9491	21,5795	23,5173	23,0374	7	50,118	Q53YD7;P2	Q53YD7;P2	Elongation
NaN	NaN	21,625	NaN	7	67,567	P27824;D6	P27824	Calnexin
NaN	20,0915	NaN	NaN	7	32,668	P46926;D6	P46926;D6	Glucosamir
NaN	NaN	NaN	21,0185	7	64,149	P48163;A8	P48163;A8	NADP-depe
23,9762	23,8077	24,2044	24,4716	7	23,207	V9HWE8;P5	V9HWE8;P5	Rho GDP-di
NaN	NaN	NaN	NaN	7	585,56	P98088;T1	P98088;T1	Mucin-5AC
25,8887	26,2977	24,6118	24,8117	7	12,993	Q14508;A8	Q14508	WAP four-c
25,198	25,5296	25,0941	24,3226	7	51,098	Q6MZU6	Q6MZU6	
20,6575	NaN	21,4678	21,5211	7	63,254	Q6P4A8;F5	Q6P4A8	Phospholip
NaN	NaN	NaN	20,5806	7	75,952	Q86UX7;F5	Q86UX7;F5	Fermitin fa
27,7006	27,6572	23,0712	23,1012	7	39,158	Q8TAX7;D6	Q8TAX7;D6	Mucin-7
NaN	22,1957	23,5133	23,8977	7	22,171	Q9H0U4;Q	Q9H0U4;Q	Ras-related
NaN	NaN	25,6954	25,7084	7	11,662	Q9HCY8	Q9HCY8	Protein S10
NaN	NaN	23,6506	22,5434	6	65,816	Q53G41;A0	Q53G41;A0	Tripartite m
23,1436	23,4042	21,3251	21,4677	6	24,281	A0A172Q3	A0A172Q3	Folate recep
NaN	NaN	20,9355	21,5173	6	18,985	A0A024R7(	A0A024R7(	Tubulin pol
NaN	21,1225	NaN	NaN	6	59,761	A0A0S2Z45	A0A0S2Z45	Neutrophil
20,4802	NaN	21,6806	21,6928	6	38,746	P09651;F8	P09651;F8	Heterogene
NaN	NaN	22,089	22,4996	6	51,712	O75390;A0	O75390;A0	Citrate synt
19,2774	NaN	NaN	NaN	6	70,859	B4DV63;A0	B4DV63;A0	Aminopept
20,9135	20,7299	NaN	NaN	6	108,34	A0A087X0S	A0A087X0S	Collagen al
NaN	NaN	NaN	NaN	6	131,13	X6R433;A0	X6R433;A0	Protein-tyr
NaN	NaN	NaN	NaN	6	82,688	A0A0F7NG	A0A0F7NG	Leucine-ric
20,8974	20,7326	NaN	19,7666	6	34,348	A0A0G2JR5	A0A0G2JR5	Neutrophil
20,4299	20,5406	22,6102	22,6104	6	65,335	A0A0S2Z3S	A0A0S2Z3S	Cytochrom
21,036	21,037	NaN	NaN	6	47,094	Q9BVJ8;H3	Q9BVJ8;H3	Beta-hexos
NaN	NaN	21,221	NaN	6	273,42	P49327;A0	P49327;A0	Fatty acid s
NaN	NaN	21,0321	20,9514	6	26,411	A0A109NG	A0A109NG	Proteasom
20,4355	20,7106	NaN	NaN	6	82,265	A0A140VJE	A0A140VJE	Alpha-N-ac
NaN	21,7855	20,8255	NaN	6	24,588	Q6LET3;A0	Q6LET3;A0	Hypoxanth
NaN	NaN	20,814	20,8954	6	37,512	P62136;A0	P62136;A0	Serine/thre
NaN	NaN	20,9985	20,6525	6	26,491	Q53FT8;A0	Q53FT8;A0	Proteasom
NaN	NaN	NaN	NaN	6	49,88	P14136;A0	P14136;A0	Glial fibrilla
NaN	NaN	NaN	NaN	6	16,055	A0N071;P0	A0N071;P0	Hemoglobi
20,7591	20,4032	NaN	NaN	6	32,967	Q53GE2;Q5	Q53GE2;Q5	F-actin-cap
22,923	22,8417	22,2593	NaN	6	41,85	A8MVU1;P	A8MVU1;P	Putative ne
22,8622	22,6592	NaN	NaN	6	29,114	D6RHI9;A6	D6RHI9;A6	Ribonuclea
NaN	NaN	NaN	NaN	6	62,639	V9HW72;A	V9HW72;A	Stress-indu
21,4203	21,5157	21,5535	21,7811	6	25,982	P58499;A8	P58499;A8	Protein FAM
NaN	NaN	NaN	NaN	6	42,981	P10644;B2	P10644;B2	cAMP-depe

NaN	NaN	NaN	NaN	6	49,616	B4DQ92;E7	B4DQ92;E7	Hematopoi
NaN	NaN	22,59	22,7267	6	26,221	B4DVY2;V9	B4DVY2;V9	Tropomyos
20,4359	20,53	NaN	NaN	6	129,83	B4DZ36;O7	B4DZ36;O7	Attractin
23,7161	23,904	25,0528	25,406	6	9,9355	B7ZLF8	B7ZLF8	
NaN	NaN	20,8133	20,3598	6	14,186	CON__P007	CON__P00711	
21,8418	NaN	NaN	NaN	6	61,212	Q53HP2;D3	Q53HP2;D3	Tripeptidyl
27,0096	26,9203	24,302	24,7722	6	23,625	D6CHE9;U3	D6CHE9;U3	Myeloblast
21,3045	21,7075	NaN	NaN	6	10,08	E5RIW3;Q6	E5RIW3;Q6	Tubulin-spe
20,5202	NaN	NaN	NaN	6	24,526	H0YMZ1;H0	H0YMZ1;H0	Proteasome
NaN	NaN	NaN	NaN	6	35,078	O00602;Q9	O00602;Q9	Ficolin-1
22,2929	22,5496	19,8505	19,8539	6	22,974	Q5VY30;P0	Q5VY30;P0	Retinol-bin
25,67	25,5064	27,0639	27,1827	6	11,139	Q76LA1;P0	Q76LA1;P0	Cystatin-B
22,6441	22,9557	NaN	NaN	6	21,252	X5D932;X5	X5D932;X5	Glutathion
NaN	NaN	20,4071	NaN	6	35,709	P09758;C5	P09758;C5	Tumor-asso
NaN	NaN	NaN	21,165	6	48,275	P14324;B3	P14324;B3	Farnesyl py
NaN	NaN	NaN	NaN	6	44,566	P15309;A0	P15309;A0	Prostatic ac
NaN	NaN	NaN	NaN	6	12,269	P19957	P19957	Elafin
NaN	NaN	NaN	NaN	6	57,709	P19961	P19961	Alpha-amyl
21,9147	21,6022	23,595	23,0737	6	26,663	Q5ISS9;P27	Q5ISS9;P27	14-3-3 prot
NaN	21,0287	NaN	NaN	6	35,554	Q5TZP7;P2	Q5TZP7;P2	DNA-(apuri
25,1216	24,8551	25,9268	25,8858	6	11,74	P31949;V9	P31949;V9	Protein S10
NaN	NaN	NaN	20,5583	6	22,949	P49720;A0	P49720;A0	Proteasome
25,8686	26,3534	26,1705	26,2316	6	42,051	P68133;P6	P68133;P6	Actin, alpha
24,6876	24,6832	25,082	24,7563	6	10,575	P80511	P80511	Protein S10
21,0153	NaN	NaN	NaN	6	39,548	Q6IBS0;D6I	Q6IBS0;D6I	Twinfilin-2
NaN	NaN	23,5943	23,7175	6	30,847	Q6ZVX7	Q6ZVX7	F-box only p
NaN	NaN	NaN	NaN	6	46,337	Q86T26	Q86T26	Transmemb
22,0725	23,1059	NaN	23,2064	6	8,26	Q9BPY8	Q9BPY8	Homeodom
23,4234	23,2554	24,9502	25,1656	6	26,712	Q9NP55	Q9NP55	BPI fold-co
NaN	NaN	22,6685	22,6186	6	17,684	Q9UHA7	Q9UHA7	Interleukin
NaN	NaN	22,6314	22,8449	5	30,348	A0A0A0MR	A0A0A0MR	Voltage-dep
22,86	22,5913	27,2017	26,9518	5	13,989	Q99879;U3	Q99879;U3	Histone H2
22,7205	22,4028	23,6748	23,7161	5	13,897	Q86TY5;Q6	Q86TY5;Q6	Galectin;Ga
23,1248	23,3441	23,0522	23,0783	5	13,078	G3V2V8;J3	G3V2V8;J3	Epididymal
NaN	NaN	NaN	NaN	5	29,168	B4DQ93;A0	B4DQ93;A0	Syntenin-1
NaN	NaN	20,5958	NaN	5	30,188	A0A024R83	A0A024R83	Inositol mo
NaN	21,6254	NaN	NaN	5	25,84	Q53GF5;A0	Q53GF5;A0	Proteasome
19,8978	21,3587	NaN	NaN	5	38,258	Q5URX0;A0	Q5URX0;A0	Beta-hexos
21,1658	21,5735	NaN	NaN	5	11,776	P35754;A0	P35754;A0	Glutaredox
20,5057	NaN	NaN	NaN	5	107,23	Q9NZ08;A0	Q9NZ08;A0	Endoplasm
21,4225	NaN	24,8784	25,3535	5	13,906	A3KPC7;A0	A3KPC7;A0	Histone H2
NaN	NaN	21,3337	21,2187	5	23,707	P61020;A0	P61020;A0	Ras-related
NaN	19,9343	NaN	NaN	5	38,737	A0A024RBI	A0A024RBI	Lamina-ass
NaN	NaN	23,7756	24,2413	5	11,665	P05387;A0	P05387;A0	60S acidic r
22,4258	21,0072	21,7073	21,5922	5	18,311	Q5T7C4;B7	Q5T7C4;B7	High mobil
NaN	21,4909	NaN	NaN	5	15,887	E9KL36;A6	E9KL36;A6	Transthyre
NaN	NaN	21,8125	21,4027	5	31,121	P29692;E9	P29692;E9	Elongation
21,2106	20,7028	NaN	NaN	5	20,683	B7Z972;A0	B7Z972;A0	Protein-L-is
NaN	NaN	NaN	NaN	5	126,49	A0A0J9YX7	A0A0J9YX7	Maltase-glu
NaN	NaN	NaN	NaN	5	34,52	B7Z3I9;A0A	B7Z3I9;A0A	Delta-amin
22,2678	22,3589	22,1306	22,54	5	13,941	A0A140VJY	A0A140VJY	Thioredoxi
NaN	NaN	21,5633	21,5321	5	28,415	Q6IB71;A0A	Q6IB71;A0A	Proteasome

NaN	NaN	NaN	NaN	5	56,5	A0A140VK(	A0A140VK(	V-type prot
22,2594	21,9172	22,8082	22,5365	5	43,831	A0A286YFJ	A0A286YFJ	Ig gamma-4
25,9204	25,9007	26,7125	26,5725	5	25,164	A2KBC4;A2	A2KBC4;A2	KBC2;A2KB(
25,809	26,3337	22,4356	22,6769	5	10,392	A2MYE1;A2	A2MYE1;A2	Ig kappa ch
26,4246	26,0626	25,1655	26,0618	5	24,654	A2NUT2;Q6	A2NUT2;Q6	PJG0;Q6IP(
24,2905	24,2096	24,6509	NaN	5	35,97	B2RBR3;A8	B2RBR3;A8	Ly6/PLAUR
NaN	NaN	22,317	22,864	5	46,153	P60842;A8	P60842;A8	Eukaryotic
NaN	NaN	20,2499	20,3659	5	34,486	A8K8J9;B3I	A8K8J9;B3I	Dynactin su
20,4064	20,515	21,0861	21,6695	5	136,32	A8K8U1;Q8	A8K8U1;Q8	Cullin-asso(
21,2847	NaN	20,9844	20,7279	5	25,148	Q6ICN0;B3	Q6ICN0;B3	Growth fac
21,6474	NaN	NaN	NaN	5	45,094	B2R9F2;P0	B2R9F2;P0	Corticoster
NaN	NaN	NaN	NaN	5	52,276	B3KTA3;Q1	B3KTA3;Q1	Fascin
NaN	NaN	23,19	22,9093	5	62,615	B4DE59;C9	B4DE59	
NaN	NaN	NaN	NaN	5	68,877	B4DL56;Q2	B4DL56;Q2	Polypeptid
NaN	NaN	NaN	NaN	5	75,959	E9PGM4;B4	E9PGM4;B4	1,4-alpha-g
NaN	NaN	NaN	NaN	5	52,878	Q96KP4;B4	Q96KP4;B4	Cytosolic n
NaN	19,5332	21,0852	20,994	5	16,712	H7C2Z6;H7	H7C2Z6;H7	Grancalcin
NaN	NaN	20,9069	21,0599	5	430,4	I0B0K8;CO	I0B0K8;CO	Filaggrin
NaN	NaN	23,012	22,8352	5	64,842	CON__Q6IS	CON__Q6IS	Keratin, typ
NaN	21,1573	NaN	NaN	5	25,572	E9PGT1;Q1	E9PGT1;Q1	Translin
NaN	NaN	23,477	23,4968	5	20,03	E9PR44;V9	E9PR44;V9	Alpha-cryst
NaN	NaN	23,2584	23,3378	5	13,069	F2Z2S8;H0	F2Z2S8;H0	40S riboso
21,6363	22,0683	NaN	21,0265	5	35,102	V9HWC3;O	V9HWC3;O	Pyridoxal k
NaN	NaN	NaN	NaN	5	188,3	P01031;Q5	P01031;Q5	Compleme
21,0867	20,6824	21,594	21,6736	5	15,603	Q53R15;P0	Q53R15;P0	Myosin ligh
20,1101	20,3259	21,326	21,6274	5	65,953	Q59FC6;Q5	Q59FC6;Q5	Endoplasm
NaN	NaN	24,2863	23,8804	5	22,58	P16401;Q1	P16401	Histone H1
NaN	20,3085	NaN	19,8781	5	29,555	P25786;V9	P25786;V9	Proteasom
24,8677	25,0453	24,4037	24,8292	5	10,4	P25815	P25815	Protein S1(
NaN	NaN	20,3781	20,378	5	31,554	P29966;Q6	P29966;Q6	Myristoylat
19,87	19,6243	20,8195	20,5844	5	22,119	V9HWI1;P3	V9HWI1;P3	Flavin redu
22,378	22,2913	NaN	NaN	5	16,185	P32320;Q7	P32320	Cytidine de
NaN	NaN	21,7606	NaN	5	68,303	P38606;C9	P38606;C9	V-type prot
NaN	NaN	NaN	19,9495	5	13,802	P49773;D6	P49773	Histidine tr
NaN	NaN	21,1733	21,251	5	54,106	Q7Z759;Q5	Q7Z759;Q5	T-complex
NaN	NaN	NaN	NaN	5	26,599	P56537;B7	P56537	Eukaryotic
NaN	NaN	20,7719	NaN	5	17,138	V9HW41;P	V9HW41;P	Ubiquitin-(
NaN	NaN	NaN	NaN	5	50,435	Q07960;H(	Q07960;H(	Rho GTPase
NaN	NaN	NaN	NaN	5	113,43	Q9NS13;Q(	Q9NS13;Q(	Apolipopro
NaN	NaN	21,5167	20,7909	5	531,78	Q15149;D3	Q15149	Plectin
NaN	NaN	NaN	NaN	5	32,66	Q15181;V9	Q15181;V9	Inorganic p
NaN	NaN	20,3079	19,9069	5	227,87	Q7Z406;M(	Q7Z406;M(	Myosin-14
NaN	NaN	24,6143	24,8617	5	11,801	Q96FQ6	Q96FQ6	Protein S1(
21,7942	NaN	21,7362	21,8913	5	33,868	Q9UBR2;Q5	Q9UBR2;Q5	Cathepsin Z
NaN	NaN	20,5893	20,1216	4	21,537	Q5TD07;B3	Q5TD07;B3	Ribosyldihy
NaN	21,445	23,2069	23,3325	4	37,377	P62873;A0	P62873;A0	Guanine nu
22,4998	21,7907	NaN	21,7248	4	17,789	A8K4W8;A(	A8K4W8;A(	Ubiquitin-(
NaN	NaN	NaN	NaN	4	119,77	A0A024R1Y	A0A024R1Y	ATP-citrate
NaN	20,9293	20,4171	NaN	4	39,869	A0A024R49	A0A024R49	Calcium-bi
NaN	NaN	22,5998	22,208	4	22,591	A0A024R4I	A0A024R4I	40S riboso
NaN	NaN	NaN	NaN	4	19,376	E9PR22;A0	E9PR22;A0	Kallikrein-1
NaN	NaN	19,8427	20,4764	4	17,484	W8GIV4;W	W8GIV4;W	Kallikrein-1

NaN	NaN	20,8654	NaN	4	12,623	C9JTH1;A0.	C9JTH1;A0.	Interleukin
NaN	NaN	NaN	NaN	4	53,768	B4DMV9;B4	B4DMV9;B4	EH domain-
21,4476	21,1971	NaN	NaN	4	55,799	A0A024R5I	A0A024R5I	Lysosomal
NaN	20,1272	NaN	NaN	4	53,165	A0A024R6I	A0A024R6I	Tryptophar
21,0064	NaN	NaN	NaN	4	14,478	P61970;A0	P61970;A0	Nuclear tra
NaN	NaN	NaN	NaN	4	24,267	A0A024R7(	A0A024R7(	Ras-related
NaN	NaN	21,6198	21,2254	4	23,897	P61106;A0	P61106;A0	Ras-related
NaN	20,3739	21,314	21,4717	4	25,964	B4E2V5;B4	B4E2V5;B4	Erythrocyte
NaN	NaN	21,4107	22,4993	4	60,13	A0A024R9S	A0A024R9S	Copine-3
NaN	NaN	21,8616	22,289	4	21,659	F5H0U5;A0	F5H0U5;A0	Glycolipid
NaN	NaN	21,4603	NaN	4	32,741	Q8TBK5;Q8	Q8TBK5;Q8	60S ribosor
NaN	NaN	NaN	19,6902	4	31,601	Q1EPW1;A6H580;A0A0B7MGC3;A(		
22,752	24,2347	22,7069	22,2764	4	13,079	A0A0C4DH	A0A0C4DH68;A0A075I	
NaN	NaN	21,7273	NaN	4	44,325	Q1T7A3;A0	Q1T7A3;A0	CD177 anti
NaN	NaN	NaN	NaN	4	27,638	P41439;A0	P41439;A0	Folate rece
19,7139	19,9847	NaN	NaN	4	25,402	A0A087X1J	A0A087X1J	Glutathion
21,6131	21,2738	NaN	NaN	4	19,757	A0A087X27	A0A087X27	Calponin;C
25,82	25,9225	25,8113	25,7722	4	8,641	Q07654;A0	Q07654;A0	Trefoil fact
NaN	NaN	21,1331	21,3177	4	19,595	A0A0P1J1F	A0A0P1J1F	Translation
23,1241	NaN	NaN	22,7829	4	11,359	A0A0F7T73	A0A0F7T73	Ig heavy cha
NaN	NaN	NaN	NaN	4	20,96	A2ACR1;A0	A2ACR1;A0	Proteasom
NaN	NaN	NaN	NaN	4	26,851	J9ZVQ3;A0.	J9ZVQ3;A0.	Apolipoprc
NaN	NaN	NaN	NaN	4	9,9937	P11684;A0	P11684;A0	Uteroglobi
21,4819	21,4322	NaN	NaN	4	44,512	A0A0S2Z5K	A0A0S2Z5K	Nucleotide
24,4533	24,6434	24,5416	25,0305	4	13,525	A0A0X9UW	A0A0X9UWK7	
NaN	NaN	NaN	NaN	4	30,188	A0A140CZL	A0A140CZL	S-methyl-5-
20,6123	20,0811	NaN	21,0774	4	33,428	P53004;A0	P53004;A0	Biliverdin r
NaN	NaN	NaN	NaN	4	35,892	A0A140VK(	A0A140VK(	GDP-L-fuco
NaN	NaN	NaN	NaN	4	53,91	A0A1B0GTJ	A0A1B0GTJ	Alpha-amir
NaN	NaN	NaN	NaN	4	22,837	A0A1B0GV(	A0A1B0GV(	Acid ceram
NaN	NaN	NaN	NaN	4	32,69	A0A1W2PC	A0A1W2PC	Low affinity
20,0662	20,5582	20,947	20,9419	4	20,98	H3BN55;A2	H3BN55;A2	Ras-related
NaN	NaN	NaN	NaN	4	19,408	A2VCR0;B4	A2VCR0;B4	Microtubul
NaN	NaN	NaN	NaN	4	25,895	A6NFX8;Q9	A6NFX8;Q9	ADP-sugar
NaN	NaN	NaN	NaN	4	100,94	A8K6D3;A7	A8K6D3;A7	Beta-mann
21,7324	21,9877	NaN	NaN	4	57,039	B4E370;A8	B4E370;A8	Delta and N
NaN	NaN	20,7814	NaN	4	52,33	B7Z9L0;B7	B7Z9L0;B7	T-complex
20,2476	20,123	NaN	NaN	4	65,059	G3XAM2;A	G3XAM2;A	Complemer
NaN	NaN	NaN	NaN	4	42,135	Q59GW6;A	Q59GW6;A	Acetyl-CoA
NaN	NaN	NaN	NaN	4	23,714	A8K646;Q9	A8K646;Q9	Osteoclast-
NaN	NaN	NaN	NaN	4	107,46	B4E0K9;Q5	B4E0K9;Q5	Alpha-mani
21,4177	20,6387	NaN	NaN	4	35,314	A8KAM5;Q	A8KAM5;Q	Secreted fri
19,8834	NaN	NaN	NaN	4	124,78	B0I1T1;Q4I	B0I1T1;Q4I	Unconvent
23,4765	23,0932	22,793	22,8767	4	20,564	Q2LE71;B2	Q2LE71;B2	Actin-relate
21,7275	21,5893	NaN	NaN	4	21,51	G5E9W8;B	G5E9W8;B	Glycogenin
NaN	NaN	21,4607	21,8619	4	38,898	B2R6A3;O1	B2R6A3;O1	Na(+)/H(+) e
23,2447	23,3352	NaN	NaN	4	59,578	P04196;B2	P04196;B2	Histidine-ri
NaN	NaN	NaN	NaN	4	51,804	Q02790;B2	Q02790;B2	Peptidyl-pr
20,337	20,5039	22,2265	20,8984	4	97,183	B2RBR9;Q1	B2RBR9;Q1	Importin su
NaN	NaN	NaN	NaN	4	70,36	B4DKZ9;B3	B4DKZ9;B3	Threonine-
NaN	NaN	23,0796	23,155	4	9,3195	B3EWG6;B	B3EWG6;B	Protein FAI
23,1486	22,9744	22,8383	22,8301	4	143,66	B3KVV6	B3KVV6	

NaN	NaN	21,3586	NaN	4	34,459	B4DE02;V9	B4DE02;V9	Annexin;Ar
NaN	NaN	21,011	NaN	4	60,405	B4E1D8;B4	B4E1D8;B4	C4b-bindin
NaN	NaN	NaN	NaN	4	47,362	B7Z1V7;B7	B7Z1V7;B7	Stress-70 p
NaN	NaN	19,4848	NaN	4	44,812	F8VQ14;B7	F8VQ14;B7	T-complex
NaN	NaN	23,2024	NaN	4	22,975	CON__P02(	CON__P02662	
NaN	NaN	NaN	23,6433	4	49,167	CON__P19(	CON__P19(	Keratin, typ
NaN	NaN	NaN	NaN	4	65,056	CON__Q1R	CON__Q1RMK2	
NaN	NaN	23,0692	23,3953	4	50,525	CON__Q6K	CON__Q6K	Keratin, typ
NaN	NaN	NaN	NaN	4	53,728	CON__REFS	CON__REFSEQ:XP_9866	
23,4432	23,4655	21,7016	21,9487	4	9,3804	Q5T123;Q8	Q5T123;Q8	SH3 domai
21,5842	21,753	NaN	NaN	4	37,288	Q5UGI6;H9	Q5UGI6;H9	Plasma pro
22,3461	22,4602	22,8301	23,0038	4	11,985	E9PNW4;Q	E9PNW4;Q	CD59 glyco
NaN	NaN	NaN	NaN	4	15,397	F6RFD5;V9	F6RFD5;V9	Destrin
25,0166	24,8158	24,0803	24,2047	4	8,4984	H0YLF3	H0YLF3	
NaN	NaN	NaN	NaN	4	79,317	H3BM42;Q	H3BM42;Q	Golgi appar
NaN	NaN	NaN	NaN	4	16,323	H3BQF1;P0	H3BQF1;P0	Adenine ph
23,0082	22,2494	21,4499	21,5663	4	16,32	O15511;B1	O15511;B1	Actin-relate
21,1064	NaN	NaN	NaN	4	38,087	O43866	O43866	CD5 antige
NaN	NaN	NaN	NaN	4	10,884	O75556	O75556	Mammaglo
24,2614	24,2311	NaN	20,7262	4	21,731	O75594	O75594	Peptidogly
NaN	NaN	NaN	NaN	4	26,648	O95833;Q6	O95833;Q6	Chloride in
21,972	NaN	NaN	NaN	4	15,936	V9HWC9;P	V9HWC9;P	Superoxide
NaN	NaN	NaN	NaN	4	133,99	P01133	P01133	Pro-epider
NaN	19,7392	NaN	NaN	4	39,724	Q6IRT1;Q6	Q6IRT1;Q6	S-(hydroxy
NaN	NaN	20,5023	NaN	4	47,036	P12532;B4	P12532;B4	Creatine ki
23,9329	23,6544	NaN	NaN	4	78,457	P14780	P14780	Matrix met
23,6396	24,0103	NaN	NaN	4	23,602	P19652	P19652	Alpha-1-aci
NaN	NaN	21,0712	21,3632	4	66,408	P20700;B4	P20700;B4	Lamin-B1
NaN	NaN	23,8921	23,9683	4	59,75	V9HW26;P	V9HW26;P	ATP syntha
20,6492	20,7495	20,1864	20,2995	4	39,731	P27169;B4	P27169;B4	Serum para
NaN	NaN	NaN	20,6106	4	29,696	Q6FHU0;X5	Q6FHU0;X5	Proteasom
21,9549	21,7366	24,8617	24,6883	4	11,117	P29034;R4	P29034;R4	Protein S10
NaN	NaN	22,5129	22,1417	4	17,818	P30050;Q5	P30050;Q5	60S ribosor
22,0269	21,2196	21,5629	21,3721	4	16,84	Q53XB4;P3	Q53XB4;P3	Ribonuclea
NaN	NaN	NaN	NaN	4	7,9653	P35326	P35326	Small proli
NaN	NaN	NaN	NaN	4	11,972	Q6I9S7;P4	Q6I9S7;P4	C-X-C moti
NaN	NaN	NaN	NaN	4	33,269	P49247;Q5	P49247;Q5	Ribose-5-p
NaN	NaN	NaN	NaN	4	12,895	P58546;Q6	P58546;Q6	Myotrophi
27,6714	27,6799	27,6836	26,4437	4	10,245	Q6EZE9;P5	Q6EZE9;P5	Neutrophil
21,6341	22,0633	NaN	NaN	4	11,424	Q5W0X3;Q	Q5W0X3;Q	Peptidyl-pr
NaN	20,6407	24,4815	24,5687	4	49,924	P68366;C9	P68366	Tubulin alp
NaN	NaN	22,9666	22,5125	4	11,284	P81605	P81605	Dermcidin;
NaN	NaN	20,9818	NaN	4	93,834	Q9HB00;Q	Q9HB00;Q	Desmocolli
20,9213	21,2288	22,0862	22,147	4	37,497	Q53SS8;Q1	Q53SS8;Q1	Poly(rC)-bi
NaN	NaN	NaN	NaN	4	59,328	Q6IBT3;Q5	Q6IBT3;Q5	T-complex
23,3489	23,6275	NaN	NaN	4	24,794	Q5FWF9;P	Q5FWF9	
NaN	NaN	NaN	NaN	4	44,648	Q5K634	Q5K634	
NaN	NaN	20,7181	NaN	4	64,135	Q5T749	Q5T749	Keratinocy
22,6624	22,7872	21,6843	21,6064	4	51,082	Q6GMX6;P	Q6GMX6;P	Ig gamma-1
NaN	NaN	NaN	NaN	4	22,72	Q6MZM9	Q6MZM9	Proline-ric
27,9496	28,1932	NaN	NaN	4	56,423	Q6N092	Q6N092	
NaN	NaN	21,8359	21,92	4	68,064	Q8WVV4	Q8WVV4	Protein PO

21,721	NaN	NaN	NaN	4	11,594	Q96SB0	Q96SB0	
22,3659	22,0287	21,9757	21,9776	4	11,419	Q9HD89	Q9HD89	Resistin
NaN	NaN	NaN	NaN	4	18,721	Q9NZH8	Q9NZH8	Interleukin
NaN	NaN	NaN	20,2315	4	48,207	Q9UJU6;B4	Q9UJU6;B4	Drebrin-lik
NaN	NaN	22,0363	21,6508	4	87,081	Q9Y446;E9	Q9Y446;E9	Plakophilin
NaN	NaN	NaN	NaN	3	15,222	H3BN54;A0	H3BN54;A0	Myosin reg
NaN	20,3384	NaN	NaN	3	63,173	P02748;A0	P02748;A0	Compleme
NaN	NaN	NaN	NaN	3	57,229	T2HVB7;A0	T2HVB7;A0	Carcinoem
NaN	NaN	NaN	22,0841	3	29,163	M0R0Y2;A0	M0R0Y2;A0	Alpha-solu
20,9471	NaN	NaN	NaN	3	21,932	A0A024R2	A0A024R2	Myosin ligh
NaN	NaN	NaN	NaN	3	42,621	A0A024R3	A0A024R3	Mannose-1
NaN	NaN	NaN	NaN	3	24,763	A0A024R3	A0A024R3	Elongation
NaN	NaN	NaN	NaN	3	58,554	B3KM80;Q	B3KM80;Q	Nucleolin
NaN	NaN	NaN	NaN	3	30,892	A0A024R4	A0A024R4	Uncharacte
NaN	NaN	NaN	NaN	3	79,398	B4DGN8;B	B4DGN8;B	Procollage
NaN	NaN	NaN	NaN	3	82,682	A0A024R8	A0A024R8	Niban-like
NaN	NaN	NaN	NaN	3	105,34	A0A024R8	A0A024R8	Lysosomal
NaN	NaN	19,7745	NaN	3	29,466	K7EN15;A0	K7EN15;A0	Soluble cal
NaN	NaN	21,3015	21,4296	3	12,656	E5RI99;A0	E5RI99;A0	60S ribosor
NaN	NaN	NaN	NaN	3	24,347	Q9UL25;A0	Q9UL25;A0	Ras-related
NaN	NaN	NaN	NaN	3	34,608	H0YFA9;B4	H0YFA9;B4	N-acetylglu
NaN	NaN	NaN	NaN	3	53,28	Q53G58;B3	Q53G58;B3	Coronin;Co
NaN	NaN	NaN	NaN	3	70,905	Q16881;B2	Q16881;B2	Thioredoxi
NaN	NaN	21,6917	21,9931	3	34,273	P05388;F8	P05388;F8	60S acidic
NaN	NaN	NaN	NaN	3	48,991	Q13838;B4	Q13838;B4	Spliceosom
NaN	NaN	NaN	NaN	3	41,293	A0A024RD	A0A024RD	Mitogen-ac
NaN	20,6121	NaN	NaN	3	12,553	D6RF44;H0	D6RF44;H0	Heterogene
NaN	NaN	NaN	NaN	3	274,41	K9MS24;B2	K9MS24;B2	Spectrin be
NaN	NaN	NaN	NaN	3	16,89	A0A087WY	A0A087WY	Proline-ricl
NaN	NaN	NaN	NaN	3	43,412	H0YAL9;A8	H0YAL9;A8	Transmemb
NaN	22,8153	22,8988	22,2624	3	13,056	A0A0A0MS	A0A0A0MS15;Q0ZCH6	
21,9343	22,7475	NaN	NaN	3	11,676	A0A0F7TAG	A0A0F7TAG7;A0A0B4J	
NaN	NaN	NaN	NaN	3	54,227	Q5SSG8;A0	Q5SSG8;A0	Mucin-21
NaN	20,763	21,0865	21,393	3	12,748	A0A0G2JR	A0A0G2JRQ6	
NaN	NaN	NaN	NaN	3	143,72	G1UI17;A0	G1UI17;A0	Glycogen d
22,8887	23,343	29,2076	28,7794	3	57,838	B4DRR0;C0	B4DRR0;C0	Keratin, typ
NaN	NaN	NaN	NaN	3	32,673	A0A0U1RQ	A0A0U1RQ	Brain-speci
20,8242	NaN	NaN	NaN	3	35,549	Q59H49;A6	Q59H49;A6	Polypyrimi
24,3973	24,4382	NaN	NaN	3	11,845	A0A0X9T7	A0A0X9T7V9	
NaN	NaN	NaN	NaN	3	12,107	A0A0X9V9	A0A0X9V9B3	
NaN	NaN	NaN	NaN	3	11,464	A0A125U0	A0A125U0U6;A0A0X9	
25,4281	25,7128	21,706	22,4356	3	13,096	A0A125U0	A0A125U0U7	
NaN	NaN	NaN	NaN	3	38,737	Q86U79;A0	Q86U79;A0	Adenosine
NaN	NaN	NaN	NaN	3	54,582	J3K000;A0	J3K000;A0	Xaa-Pro dip
NaN	NaN	NaN	NaN	3	35,575	P62714;Q8	P62714;Q8	Serine/thre
NaN	21,1071	21,1343	21,4223	3	20,207	B7Z478;A0	B7Z478;A0	Proteasom
NaN	21,7193	NaN	NaN	3	10,364	Q5NV73;Q	Q5NV73;Q	Ig lambda c
NaN	NaN	NaN	NaN	3	25,948	Q9HC80;Q	Q9HC80;Q	Kallikrein-9
20,9303	21,4525	23,2264	23,1786	3	17,965	P62979;F5	P62979;F5	Ubiquitin-4
NaN	NaN	22,1081	21,7	3	28,708	Q96DV6;A2	Q96DV6;A2	40S ribosor
NaN	22,2327	22,9947	22,6161	3	10,685	A2J1M8	A2J1M8	
24,836	24,889	22,2907	23,2812	3	12,388	A2NB45;A0	A2NB45	

NaN	NaN	21,2713	21,4821	3	21,893	Q4VB24;B2	Q4VB24;B2	Histone H1
NaN	NaN	NaN	NaN	3	12,389	E5RFR7;H0	E5RFR7;H0	Tumor prot
NaN	NaN	21,3095	NaN	3	15,906	B4DPP0;A6	B4DPP0;A6	Tetraspanir
20,2307	20,1431	21,8856	NaN	3	45,604	B3KQV6;Q8	B3KQV6;Q8	Serine/thre
NaN	NaN	NaN	NaN	3	27,666	A9UFC0;B2	A9UFC0;B2	Caspase-14
NaN	NaN	NaN	NaN	3	30,858	B1AKG0;Q(	B1AKG0;Q(	Complemer
NaN	NaN	NaN	NaN	3	58,661	B4DP06;V9	B4DP06;V9	Bifunctiona
NaN	NaN	NaN	NaN	3	26,726	B3KV49;B4	B3KV49;B4	Glyoxalase
NaN	NaN	NaN	21,1875	3	55,674	B4DUR8;B3	B4DUR8;B3	T-complex
NaN	NaN	NaN	NaN	3	75,63	X5D7S8;B4	X5D7S8;B4	Major vault
NaN	NaN	NaN	NaN	3	27,021	B4DKM4;Q	B4DKM4;Q	Carcinoeml
NaN	NaN	NaN	NaN	3	49,921	B7Z4L4;Q9	B7Z4L4;Q9	Dolichyl-di
NaN	NaN	23,0492	23,405	3	49,597	F5GZQ3;B4	F5GZQ3;B4	Trifunction
NaN	NaN	NaN	NaN	3	39,811	B4E2S7;P1	B4E2S7;P1	Lysosome-a
NaN	NaN	19,6798	NaN	3	54,269	B4E324;X6	B4E324;X6	Carboxypep
NaN	NaN	NaN	NaN	3	40,876	Q16769;B5	Q16769;B5	Glutaminyl
NaN	NaN	NaN	NaN	3	18,976	Q5ISJ3;E5R	Q5ISJ3;E5R	Stathmin;S
20,6955	20,742	21,1572	21,429	3	47,766	Q5JP53;Q5	Q5JP53;Q5	Tubulin bet
20,5739	20,5594	NaN	19,8819	3	54,576	Q96N83;B7	Q96N83;B7	Podocalyxi
NaN	21,4196	21,4357	21,6524	3	12,203	P06454;Q1	P06454;Q1	Prothymos
21,8583	22,1004	20,6484	20,3135	3	24,158	C9JF17;P0	C9JF17;P0	Apolipoprc
NaN	NaN	NaN	NaN	3	24,409	CON__P007	CON__P00761	
20,0014	20,1989	20,5514	NaN	3	13,562	D3DQX7;E9	D3DQX7;E9	Serum amy
NaN	NaN	NaN	NaN	3	54,924	D3DVW9;P	D3DVW9;P	Tyrosine-pr
NaN	NaN	21,4057	21,7215	3	47,401	H0Y704;Q6	H0Y704;Q6	Zinc finger
NaN	NaN	NaN	NaN	3	20,79	H0YKU1;H(	H0YKU1;H(	Tropomodu
NaN	NaN	NaN	NaN	3	34,684	H3BLU7;V9	H3BLU7;V9	Aflatoxin B
NaN	NaN	21,9515	21,5916	3	44,634	H3BRG4;P2	H3BRG4;P2	Cytochrom
NaN	NaN	NaN	NaN	3	18,888	K7EII6;K7E	K7EII6;K7E	Echinoderm
NaN	NaN	NaN	NaN	3	40,475	O15335	O15335	Chondroad
NaN	NaN	NaN	NaN	3	29,815	O15400;B4	O15400;B4	Syntaxin-7
21,8438	21,8064	NaN	NaN	3	16,859	Q8TDZ6;O6	Q8TDZ6;O6	Glia matura
NaN	NaN	NaN	NaN	3	58,502	O95498;E9	O95498;E9	Vascular nc
NaN	NaN	NaN	NaN	3	29,644	V9HW53;O	V9HW53;O	N(G),N(G)-c
22,4683	22,2954	NaN	NaN	3	39,029	P00739;A0	P00739;A0	Haptoglobi
NaN	NaN	NaN	NaN	3	11,006	P01040;C9	P01040;C9	Cystatin-A;
23,2968	22,9597	22,5443	22,0335	3	10,649	V9GYG9;V9	V9GYG9;V9	Apolipoprc
26,9517	NaN	27,3776	27,6347	3	7,3044	P02808	P02808	Statherin
24,2359	24,0936	NaN	24,5681	3	9,681	R4GN98;P(	R4GN98;P(	Protein S1(
19,9179	20,5707	NaN	NaN	3	80,214	P08582;C9	P08582	Melanotrar
NaN	NaN	NaN	NaN	3	14,716	P09382;F8	P09382	Galectin-1
24,9351	25,2071	23,4288	23,7954	3	23,379	P0DOX7	P0DOX7	
23,0983	23,5851	NaN	21,2821	3	18,354	W0UV60;P	W0UV60;P	Non-secret
NaN	NaN	NaN	NaN	3	37,495	P25774;U3	P25774;U3	Cathepsin S
NaN	NaN	NaN	NaN	3	28,993	P30040;V9	P30040;V9	Endoplasm
NaN	NaN	20,4143	20,4904	3	27,624	Q53HC2;P3	Q53HC2;P3	Thioredoxi
NaN	NaN	NaN	NaN	3	50,91	Q53GL5;P4	Q53GL5;P4	Isocitrate d
NaN	NaN	NaN	NaN	3	11,186	P55000	P55000	Secreted Ly
NaN	NaN	20,1423	NaN	3	9,1175	P61960;H(	P61960;H(	Ubiquitin-f
21,098	21,2754	NaN	NaN	3	28,302	P61981;B4	P61981;B4	14-3-3 prot
NaN	NaN	22,1315	22,2616	3	21,879	Q5JR95;Q5	Q5JR95;Q5	40S ribosor
NaN	NaN	21,8122	22,0866	3	17,718	P62269;A0	P62269;A0	40S ribosor

NaN	NaN	21,6533	22,7746	3	29,995	P62424;Q9	P62424;Q9	60S ribosor
NaN	NaN	NaN	NaN	3	5,0256	P63313;D6	P63313	Thymosin b
NaN	NaN	NaN	NaN	3	21,308	Q6ICQ8;P8	Q6ICQ8;P8	Rho-relatec
NaN	NaN	NaN	NaN	3	20,777	X5DNM4;V	X5DNM4;V	Lactoylglut
NaN	NaN	NaN	NaN	3	13,286	Q14210	Q14210	Lymphocyt
NaN	NaN	NaN	NaN	3	7,8547	Q5TBU5;Q1	Q5TBU5;Q1	Adipogenes
NaN	21,9857	NaN	22,0621	3	36,431	Q16651;H3	Q16651	Prostasin;P
26,2813	26,6614	NaN	NaN	3	77,079	Q53H26	Q53H26	
NaN	NaN	NaN	NaN	3	72,044	Q5HYM2;Q	Q5HYM2;Q	Vacuolar pr
21,9482	21,3006	NaN	NaN	3	34,977	Q5IWS5;Q8	Q5IWS5;Q8	Intelectin-1
22,1463	21,7605	22,12	21,4613	3	10,643	Q5NV88;A0	Q5NV88	
NaN	NaN	25,7941	24,0804	3	15,43	Q5TEC6	Q5TEC6	Histone H3
NaN	NaN	NaN	NaN	3	131,6	Q6MZM0	Q6MZM0	Hephaestin
23,2119	NaN	NaN	NaN	3	52,042	Q6MZQ6;A0	Q6MZQ6	
20,7026	NaN	NaN	NaN	3	54,159	Q6N091	Q6N091	
23,5725	25,4197	23,9226	24,078	3	25,773	Q6P5S8	Q6P5S8	
NaN	NaN	NaN	NaN	3	25,015	Q7Z2U7	Q7Z2U7	
NaN	NaN	NaN	NaN	3	91,624	Q86VR7	Q86VR7	V-set and ir
NaN	NaN	NaN	NaN	3	66,175	S4R3V8;Q8	S4R3V8;Q8	Lipolysis-st
NaN	NaN	NaN	23,4232	3	24,792	Q8N355;P8	Q8N355	
NaN	NaN	NaN	NaN	3	80,25	Q8TE68;K7	Q8TE68;K7	Epidermal g
NaN	NaN	NaN	NaN	3	18,795	Q969H8;M	Q969H8;M	Myeloid-de
NaN	NaN	25,1543	25,351	3	533,64	Q96M86;E9	Q96M86;E9	Dynein hea
NaN	NaN	NaN	NaN	3	7,8052	Q96RM1	Q96RM1	Small prolir
22,2875	22,8314	NaN	NaN	3	27,216	Q99935	Q99935	Proline-rich
NaN	NaN	NaN	NaN	3	54,341	Q9UHL4;B4	Q9UHL4;B4	Dipeptidyl
NaN	21,675	NaN	NaN	3	11,928	Q9UL86	Q9UL86	
22,9667	23,3828	21,8105	22,4283	3	12,437	Q9UL90;A0	Q9UL90;A0A0J9YY99	
NaN	NaN	NaN	NaN	3	15,892	Q9Y2V2;H3	Q9Y2V2;H3	Calcium-reg
NaN	NaN	NaN	NaN	2	34,165	P50225;A0	P50225;A0	Sulfotransfe
NaN	NaN	21,1217	21,41	2	14,529	F8VUA6;Q0	F8VUA6;Q0	60S ribosor
NaN	NaN	NaN	NaN	2	45,233	A0A0A0MR	A0A0A0MR	NAD kinase
20,5572	20,6665	NaN	NaN	2	28,001	H0Y3T6;G3	H0Y3T6;G3	45 kDa calc
22,0478	21,5012	NaN	NaN	2	8,647	K7ERI9;A0A	K7ERI9;A0A	Apolipopro
NaN	NaN	NaN	NaN	2	11,897	F2Z2F1;B0(	F2Z2F1;B0(	Myoglobin
NaN	NaN	NaN	NaN	2	64,24	B4DE32;B1	B4DE32;B1	X-ray repair
NaN	22,0064	NaN	NaN	2	29,645	A8K1D2;A0	A8K1D2;A0	LIM and SH
NaN	NaN	25,0434	25,1287	2	29,979	A0A024R1X	A0A024R1X6	
NaN	NaN	NaN	NaN	2	37,53	B3KQK4;A5	B3KQK4;A5	Cathepsin L
NaN	NaN	NaN	NaN	2	9,5355	A6NL93;Q6	A6NL93;Q6	Non-histon
NaN	NaN	NaN	19,9153	2	58,022	O95747;A0	O95747;A0	Serine/thre
NaN	NaN	NaN	20,0739	2	76,666	Q5ISL3;B4D	Q5ISL3;B4D	Catenin bet
NaN	NaN	22,8353	22,4885	2	15,722	E7EX53;E7I	E7EX53;E7I	Ribosomal
NaN	NaN	NaN	NaN	2	97,539	A0A024R2V	A0A024R2V	Dystroglyca
21,142	21,0966	21,328	21,246	2	33,117	P02686;E9	P02686;E9	Myelin basi
NaN	NaN	NaN	NaN	2	16,892	B4E0Z3;C9.	B4E0Z3;C9.	Lamin-B rec
NaN	NaN	NaN	NaN	2	16,111	C4P0D4;C4	C4P0D4;C4	Translin-ass
NaN	NaN	NaN	NaN	2	7,5406	A0A024R3X	A0A024R3X	10 kDa hea
NaN	17,7858	NaN	NaN	2	54,661	H0YCY6;A0	H0YCY6;A0	Bifunctiona
NaN	NaN	NaN	22,1291	2	25,609	A0A024R5(	A0A024R5(	Reticulon;F
NaN	NaN	NaN	NaN	2	10,058	O75531;A0	O75531;A0	Barrier-to-a
21,3708	21,365	NaN	NaN	2	23,549	Q6FGX3;Q6	Q6FGX3;Q6	Ras-related

NaN	NaN	NaN	NaN	2	19,943	H3BMS6;H:	H3BMS6;H:	Sulfide:quir
NaN	NaN	NaN	NaN	2	11,399	Q7Z612;Q6	Q7Z612;Q6	60S acidic r
NaN	NaN	NaN	NaN	2	44,453	Q6FG59;A0	Q6FG59;A0	Hsp90 co-c
NaN	NaN	NaN	19,4155	2	51,377	K7ERN2;Q5	K7ERN2;Q5	Intercellula
NaN	NaN	NaN	NaN	2	26,807	B4DUJ3;V9	B4DUJ3;V9	Carbonic ar
NaN	NaN	20,2462	20,0894	2	10,871	A0A024R8S	A0A024R8S	Small ubiqu
NaN	NaN	NaN	NaN	2	32,117	B3KS64;B4	B3KS64;B4	Fibromodu
NaN	NaN	NaN	NaN	2	70,67	P11940;E7	P11940;E7	Polyadenyl
NaN	NaN	NaN	NaN	2	10,979	E5RJY1;E7E	E5RJY1;E7E	Protein ND
NaN	NaN	NaN	19,9697	2	61,891	B4DIU3;A0	B4DIU3;A0	Protein-arg
NaN	NaN	20,2992	NaN	2	18,697	A0A024RB:	A0A024RB:	Prostagland
NaN	NaN	NaN	NaN	2	15,016	F8VZJ2;F8V	F8VZJ2;F8V	Nascent po
NaN	NaN	NaN	NaN	2	17,279	H0YIN7;F8\	H0YIN7;F8\	Proliferatic
NaN	NaN	20,4023	20,583	2	58,15	B3KVX6;Q8	B3KVX6;Q8	Cytoskeletc
NaN	NaN	NaN	NaN	2	37,111	D6RF62;A0	D6RF62;A0	Multifuncti
NaN	NaN	20,2604	NaN	2	35,32	Q4W5K9;A	Q4W5K9;A	PDZ and LIN
NaN	NaN	NaN	NaN	2	27,379	F8W943;A(	F8W943;A(	Argininosu(
NaN	NaN	NaN	NaN	2	13,158	A0A068LK(	A0A068LKQ2	
NaN	NaN	NaN	NaN	2	13,384	A0A068LN(	A0A068LN03	
NaN	NaN	NaN	NaN	2	10,375	Q5NV62;A(	Q5NV62;A0A075B6I0	
20,9275	NaN	NaN	NaN	2	12,848	A0A075B6I	A0A075B6R2;A0A087V	
NaN	NaN	NaN	21,1697	2	13,143	A0A075B6S	A0A075B6S2;A2NJV5;/	
NaN	NaN	NaN	NaN	2	553,12	A0A075B7:	A0A075B7:	Epiplakin
NaN	NaN	21,1306	NaN	2	15,958	K7EM73;K7	K7EM73;K7	Calpain sma
NaN	NaN	NaN	NaN	2	13,08	A0A075B7I	A0A075B7D8	
NaN	NaN	NaN	NaN	2	11,434	A0A087X0(	A0A087X0(	Ig kappa ch
NaN	NaN	NaN	NaN	2	33,879	Q9NP79;A(	Q9NP79;A(	Vacuolar pr
19,7692	19,9949	NaN	NaN	2	28,122	Q9Y5B2;A0	Q9Y5B2;A0	Junctional
NaN	NaN	NaN	NaN	2	12,586	Q5J908;A0	Q5J908;A0	Tumor prot
NaN	22,2988	NaN	NaN	2	24,421	A0A087WZ	A0A087WZ	Low affinity
NaN	NaN	NaN	NaN	2	77,569	A0A087X0)	A0A087X0)	Heterogene
NaN	NaN	21,708	21,5922	2	23,608	A0A088LVI	A0A088LVH8	
NaN	NaN	NaN	NaN	2	11,578	M0QZK8;A(	M0QZK8;A(	Gamma-glu
NaN	NaN	NaN	NaN	2	14,715	A0A0A0MC	A0A0A0MC	Kallikrein-8
NaN	NaN	NaN	NaN	2	12,839	K7EJ28;Q6	K7EJ28;Q6	Tumor necr
NaN	22,2148	NaN	NaN	2	11,041	A2MYC8;A(	A2MYC8;A0A0B4J1Y8;(	
NaN	21,8677	NaN	NaN	2	12,758	A0A0C4DH	A0A0C4DH36	
NaN	NaN	NaN	NaN	2	13,312	A0A0C4DH	A0A0C4DH43;A0A0F7S	
NaN	NaN	NaN	NaN	2	47,113	Q4W5P3;B	Q4W5P3;B	Transmemb
NaN	NaN	NaN	NaN	2	11,142	Q5NV82;A(	Q5NV82;A0A0G2JSC0;/	
NaN	NaN	NaN	NaN	2	32,708	P09493;A0	P09493;A0	Tropomyos
NaN	NaN	NaN	19,7626	2	24,16	H0YK49;H(	H0YK49;H(	Electron tra
NaN	NaN	NaN	19,5892	2	102,48	H7C5W9;A	H7C5W9;A	Sarcoplasm
NaN	NaN	NaN	NaN	2	14,221	G3V461;G:	G3V461;G:	Creatine kir
NaN	NaN	NaN	18,1138	2	54,636	A0A0S2Z4(	A0A0S2Z4(	Fumarate h
NaN	NaN	20,0518	19,6516	2	53,928	E7ER27;B4I	E7ER27;B4I	Peroxisoma
24,6934	24,4289	22,2001	22,2045	2	12,468	A0A0X9UW	A0A0X9UWL5;A0A0X9T	
21,7963	21,8215	NaN	NaN	2	14,369	A0A0X9UW	A0A0X9UWM4	
NaN	NaN	NaN	NaN	2	13,228	A0A0X9V9(	A0A0X9V9C4	
22,2648	22,4439	NaN	NaN	2	13,509	A0A125U0'	A0A125U0V1	
21,4073	NaN	NaN	19,9049	2	13,781	A0A125U0'	A0A125U0V4	
22,0023	21,8206	NaN	NaN	2	187,66	A0A140TA2	A0A140TA29;F5GXS0;/	

NaN	NaN	NaN	19,8849	2	30,037	A0A140VJG	A0A140VJG	Catechol O-
NaN	NaN	NaN	NaN	2	33,178	A0A140VJX	A0A140VJX	3-mercapto
NaN	NaN	NaN	NaN	2	18,36	B4DFL3;Q5	B4DFL3;Q5	Proteasome
NaN	NaN	NaN	NaN	2	16,219	H7C3I1;F6\	H7C3I1;F6\	Putative pr
NaN	NaN	NaN	NaN	2	25,789	A0A140VK/	A0A140VK/	Dihydropte
25,6375	25,9216	26,0235	25,857	2	14,481	A0A193CH(	A0A193CHQ9;A0A0B4J	
21,8355	NaN	NaN	NaN	2	3,7183	A0A1B1CY(	A0A1B1CYC5	
NaN	NaN	NaN	NaN	2	14,566	A0A1C9J6F	A0A1C9J6R3;A0A1C9J6	
NaN	NaN	NaN	NaN	2	16,695	B4DEI3;B3I	B4DEI3;B3I	Voltage-dep
NaN	NaN	NaN	NaN	2	16,703	Q4V347;AC	Q4V347;AC	Allograft in
NaN	NaN	NaN	NaN	2	49,896	Q0EFA5;A8	Q0EFA5;A8	Corneodesr
NaN	NaN	NaN	NaN	2	59,897	A0A1W2PF	A0A1W2PF	NAD-depen
NaN	NaN	NaN	NaN	2	13,133	A0A1W2PF	A0A1W2PF	Alpha-endo
NaN	NaN	NaN	NaN	2	13,51	A0A1W6IYI	A0A1W6IYI3;A0A1W6IY	
25,5275	26,1372	24,7517	23,8681	2	13,519	A0A1W6IYI	A0A1W6IYI5;A0A1W6IY	
NaN	NaN	NaN	NaN	2	13,673	A0A1W6IYI	A0A1W6IYI9;A0A1W6IY	
21,4204	NaN	NaN	NaN	2	19,46	H0YDX6;AC	H0YDX6;AC	CD44 antig
NaN	NaN	NaN	20,4853	2	16,363	A0M8W4;I:	A0M8W4;I:	Ubiquitin-c
NaN	NaN	NaN	NaN	2	12,528	A0N5G1;P(	A0N5G1;P(	Ig kappa ch
22,0471	22,1648	NaN	NaN	2	14,534	A0N7J6	A0N7J6	
NaN	21,0838	NaN	NaN	2	12,383	A2IPI6	A2IPI6	
NaN	NaN	22,8586	23,3761	2	10,499	A2J1N0	A2J1N0	
NaN	NaN	NaN	NaN	2	10,45	A2J1N5	A2J1N5	
NaN	NaN	NaN	21,2765	2	12,626	A2J1N6;A0	A2J1N6	
NaN	NaN	23,5411	22,1725	2	10,496	A2J1N7	A2J1N7	
21,482	21,6232	NaN	NaN	2	13,908	A2JA14;A2.	A2JA14	
NaN	20,5315	NaN	NaN	2	11,621	A2JA16	A2JA16	
21,6594	22,1346	NaN	NaN	2	11,886	A2N2F4	A2N2F4	
20,2402	20,234	NaN	NaN	2	27,548	A2NX48	A2NX48	
NaN	NaN	NaN	NaN	2	13,992	A2NYU7	A2NYU7	
NaN	NaN	NaN	NaN	2	12,675	A2NYU8	A2NYU8	
NaN	NaN	NaN	NaN	2	20,811	A4D177;Q1	A4D177;Q1	Chromobo:
NaN	NaN	NaN	20,8997	2	25,525	A8K3C1;S4	A8K3C1;S4	Vacuolar pr
NaN	NaN	20,9456	21,2012	2	14,692	J3QSB4;Q6	J3QSB4;Q6	60S ribosor
NaN	NaN	NaN	NaN	2	13,212	K7EP16;K7	K7EP16;K7	Eukaryotic
NaN	NaN	NaN	NaN	2	104,72	A8K8Z4;P1	A8K8Z4;P1	Complemer
NaN	17,9855	NaN	NaN	2	33,67	P07910;G3	P07910;G3	Heterogene
NaN	NaN	NaN	NaN	2	51,911	X6R9L0;A8	X6R9L0;A8	DnaJ homo
NaN	NaN	NaN	NaN	2	57,21	B0YIW5;Q6	B0YIW5;Q6	Coatomer s
NaN	NaN	NaN	20,6994	2	8,1894	M0R2L9;M	M0R2L9;M	40S ribosor
NaN	NaN	NaN	NaN	2	11,796	B1N7B8	B1N7B8	
NaN	NaN	22,4338	NaN	2	27,259	Q96IR1;Q5	Q96IR1;Q5	40S ribosor
NaN	NaN	NaN	NaN	2	16,177	M0R0P7;M	M0R0P7;M	60S ribosor
NaN	NaN	NaN	20,6185	2	11,477	I3L3P7;I3L:	I3L3P7;I3L:	40S ribosor
NaN	NaN	NaN	NaN	2	22,537	B2R582;Q6	B2R582;Q6	Tetranectir
NaN	NaN	NaN	NaN	2	24,956	B2R5I8;Q1:	B2R5I8;Q1:	Ras-related
NaN	NaN	NaN	NaN	2	18,979	Q5T0D2;B4	Q5T0D2;B4	UMP-CMP I
NaN	NaN	24,3834	24,1002	2	60,026	B2R853;B4	B2R853;B4DRY0	
NaN	NaN	NaN	20,1278	2	57,003	B2RAN2;O9	B2RAN2;O9	Pantethein
NaN	19,6397	NaN	NaN	2	33,173	H3BTQ8;H3	H3BTQ8;H3	Kunitz-type
NaN	NaN	NaN	NaN	2	38,505	B4E1F5;H0	B4E1F5;H0	Pulmonary
NaN	NaN	20,5679	NaN	2	36,053	B3KM97;Q!	B3KM97;Q!	Very-long-c

NaN	NaN	NaN	NaN	2	27,626	B3KP25;Q9	B3KP25;Q9	Serine prot
NaN	16,6858	NaN	NaN	2	48,036	Q05DJ8;B3	Q05DJ8;B3	Serine prot
NaN	NaN	22,0141	NaN	2	52,342	B3KS49	B3KS49	
NaN	NaN	NaN	NaN	2	43,032	Q96GI1;B4	Q96GI1;B4	T-complex
NaN	NaN	20,3856	20,4794	2	62,609	B4DDZ5;B4	B4DDZ5;B4	Trifunction
NaN	NaN	NaN	NaN	2	23,417	B4DF38;I3I	B4DF38;I3I	Platelet-act
NaN	NaN	NaN	NaN	2	30,913	B4DKH5;B4	B4DKH5;B4	AP complex
NaN	NaN	NaN	NaN	2	20,018	B4DKK9;Q6	B4DKK9;Q6	DnaJ homo
NaN	NaN	NaN	NaN	2	16,637	F8W0G4;F8	F8W0G4;F8	Poly(rC)-bir
NaN	NaN	NaN	NaN	2	35,536	Q53FA7;B4	Q53FA7;B4	Quinone ox
NaN	NaN	NaN	NaN	2	19,796	B4DNR3;B4	B4DNR3;B4	Alpha/beta
NaN	NaN	20,7408	20,6361	2	40,372	B4DUL5;P3	B4DUL5;P3	Cytochrom
NaN	NaN	NaN	NaN	2	26,936	Q6UWN5;B	Q6UWN5;B	Ly6/PLAUR
NaN	NaN	NaN	NaN	2	62,699	B4DY46;O0	B4DY46;O0	Syntaxin-bi
NaN	NaN	NaN	NaN	2	22,127	P62081;B5	P62081;B5	40S ribosor
NaN	NaN	NaN	NaN	2	13,737	C9IY94;C9I	C9IY94;C9I	Septin-2
NaN	NaN	NaN	NaN	2	13,288	B6EDE2;A2	B6EDE2	
NaN	NaN	NaN	NaN	2	21,649	B7Z5R3;B7	B7Z5R3;B7	Src kinase-a
NaN	NaN	NaN	NaN	2	41,261	B7ZKW8;Q	B7ZKW8;Q	CapZ-intera
NaN	NaN	NaN	NaN	2	47,082	Q6E0U4;C0	Q6E0U4;C0	Dermokine
NaN	NaN	NaN	NaN	2	43,9	CON__ENSE	CON__ENSEMBL:ENSBTA	
NaN	NaN	NaN	NaN	2	59,504	CON__O95	CON__O95	Keratin, typ
NaN	NaN	23,2478	23,3392	2	24,348	CON__P02	CON__P02663	
NaN	NaN	NaN	NaN	2	44,091	CON__P08	CON__P08	Keratin, typ
NaN	NaN	22,5119	NaN	2	47,754	CON__P08	CON__P08730-1	
NaN	NaN	20,7516	NaN	2	58,923	CON__Q32	CON__Q32	Keratin, typ
23,063	NaN	NaN	NaN	2	57,407	CON__Q3N	CON__Q3MHH8	
NaN	NaN	NaN	NaN	2	22,06	CON__Q3S	CON__Q3SX09;CON__P	
21,7019	NaN	NaN	NaN	2	62,87	CON__Q3ZI	CON__Q3ZBD7	
20,5511	20,8072	21,356	21,5956	2	248,07	CON__Q5D	CON__Q5D	Filaggrin-2
NaN	NaN	NaN	21,7338	2	10,662	CON__Q61	CON__Q61782	
NaN	NaN	NaN	19,7147	2	282,39	CON__Q86	CON__Q86	Hornerin
NaN	NaN	NaN	NaN	2	56,648	Q701L7;CC	Q701L7;CC	Keratin, typ
NaN	NaN	NaN	NaN	2	42,44	Q63HR1;Q5	Q63HR1;Q5	Plasminoge
NaN	NaN	NaN	20,2274	2	11,959	D3DWI6;O	D3DWI6;O	Prostate ste
NaN	NaN	NaN	NaN	2	49,061	D3DXC9;Q5	D3DXC9;Q5	Serine hydr
22,6709	22,4165	NaN	NaN	2	6,5625	E5RFX6;P5	E5RFX6;P5	Serine prot
NaN	NaN	20,903	20,4263	2	18,72	E5RJR5;P6	E5RJR5;P6	S-phase kin
NaN	NaN	NaN	NaN	2	36,456	F5H282;E7	F5H282;E7	T-complex
NaN	NaN	NaN	NaN	2	21,154	H0YEN5;E9	H0YEN5;E9	40S ribosor
NaN	NaN	22,743	22,9045	2	21,062	F8W148	F8W148	
NaN	NaN	NaN	NaN	2	14,411	G1FM85;P(	G1FM85;P(	Ig heavy cha
NaN	NaN	NaN	NaN	2	17,075	G3V1V8;Q7	G3V1V8;Q7	Myosin regu
NaN	NaN	NaN	NaN	2	40,798	Q9UN36;G	Q9UN36;G	Protein ND
NaN	NaN	NaN	NaN	2	57,643	H0Y2Y8;Q1	H0Y2Y8;Q1	Zyxin
NaN	NaN	NaN	NaN	2	84,69	H3BQZ7;Q1	H3BQZ7;Q1	Heterogene
NaN	NaN	20,4916	20,4276	2	16,019	I3L397;I3L	I3L397;I3L	Eukaryotic
NaN	NaN	NaN	NaN	2	12,476	I4AY87;P14	I4AY87;P14	Macrophag
NaN	NaN	NaN	NaN	2	9,5162	J3KSH8;J3K	J3KSH8;J3K	Hematolog
NaN	NaN	21,7333	NaN	2	2,8323	K4EN11	K4EN11	
NaN	NaN	NaN	NaN	2	22,49	K7ER15;V9	K7ER15;V9	Haloacid de
NaN	NaN	NaN	NaN	2	17,195	M0R2E9;M	M0R2E9;M	Urokinase

21,5507	21,9025	NaN	NaN	2	19,116	O60888;C9	O60888;C9	Protein Cut
NaN	NaN	NaN	20,3104	2	61,603	Q53GY1;O9	Q53GY1;O9	BAG family
30,2987	30,3337	NaN	NaN	2	40,799	P02812	P02812	Basic saliva
30,8638	30,4807	23,7842	23,785	2	8,1875	P02814;Q5	P02814;Q5	Submaxilla
NaN	NaN	NaN	NaN	2	53,688	P04066;B7	P04066;B7	Tissue alpha
NaN	NaN	NaN	NaN	2	9,1494	P04155	P04155	Trefoil facto
NaN	NaN	NaN	20,2042	2	56,367	Q53FB6;P0	Q53FB6;P0	Aldehyde d
NaN	NaN	NaN	NaN	2	56,957	Q8IVC0;P0	Q8IVC0;P0	Heparin co
NaN	NaN	NaN	NaN	2	52,948	R4SBI6;Q6	R4SBI6;Q6	Epoxide hy
NaN	NaN	22,0957	21,715	2	20,863	P07305	P07305	Histone H1
NaN	NaN	NaN	NaN	2	22,277	P07360;Q5	P07360;Q5	Complemen
NaN	NaN	NaN	NaN	2	32,85	P07951;Q5	P07951;Q5	Tropomyos
24,3348	24,7479	23,929	24,4011	2	22,83	P0DOX8;Q6	P0DOX8;Q6	PIK1
NaN	NaN	NaN	NaN	2	223,11	V9HWC1;P	V9HWC1;P	Myosin-1;M
NaN	NaN	NaN	NaN	2	117,97	P14735;B7	P14735;B7	Insulin-deg
NaN	25,0127	NaN	NaN	2	6,9628	P15515	P15515	Histatin-1;I
NaN	NaN	NaN	NaN	2	9,8875	P22528	P22528	Cornifin-B
NaN	NaN	NaN	NaN	2	13,085	P23083;A0	P23083;A0	Ig heavy cha
NaN	NaN	NaN	NaN	2	14,515	P25398	P25398	40S ribosor
20,945	20,9762	20,633	19,9812	2	11,728	P26447	P26447	Protein S10
NaN	NaN	NaN	NaN	2	25,357	Q6IAT9;P28	Q6IAT9;P28	Proteasome
NaN	NaN	NaN	NaN	2	72,968	P33908	P33908	Mannosyl-o
NaN	NaN	NaN	NaN	2	9,8774	P35321	P35321	Cornifin-A
NaN	NaN	NaN	NaN	2	26,145	Q53Y06;P3	Q53Y06;P3	V-type prot
NaN	NaN	NaN	NaN	2	23,662	Q5QTS3;Q5	Q5QTS3;Q5	60S ribosor
NaN	NaN	NaN	NaN	2	61,132	P43251;C9	P43251;C9	Biotinidase
NaN	NaN	NaN	NaN	2	45,402	P48595	P48595	Serpin B10
NaN	NaN	22,6483	23,0887	2	49,541	P49411;H3	P49411;H3	Elongation
NaN	NaN	NaN	NaN	2	7,8501	P59768;G3	P59768;G3	Guanine nu
NaN	NaN	NaN	NaN	2	11,05	P60985	P60985	Keratinocyt
NaN	NaN	21,9449	NaN	2	22,541	P61026;Q5	P61026;Q5	Ras-related
NaN	NaN	22,6445	22,798	2	10,366	Q6FGH9;P6	Q6FGH9;P6	Dynein ligh
25,8844	26,0126	25,315	25,4406	2	24,03	Q0KKI6;Q8	Q0KKI6;Q8	Ig kappa ch
NaN	NaN	NaN	NaN	2	26,697	Q6SYC2;Q1	Q6SYC2;Q1	Nectin-1
21,336	21,5471	21,5333	21,6923	2	9,0714	Q15843;F8	Q15843;F8	NEDD8
NaN	NaN	NaN	19,8066	2	72,307	Q53GW1;C	Q53GW1;C	Sec1 family
NaN	NaN	NaN	NaN	2	42,003	Q562R1	Q562R1	Beta-actin-l
20,9262	20,9712	NaN	NaN	2	10,335	Q5NV69;P0	Q5NV69;P0	Ig lambda c
NaN	NaN	NaN	NaN	2	10,229	Q5NV91;P0	Q5NV91;P0	Ig lambda c
NaN	NaN	NaN	NaN	2	10,573	Q5NV92;A2	Q5NV92;A2	N2G8;A0A0
NaN	NaN	NaN	NaN	2	24,809	Q6GMX4	Q6GMX4	
22,3867	22,5945	NaN	NaN	2	51,724	Q6N089;P0	Q6N089	
23,8756	NaN	NaN	NaN	2	46,06	Q6N093;P0	Q6N093;P0	Ig gamma-2
NaN	25,8461	NaN	NaN	2	12,362	Q6UW32	Q6UW32	Insulin grov
NaN	NaN	NaN	NaN	2	161,69	Q6YHK3	Q6YHK3	CD109 anti
NaN	NaN	NaN	NaN	2	72,426	Q6ZN66;B4	Q6ZN66;B4	Guanylate-l
25,019	NaN	24,7211	24,7811	2	52,874	Q6ZVX0;Q6	Q6ZVX0;Q6	MZX9
NaN	NaN	NaN	NaN	2	51,62	Q7Z379	Q7Z379	
19,0932	NaN	NaN	NaN	2	106,26	Q7Z7M9;A5	Q7Z7M9;A5	Polypeptid
22,0767	21,7736	NaN	NaN	2	24,961	Q8N5F4	Q8N5F4	
NaN	NaN	NaN	22,4431	2	53,224	Q8NCL6;Q9	Q8NCL6;Q9	Ig alpha-1 c
NaN	NaN	NaN	NaN	2	25,024	Q8NEJ1	Q8NEJ1	

NaN	NaN	21,1203	21,2982	2	1519,2	Q8WXI7	Q8WXI7	Mucin-16
NaN	NaN	21,3295	21,1255	2	10,1	Q96QR1	Q96QR1	Secretoglol
22,004	22,231	NaN	NaN	2	11,52	Q96SA9;A0	Q96SA9;A0	Ig kappa cha
NaN	20,5193	NaN	NaN	2	31,905	Q99674	Q99674	Cell growth
NaN	NaN	NaN	NaN	2	46,971	Q9BS26	Q9BS26	Endoplasm
NaN	NaN	NaN	NaN	2	8,1576	Q9BYE4	Q9BYE4	Small proli
NaN	NaN	NaN	NaN	2	14,246	Q9GZZ8;F8	Q9GZZ8;F8	Extracellula
22,1344	22,5074	20,8808	NaN	2	9,0564	Q9P1F3;Q5	Q9P1F3;Q5	Costars fam
NaN	NaN	22,8607	23,0644	2	9,7358	Q9UGL9	Q9UGL9	Cysteine-ric
NaN	NaN	NaN	NaN	2	13,623	Q9UHA4;Q	Q9UHA4;Q	Ragulator c
NaN	NaN	NaN	NaN	2	28,932	Q9UHY7;A0	Q9UHY7;A0	Enolase-pho
26,7459	26,7979	24,9483	24,6287	2	11,646	Q9UL78;A2	Q9UL78;A2NB46	
21,5038	21,5586	NaN	NaN	2	12,605	Q9UL89	Q9UL89	
NaN	NaN	NaN	NaN	2	58,561	REV__P064	REV__P06417;REV__A0	
NaN	NaN	NaN	NaN	2	50,87	S6B291	S6B291	
NaN	NaN	NaN	NaN	2	25,035	V9HVZ7	V9HVZ7	
23,5927	23,4142	23,3051	NaN	2	78,381	W8QEY1;Q	W8QEY1;Q2TUW9;B2M	

T: Gene names
MUC5B;MUC5AC
ALB
FCGBP
DSP;DSP variant protein
HEL-S-62p;C3
MYH9
AHNAK
A2M

HEL110;LTF
PPL
AMY1A
PIGR
KRT1
ACTN4
A2ML1
HEL-S-37;LCP1
KRT4

FLNA;FLJ00119
KRT13
PYGL
LPO
HEL70;MSN
KRT9
CP
KRT2
TLN1
MPO
KRT16
HEL107;TKT
SPRR3
PKM;HEL-S-30;PKM2
TGM3
IQGAP1;hCG_1991735
HSPA1A;HSPA1B;HEL-S-103
ENO1

HEL-S-78p;FGB
phosphate isomerase
KRT10
P4HB
DSG3
LTA4H
GDI2
GSN
HEL-S-89n;HSPA5
ITGAM
DMBT1
JUP
C4A

CAP1

CAT
EVPL
HSPA8;HEL-S-72p
NPEPPS
HEL-S-68p;PGK1
ANXA1
IVL
HEL113;VIM
SPINK5
APOA1
G6PD
CFB
KRT5
HEL-S-87p;ALDOA
EEF2
ACTG1
PGM2
VCL;HEL114
ACTN1
TPM3;DKFZp686J1372;TPM3-ROS1
AZGP1
MYH7;MYH6
CRNN
EL52;HSP90AA1
HEL-S-52;WDR1
LDHA;HEL-S-133P
HEL-S-51;GC
HEL57;SERPINB1
KRT78
hCG_40889;CFH;HF
FGG;DKFZp779N0926
NUCB1
APOB
HEL-S-162eP;GAPDH
SBSN
BPIFB1
ANXA2;HEL-S-270;ANXA2P2

LMNA
PSAP
HK3
HEL-S-109;NUCB2;Nucb2
DSC2;DKFZp686P18250;DKFZp686I11137
HP
HPX
PGD
IGK@
CST4
SFN

PGM1
KRT76
OLFM4
UBE1;UBA1
SERPINB5
CORO1A
PBEF1;NAMPT
SPARCL1
TALDO1
IGHM

QSOX1;BPGF-1
HEL-S-49;TPI1
DSG1
ACTR3
LGALS3BP
HEL-S-269;PDIA3
PLS3
PLG
ITGB2
RNH1
CTSD;HEL-S-130P
TGM1
FABP5
CHIT1
ERO1L
ARHGDIB
PZP
ALDH9A1
KRT14
EZR;HEL-S-105;EZR-ROS1
IGJ;JCHAIN
FGA
PGAM1;hCG_2015269
HEL-S-128m;PRDX6
TUBB4B;TUBB2C;TUBB4A
PRDX1
LEG1
BPIFB2
[illegible]S8
GOLPH2;GOLM1
HBB
SERPINA1
ARPC2
HEL-S-156an;PNP
CST1
ANXA3
CES1
HEL-S-99n;CALR
SERPINB3
ALDH3A1
NAGK

CAPN1
LCN1
CLTC
CD133;PROM1
ECM1
RNPEP
LYZ
YWHAZ
CAMP
PIP
PAM
ENDOU
BPIFA2
SERPINB13
MYH2;MYH8

CAPZA1
VAT1
FN1;DKFZp686O12165
PKP1
GOT1;GIG18
HEL-S-26;IDH1;HEL-216
CAPZB
TYMP;hCG_1988078
CPE
GRN
KNG1
CA6
CLIC1
GNAI2;WUGSC:H_LUCA16.1
S100A8
LDHB
PFN1
HEL-S-22;GSTP1
HEL-S-15;CFL1
ACTR2
CLCA4
UGP2
PSME1
ITIH2
MMP8
LCN2;NGAL
HEL-S-34;PEBP1
CRISP3
AKR1B10
SCEL
HEL-S-163pA;A1BG
ARG1
ATP5B;HEL-S-271
CLU
TAGLN2
HEL-S-70;VCP;DKFZp434K0126

MDH2
ADSS
CES2
FAM49B;DKFZp686B04128
THBS1
HSP90AB1
ADH7
CAST
ZG16B;PAUF;EECP
CST3
PLIN3
PSMA6
HEL-S-69p;PPIA
HIST1H4H;HIST1H4A
HDGF
ITIH4;DKFZp686G21125
TUBA1B;TUBA1C
APOH
DPP3;DKFZp686O1117
HEL-S-11;CA1
HEL-S-102;HSPB1
S100A9
HEL-S-39;PPIB
CALML3
HEL-S-55;PRDX5
S100A7
HSPA4;HEL-S-5a;HS24/p52
HEL-S-32;MDH1
EEF1A1P5;EEF1A1;EEF1A1L14;EEF1A2
RAB11B;RAB11A
NME1-NME2;NME2
ALDOC
SERPINC1
KLK1
KLK13

MNDA
CD14
HEL-S-21;GSTO1
HEL-S-66;CAPG
SERPINB10
HEL-S-164nA;GANAB

ITIH1
KRT36
TXN
H2AFY
APOA4
CST2
TCN1
AHCY
CRABP2

LSP1
HEL-S-64p;GSS
MSLN
AGT

VASP
SPTAN1
CDC42;hCG_39634
DSC3
FURIN
CDH1
PSMA7;PSMA8
GDI1
SERPINF1
HBA1;HBA2
TMSB4X
PIG59;GLUL
TIMP1
CALU
CTSC
GNB2L1
F2
ELANE;ELA2
CBR1
HIST1H1C
BPI
SPRR2D
HEL-S-2a;PRDX2
HEL2;YWHAE;YWHAE/FAM22B fusion;YWHAE/FAM22A fusion
HEBP2
COTL1
PDIA6
HMFT1766
NAPRT
HEL-S-67p;PARK7
CPPED1
ARF1;ARF3
LTB4DH;PTGR1
APEH
RHOA;hCG_2043376;RHOC;ARHA
HSPD1
KLK6
SERPINA3
RAB2;RAB2A;DKFZp313C1541;RAB2B
CHI3L1
HNRNPA2B1;HNRPA2B1
PSME2
FAM3D
PGLS
RAB7A
TMPRSS11D
FUT3;FUT5

PREP
CALM3;CALM2;CALM1
ANXA8;ANXA8L2;ANXA8L1

KRT17
VTN
MYL6
MYL12A;MYL12B;MYL9
HEL-75;GSR
HEL-S-153w;ORM1
SLPI
DBI
CTSG
KRT3
HEL-S-165mP;AKR1A1
CST5
HEL-S-1;YWHAB
AFM
LGALS7
HBG1;HBG2
BASP1
COL14A1
CHI3L2
PDCD6IP;DRIP4
HEL-S-8a;NIT2
CALML5
PYCARD
EFHD2
YWHAH
RAC2
HNRPK;HNRNPK
IL1RN
PRKCSH
RAP1B;RAP1A
ANXA6
KLK11
BST1
GGH
PLTP
RAD23B
ANXA11
RAN
AHSG
AP00000024146

Q0IIK2
ARPC4-TTLL3;ARPC4
ANP32A
UGDH
HEL-S-115;SH3BGRL
CPD
HEL-S-53e;HEL-9;ALDH1A1

HEL-76;CA2
DKFZp686I15212;IGHG3;FLJ00385
SERPINB2
SLC25A5;SLC25A6
CKM
HEL-S-7;ANXA5

FLJ00382
PYGB
PFKL
AZU1
HMGB2
EEF1G
CANX
GNPDA1
ME1
HEL-S-47e;ARHGDIA
MUC5AC
WFDC2
DKFZp686C15213
PLBD1
FERMT3
MUC7
RAB1B
S100A14
TRIM29
FOLR1
CGI-38;TPPP3
NCF2
HNRNPA1;HNRPA1;hCG_2020860;RP11-78J21.1;HNRNPA1L2
CS
ANPEP
COL6A1
PTPRC
LRRFIP1
NCF4
CYBB
HEXA
FASN
PSMA5
NAGLU;ufHSD2
HPRT1
PPP1CA;PPP1CC;PPP1CB
PSMB1
GFAP
HBD
CAPZA2
NCF1C;NCF1;NCF1B
RNASET2
HEL-S-94n;STIP1
FAM3B
PRKAR1A;DKFZp779L0468

HCLS1
HEL-S-108;TPM4
ATRN

TPP1
PRTN3
TBCA
PSMA4
FCN1;DKFZp781B1032
RBP4
CSTB
GSTM1
TACSTD2
FDPS
ACPP
PI3
AMY2B
YWHAQ
APEX1
S100A11;HEL-S-43
PSMB3
ACTA1;ACTC1;ACTA2;ACTG2
S100A12
TWF2
NCCRP1
TMPRSS11B
HOPX
BPIFA1
IL36A
VDAC2
HIST1H2BM;HIST1H2BN;HIST1H2BD;HIST1H2BK;HIST1H2BI;H2BFS;HIST1H2BC;HIST1H2BL;HIST1H2BH;HI
LGALS3;hCG_22119
NPC2
SDCBP
IMPA1
PSMA2
HEXB
GLRX
ERAP1;ARTS-1
HIST1H2AH;H2AFJ;HIST1H2AK;HIST1H2AJ;HIST2H2AC;HIST2H2AA3;HIST1H2AD;HIST1H2AG
RAB5B
TMPO
RPLP2
HMGB1;HMGB1P1;WUGSC:H_NH0244E06.1
HEL111;TTR
EEF1D
PCMT1
MGAM
ALAD
TXNDC17
PSMA3

ATP6V1B2
IGHG4
IGHV
A30
IGL@
LYPD3;DKFZp686D0114
EIF4A1
DCTN2;HEL-S-77
CAND1
GRB2
SERPINA6
FSCN1

GALNT6
GBE1
CNDP2
GCA
FLG
KRT84
TSN
CRYAB;HEL-S-101
RPS3
HEL-S-1a;PDXK
C5
MYL1
TRA1;HEL-S-125m;HSP90B1
HIST1H1B
PSMA1;HEL-S-275
S100P
MARCKS
HEL-S-10;BLVRB
CDA
ATP6V1A
HINT1
CCT8
EIF6
HEL-S-71;UBE2N
ARHGAP1
APOB48R;APOBR
PLEC
PPA1;HEL-S-66p
MYH14
S100A16
CTSZ
NQO2
GNB1;GNB2
UBE2L3
ACLY;ACLY variant protein
CAB39
RPS9
KLK12
KLK10

IL36RN;IL1F5
EHD1
PRCP
WARS
NUTF2
RAB3D
RAB14
STOM
CPNE3
GLTP
RPL6
0A1S6GKZ7;A0A1S5V2W8;A0A075MGT0;A0A1X9PWD3;A0A060VCZ2;A0A0B7MB11;U6BN77;Q5SS57;L8E
IGKV2-24;IGKV2D-24
CD177
FOLR3
GPX3
CNN2
TFF3
TPT1
IGHV4-61;VH4-34;VH4;IGM
PSMB9
APOE
SCGB1A1
SIL1

MTAP
BLVRA
TSTA3
ALDH7A1
ASAH1
FCGR3A
RAB27A
MAPRE1
NUDT5
MANBA
DNER
CCT4
CFI
ACAT2
OSTF1
MAN2B1
SFRP1
MYO1F variant protein;MYO1F;FLJ00395
ARPC3
GYG1
SLC9A3R1
HRG;DKFZp779H1622
FKBP4
KPNB1
TARS
FAM25G;FAM25C;FAM25A

HEL-S-274;ANXA4
C4BPA
HEL-S-124m;HSPA9
CCT2;HEL-S-100n

KRT15

KRT80
530;CON__A2A5Y0
SH3BGRL3;HEL-S-297
SERPING1
CD59
DSTN;HEL32
B2M
GLG1
APRT
ARPC5
CD5L
SCGB2A1
PGLYRP1
CLIC3
HEL-S-44;SOD1;SOD-1
EGF
ADH5
CKMT1A;CKMT1B
MMP9
ORM2
LMNB1
HEL-S-123m;ATP5A1
PON1
PSMB8;PSM8
S100A2
RPL12
RAB1;RNASE4
SPRR2A
CXCL5
RPIA
MTPN;DKFZp761E1322
DEFA3;DEFA1
FKBP1A;FKBP12-Exip2
TUBA4A
DCD
DSC1
PCBP1
CCT7
IGL@

KPRP
IGH@;IGHG1;DKFZp686N02209;DKFZp686H20196;DKFZp686K03196
PRR27
DKFZp686K18196
POF1B

RETN
IL36G
DBNL
PKP3
MYLPF
C9
CEACAM5;DKFZp781M2392
NAPA
MYL3
GMPPB
EEF1B2;LOC392793
NCL
FLJ22671;C2orf54
PLOD1
C9orf88;FAM129B
GAA
CANT1
RPL30
RAB21
GNS;DKFZp686E12166
CORO1C
TXNRD1
RPLP0;RPLP0P6
DDX39B;hCG_2005638;DDX39A;DKFZp547B159
MAPK14
HNRNPD;HNRPD
SPTBN1
PRR4
TMPRSS11A
IGHV3-49
IGHV3-72
MUC21

AGL
KRT6A
PRSS22
PTBP1

/981;A0A0X9T7P6

ADK
PEPD
PPP2CB;PPP2CA
PSMB2
V2-13;VL4;VL3L
KLK9
RPS27A;UBC;UBB;DKFZp434K0435;UbC;HEL112;UBA52
RPS6

HIST1H1E
TPD52
CD9
PPP2R1A
CASP14
CFHR1
HEL-S-70p;ATIC
GLOD4
CCT3
MVP
CEACAM7
RPN1
HADHB
LAMP2
CTSA;PPGB
QPCT
STMN2
TUBB;TUBB2A;TUBB2B;XTP3TPATP1
PODXL
PTMA;PTMAP7
APOD

SAA1
PTPNS1;SIRPA
ZNF185;DKFZp686B22130
TMOD3;DKFZp686E1899
AKR7A2;HEL-S-166mP
UQCRC2
EML2
CHAD
STX7
GMFG
VNN2
HEL-S-277;DDAH2
HPR
CSTA
APOA2
STATH
S100A6
MFI2
LGALS1

RAF3;RNASE2
CTSS
ERP29;HEL-S-107
PRDX3
IDH2
SLURP1
UFM1
YWHAG
RPS8
RPS18

RPL7A;RP-L7a
TMSB10
ARHG;RHOG
GLO1;HEL-S-74
LY6D
hCG_1773630;ADIRF
PRSS8

DKFZp686O2462;VPS35
ITLN1
V1-22
HIST2H3PS2
HEPHL1
DKFZp686G11190
DKFZp686C02220
IGK@

VSIG10L
LSR
IGL@
EPS8L1
MYDGF
DNHD1
SPRR2F
PROL1
DPP7

CARHSP1
SULT1A1;hCG_1993905;STP1;ST1A5;SULT1A4;SULT1A2;SULT1A3
RPL18
NADK
SDF4
APOC1
MB
XRCC6
LASP1
KRT14
CTSL1;CTSL
HMGN1;hCG_17955;hCG_1979072
OXSR1
CTNNB1
RPL15
DAG1
MBP
LBR
DISC1;TSNAX
HSPE1;EPFP1;HSPE1-MOB4
TKFC;DAK
RTN3
BANF1
RAB6A;RAB6B;RAB39A

SQRDL;hCG_2001986
RPLP1
CDC37;MBD5
ICAM3;hCG_2033729
HEL-S-167mP;CA3
SUMO2;SUMO3;SUMO4
FMOD
PABPC1;PABPC3
NDRG1;TRG14
PADI2
PTGES3
NACA;hCG_2016482;NACAP1
PA2G4
CKAP4
PAICS
LIM;PDLIM5
ASL

V3-4;IGLV8-61
IGHV4-4
IGKV2D-29;IGKV A18;IGKV2D-26
EPPK1
CAPNS1
IGHV3OR15-7
IGKV2-40;IGKV2D-28
VTA1
F11R
TPD52L2;DKFZp686A1765
FCGR3B;Fc-gamma receptor IIIB
HNRNPM;HNRPM;ORF

C7orf24;GGCT
KLK8
TNFSF13;hCG_2045906;TNFSF12-TNFSF13
V5-2;IGLV9-49
IGHV3-38
Z86;A0A0F7T0G6;A0A0J9YVU5;A0A068LRW6
DESC1;TMPRSS11E
V4-2;IGLV5-45
TPM1
ETFA
ATP2A2
CKB;HEL-S-29;HEL-211
FH
HSD17B4
TDD0

C4B

COMT
MPST
PSMB4
ST13;ST13P4;hCG_1990625;ST13P5
QDPR
IGHV3-15;VH87-2

5R2
VDAC1
AIF1
S;CDSN
ME2
ENSA
YI6;A0A1W6IYJ7
YJ6
YJ5;A0A1W6IYJ2
CD44
UBE2V2;TMEM189-UBE2V1;UBE2V1
V<kappa>1;IGKV1-5

VK3
BPI

CBX3
VPS26A
RPL13
EIF3G
C6
HNRNPC;hCG_1641229
DNAJC3
ARCN1;DKFZp686M09245
RPS19

RPS4X
RPL18A
RPS15A;hCG_1994130
DKFZp686H17246;CLEC3B
RAB32;RAB38
CMPK1;CMPK

VNN1;VNN3
SPINT1
SFTPB
TECR

PRSS27
HTRA1

CCT5;HEL-S-69
HADHA
PAFAH1B1
DKFZp686A01208;AP2B1;DKFZp781K0743;AP1B1
DNAJB1
PCBP2;PCBP3
TP53I3
ABHD14B;HEL-S-299
UQCRC1
LYPD5
STXBP2
RPS7
SEPT2
HEL180
SCAP2;SKAP2
RCSD1
DMKN
AP00000024466;CON__ENSEMBL:ENSBTAP00000024462
KRT75

KRT19

KRT73;KRT71

02070

FLG2

HRNR
KRTHB2;KRT82
DKFZp686P17171;SERBP1
PSCA
SHMT1
SPINK7
SKP1
TCP1
RPS2;OK/KNS-cl.7;rps2
CA6
IGH@
MYL2;MYL10
NDRG2
ZYX
HNRNPUL2-BSCL2;HNRNPUL2
EIF5A;EIF5A2;EIF5AL1
MIF
HN1

HDHD2;HEL-S-301
PLAUR

CUTA
BAG3
PRB2
SMR3B
FUCA1
TFF1
ALDH2
SERPIND1
EPHX1
H1F0
C8G
TPM2;TPM2b;HEL-S-273
IGL@
HEL71;MYH1;MYH4
IDE
HTN1
SPRR1B
scFv
RPS12
S100A4
PSMB6
MAN1A1
SPRR1A
ATP6V1E1;ATP6V1E2
RPL13A;RPL13a;RPL13AP3
BTD
SERPINB10
TUFM
GNG2
KRTDAP
RAB10
DNCL1;DYNLL1;DYNLL2
IGKC
PVRL1
NEDD8;NEDD8-MDP1
SCFD1
ACTBL2
V1-13
V2-19
V5-6;VLC8;IGLV4-69
IGL@
DKFZp686P15220
DKFZp686I04196;IGHG2;DKFZp686E23209
IGFL1
CD109
GBP6
DKFZp686M08189
DKFZp686K04218
GALNT5
IGL@
SNC73;DKFZp686J11235;IGHA1;DKFZp686G21220

MUC16
SCGB3A1
ain V-I region Daudi;Ig kappa chain V-I region DEE
CGREF1
ERP44
SPRR2G
LACRT
ABRACL
CRCT1
LAMTOR3
ENOPH1

A1X9YLN0;REV__Q81953;REV__Q81954;REV__Q9Q284;REV__Q9Q285;REV__Q81946;REV__Q81951;REV_

HEL-176
LTF

ST2H2BF;HIST2H2BE;HIST1H2BB;HIST1H2BJ;HIST1H2BO;HIST3H2BB

[illegible]9Q0;I7JHQ8;M4QFU4;A8K861;A0A1D0BQW3;A0A286MCF2;Q6F3E1;Q6IV50;Q8HWL6;Q9GJF5;U3MYE2;[illegible]

_Q81952;REV__Q81955;REV__O56933;REV__R9QCH2;REV__P89917;REV__P22424;REV__P88804;REV__A

R5AK04;U4Q5A9;F4NBP5;P30480;C6K6H9;N1NSZ5;A0A1D0BU97;Q7YQ88;G9DBH0;A0A286MCG3;J7HH0

A9JPG1;REV__Q4JJY7;REV__Q9Q283;REV__Q9Q282;REV__Q81950;REV__Q81937;REV__Q81938;REV__Q81

7;A1Z0M3;A0A286MCE9;P30462;A0A0B7MB28;P30460;P18463;S6EYQ3;A0A1W1B6C0;A0A1S6GL35;A4I

947;REV__Q81933;REV__Q81934;REV__Q81935;REV__Q81936;REV__Q81940;REV__Q81948;REV__Q819[illegible]

FRG0;A0A0U2X5S9;A0A090KF41;A0A1C3PHX5;A0A140HLF2;I2GAD8;U4N162;A0A0S2ILF3;A0A0S2IIS6;A0

49;REV__Q81941;REV__Q9Q286;REV__Q81944;REV__Q81945;REV__Q81942;REV__P26537

)A191XZP2;A0A141MJG8;A0A1G4HPR8;R4ZGR5;O19691;Q29637;Q1ZZH2;A0A0B7M9W0;I6QS32;Q2YHR

9;Q52QV7;V6E0U6;C0KXH2;C5J3Z7;A0A0B7MGZ1;R4ZGR3;A0A1K0J517;Q56H29;D5H3H6;A0A0H5CW8(

);A7XBN0;A0A110BIM8;A0A0X8XVK9;A0A0B7MH26;U6BN41;A0A0B7MHW2;A0A060VHG3;E3Q1J1;E3UP

B1;A0A0B7MI02;A5PI53;A7WPJ0;F0VRV8;Q56H30;A0A167RN89;E1Y6U0;E0WMY5;D6CIN1;D2KZ22;B2N[illegible]

J16;C8ZLL2;Q6IUU8;I7GGT2;R5A8X2;F4NBU2;A0A1A7G6U7;A0A1C3PHN4;A0A1C3PHM3;A7MAE0;A0A1C

3PHF6;A0A1C3PHK7;A0A1C3PHK4;Q4A1W0;A0A1C3PHG5;A0A1N7SXR8;A0A1C3PHG2;G9DBH1;A0A1C3I

PHE9;J7RMN3;Q3LAZ3;Q306J9;U6C266;A0A286MCF5;G5DSS2;A0A173ADG8;A0A1C3PHK8;A0A173ADA6;

;E7
